# DBU-Mediated Diastereoselective (3+2)-Cycloaddition of Isatin Ketonitrones and Coumarins to Construct Coumarin-Fused Spiropyrolidine Oxoindoles

**DOI:** 10.3390/molecules31081303

**Published:** 2026-04-16

**Authors:** Lan Ma, Qian Zhong, Zixin Zhang, Ruyi Zhou, Chunyan Long, Wanbing Wu, Sicheng Li, Qiao He, Guizhou Yue

**Affiliations:** 1 College of Science, Sichuan Agricultural University, Ya’an 625014, China; 2 Sichuan Jisheng Biopharmaceutical Co., Ltd., Leshan 614000, China; 3 The Yingjing County Emergency Management Agency, Ya’an 625200, China; 4 Agriculture and Rural Bureau of Daying Country, Suining 629300, China

**Keywords:** cycloaddition, ketonitrone, coumarin, spirooxoindole

## Abstract

The synthesis of novel dicyclic spiropyrrolidine oxoindole derivatives is described. This approach relies on a (3+2)-cycloaddition reaction between coumarins and isatin ketonitrone 1,3-dipoles, which were formed in situ by condensation of various substituted isatins with arylhydroxylamines. The corresponding pentacyclic products, featuring four contiguous stereocenters—including two quaternary carbon stereocenters fused within a single ring system—were obtained smoothly in moderate to excellent yields (22–98%), with high regioselectivity (*α* and *exo* type) and diastereoselectivity (>20:1 *dr*). Over 45 examples of the synthesized compounds were fully characterized using a range of spectroscopic techniques, including single-crystal X-ray diffraction, FTIR, NMR, and mass spectrometry.

## 1. Introduction

**Figure 1:**
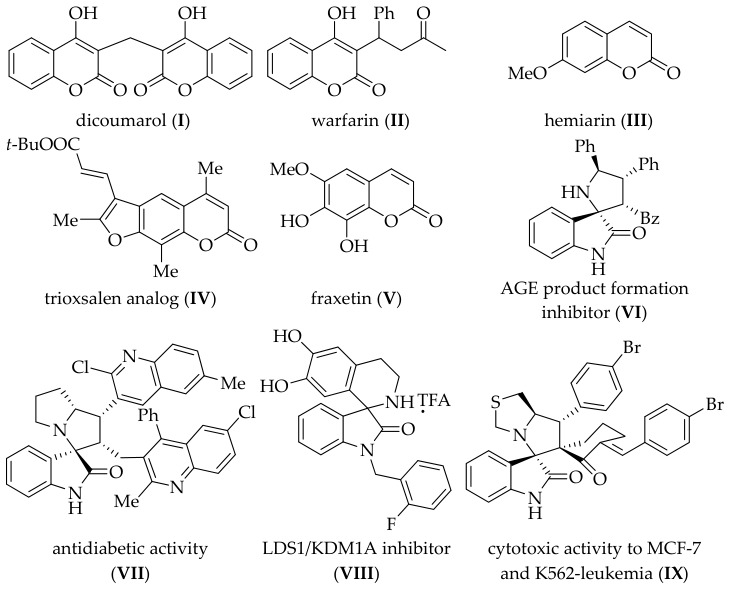
Representatives of natural and artificial coumarin and spirooxindole derivatives (**I**–**IX**).

Coumarins, a class of naturally occurring benzopyrone derivatives, are widely present in medicinal plants and have been isolated since 1820 [1,2,3,4,5,6,7,8]. Many of these compounds have demonstrated significant biological activities (anticancer, anti-HIV, antibacterial, anti-pathogen, anticoagulant, and antispasmodic activities…) [9,10,11,12,13,14,15,16,17,18,19,20]. For examples, a famous compound warfarin (**II**), derived from dicoumarol (**I**) (Figure 1), exhibits an anticoagulant activity, and was subsequently used as a rodenticide and a drug for prevention of cerebral thrombosis [21,22]. Compounds **III**–**V** also displayed considerable chemotherapeutic efficacy against mammary cancer induced by polycyclic aromatic hydrocarbons, potent inhibitory activity against soybean lipoxygenase (LOX), and significant anticancer activity against both bladder cancer and glioma, respectively [23,24,25,26]. In addition, versatile coumarin skeletons can serve as key components in organic optical devices after facile chemical modification [27,28,29,30]. The chemical syntheses of coumarins have been well developed by various methods, including common condensations/lactonizations, transition-metal-catalyzed reactions, and photoinduced organocatalytic methods [31,32,33,34,35,36,37,38]. Meanwhile, as important synthons, coumarins can take part in various chemical reactions, affording structurally more complex coumarin derivatives [39,40,41]. Spirooxindoles, on the other hand, are a class of tricyclic organic molecules featuring multiple contiguous quaternary or tertiary stereocenters and are commonly found in natural products and bioactive compounds [42,43,44,45,46,47,48,49,50,51,52,53,54,55,56,57,58,59,60,61,62,63,64,65]. Their unique structures and remarkable biological activities make them highly promising in the field of medicine, positioning them as potential drug candidates. For instance, compound **VI** inhibited an advanced glycation end (AGE) product formation (IC_50_ = 11.37 nM) [66], whereas compound **VII** showed inhibitory activity against the *α*-amylase and *α*-glucosidase enzymes (IC_50_ = 0.28 and 0.31 mg mL^−1^, respectively) [67]. Furthermore, the tetrahydroisoquinolinyl oxindole **VIII** showed a highly effective and selective inhibitory activity against histone lysine-specific demethylase 1 (LSD1) (IC_50_ = 42 nM) [68]. The spiropyrrolothiazolyl oxindole **IX** was discovered to exhibit in vitro activity against MCF-7 and K562 leukemia cancer cells, with IC_50_ values of 15.32 and 14.74 μM, respectively [69].

**Scheme 1:**
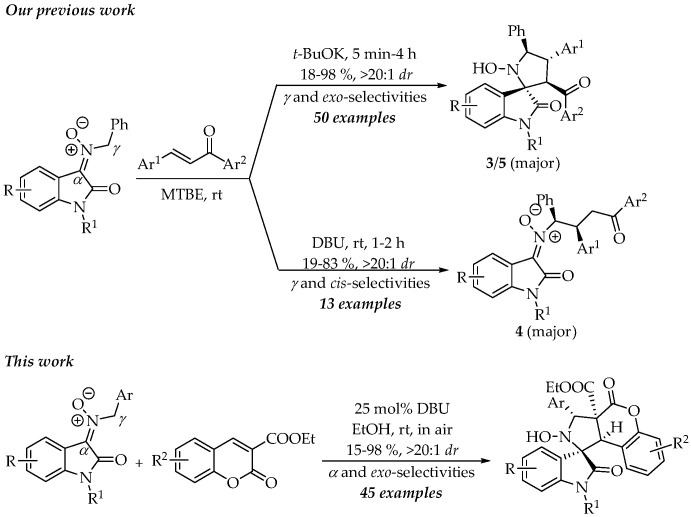
(3+2)-Cycloaddition and Michael addition of isatin ketonitrones with dipolarophiles.

Among the various spirooxindoles, spiropyrrolidine derivatives represent the most frequently encountered class. They exhibit a wide range of significant biological activities, which have captured considerable attention and interest from organic chemists worldwide [70,71,72,73,74,75,76,77,78,79,80,81,82]. Among the numerous synthetic routes to spiropyrrolidine oxindoles, the 1,3-dipolar cycloaddition between isatin-derived dipoles (e.g., azomethine ylides, azomethine imines, and nitrones) and dipolarophiles is regarded as one of the most practical and straightforward strategies for constructing the pyrrolidine ring [83,84,85,86,87,88,89,90,91,92,93,94,95,96,97,98,99,100,101,102,103,104,105,106,107,108,109,110,111,112]. As versatile synthons, nitrones have been extensively employed in various organic transformations to build complex molecular architectures [113,114,115,116,117,118,119,120,121,122,123,124,125,126,127,128,129,130,131,132,133,134,135,136,137,138,139,140,141,142,143]. Recently, we reported a rapid (3+2)-cycloaddition of isatin ketonitrone 1,3-dipoles with chalcones, which afforded novel dicyclic spiropyrrolidine oxindole derivatives in 18–98% yields with *dr* values > 20:1 in the presence of tBuOK (Scheme 1) [144]. Meanwhile, under DBU conditions, Michael adducts were obtained in 18–83% yields with *dr* values ranging from 1:1 to >20:1. As an extension of our investigations into the 1,3-dipolar cycloaddition of isatin ketonitrones, we attempted to treat isatin ketonitrone **1a** with coumarin **2a** in the presence of a base, anticipating the formation of pentacyclic spiropyrrolidine oxoindoles.

## 2. Results and Discussion

**Figure 2:**
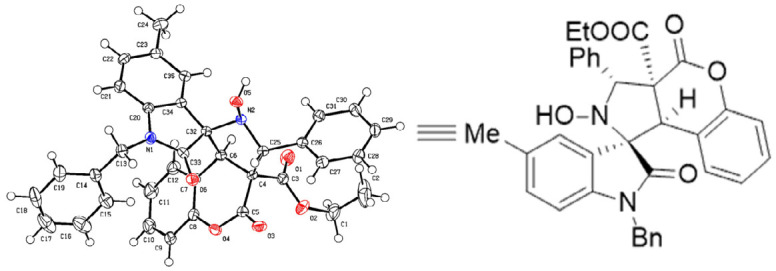
X-ray crystal structure of **3b** (CCDC: 2375731).

Prior to this study, *N*-Bn isatin ketonitrone **1a** and ethyl coumarin-3-carboxylate **2a** were chosen as the model substrates (Table 1). The reaction was initially carried out with DBU (25 mol%) in various protic and aprotic solvents, including H_2_O, EtOH, MTBE, DMF, THF, and DCM. The results showed that aprotic solvents failed to produce the desired cycloadduct **3a** at either room temperature or under heating (entries 1 and 2). Specifically, no reaction occurred in DCM, while a complex and intractable mixture formed in DMF. Fortunately, the protic polar solvent EtOH delivered **3a** in 70% yield (entry 4). The ^1^H NMR spectrum of **3a** exhibited two singlet signals at 6.39 and 4.70 ppm, corresponding to the two protons on the pyrrolidine ring. These data supported the nucleophilic attack of the α-carbon of isatin ketonitrone **1a** on the electrophilic carbon of the electron-deficient C=C bond of coumarin **2a**, leading to the cycloadduct with high *α*-diastereoselectivity. Moreover, the *exo*-configuration of **3a** was indirectly confirmed by single-crystal X-ray diffraction of compound **3b** (Figure 2). Meanwhile, 2D NOESY for compound **3b** showed that the two hydrogen atoms on the tetrahydropyrrole ring were in a *trans* configuration (see the Supplementary Information). Several alcohols, including MeOH, *i*PrOH, *n*BuOH, and *t*BuOH, were screened in an attempt to improve the yield of compound **3a**. However, all of them furnished lower yields than ethanol. Next, other organic and inorganic bases were screened using ethanol as the solvent. The inorganic bases (for instance, *t*BuOK, KOH, NaOH, and NaHCO_3_) were ineffective at rt and gave a complex reaction mixture under heating or reflux conditions. Although both EtONa and MeONa promoted the reaction smoothly, the conversion of **1a** remained too low. Among the organic bases tested, no reaction was observed with DMAP, DABCO, or Ph_3_P. By contrast, the use of other secondary and tertiary amines—including Et_2_NH, Bn_2_NH, TEA, Bn_3_N, *n*Pr_3_N, DIPEA, tri-*n*-amylamine, and tri-*n*-octylamine—led to only low conversion of **1a**, even when the reaction temperature was raised. Changing the amount of coumarin **2a** to 2.1 equiv. gave almost the same yield as that obtained with 1.0 equiv. of **2a**. Finally, the amount of DBU and the concentration of the reaction were investigated. A higher concentration (0.2 M) slightly increased the yield to 74%. Considering the above factors, the optimal reaction conditions for the 1,3-dipolar cycloaddition were established as isatin ketonitrone **1a** (1 equiv.), coumarin **2a** (1.05 equiv.), and base DBU (0.25 equiv.) in EtOH at rt for 5 h (entry 14).

After the optimal reaction conditions were established, a wide range of different substituted isatin ketonitrones were explored for this 1,3-dipolar cycloaddition. As outlined in Table 2, various substituent groups on isatin ketonitrones were well tolerated, furnishing the desired cycloadducts in 38–98% yields with >20:1 *dr* values, except in the cases of **3h**–**i**, **3o**, and **3v**. It is worth noting that the cycloaddition for the synthesis of **3a** was scaled up to 0.5 mmol and 5 mmol, giving the same yield. It is worth noting that the substituents attached to the benzene ring of isatin (see R^1^ in Table 2) show a significant effect on the yields of compounds **3a**–**3i**, demonstrating that isatins bearing electron-donating groups provided relatively higher yields than isatins containing electron-withdrawing groups. For example, a 5-methyl group installed on the benzene ring afforded the highest yield (98%) (entry 2), whereas strong electron-withdrawing groups (5-NO_2_ and 7-CF_3_) gave complex mixtures (entries 8 and 9). Changing the substituent groups (R = H, Me, Et, allyl, and propargyl) on the nitrogen atom of the isatin part of ketonitrones **1**, the corresponding cycloadducts were also consistently rendered in satisfactory yields (59–85%). Subsequently, when the R^2^ group (R^2^ = Ph) was replaced by various substituted phenyl groups, products **3p**–**u** were generated smoothly in moderate to good yields as a single isomer. However, only a trace amount of **3o** was obtained when R^2^ was a hydrogen atom. This may be attributed to the difficulty of the ketonitrone in forming the active C-N-C type 1,3-dipole intermediate in the presence of DBU, even with EtONa (entry 15). A similar result was also observed in the case of **3v** (entry 22). Next, based on the high yield of **3b**, these reactions were further investigated using substituted phenyl groups in place of the phenyl group at the R^2^ position, while R^1^ was a 5-methyl group. The results demonstrated that the corresponding yields were analogous to those of **3p**–**u** (entries 23–29). Finally, the substrate bearing a benzyl group on the nitrogen atom and aryl groups at the R^2^ position also underwent the 1,3-dipolar cycloaddition, although the yields were generally lower than those of the above examples, especially for **3ad** and **3ae**. From the above results, the yields were seemingly irrelevant to the electronic effects of substituents at the site of R^2^. However, the yield decreased as the steric hindrance of the substrate increased (entry 26).

**Figure 3:**
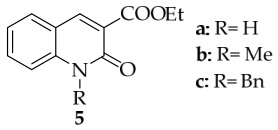
Other unsuccessful like-coumarins **5**.

In order to further explore the generality of the cycloaddition reaction, various substituted coumarins were studied. All results are illuminated in Table 3. First, coumarins bearing various groups on the benzene ring were employed to react with **1a**, showing that the electron effects and positions of the substituents had a significant impact on the yields and diastereoselectivities of the reaction. For example, 8-ethoxyl coumarin smoothly underwent the cycloaddition to give the desired product in 86% yield with >20:1 *dr*, whereas 7-methoxyl coumarin gave only 22% yield. Furthermore, when the 7-methoxy group was replaced by a 7-diethylamino group, the reaction gave rise to a complex mixture. All coumarins bearing electron-withdrawing or electron-deficient groups at the 6-position afforded the desired products in 67–82% yield. However, 6,8-dibromocoumarin gave only 30% yield, similar to that observed for 7-methoxylcoumarin. It is worth noting that 6-nitrocoumarin **2h** was subjected to the cycloaddition with **1a** in EtOH, and then the lactone ring of the cycloadduct was opened to yield compound **4g’** in 24% yield. After many experiments, the reaction between **2h** and **1a** was carried out in the non-nucleophilic solvent *t*-BuOH, leading to the desired product **4g** in good yield. Next, other coumarin 3-carboxylic esters **2k**–**n** were investigated. Both *i*-propyl and *n*-butyl esters **2l**–**m** were treated with **1a**, leading to the same and slightly lower yield compared with **2a**, whereas benzyl esters **2n** could lead to a higher yield than **2a**. Surprisingly, the yield for the reaction of methyl ester **2k** sharply decreased, maybe because of its instability under the current reaction conditions. Thirdly, when the entire ethyl ester group of **2a** was replaced by other groups, such as H, CN, X, Ac, Bz, carboxyl, and amide, all reactions were very messy. In addition, the analog 2-quinolinones **5** was also applied in this reaction, though the attempts were unsuccessful (Figure 3).

To develop an asymmetric version of this reaction, we screened several chiral catalysts—including quinine, quinidine, hydroquinidine, (*S*)-BINOL, and (*R*)-BINAP—in place of DBU in the model reaction of **1a** and **2a** in refluxing EtOH. Unfortunately, none of these catalysts proved suitable for the reaction, giving 0–26% yields and 0% *ee*.

**Scheme 2:**
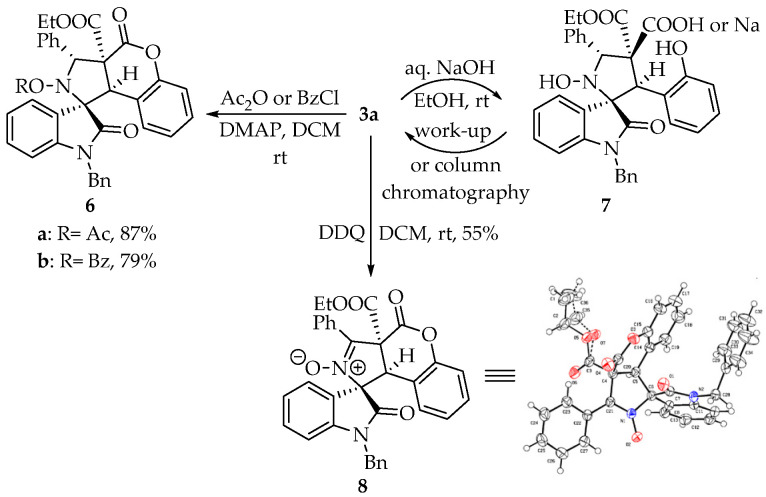
Transformation of **3a** and X-ray crystal structure of the compound **8** (CCDC: 2518523).

To demonstrate the synthetic utility of these spiropyrrolidine scaffolds, we carried out the derivatization of the model substrate **3a**. As illustrated in Scheme 2, the hydroxyl group of **3a** was protected with acetyl or benzoyl groups in the presence of DMAP, affording compounds **6a** and **6b** in 87% and 79% yields, respectively. Subsequently, saponification of **3a** using NaOH smoothly produced the desired carboxylic acid **7**. Unexpectedly, TLC analysis revealed that the carboxylic acid rapidly reconverted back to **3a**, even without acidification of the sodium salt of **7**, let alone during attempted isolation of **7** by column chromatography (using silica gel with 0.5% TEA in EtOAc/petroleum ether as eluent). When **3a** was exposed to DDQ in DCM, it was oxidized to the new nitrone **8** in 55% yield. The configuration of oxidation product **8** was unequivocally confirmed by single-crystal X-ray diffraction.

**Scheme 3:**
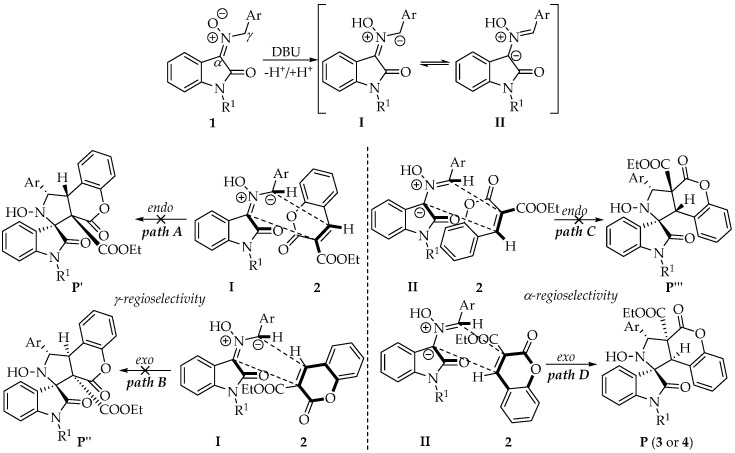
The plausible reaction mechanism.

Based on previously reported literature, our experimental results [144], and X-ray diffraction analysis, plausible reaction pathways were proposed in Scheme 3. Under basic conditions, ketonitrone **1** first isomerizes to the C-N-C type 1,3-dipole **I**, which is then converted into the other form **II**. In dipole **II**, the negative charge at the α position could be delocalized by both the benzene group and the carbonyl group, making **II** thermodynamically more stable than **I**. Therefore, the dipole **I** could not effectively compete with **II** in the cycloaddition reaction with coumarins **2** to give the *γ*-regioselective products **P’** or **P″** (paths A and B). On the other hand, the active dipole **II** reacted smoothly with coumarins **2** to afford the *α*-regioselective products **P** (compounds **3** or **4**), finally, either through a concerted 1,3-dipolar cycloaddition or a Michael-initiated ring closure (MIRC) annulation. Pathway D led to the formation of the *exo*-cycloadducts **3** or **4**, whereas pathway C gave the *endo*-products **P‴**. The observed predominance of *exo*-cycloadducts **P** could be rationalized by the *trans* arrangement of the two phenyl groups in the products, which minimized steric hindrance and electrostatic repulsion.

## 3. Materials and Methods

### 3.1. General Methods

All reactions were carried out without strict water-free and oxygen-free conditions. Unless otherwise noted, all reagents were obtained from commercial suppliers and used as received, without further purification. When reactions were performed in the presence of MeONa, EtONa, or *t*BuONa, the solvents (DCM, DCE, dioxane, and MTBE) were pre-dried over CaH_2_. Flash column chromatography was carried out using silica gel (200–300 mesh). Reaction progress was monitored by TLC. Visualization was achieved under a UV lamp (254 nm and 365 nm), by exposure to iodine vapor, or by staining with anisaldehyde. ^1^H, ^19^F and ^13^C NMR spectra were recorded on 400 MHz and 600 MHz spectrometers, with tetramethylsilane (TMS) as the internal standard. Chemical shifts were calibrated using residual solvent signals as internal references (CDCl_3_: ^1^H NMR δ 7.26, ^13^C NMR δ 77.16; DMSO-*d*_6_: ^1^H NMR δ 2.50, ^13^C NMR δ 39.52). IR spectra were acquired on an FT-IR spectrometer and are reported in wavenumbers (cm^−1^). High-resolution mass spectra (HRMS) were obtained using electrospray ionization (ESI). The following abbreviations are used for the multiplicities: s: singlet, d: doublet, t: triplet, sept: septet, m: multiplet, and br s: broad singlet for proton spectra. Coupling constants (*J*) are reported in Hertz (Hz).

### 3.2. Preparation of Intermediates

All *N*-substituted isatins were prepared by isatin (1.0 equiv.) and alkyl halides (1.5 equiv.) with NaH (2.0 equiv.) in DMF at 0 °C-rt [145]. All *O*-substituted benzyl hydroxylamine hydrochlorides were prepared in two steps, including the aldoxime reaction (1 equiv. aromatic aldehydes/1.2 equiv. hydroxylamine hydrochloride/2.0 equiv. NaOAc/1:1 EtOH:H_2_O and the reduction in aldoxime (1.0 equiv. aromatic aldoxime/1.2 equiv. NaBH_3_CN/10.0 equiv. con. HCl/MeOH/0 °C-rt) [146]. All isatin ketonitrones **1** were prepared by isatin or substituted isatins (1.0 equiv.) and *O*-benzyl or substituted benzyl hydroxylamine hydrochlorides (1.1 equiv.) in the presence of HOAc (2.0 equiv.) in MeOH at rt [147].

All coumarins **2** were prepared by adding a catalytic amount of piperidine (0.35 equiv.) to the equimolar mixture of salicyladehyde (1.0 equiv.) and malonate ester (3.2 equiv) in absolute ethanol or tetrahydrofuran, and the resulting homogeneous mixture was refluxed for 5–8 h. Completion of the reaction was confirmed by TLC; the whole reaction mixture was dispensed into crushed ice, and the resulting precipitate was filtered, dried, and recrystallized from methanol [148]. Ethyl quinoline-2(2*H*)-one-3-carboxylate derivatives were prepared according to previously reported literature. Diethyl malonate (1.5 equiv.) and NaHCO_3_ were added to a solution of 2-nitrobenzaldehyde (1.0 equiv) in acetic anhydride. The reaction mixture was stirred at 110 °C until 2-nitrobenzaidehyde was consumed. After cooling, the mixture was portioned between ethyl acetate and water. The organic layer was washed successively with water, 5% aq Na_2_CO_3,_ and brine. The solvent was removed in vacuo to give a brown residue, which was used for the next reaction without purification. Iron powder (4.0 equiv) was added to a pre-heated 85 °C solution of brown residue in acetic acid. The reaction mixture was maintained at 85 °C until the brown residue was consumed, cooled, and filtered through a Celite pad. The filtrate was evaporated in vacuo to leave a brown residue, which was purified by column chromatography to give the corresponding compounds **5a** [149,150]. Potassium carbonate (1.2 equiv.) was added to a stirred solution of **5a** (1.0 equiv.) in CH_3_CN at 0 °C. After stirring for 30 min, halogen (methyl iodide or benzyl bromide) (1.2 equiv.) was added dropwise to the mixture. The reaction mixture was stirred at room temperature until **5a** disappeared. The reaction mixture was filtered. The filtrate was evaporated in vacuo to leave a residue, which was purified by column chromatography to give the corresponding compounds **5b** or **5c** [151].

### 3.3. General Procedure for Condition Optimization

A 10 mL tube was charged with isatin ketonitrone **1a** (0.2 mmol, 1.0 equiv.), coumarin **2a** (0.21–0.22 mmol, 1.05–1.1 equiv.), base (0.05–0.2 mmol, 25–100 mol%), and solvent (1–4 mL). After the completion of the reaction, the reaction mixture was directly concentrated under vacuum. The residue was purified by flash silica gel chromatography eluted with EtOAc:PE (1:12 to 1:2, *v*/*v*) to afford the corresponding product **3a**.

### 3.4. General Procedure for Typical Procedure for Cycloaddition

A 25 mL tube was charged with isatin ketonitrone 1 (0.5 mmol, 1.0 equiv.), coumarin 2 (0.525 mmol, 1.05 equiv.), DBU (0.125 mmol, 25 mol%), and EtOH/*t*-BuOH (2.5 mL). The reaction mixture was stirred at rt and monitored by TLC. After the completion of the reaction, the reaction mixture was directly concentrated under vacuum. The residue was purified by flash silica gel chromatography eluted with EtOAc:PE (1:12 to 1:2, *v*/*v*) to afford the corresponding products **3**.

### 3.5. The Phenomenon of the Reaction and TLC

**Figure 4:**
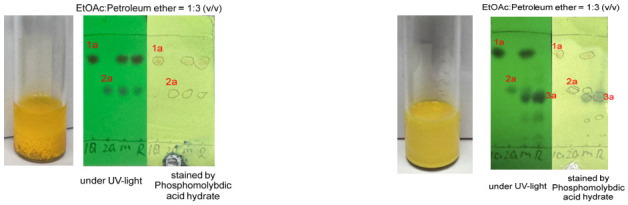
Before adding DBU (**left**) and after adding DBU (**right**).

Figure 4 illustrates the reaction of **1a** and **2a** under DBU conditions, as well as the TLC results of the starting materials (**1a**/**2a**) and product **3a**.

### 3.6. Derivatization of ***3a***

To a solution of **3a** (280 mg, 0.5 mmol, 1.0 equiv.) in DCM (3 mL) was added DMAP (18 mg, 0.15 mmol, 0.3 equiv.) and Ac_2_O (70 μL, 0.75 mmol, 1.5 equiv.) at rt. The mixture was stirred at rt for 18 h before it was quenched with a saturated NaHCO_3_ solution (10 mL). The aqueous solution was extracted with EtOAc (3 × 10 mL). The combined organic layers were dried over Na_2_SO_4_, filtered, and concentrated under reduced pressure. The residue was purified by flash column chromatography on silica gel (petroleum ether: EtOAc = 8:1 to 2:1) to provide the acetate **6a** in 87% yield.

To a solution of **3a** (280 mg, 0.5 mmol, 1.0 equiv.) in DCM (3 mL) was added DMAP (112 mg, 1.0 mmol, 2.0 equiv.) and BzCl (145 μL, 1.25 mmol, 2.5 equiv.) at rt. The mixture was stirred at rt for 21 h before it was quenched with a saturated NaHCO_3_ solution (10 mL). The aqueous solution was extracted with EtOAc (3 × 10 mL). The combined organic layers were dried over Na_2_SO_4_, filtered, and concentrated under reduced pressure. The residue was purified by flash column chromatography on silica gel (petroleum ether: EtOAc = 8:1 to 6:1) to furnish the benzoate **6b** in 79% yield.

To a solution of **3a** (280 mg, 0.5 mmol, 1.0 equiv.) in DCM (5 mL) was added DDQ (170 mg, 0.75 mmol, 1.5 equiv.). The mixture was stirred at rt for 3 h before it was quenched with a saturated Na_2_SO_3_ solution (15 mL). The aqueous solution was extracted with DCM (3 × 10 mL). The combined organic layers were dried over Na_2_SO_4_, filtered, and concentrated under reduced pressure. The residue was washed with methanol to give the pure product **8** in 55% yield.

### 3.7. Data for All New Compounds


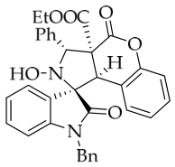

*(±)-Ethyl (1R,3R,3aS,9bR)-1′-benzyl-2-hydroxy-2′,4-dioxo-3-phenyl-2,3-dihydro-4H-spiro[chromeno[3,4-c]pyrrole-1,3′-indoline]-3a(9bH)-carboxylate* (**3a**). 207.4 mg, 74%, a white solid, >20:1 *dr*, mp: 182.9–184.1 °C; IR (thin film): *ν*_max_ 3470, 3061, 3032, 2977, 2911, 1760, 1732, 1615, 1491, 1369, 1228, 1212, 1179, 768, 732, 610, 532 cm^−1^; ^1^H NMR (400 MHz, DMSO-*d*_6_) δ 8.54 (s, 1H), 7.82 (d, *J* = 6.8 Hz, 1H), 7.72 (d, *J* = 7.6 Hz, 2H), 7.40 (ψt, *J* = 7.4 Hz, 3H), 7.34–7.26 (m, 3H), 7.14–7.11 (m, 2H), 7.05 (ψt, *J* = 7.4 Hz, 2H), 6.90 (ψt, *J* = 7.4 Hz, 1H), 6.69 (d, *J* = 7.2 Hz, 1H), 6.53 (d, *J* = 7.2 Hz, 2H), 6.38 (d, *J* = 7.52 Hz, 1H), 5.76 (s, 1H), 4.88 (d, *J* = 16.4 Hz, 1H), 4.68 (s, 1H), 4.51 (d, *J* = 16.0 Hz, 1H), 3.61–3.49 (m, 2H), 0.65 (t, *J* = 7.0 Hz, 3H); ^13^C NMR (100 MHz, DMSO-*d*_6_): δ 175.1, 167.2, 164.6, 150.8, 144.1, 138.2, 135.7, 130.6, 128.9, 128.4, 128.2, 128.1, 127.4, 126.7, 126.4, 125.0, 124.9, 123.7, 117.5, 115.2, 109.9, 77.4, 72.4, 63.0, 59.3, 47.0, 42.6, 13.5 (Two carbon atoms are missing in the ^13^C NMR spectrum. The reason may be that the NMR signals of two carbon atoms of the benzene ring overlap with those of other carbon atoms of the benzene ring.); HRMS (ESI): *m*/*z* calcd for C_34_H_28_N_2_O_6_Na [M+Na]^+^ 583.1845, found 583.1836.

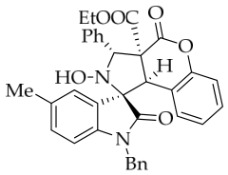

*(±)-Ethyl (1R,3R,3aS,9bR)-1′-benzyl-2-hydroxy-5′-methyl-2′,4-dioxo-3-phenyl-2,3-dihydro-4H-spiro[chromeno[3,4-c]pyrrole-1,3′-indoline]-3a(9bH)-carboxylate* (**3b**). 281.0 mg, 98%, a white solid, >20:1 *dr*, mp: 185.3–186.6 °C; IR (thin film): *ν*_max_ 3479, 3377, 3065, 3032, 2982, 2919,1767, 1728, 1705, 1496, 1369, 1227, 1174, 808, 765, 527 cm^−1^; ^1^H NMR (600 MHz, CDCl_3_) δ 7.84 (d, *J* = 7.8 Hz, 1H), 7.60 (s, 1H), 7.40 (ψt, *J* = 7.6 Hz, 2H), 7.32 (t, *J* = 6.8 Hz, 1H), 7.27 (t, *J* = 7.4 Hz, 1H), 7.11 (ψt, *J* = 7.2 Hz, 1H), 7.09–7.06 (m, 2H), 7.04 (ψt, *J* = 7.8 Hz, 2H), 6.80 (ψt, *J* = 7.4 Hz, 1H), 6.56 (d, *J* = 7.8 Hz, 2H), 6.44 (d, *J* = 7.8 Hz, 2H), 6.11 (s, 1H), 5.01 (d, *J* = 16.2 Hz, 1H), 4.78 (s, 1H), 4.72 (s, 1H), 4.35 (d, *J* = 15.6 Hz, 1H), 3.69–3.64 (m, 1H), 3.60–3.55 (m, 1H), 2.46 (s, 3H), 0.76 (t, *J* = 7.2 Hz, 3H); ^13^C NMR (150 MHz, DMSO-*d*_6_): δ 174.4, 167.0, 164.6, 151.1, 141.8, 137.4, 135.0, 133.0, 130.8, 129.6, 128.9, 128.6, 128.2 (2C), 128.1, 127.2, 126.7, 125.8, 124.8, 124.2, 117.6, 114.9, 109.6, 72.51, 63.0, 59.1, 46.9, 43.4, 21.3, 13.3; HRMS (ESI): *m*/*z* calcd for C_35_H_30_N_2_O_6_Na [M+Na]^+^ 597.2002, found 597.1976.

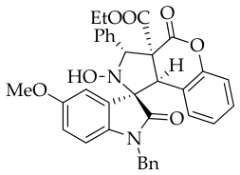

*(±)-Ethyl (1R,3R,3aS,9bR)-1′-benzyl-2-hydroxy-5′-methoxy-2′,4-dioxo-3-phenyl-2,3-dihydro-4H-spiro[chromeno[3,4-c]pyrrole-1,3′-indoline]-3a(9bH)-carboxylate* (**3c**). 242.6 mg, 82%, a white solid, >20:1 *dr*, mp: 176.3–177.9 °C; IR (thin film): *ν*_max_ 3476, 3067,3026, 2920, 2841, 1770, 1707, 1495, 1435, 1353, 1247, 1171, 1019, 1005, 763, 704 cm^−1^; ^1^H NMR (400 MHz, DMSO-*d*_6_) δ 8.51 (s, 1H), 7.72 (d, *J* = 7.2 Hz, 2H), 7.40–7.38 (m, 4H), 7.31 (t, *J* = 7.0 Hz, 1H), 7.12 (d, *J* = 8.4 Hz, 2H), 7.05 (ψt, *J* = 7.2 Hz, 2H), 6.93 (ψt, *J* = 7.6 Hz, 1H), 6.88 (dd, *J* = 8.8, 1.6 Hz, 1H), 6.59 (d, *J* = 8.4 Hz, 1H), 6.51 (d, *J* = 7.6 Hz, 2H), 6.47 (d, *J* = 7.6 Hz, 1H), 5.74 (s, 1H), 4.85 (d, *J* = 16.0 Hz, 1H), 4.68 (s, 1H), 4.46 (d, *J* = 16.0 Hz, 1H), 3.85 (s, 3H), 3.62–3.46 (m, 2H), 0.65 (t, *J* = 7.0 Hz, 3H). ^13^C NMR (100 MHz, DMSO-*d*_6_): δ 174.8, 167.2, 164.6, 156.5, 150.8, 138.3, 137.4, 135.8, 130.6, 128.9, 128.8, 128.6, 128.2, 128.1, 127.7, 127.4, 126.7, 124.9, 117.4, 115.3, 114.9, 112.0, 110.4, 77.7, 72.4, 62.9, 59.3, 56.2, 47.0, 42.7, 13.5; HRMS (ESI): *m*/*z* calcd for C_35_H_30_N_2_O_7_Na [M+Na]^+^ 613.1951, found 613.1946.

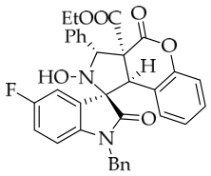

*(±)-Ethyl (1R,3R,3aS,9bR)-1′-benzyl-5′-fluoro-2-hydroxy-2′,4-dioxo-3-phenyl-2,3-dihydro-4H-spiro[chromeno[3,4-c]pyrrole-1,3′-indoline]-3a(9bH)-carboxylate* (**3d**). 236.3 mg, 82%, a white solid, >20:1 *dr*, mp: 188.1–189.0 °C; IR (thin film): *ν*_max_ 3469, 3065, 3039, 2921, 1767, 1708, 1492, 1370, 1355, 1249, 1172, 767, 705, 609, 557 cm^−1^; ^1^H NMR (400 MHz, DMSO-*d*_6_) δ 8.59 (s, 1H), 7.77–7.71 (m, 3H), 7.42–7.38 (m, 3H), 7.32 (t, *J* = 6.8 Hz, 1H), 7.20–7.12 (m, 3H), 7.05 (ψt, *J* = 7.4 Hz, 2H), 6.94 (ψt, *J* = 7.4 Hz, 1H), 6.68 (dd, *J* = 8.2, 3.8 Hz, 1H), 6.51 (d, *J* = 7.6 Hz, 3H), 5.73 (s, 1H), 4.88 (d, *J* = 16.4 Hz, 1H), 4.73 (s, 1H), 4.50 (d, *J* = 16.0 Hz, 1H), 3.62–3.48 (m, 2H), 0.65 (t, *J* = 7.0 Hz, 3H). ^13^C NMR (100 MHz, DMSO-*d*_6_): δ 175.0, 167.0, 164.6, 150.8, 140.3, 138.2, 135.5, 130.7, 128.9, 128.6, 128.4, 128.3, 128.2, 127.5, 126.7, 125.0, 117.5, 117.1, 116.8, 115.0, 113.4, 113.2, 110.8 (*J* = 31.6 Hz), 77.7 (*J* = 4.0 Hz), 72.4, 62.9, 59.3, 46.8, 42.7, 13.4; ^19^F NMR (376 MHz, DMSO-*d*_6_): −119.7; HRMS (ESI): *m*/*z* calcd for C_34_H_27_N_2_O_6_FNa [M+Na]^+^ 601.1751, found 601.1757.

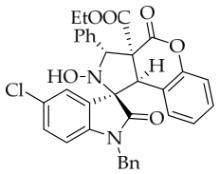

*(±)-Ethyl (1R,3R,3aS,9bR)-1′-benzyl-5′-chloro-2-hydroxy-2′,4-dioxo-3-phenyl-2,3-dihydro-4H-spiro[chromeno[3,4-c]pyrrole-1,3′-indoline]-3a(9bH)-carboxylate* (**3e**). 221.1 mg, 74%, a white solid, >20:1 *dr*, mp: 189.7–191.1 °C; IR (thin film): *ν*_max_ 3469, 3067, 3037, 2985, 1765, 1734, 1486, 1354, 1256, 1170, 767, 704, 693, 550 cm^−1^; ^1^H NMR (400 MHz, DMSO-*d*_6_) δ 8.63 (s, 1H), 7.91 (s, 1H), 7.91 (s, 1H), 7.20 (d, *J* = 7.2 Hz, 2H), 7.43–7.38 (m, 4H), 7.20 (d, *J* = 7.2 Hz, 2H), 7.32 (t, *J* = 7.2 Hz, 1H), 7.15–7.12 (m, 2H), 7.06 (ψt, *J* = 7.4 Hz, 2H), 6.96 (ψt, *J* = 7.4 Hz, 1H), 6.71 (d, *J* = 8.4 Hz, 1H), 6.50 (ψd, *J* = 6.8 Hz, 3H), 5.72 (s, 1H), 4.88 (d, *J* = 16.0 Hz, 1H), 4.75 (s, 1H), 4.51 (d, *J* = 16.4 Hz, 1H), 3.62–3.49 (m, 2H), 0.64 (t, *J* = 7.2 Hz, 3H). ^13^C NMR (100 MHz, DMSO-*d*_6_): δ 174.9, 167.0, 164.6, 150.8, 143.0, 138.1, 135.4, 130.7, 130.6, 128.9, 128.6, 128.5, 128.2, 127.9, 127.5, 126.7, 125.4, 125.0, 117.5, 115.0, 111.4, 77.6, 72.4, 63.0, 59.3, 46.8, 42.7, 13.5 (Two carbon atoms are missing in the ^13^C NMR spectrum. The reason may be that the NMR signals of two carbon atoms of benzene ring overlap with those of other carbon atoms of benzene ring); HRMS (ESI): *m*/*z* calcd for C_34_H_27_N_2_O_6_ClNa [M+Na]^+^ 617.1455, found 617.1447.

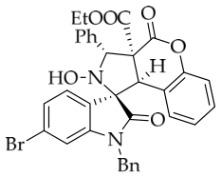

*(±)-Ethyl (1R,3R,3aS,9bR)-1′-benzyl-6′-bromo-2-hydroxy-2′,4-dioxo-3-phenyl-2,3-dihydro-4H-spiro[chromeno[3,4-c]pyrrole-1,3′-indoline]-3a(9bH)-carboxylate* (**3f**). 185.8 mg, 58%, a white solid, >20:1 *dr*, mp: 193.7–194.5 °C; IR (thin film): *ν*_max_ 3367, 3067, 2924, 2995, 2852, 1754, 1716, 1615, 1491, 1454, 1228, 1183, 761, 701, 698, 651, 511 cm^−1^; ^1^H NMR (600 MHz, DMSO-*d*_6_) δ 8.59 (s, 1H), 7.79 (d, *J* = 9.4 Hz, 1H), 7.70 (d, *J* = 7.8 Hz, 2H), 7.48 (dd, *J* = 8.1, 1.5 Hz, 1H), 7.43–7.38 (m, 3H), 7.32 (t, *J* = 7.5 Hz, 1H), 7.15 (t, *J* = 7.5 Hz, 1H), 7.14 (d, *J* = 7.8 Hz, 1H), 7.07 (ψt, *J* = 7.8 Hz, 2H), 6.98–6.95 (m, 2H), 6.53 (d, *J* = 7.8 Hz, 2H), 6.48 (d, *J* = 6.6 Hz, 1H), 5.71 (s, 1H), 4.87 (d, *J* = 16.2 Hz, 1H), 4.70 (s, 1H), 4.57 (d, *J* = 16.2 Hz, 1H), 3.60–3.45 (m, 2H), 0.65 (t, *J* = 7.2 Hz, 3H). ^13^C NMR (100 MHz, DMSO-*d*_6_): δ 175.1, 167.0, 164.6, 150.8, 145.7, 138.0, 135.4, 130.8, 129.0, 128.9, 128.5, 128.2, 127.6, 127.1, 126.7, 126.5, 125.7, 125.1, 123.4, 117.6, 114.9, 112.9, 77.2, 72.4, 63.0, 59.3, 46.7, 42.6, 13.5 (One carbon atom is missing in the ^13^C NMR spectrum. The reason may be that the NMR signals of one carbon atom of benzene ring overlap with those of other carbon atoms of benzene ring); δ HRMS (ESI): *m*/*z* calcd for C_34_H_27_N_2_O_6_BrNa [M+Na]^+^ 661.0950, found 661.0952.

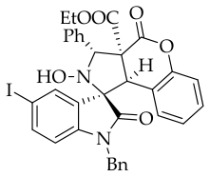

*(±)-Ethyl (1R,3R,3aS,9bR)-1′-benzyl-5′-iodo-2-hydroxy-2′,4-dioxo-3-phenyl-2,3-dihydro-4H-spiro[chromeno[3,4-c]pyrrole-1,3′-indoline]-3a(9bH)-carboxylate* (**3g**). 175.7 mg, 51%, a white solid, >20:1 *dr*, mp: 184.4–185.4 °C; IR (thin film): *ν*_max_ 3413, 3067, 2983, 2929, 2852, 1754, 1613, 1351, 1262, 1228, 1169, 1029, 808, 767, 701, 608, 534 cm^−1^; ^1^H NMR (600 MHz, DMSO-*d*_6_) δ 8.61 (s, 1H), 8.13 (d, *J* = 1.2 Hz, 1H), 7.72 (d, *J* = 7.2 Hz, 2H), 7.68 (dd, *J* = 8.4, 1.8 Hz, 1H), 7.42 (dd, *J* = 7.2, 1.2 Hz, 1H), 7.40 (ψt, *J* = 7.8 Hz, 2H), 7.32 (t, *J* = 7.5 Hz, 1H), 7.13 (t, *J* = 7.2 Hz, 1H), 7.05 (ψt, *J* = 7.5 Hz, 2H), 6.96 (td, *J* = 7.5, 0.9 Hz, 1H), 6.54 (d, *J* = 8.4 Hz, 1H), 6.50 (d, *J* = 7.2 Hz, 2H), 6.50 (ψt, *J* = 7.5 Hz, 1H), 5.72 (s, 1H), 4.87 (d, *J* = 16.2 Hz, 1H), 4.72 (s, 1H), 4.49 (d, *J* = 16.2 Hz, 1H), 3.59 (dq, *J* = 10.8, 7.2 Hz, 1H), 3.53 (dq, *J* = 10.8, 7.2 Hz, 1H), 0.64 (t, *J* = 7.2 Hz, 3H). ^13^C NMR (100 MHz, DMSO-*d*_6_): δ 174.6, 167.0, 164.6, 150.8, 143.9, 139.2, 138.1, 135.3, 133.4, 130.7, 128.9 (2C), 128.5, 128.2 (2C), 127.5, 126.7, 125.0, 117.5, 115.0, 112.3, 87.1, 77.4, 72.4, 63.0, 59.3, 46.8, 42.6, 13.5 (One carbon atom is missing in the ^13^C NMR spectrum. The reason may be that the NMR signals of one carbon atom of benzene ring overlap with those of other carbon atoms of benzene ring); HRMS (ESI): *m*/*z* calcd for C_34_H_27_N_2_O_6_INa [M+Na]^+^ 709.0811, found 709.0793.

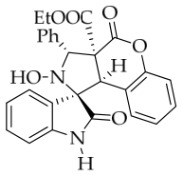

(±)-*Ethyl (1R,3R,3aS,9bR)-2-hydroxy-2′,4-dioxo-3-phenyl-2,3-dihydro-4H-spiro[chromeno[3,4-c]pyrrole-1,3′-indoline]-3a(9bH)-carboxylate* (**3j**). 178.7 mg, 76%, a white solid, >20:1 *dr*, mp: 186.3–187.4 °C; IR (thin film): *ν*_max_ 3367, 3283, 3057, 3043, 2995, 2935, 1778, 1714, 1620, 1498, 1351, 1257, 1228, 1181, 1019, 763, 741, 598, 567 cm^−1^; ^1^H NMR (400 MHz, DMSO-*d*_6_) δ 10.27 (s, 1H), 8.40 (s, 1H), 7.71 (t, *J* = 8.8 Hz, 1H), 7.70 (d, *J* = 8.4 Hz, 2H), 7.40–7.21 (m, 6H), 7.07 (d, *J* = 8.0 Hz, 1H), 6.88 (ψt, *J* = 7.4 Hz, 1H), 6.75 (d, *J* = 7.6 Hz, 1H), 6.36 (d, *J* = 7.6 Hz, 1H), 5.70 (s, 1H), 4.55 (s, 1H), 3.59–3.47 (m, 2H), 0.64 (t, *J* = 7.0 Hz, 3H). ^13^C NMR (100 MHz, DMSO-*d*_6_): δ 176.7, 167.3, 164.6, 150.8, 143.9, 138.4, 130.6, 130.3, 128.9, 128.2, 128.1, 128.0, 127.2, 125.0, 124.7, 122.9, 117.3, 115.6, 110.3, 77.5, 72.3, 62.9, 59.2, 47.0, 13.5; HRMS (ESI): *m*/*z* calcd for C_27_H_22_N_2_O_6_Na [M+Na]^+^ 493.1376, found 493.1339.

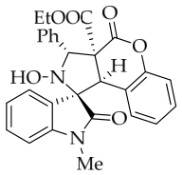

*(±)-Ethyl (1R,3R,3aS,9bR)-2-hydroxy-1′-methyl-2′,4-dioxo-3-phenyl-2,3-dihydro-4H-spiro[chromeno[3,4-c]pyrrole-1,3′-indoline]-3a(9bH)-carboxylate* (**3k**). 193.1 mg, 80%, a white solid, >20:1 *dr*, mp: 172.1–172.8 °C; IR (thin film): *ν_max_* 3477, 3060, 3038, 2985, 2944, 1765, 1711, 1617, 1498, 1378, 1250, 1228, 1174, 1095, 771, 754, 699, 610, 540 cm^−1^; ^1^H NMR (400 MHz, DMSO-*d*_6_) δ 8.43 (s, 1H), 7.79 (d, *J* = 7.2 Hz, 1H), 7.70 (d, *J* = 7.2 Hz, 2H), 7.45 (t, *J* = 7.6 Hz, 1H), 7.38 (ψt, *J* = 7.2 Hz, 2H), 7.32 (ψt, *J* = 7.4 Hz, 1H), 7.30 (ψt, *J* = 7.0 Hz, 1H), 7.24 (ψt, *J* = 7.6 Hz, 1H), 7.06 (d, *J* = 8.0 Hz, 1H), 6.97 (d, *J* = 8.0 Hz, 1H), 6.85 (ψt, *J* = 7.4 Hz, 1H), 6.31 (d, *J* = 7.6 Hz, 1H), 5.70 (s, 1H), 4.60 (s, 1H), 3.59–3.47 (m, 2H), 2.82 (s, 3H), 0.64 (t, *J* = 7.0 Hz, 3H). ^13^C NMR (100 MHz, DMSO-*d*_6_): δ 174.8, 167.2, 164.6, 150.6, 145.3, 138.2, 130.7, 130.3, 128.9, 128.2, 128.1, 127.9, 126.4, 124.7, 124.6, 123.6, 117.4, 115.3, 109.2, 77.3, 72.3, 62.9, 59.2, 47.0, 26.0, 13.5; HRMS (ESI): *m*/*z* calcd for C_28_H_24_N_2_O_6_Na [M+Na]^+^ 507.1532, found 507.1527.

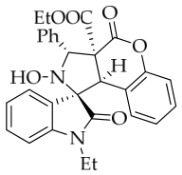

*(±)-Ethyl (1R,3R,3aS,9bR)-1′-ethyl-2-hydroxy-2′,4-dioxo-3-phenyl-2,3-dihydro-4H-spiro[chromeno[3,4-c]pyrrole-1,3′-indoline]-3a(9bH)-carboxylate* (**3l**). 195.4 mg, 78%, a white solid, >20:1 *dr*, mp: 170.4–171.0 °C; IR (thin film): *ν_max_* 3449, 3063, 2981, 2931, 1764, 1732, 1711, 1616, 1491, 1468, 1455, 1373, 1258, 1229, 1178, 763, 750, 610, 551 cm^−1^; ^1^H NMR (400 MHz, DMSO-*d*_6_) δ 8.44 (s, 1H), 7.77 (d, *J* = 7.2 Hz, 1H), 7.71 (d, *J* = 7.2 Hz, 2H), 7.44 (t, *J* = 7.6 Hz, 1H), 7.39 (ψt, *J* = 7.4 Hz, 2H), 7.31 (ψt, *J* = 7.2 Hz, 2H), 7.24 (ψt, *J* = 7.6 Hz, 1H), 7.06 (d, *J* = 8.0 Hz, 1H), 7.00 (d, *J* = 7.6 Hz, 1H), 6.83 (ψt, *J* = 7.4 Hz, 1H), 6.22 (d, *J* = 7.6 Hz, 1H), 5.74 (s, 1H), 4.54 (s, 1H), 3.55–3.49 (m, 3H), 3.32–3.27 (m, 1H), 0.65 (t, *J* = 7.0 Hz, 3H), 0.49 (t, *J* = 6.8 Hz, 3H). ^13^C NMR (100 MHz, DMSO-*d*_6_): δ 174.6, 167.2, 164.6, 150.7, 144.0, 138.3, 130.7, 130.3, 128.9, 128.2, 128.1, 127.8, 126.7, 124.8, 124.5, 123.5, 117.1, 115.1, 109.3, 77.3, 72.5, 62.9, 59.2, 47.7, 34.0, 13.5, 12.0; HRMS (ESI): *m*/*z* calcd for C_29_H_26_N_2_O_6_Na [M+Na]^+^ 521.1689, found 521.1683.

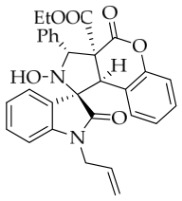

*(±)-Ethyl (1R,3R,3aS,9bR)-1′-allyl-2-hydroxy-2′,4-dioxo-3-phenyl-2,3-dihydro-4H-spiro[chromeno[3,4-c]pyrrole-1,3′-indoline]-3a(9bH)-carboxylate* (**3m**). 217.6 mg, 85%, a white solid, >20:1 *dr*, mp: 168.4–169.9 °C; IR (thin film): *ν_max_* 3448, 3092, 3059, 2982, 2915, 1762, 1734, 1715, 1618, 1492, 1469, 1369, 1257, 1234, 1181, 767, 749, 732, 702, 608, 527 cm^−1^; ^1^H NMR (400 MHz, DMSO-*d*_6_) δ 8.48 (s, 1H), 7.80 (d, *J* = 6.8 Hz, 1H), 7.70 (d, *J* = 7.2 Hz, 2H), 7.41 (t, *J* = 8.2 Hz, 1H), 7.40 (ψt, *J* = 7.8 Hz, 2H), 7.31 (ψt, *J* = 6.8 Hz, 2H), 7.26 (ψt, *J* = 7.6 Hz, 1H), 7.06 (d, *J* = 8.0 Hz, 1H), 6.87 (d, *J* = 7.6 Hz, 1H), 6.85 (ψt, *J* = 7.6 Hz, 1H), 6.34 (d, *J* = 7.2 Hz, 1H), 5.72 (s, 1H), 5.38–5.29 (m, 1H), 4.70 (d, *J* = 10.0 Hz, 1H), 4.61 (s, 1H), 4.27 (d, *J* = 17.2 Hz, 1H), 4.15 (d, *J* = 16.4 Hz, 1H), 3.95(t, *J* = 17.2 Hz, 1H), 3.60–3.47 (m, 2H), 0.65 (t, *J* = 7.0 Hz, 3H). ^13^C NMR (100 MHz, DMSO-*d*_6_): δ 174.7, 167.2, 164.6, 150.8, 144.3, 138.3, 131.2, 130.6, 130.4, 128.9, 128.2, 128.1, 126.3, 124.8, 124.7, 123.6, 117.4, 115.9, 115.2, 109.2, 77.4, 72.4, 62.9, 59.2, 47.2, 41.3, 13.5 (One carbon atom is missing in the ^13^C NMR spectrum. The reason may be that the NMR signals of one carbon atom of benzene ring overlap with those of other carbon atoms of benzene ring); HRMS (ESI): *m*/*z* calcd for C_30_H_26_N_2_O_6_Na [M+Na]^+^ 533.1689, found 533.1686.

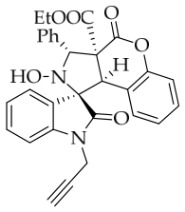

*(±)-Ethyl (1R,3R,3aS,9bR)-2-hydroxy-2′,4-dioxo-3-phenyl-1′-(prop-2-yn-1-yl)-2,3-dihydro-4H-spiro[chromeno[3,4-c]pyrrole-1,3′-indoline]-3a(9bH)-carboxylate* (**3n**). 148.9 mg, 59%, a white solid, >20:1 *dr*, mp: 176.3–168.4 °C; IR (thin film): *ν_max_* 3270, 3067, 3041, 2975, 2923, 1775, 1730, 1711, 1615, 1491, 1369, 1245, 1226, 1163, 759, 700, 612,529 cm^−1^; ^1^H NMR (600 MHz, CDCl_3_) δ 7.81 (ψd, *J* = 7.8 Hz, 3H), 7.47 (td, *J* = 7.8, 0.9 Hz, 1H), 7.39 (ψt, *J* = 7.8 Hz, 2H), 7.34 (t, *J* = 7.8 Hz, 1H), 7.31 (ψt, *J* = 7.2Hz, 1H), 7.16 (td, *J* = 7.1, 1.2 Hz, 1H), 7.01 (d, *J* = 8.4 Hz, 1H), 6.97 (d, *J* = 7.8 Hz, 1H), 6.75 (ψt, *J* = 7.5 Hz, 1H), 6.32 (d, *J* = 7.8 Hz, 1H), 6.05 (s, 1H), 4.74 (s, 1H), 4.72 (s, 1H), 4.31 (dd, *J* = 17.4, 2.4 Hz, 1H), 4.12 (dd, *J* = 17.4, 2.4 Hz, 1H), 3.64 (dq, *J* = 10.8, 7.2 Hz, 1H), 3.56 (dq, *J* = 10.8, 7.2 Hz, 1H), 1.92 (t, *J* = 2.4 Hz, 1H), 0.74 (t, *J* = 7.2 Hz, 3H); ^13^C NMR (100 MHz, CDCl_3_): δ 173.5, 166.8, 164.5, 150.9, 143.2, 137.2, 130.5, 129.6, 128.8, 128.2, 128.1, 127.6, 125.9, 124.1, 124.0, 123.7, 117.5, 114.5, 109.7, 77.2, 76.1, 72.6, 71.8, 63.0, 58.0, 47.2, 28.8, 13.2; HRMS (ESI): *m*/*z* calcd for C_30_H_24_N_2_O_6_Na [M+Na]^+^ 531.1532, found 531.1511.

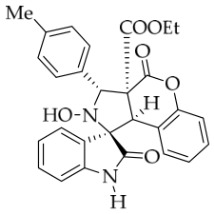

*(±)-Ethyl (1R,3R,3aS,9bR)-2-hydroxy-2′,4-dioxo-3-(p-tolyl)-2,3-dihydro-4H-spiro[chromeno[3,4-c]pyrrole-1,3′-indoline]-3a(9bH)-carboxylate* (**3p**). 160.2 mg, 66%, a white solid, >20:1 *dr*, mp: 162.2–164.0 °C; IR (thin film): *ν_max_* 3384, 3098, 3032, 2981, 2921, 2851, 1763, 1736, 1713, 1621, 1491, 1458, 1350, 1228, 1170, 754, 641, 583 cm^−1^; ^1^H NMR (400 MHz, DMSO-*d*_6_) δ 10.28 (s, 1H), 8.37 (s, 1H), 7.71 (d, *J* = 7.2 Hz, 1H), 7.56 (d, *J* = 8.0 Hz, 2H), 7.34 (t, *J* = 7.6 Hz, 1H), 7.27–7.17 (m, 4H), 7.06 (d, *J* = 8.4 Hz, 1H), 6.87 (ψt, *J* = 7.4 Hz, 1H), 6.74 (d, *J* = 7.6 Hz, 1H), 6.35 (d, *J* = 7.6 Hz, 1H), 5.65 (s, 1H), 4.53 (s, 1H), 3.58–3.51 (m, 2H), 2.30 (s, 3H), 0.65 (t, *J* = 7.2 Hz, 3H). ^13^C NMR (100 MHz, DMSO-*d*_6_): δ 176.9, 167.3, 164.6, 150.8, 143.9, 137.2, 135.3, 130.5, 130.2, 128.8, 128.7, 128.0, 127.3, 125.0, 124.6, 122.8, 117.3, 115.6, 110.3, 77.4, 72.0, 62.8, 59.1, 46.9, 21.2, 13.4; HRMS (ESI): *m*/*z* calcd for C_28_H_24_N_2_O_6_Na [M+Na]^+^ 507.1532, found 507.1521.

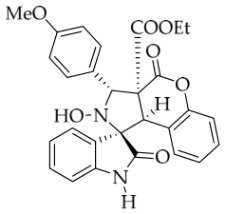

*(±)-Ethyl (1R,3R,3aS,9bR)-2-hydroxy-3-(4-methoxyphenyl)-2′,4-dioxo-2,3-dihydro-4H-spiro[chromeno[3,4-c]pyrrole-1,3′-indoline]-3a(9bH)-carboxylate* (**3q**). 135.5 mg, 54%, a white solid, >20:1 *dr*, mp: 150.5–151.3 °C; IR (thin film): *ν_max_* 3422, 3189, 3086, 2917, 2848, 1778, 1736, 1716, 1619, 1512, 1471, 1247, 1166, 1026, 751, 637, 558 cm^−1^; ^1^H NMR (400 MHz, DMSO-*d*_6_) δ 10.28 (s, 1H), 8.36 (s, 1H), 7.70 (d, *J* = 7.2 Hz, 1H), 7.58 (d, *J* = 8.4 Hz, 2H), 7.33 (ψt, *J* = 7.6 Hz, 1H), 7.27–7.19 (m, 2H), 7.06 (d, *J* = 8.0 Hz, 1H), 6.94 (d, *J* = 8.4 Hz, 2H), 6.87 (ψt, *J* = 7.4 Hz, 1H), 6.74 (d, *J* = 7.6 Hz, 1H), 6.34 (d, *J* = 7.6 Hz, 1H), 5.64 (s, 1H), 4.54 (s, 1H), 3.75 (s, 3H), 3.59–3.52 (m, 2H), 0.68 (t, *J* = 7.0 Hz, 3H). ^13^C NMR (100 MHz, DMSO-*d*_6_): δ 176.9, 167.3, 164.7, 159.4, 150.8, 143.8, 130.5, 130.2, 130.1, 130.0, 128.0, 127.3, 124.9, 124.6, 122.8, 117.3, 115.7, 113.6, 110.3, 77.4, 71.9, 62.9, 59.1, 55.6, 46.8, 13.5; HRMS (ESI): *m*/*z* calcd for C_28_H_24_N_2_O_7_Na [M+Na]^+^ 523.1481, found 523.10467.

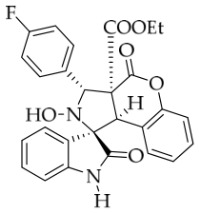

*(±)-Ethyl (1R,3R,3aS,9bR)-3-(4-fluorophenyl)-2-hydroxy-2′,4-dioxo-2,3-dihydro-4H-spiro[chromeno[3,4-c]pyrrole-1,3′-indoline]-3a(9bH)-carboxylate* (**3r**). 189.7 mg, 78%, a white solid, >20:1 *dr*, mp: 205.6–206.3 °C; IR (thin film): *ν_max_* 3350, 3096, 2980, 2840, 1760, 1737, 1711, 1621, 1509, 1472, 1458, 1226, 1177, 1158, 756, 643, 509 cm^−1^; ^1^H NMR (400 MHz, DMSO-*d*_6_) δ 10.31 (s, 1H), 8.47 (s, 1H), 7.72 (m, 3H), 7.34 (t, *J* = 7.4 Hz, 1H), 7.28–7.21 (m, 4H), 7.07 (d, *J* = 8.4 Hz, 1H), 6.88 (ψt, *J* = 7.2 Hz, 1H), 6.75 (d, *J* = 7.6 Hz, 1H), 6.35 (d, *J* = 8.0 Hz, 1H), 5.68 (s, 1H), 4.53 (s, 1H), 3.59 (q, *J* = 7.0 Hz, 2H), 0.69 (t, *J* = 7.0 Hz, 3H). ^13^C NMR (100 MHz, DMSO-*d*_6_): δ 176.8, 167.3, 164.7, 150.7, 143.9, 134.4 (d, *J* = 3.0 Hz), 130.9, 130.8 (d, *J* = 8.0 Hz), 130.6, 130.3, 128.0, 127.1, 125.0, 124.7, 122.9, 117.4, 115.5, 115.1, 114.9, 110.3, 77.4, 71.6, 63.0, 59.0, 46.9, 13.5; ^19^F NMR (376 MHz, DMSO-*d*_6_): -115.0; HRMS (ESI): *m*/*z* calcd for C_27_H_21_N_2_O_6_FNa [M+Na]^+^ 511.1281, found 511.1279.

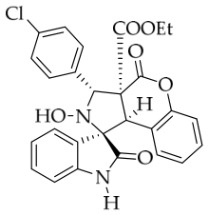

*(±)-Ethyl (1R,3R,3aS,9bR)-3-(4-chlorophenyl)-2-hydroxy-2′,4-dioxo-2,3-dihydro-4H-spiro[chromeno[3,4-c]pyrrole-1,3′-indoline]-3a(9bH)-carboxylate* (**3s**). 218.2 mg, 86%, a white solid, >20:1 *dr*, mp: 166.7–167.4 °C; IR (thin film): *ν_max_* 3368, 3088, 2983, 2836, 1766, 1736, 1709, 1621, 1490, 1457, 1227, 1177, 1017, 752, 670, 579 cm^−1^; ^1^H NMR (400 MHz, DMSO-*d*_6_) δ 10.32 (s, 1H), 8.51 (s, 1H), 7.73–7.68 (m, 3H), 7.47 (d, *J* = 8.0 Hz, 2H), 7.34 (ψt, *J* = 7.8 Hz, 1H), 7.26 (ψt, *J* = 7.6 Hz, 1H), 7.23 (ψt, *J* = 7.2 Hz, 1H), 7.08 (d, *J* = 8.0 Hz, 1H), 6.89 (ψt, *J* = 7.6 Hz, 1H), 6.75 (d, *J* = 8.0 Hz, 1H), 6.35 (d, *J* = 7.6 Hz, 1H), 5.67 (s, 1H), 4.52 (s, 1H), 3.61 (q, *J* = 7.0 Hz, 2H), 0.68 (t, *J* = 7.0 Hz, 3H). ^13^C NMR (100 MHz, DMSO-*d*_6_): δ 176.4, 167.3, 164.7, 150.7, 143.9, 137.3, 132.8, 130.7, 130.3, 128.2, 128.0, 127.0, 125.0, 124.7, 122.9, 117.4, 115.4, 110.3, 77.4, 71.5, 63.0, 59.0, 47.0, 13.5 (One carbon atom is missing in the ^13^C NMR spectrum. The reason may be that the NMR signals of one carbon atom of benzene ring overlap with those of other carbon atoms of benzene ring); HRMS (ESI): *m*/*z* calcd for C_27_H_21_N_2_O_6_ClNa [M+Na]^+^ 527.0986, found 527.0984.

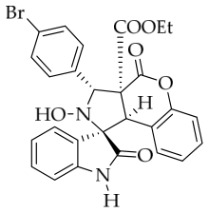

*(±)-Ethyl (1R,3R,3aS,9bR)-3-(4-bromophenyl)-2-hydroxy-2′,4-dioxo-2,3-dihydro-4H-spiro[chromeno[3,4-c]pyrrole-1,3′-indoline]-3a(9bH)-carboxylate* (**3t**). 225.9 mg, 82%, a white solid, >20:1 *dr*, mp: 161.6–163.0 °C; IR (thin film): *ν_max_* 3513, 3282, 2859, 1784, 1714, 1688, 1487, 1247, 1226, 1178, 1010, 858, 748, 665, 570 cm^−1^; ^1^H NMR (400 MHz, DMSO-*d*_6_) δ 10.32 (s, 1H), 8.51 (s, 1H), 7.72 (d, *J* = 7.2 Hz, 1H), 7.63 (d, *J* = 8.4 Hz, 2H), 7.60 (d, *J* = 8.4 Hz, 2H), 7.34 (ψt, *J* = 7.6 Hz, 1H), 7.26 (ψt, *J* = 7.6 Hz, 1H), 7.22 (ψt, *J* = 7.6 Hz, 1H),7.08 (d, *J* = 8.0 Hz, 1H), 6.88 (ψt, *J* = 7.4 Hz, 1H), 6.75 (d, *J* = 7.2 Hz, 1H), 6.35 (d, *J* = 7.6 Hz, 1H), 5.66 (s, 1H), 4.52 (s, 1H), 3.61 (q, *J* = 7.1 Hz, 2H), 0.68 (t, *J* = 7.0 Hz, 3H). ^13^C NMR (100 MHz, DMSO-*d*_6_): δ 176.7, 167.3, 164.7, 150.7, 143.9, 137.7, 131.2, 131.0, 130.6, 130.3, 128.0, 126.9, 125.0, 124.7, 122.9, 121.3, 117.4, 115.4, 110.3, 77.4, 71.6, 63.0, 59.0, 47.0, 13.5; HRMS (ESI): *m*/*z* calcd for C_27_H_21_N_2_O_6_BrNa [M+Na]^+^ 571.0481, found 571.0471.

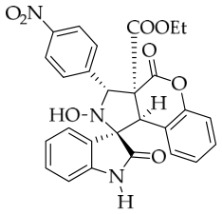

*(±)-Ethyl (1R,3R,3aS,9bR)-2-hydroxy-3-(4-nitrophenyl)-2′,4-dioxo-2,3-dihydro-4H-spiro[chromeno[3,4-c]pyrrole-1,3′-indoline]-3a(9bH)-carboxylate* (**3u**). 122.1 mg, 47%, a white solid, >20:1 *dr*, mp: 170.4–171.8 °C; IR (thin film): *ν_max_* 3360, 3288, 2941, 2345, 1782, 1735, 1605, 1523, 1492, 1347, 1256, 1174, 1112, 1017, 856, 745, 608, 573 cm^−1^; ^1^H NMR (400 MHz, DMSO-*d*_6_) δ 10.37 (s, 1H), 8.69 (s, 1H), 8.30 (d, *J* = 8.4 Hz, 2H), 7.97 (d, *J* = 8.4 Hz, 2H), 7.75 (d, *J* = 7.2 Hz, 1H), 7.36 (ψt, *J* = 7.5 Hz, 1H), 7.28 (ψt, *J* = 7.6 Hz, 1H), 7.25 (ψt, *J* = 7.2 Hz, 1H), 7.11 (d, *J* = 8.4 Hz, 1H), 6.90 (ψt, *J* = 7.4 Hz, 1H), 6.77 (d, *J* = 7.6 Hz, 1H), 6.37 (d, *J* = 7.2 Hz, 1H), 5.80 (s, 1H), 4.53 (s, 1H), 3.60 (q, *J* = 7.0 Hz, 2H), 0.66 (t, *J* = 7.0 Hz, 3H). ^13^C NMR (100 MHz, DMSO-*d*_6_): δ 176.6, 167.3, 164.7, 150.6, 147.6, 146.1, 143.9, 130.8, 130.4, 130.0, 128.1, 126.6, 125.2, 124.8, 123.5, 123.0, 117.4, 115.2, 110.4, 77.6, 71.5, 63.2, 59.1, 47.3, 13.5; HRMS (ESI): *m*/*z* calcd for C_27_H_21_N_3_O_8_Na [M+Na]^+^ 538.1226, found 538.1215.

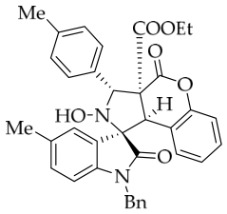

*(±)-Ethyl (1R,3R,3aS,9bR)-1′-benzyl-2-hydroxy-5′-methyl-2′,4-dioxo-3-(p-tolyl)-2,3-dihydro-4H-spiro[chromeno[3,4-c]pyrrole-1,3′-indoline]-3a(9bH)-carboxylate* (**3w**). 227.7 mg, 77%, a white solid, >20:1 *dr*, mp: 167.4–169.2 °C; IR (thin film): *ν_max_* 3369, 3037, 2975, 2915, 2851, 1757, 1741, 1709, 1609, 1498, 1369, 1271, 1248, 1178, 801, 769, 623, 565 cm^−1^;^1^H NMR (400 MHz, DMSO-*d*_6_) δ 8.47 (s, 1H), 7.63 (s, 1H), 7.58 (d, *J* = 8.0 Hz, 2H), 7.39 (t, *J* = 7.2 Hz, 1H), 7.19 (d, *J* = 8.0 Hz, 2H), 7.12 (d, *J* = 8.8 Hz, 1H), 7.11 (d, *J* = 6.8 Hz, 2H), 7.04 (ψt, *J* = 7.2 Hz, 2H), 6.91 (ψt, *J* = 7.2 Hz, 1H), 6.56 (d, *J* = 8.0 Hz, 1H), 6.50 (d, *J* = 7.2 Hz, 2H), 6.42 (d, *J* = 7.2 Hz, 1H), 5.70 (s, 1H), 4.85 (d, *J* = 16.4 Hz, 1H), 4.64 (s, 1H), 4.46 (d, *J* = 16.4 Hz, 1H), 3.64–3.51 (m, 2H), 2.42 (s, 3H), 2.31 (s, 3H), 0.66 (t, *J* = 7.0 Hz, 3H); ^13^C NMR (100 MHz, DMSO-*d*_6_): δ 175.0, 167.2, 164.7, 150.8, 141.8, 137.2, 135.8, 135.2, 132.8, 130.8, 130.5, 128.8, 128.7, 128.5, 127.4, 126.7, 126.5, 125.5, 124.9, 117.4, 115.3, 109.7, 77.4, 72.2, 62.9, 59.3, 46.9, 42.6, 21.3, 13.5; HRMS (ESI): *m*/*z* calcd for C_36_H_32_N_2_O_6_Na [M+Na]^+^ 611.2158, found.611.2137.

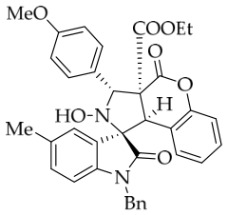

*(±)-Ethyl (1R,3R,3aS,9bR)-1′-benzyl-2-hydroxy-3-(4-methoxyphenyl)-5′-methyl-2′,4-dioxo-2,3-dihydro-4H-spiro[chromeno[3,4-c]pyrrole-1,3′-indoline]-3a(9bH)-carboxylate* (**3x**). 149.9 mg, 50%, a white solid, >20:1 *dr*, mp: 147.1–147.7 °C; IR (thin film): *ν*_max_ 3440, 3069, 3032, 2983, 2935, 2836, 1776, 1736, 1694, 1612, 1514, 1498, 1370, 1244, 1163, 1134, 1022, 823, 754, 699, 567 cm^−1^; ^1^H NMR (400 MHz, DMSO-*d*_6_) δ 8.46 (s, 1H), 7.63 (s, 1H), 7.60 (d, *J* = 8.8 Hz, 2H), 7.39 (t, *J* = 7.6 Hz, 1H), 7.11 (ψd, *J* = 7.2 Hz, 3H), 7.04 (ψt, *J* = 7.4 Hz, 2H), 6.95 (d, *J* = 8.8 Hz, 2H), 6.90 (ψt, *J* = 7.6 Hz, 1H), 6.56 (d, *J* = 8.0 Hz, 1H), 6.50 (d, *J* = 7.2 Hz, 2H), 6.42 (d, *J* = 7.6 Hz, 1H), 5.69 (s, 1H), 4.85 (d, *J* = 16.2 Hz, 1H), 4.65 (s, 1H), 4.47 (d, *J* = 16.2 Hz, 1H), 3.76 (s, 3H), 3.66–3.53 (m, 2H), 2.42 (s, 3H), 0.69 (t, *J* = 7.0 Hz, 3H). ^13^C NMR (100 MHz, DMSO-*d*_6_): δ 175.0, 167.2, 164.7, 159.4, 150.8, 141.8, 135.8, 132.8, 130.8, 130.5, 130.1, 128.8, 128.5, 127.4, 126.7, 126.5, 125.5, 124.9, 117.4, 115.3, 113.6, 109.7, 77.4, 72.1, 62.9, 59.2, 55.6, 46.8, 42.6, 21.3, 13.5 (One carbon atom is missing in the ^13^C NMR spectrum. The reason may be that the NMR signals of one carbon atom of benzene ring overlap with those of other carbon atoms of benzene ring); HRMS (ESI): *m*/*z* calcd for C_36_H_32_N_2_O_7_Na [M+Na]^+^ 627.2107, found.627.2096.

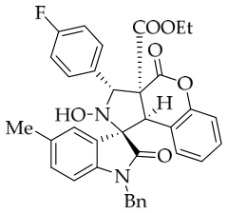

*(±)-Ethyl (1R,3R,3aS,9bR)-1′-benzyl-3-(4-fluorophenyl)-2-hydroxy-5′-methyl-2′,4-dioxo-2,3-dihydro-4H-spiro[chromeno[3,4-c]pyrrole-1,3′-indoline]-3a(9bH)-carboxylate* (**3y**). 205.6 mg, 69%, a white solid, >20:1 *dr*, mp: 185.6–186.5 °C; IR (thin film): *ν*_max_ 3478, 2989, 2960, 2918, 1774, 1737, 1710, 1613, 1516, 1496, 1158, 812, 765, 610, 503 cm^−1^; ^1^H NMR (400 MHz, DMSO-*d*_6_) δ 8.57 (s, 1H), 7.74 (d, *J* = 7.2 Hz, 1H), 7.74 (d, *J* = 6.4 Hz, 1H), 7.65 (s, 1H), 7.40 (t, *J* = 7.6 Hz, 1H), 7.25 (ψt, *J* = 8.6 Hz, 2H), 7.12 (ψt, *J* = 7.2 Hz, 3H), 7.04 (ψt, *J* = 7.4 Hz, 2H), 6.92 (ψt, *J* = 7.6 Hz, 1H), 6.57 (d, *J* = 8.0 Hz, 1H), 6.50 (d, *J* = 7.6 Hz, 2H), 6.42 (d, *J* = 7.6 Hz, 1H), 5.73 (s, 1H), 4.85 (d, *J* = 16.4 Hz, 1H), 4.64 (s, 1H), 4.47 (d, *J* = 16.4 Hz, 1H), 3.68–3.55 (m, 2H), 2.42 (s, 3H), 0.70 (t, *J* = 7.2 Hz, 3H). ^13^C NMR (100 MHz, DMSO-*d*_6_): δ 174.9, 167.3, 164.7, 150.8, 141.8, 135.8, 134.3 (d, *J* = 2.8 Hz), 132.8, 130.9, 130.8, 130.6, 128.5, 127.4, 126.7, 126.3, 125.5, 124.9, 117.5, 115.2, 114.9, 109.7, 77.4, 71.8, 63.1, 59.2, 46.9, 42.7, 21.3, 13.5 (There are two extra peaks in the ^13^C NMR spectrum. The reason may be that the splitting is caused by the coupling of the fluorine atom on the benzene ring to the attached and adjacent carbon atoms); ^19^F NMR (376 MHz, DMSO-*d*_6_): -114.9; HRMS (ESI): *m*/*z* calcd for C_35_H_29_N_2_O_6_FNa [M+Na]^+^ 615.1907, found 615.1904.

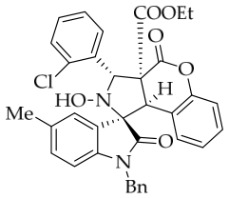

*(±)-Ethyl (1R,3S,3aS,9bR)-1′-benzyl-3-(2-chlorophenyl)-2-hydroxy-5′-methyl-2′,4-dioxo-2,3-dihydro-4H-spiro[chromeno[3,4-c]pyrrole-1,3′-indoline]-3a(9bH)-carboxylate* (**3z**). 147.3 mg, 48%, a white solid, >20:1 *dr*, mp: 155.0–156.2 °C; IR (thin film): *ν*_max_ 3447, 3069, 3030, 2979, 2921, 2867, 1782, 1717, 1605, 1497, 1457, 1370, 1259, 1226, 1152, 1007, 815, 756, 695, 532 cm^−1^; ^1^H NMR (400 MHz, DMSO-*d*_6_) δ 8.44 (s, 1H), 7.89 (d, *J* = 7.2 Hz, 1H), 7.79 (s, 1H), 7.47 (d, *J* = 7.6 Hz, 1H), 7.43–7.33 (m, 3H), 7.14–7.12 (m, 5H), 6.92 (ψt, *J* = 7.2 Hz, 1H), 6.52 (d, *J* = 8.0 Hz, 1H), 6.47 (ψd, *J* = 6.4 Hz, 3H), 6.20 (s, 1H), 4.87 (d, *J* = 16.2 Hz, 1H), 4.80 (s, 1H), 4.46 (d, *J* = 16.2 Hz, 1H), 3.69–3.61 (m, 1H), 3.48–3.40 (m, 1H), 2.41 (s, 1H), 0.63 (t, *J* = 7.0 Hz, 3H). ^13^C NMR (100 MHz, DMSO-*d*_6_): δ 174.9, 166.1, 162.4, 151.0, 141.7, 137.3, 135.8, 135.1, 132.8, 131.7, 130.8, 130.5, 130.0, 129.5, 128.9, 128.4, 127.4, 127.1, 126.6, 126.5, 125.8, 124.7, 117.3, 115.5, 109.6, 77.4, 69.5, 63.1, 60.1, 46.9, 42.6, 21.1, 13.4; HRMS (ESI): *m*/*z* calcd for C_35_H_29_N_2_O_6_ClNa [M+Na]^+^ 631.1612, found 631.1602.

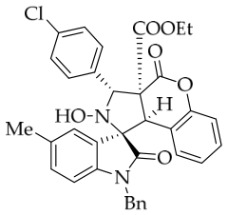

*(±)-Ethyl (1R,3S,3aS,9bR)-1′-benzyl-3-(4-chlorophenyl)-2-hydroxy-5′-methyl-2′,4-dioxo-2,3-dihydro-4H-spiro[chromeno[3,4-c]pyrrole-1,3′-indoline]-3a(9bH)-carboxylate* (**3aa**). 276.5 mg, 85%, a white solid, >20:1 *dr*, mp: 192.4–193.7 °C; IR (thin film): *ν*_max_ 3454, 3067, 3018, 2983, 2925, 2859, 1774, 1729, 1705, 1607, 1497, 1455, 1374, 1349, 1262, 1225, 1165, 1015, 811, 755, 629, 544 cm^−1^; ^1^H NMR (400 MHz, DMSO-*d*_6_) δ 8.60 (s, 1H), 7.72 (d, *J* = 8.0 Hz, 2H), 7.65 (s, 1H), 7.48 (d, *J* = 8.0 Hz, 2H), 7.40 (t, *J* = 7.6 Hz, 1H), 7.13 (ψt, *J* = 7.8 Hz, 3H), 7.04 (ψt, *J* = 7.8 Hz, 2H), 6.92 (ψt, *J* = 7.4 Hz, 1H), 6.57 (d, *J* = 8.0 Hz, 1H), 6.50 (d, *J* = 7.2 Hz, 2H), 6.42 (d, *J* = 7.6 Hz, 1H), 5.72 (s, 1H), 4.85 (d, *J* = 16.0 Hz, 1H), 4.63 (s, 1H), 4.47 (d, *J* = 16.0 Hz, 1H), 3.70–3.58 (m, 2H), 2.42 (s, 3H), 0.70 (t, *J* = 7.0 Hz, 3H). ^13^C NMR (100 MHz, DMSO-*d*_6_): δ 174.9, 167.2, 164.7, 150.8, 141.8, 137.2, 135.7, 132.8, 130.9, 130.7, 128.8, 128.5, 128.3, 127.4, 126.7, 126.2, 125.6, 125.0, 117.5, 115.1, 109.7, 77.4, 71.4, 63.1, 59.1, 47.0, 42.7, 21.2, 13.5 (Two carbon atoms are missing in the ^13^C NMR spectrum. The reason may be that the NMR signals of two carbon atoms of benzene ring overlap with those of other carbon atoms of benzene ring); HRMS (ESI): *m*/*z* calcd for C_35_H_29_N_2_O_6_ClNa [M+Na]^+^ 631.1612, found 631.1613.

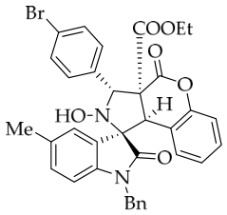

*(±)-Ethyl (1R,3S,3aS,9bR)-1′-benzyl-3-(4-bromophenyl)-2-hydroxy-5′-methyl-2′,4-dioxo-2,3-dihydro-4H-spiro[chromeno[3,4-c]pyrrole-1,3′-indoline]-3a(9bH)-carboxylate* (**3ab**). 204.7 mg, 66%, a white solid, >20:1 *dr*, mp: 193.1–194.8 °C; IR (thin film): *ν*_max_ 3370, 3032, 2977, 2929, 2902, 1753, 1741, 1708, 1497, 1369, 1250, 1225, 1182, 1013, 770, 806, 770, 612, 536 cm^−1^; ^1^H NMR (400 MHz, DMSO-*d*_6_) δ 8.60 (s, 1H), 7.67–7.61 (m, 5H), 7.40 (t, *J* = 7.2 Hz, 1H), 7.12 (ψt, *J* = 7.4 Hz, 3H), 7.04 (ψt, *J* = 7.4 Hz, 2H), 6.92 (ψt, *J* = 7.6 Hz, 1H), 6.57 (d, *J* = 8.0 Hz, 1H), 6.51 (d, *J* = 7.6 Hz, 2H), 6.42 (d, *J* = 7.6 Hz, 1H), 5.71 (s, 1H), 4.84 (d, *J* = 16.0 Hz, 1H), 4.63 (s, 1H), 4.47 (d, *J* = 16.0 Hz, 1H), 3.70–3.59 (m, 2H), 2.42 (s, 3H), 0.70 (t, *J* = 7.2 Hz, 3H). ^13^C NMR (100 MHz, DMSO-*d*_6_): δ 174.9, 167.2, 164.7, 150.7, 141.8, 137.6, 135.7, 132.8, 131.2, 131.0, 130.9, 130.6, 128.8, 128.5, 127.4, 126.7, 126.2, 125.6, 125.0, 121.4, 117.5, 115.1, 109.7, 77.4, 71.8, 63.1, 59.1, 47.0, 42.6, 21.2, 13.5; HRMS (ESI): *m*/*z* calcd for C_35_H_29_N_2_O_6_BrNa [M+Na]^+^ 675.1107, found 675.1080.

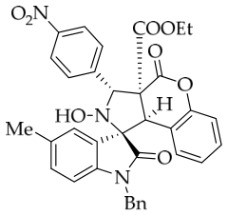

*(±)-Ethyl (1R,3R,3aS,9bR)-1′-benzyl-2-hydroxy-5′-methyl-3-(4-nitrophenyl)-2′,4-dioxo-2,3-dihydro-4H-spiro[chromeno[3,4-c]pyrrole-1,3′-indoline]-3a(9bH)-carboxylate* (**3ac**). 157.8 mg, 52%, a white solid, >20:1 *dr*, mp: 194.1–195.5 °C; IR (thin film): *v*_max_ 3500, 3063, 3037, 2989, 2921, 1782, 1707, 1605, 1516, 1495, 1456, 1347, 1261, 1247, 1231, 1177, 765, 697, 544 cm^−1^; ^1^H NMR (400 MHz, DMSO-*d*_6_) δ 8.78 (s, 1H), 8.31 (d, *J* = 7.6 Hz, 2H), 7.99 (d, *J* = 8.4 Hz, 2H), 7.68 (s, 1H), 7.42 (t, *J* = 7.6 Hz, 1H), 7.16 (d, *J* = 8.8 Hz, 1H), 7.12 (ψt, *J* = 7.2 Hz, 2H), 7.05 (ψt, *J* = 7.4 Hz, 2H), 6.94 (ψt, *J* = 7.2 Hz, 1H), 6.59 (d, *J* = 8.0 Hz, 1H), 6.51 (d, *J* = 7.2 Hz, 2H), 6.44 (d, *J* = 7.6 Hz, 1H), 5.84 (s, 1H), 4.86 (d, *J* = 16.2 Hz, 1H), 4.64 (s, 1H), 4.49 (d, *J* = 16.2 Hz, 1H), 3.67–3.59 (m, 2H), 2.43 (s, 3H), 0.67 (t, *J* = 7.0 Hz, 3H). ^13^C NMR (100 MHz, DMSO-*d*_6_): δ 174.8, 167.2, 164.7, 150.7, 147.6, 146.0, 141.8, 135.7, 132.9, 131.0, 130.7, 130.0, 128.9, 128.5, 127.4, 126.7, 125.9, 125.7, 125.1, 123.5, 117.6, 114.8, 109.8, 77.5, 71.7, 69.3, 59.2, 47.3, 42.7, 21.3, 13.5; HRMS (ESI): *m*/*z* calcd for C_35_H_29_N_3_O_8_Na [M+Na]^+^ 642.1852, found 642.1846.

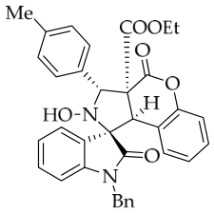

*(±)-Ethyl (1R,3R,3aS,9bR)-1′-benzyl-2-hydroxy-2′,4-dioxo-3-(p-tolyl)-2,3-dihydro-4H-spiro[chromeno[3,4-c]pyrrole-1,3′-indoline]-3a(9bH)-carboxylate* (**3ad**).133.6 mg, 46%, a white solid, >20:1 *dr,* mp: 164.5–166.5 °C; IR (thin film): *ν*_max_ 3852, 3678, 3649, 1712, 1460, 1369, 1231, 1176, 857, 738, 620, 554 cm^−1^; ^1^H NMR (400 MHz, DMSO-*d*_6_) δ 8.48 (s, 1H), 7.81 (d, *J* = 6.8 Hz, 1H), 7.58 (d, *J* = 7.6 Hz, 2H), 7.39 (t, *J* = 7.4 Hz, 1H), 7.32 (ψt, *J* = 7.2 Hz, 1H), 7.28 (ψt, *J* = 7.6 Hz, 1H), 7.20 (d, *J* = 8.0 Hz, 2H), 7.12 (d, *J* = 8.0 Hz, 2H), 7.05 (ψt, *J* = 7.4 Hz, 2H), 6.89 (ψt, *J* = 7.6 Hz, 1H), 6.68 (d, *J* = 7.6 Hz, 1H), 6.52 (d, *J* = 7.6 Hz, 2H), 6.37 (d, *J* = 7.6 Hz, 1H), 5.71 (s, 1H), 4.87 (d, *J* = 16.2 Hz, 1H), 4.66 (s, 1H), 4.50 (d, *J* = 16.2 Hz, 1H), 3.61–3.52 (m, 2H), 2.31 (s, 3H), 0.67 (t, *J* = 7.2 Hz, 3H); ^13^C NMR (100 MHz, DMSO-*d*_6_): δ 175.1, 167.2, 164.7, 150.8, 144.1, 137.3, 135.7, 135.2, 130.6, 128.8, 128.7, 128.4, 127.4, 126.7, 126.5, 125.0, 124.9, 123.7, 117.5, 115.2, 109.9, 77.3, 72.4, 62.9, 59.3, 47.0, 42.6, 21.2, 13.5 (Two carbon atoms are missing in the ^13^C NMR spectrum. The reason may be that the NMR signals of two carbon atoms of benzene ring overlap with those of other carbon atoms of benzene ring); HRMS (ESI): *m*/*z* calcd for C_35_H_30_N_2_O_6_Na [M+Na]^+^ 597.2002, found 597.2014.

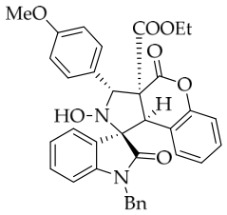

*(±)-Ethyl (1R,3R,3aS,9bR)-1′-benzyl-2-hydroxy-3-(4-methoxyphenyl)-2′,4-dioxo-2,3-dihydro-4H-spiro[chromeno[3,4-c]pyrrole-1,3′-indoline]-3a(9bH)-carboxylate* (**3ae**). 112.0 mg, 38%, a yellow solid, >20:1 *dr,* mp: 169.3–169.6 °C; IR (thin film): *ν*_max_ 3853, 3669, 3629, 3565, 1779, 1703, 1611, 1462, 1234, 1171, 1019, 855, 757, 668, 602, 516 cm^−1^; ^1^H NMR (400 MHz, DMSO-*d*_6_) δ 8.48 (s, 1H), 7.80 (d, *J* = 7.2 Hz, 1H), 7.61 (d, *J* = 8.4 Hz, 2H), 7.39 (t, *J* = 7.8 Hz, 1H), 7.32 (ψt, *J* = 7.8 Hz, 1H), 7.28 (ψt, *J* = 7.4 Hz, 1H), 7.12 (d, *J* = 8.0 Hz, 2H), 7.05 (ψt, *J* = 7.6 Hz, 2H), 6.96 (d, *J* = 8.4 Hz, 2H), 6.88 (ψt, *J* = 7.6 Hz, 1H), 6.68 (d, *J* = 7.2 Hz, 1H), 6.52 (d, *J* = 7.6 Hz, 2H), 6.37 (d, *J* = 7.6 Hz, 1H), 5.70 (s, 1H), 4.87 (d, *J* = 16.4 Hz, 1H), 4.67 (s, 1H), 4.50 (d, *J* = 16.4 Hz, 1H), 3.76 (s, 3H), 3.65–3.54 (m, 2H), 0.70 (t, *J* = 7.0 Hz, 3H); ^13^C NMR (100 MHz, DMSO-*d*_6_): δ 175.1, 167.2, 164.7, 159.4, 150.8, 144.1, 135.7, 130.6, 130.1, 130.0, 128.9, 128.4, 127.4, 126.7, 126.5, 124.9, 124.9, 123.7, 117.5, 115.3, 113.6, 109.9, 77.3, 72.1, 62.9, 59.3, 55.6, 46.8, 42.6, 13.5 (One carbon atom is missing in the ^13^C NMR spectrum. The reason may be that the NMR signals of one carbon atom of benzene ring overlap with those of other carbon atoms of benzene ring.); HRMS (ESI): *m*/*z* calcd for C_35_H_30_N_2_O_7_Na [M+Na]^+^ 613.1951, found 613.1949.

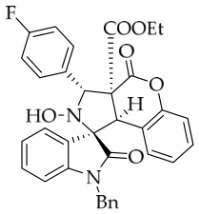

*(±)-Ethyl (1R,3R,3aS,9bR)-1′-benzyl-3-(4-fluorophenyl)-2-hydroxy-2′,4-dioxo-2,3-dihydro-4H-spiro[chromeno[3,4-c]pyrrole-1,3′-indoline]-3a(9bH)-carboxylate* (**3af**). 181.3 mg, 63%, a yellow solid, >20:1 *dr*, mp: 161.1–163.6 °C; IR (thin film): *ν_max_* 3852,3696, 3680, 3624, 3564, 3422, 1773, 1732, 1611, 1369, 1169, 858, 819, 757, 507 cm^−1; 1^H NMR (400 MHz, DMSO-*d*_6_) δ 8.59 (s, 1H), 7.82 (d, *J* = 8.0 Hz, 1H), 7.74 (dd, *J* = 8.4, 6.0 Hz, 2H), 7.40 (td, *J* = 8.4, 1.0 Hz, 1H), 7.32 (td, *J* = 7.6, 1.2 Hz, 1H), 7.32 (ψt, *J* = 8.0 Hz, 1H), 7.32 (ψt, *J* = 8.8 Hz, 2H), 7.13 (ψt, *J* = 7.4 Hz, 2H), 7.05 (ψt, *J* = 7.4 Hz, 2H), 6.90 (ψt, *J* = 7.4 Hz, 1H), 6.69 (d, *J* = 7.6 Hz, 1H), 6.53 (d, *J* = 7.6 Hz, 2H), 6.38 (d, *J* = 7.6 Hz, 1H), 5.75 (s, 1H), 4.87 (d, *J* = 16.0 Hz, 1H), 4.66 (s, 1H), 4.51 (d, *J* = 16.0 Hz, 1H), 3.65–3.58 (m, 2 H), 0.71 (t, *J* = 7.0 Hz, 3H); ^13^C NMR (100 MHz, DMSO-*d*_6_): δ 175.0, 167.2, 164.7, 150.8, 144.2, 135.7, 134.3 (d, *J* = 2.7 Hz), 130.9, 130.8, 130.7, 128.9, 128.4, 127.5, 126.7, 126.3, 125.0 (2C), 123.7, 117.5, 115.2, 115.1, 115.0, 109,9, 77.3, 71.8, 63.1, 59.1, 47.0, 42.6, 13.5; ^19^F NMR (376 MHz, DMSO-*d*_6_): -114.9; HRMS (ESI): *m*/*z* calcd for C_34_H_27_N_2_O_6_FNa [M+Na]^+^ 601.1751, found 601.1740.

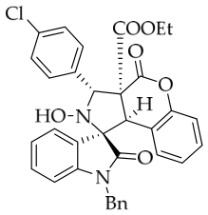

*(±)-Ethyl (1R,3R,3aS,9bR)-1′-benzyl-3-(4-chlorophenyl)-2-hydroxy-2′,4-dioxo-2,3-dihydro-4H-spiro[chromeno[3,4-c]pyrrole-1,3′-indoline]-3a(9bH)-carboxylate* (**3ag**). 225.5mg, 76%, a white solid, >20:1 *dr*, mp: 183.1–184.2 °C; IR (thin film): *ν*_max_ 3852, 3734, 3678, 3574, 3563, 2981, 2933, 1778, 1714, 1611, 1493, 1229,1165, 1009, 862, 754, 610, 544 cm^−1^; ^1^H NMR (400 MHz, DMSO-*d*_6_) δ 8.62 (s, 1H), 7.82 (d, *J* = 7.2 Hz, 1H), 7.72 (d, *J* = 8.4 Hz, 2H), 7.48 (d, *J* = 8.4 Hz, 2H), 7.40 (t, *J* = 8.0 Hz, 1H), 7.32 (ψt, *J* = 7.8 Hz, 1H), 7.28 (ψt, *J* = 7.2 Hz, 1H), 7.13 (ψt, *J* = 8.2 Hz, 1H), 7.05 (ψt, *J* = 7.6 Hz, 2H), 6.90 (ψt, *J* = 7.6 Hz, 1H), 6.69 (d, *J* = 7.2 Hz, 1H), 6.53 (d, *J* = 7.6 Hz, 2H), 6.38 (d, *J* = 7.6 Hz, 1H), 5.74 (s, 1H), 4.87 (d, *J* = 16.0 Hz, 1H), 4.65 (s, 1H), 4.51 (d, *J* = 16.0 Hz, 1H), 3.63 (q, *J* = 7.0 Hz, 2H), 0.71 (t, *J* = 7.0 Hz, 3H). ^13^C NMR (100 MHz, DMSO-*d*_6_): δ 175.0, 167.2, 164.7, 150.8, 144.1, 137.2, 135.7, 132.9, 130.7, 128.9, 128.4, 128.3, 127.5, 126.7, 126.2, 125.0 (2C), 123.7, 117.5, 115.0, 110.0, 77.3, 71.8, 63.1, 59.1, 47.0, 42.6, 13.5 (Two carbon atoms are missing in the ^13^C NMR spectrum. The reason may be that the NMR signals of two carbon atoms of the benzene ring overlap with those of other carbon atoms of the benzene ring.); HRMS (ESI): *m*/*z* calcd for C_34_H_27_N_2_O_6_ClNa [M+Na]^+^ 617.1455, found 617.1455.

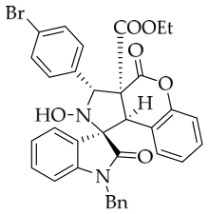

*(±)-Ethyl (1R,3R,3aS,9bR)-1′-benzyl-3-(4-bromophenyl)-2-hydroxy-2′,4-dioxo-2,3-dihydro-4H-spiro[chromeno[3,4-c]pyrrole-1,3′-indoline]-3a(9bH)-carboxylate* (**3ah**). 218.2 mg, 68%, a white solid; >20:1 *dr*, mp: 190.0–191.3 °C; IR (thin film): *ν*_max_ 3854, 3729, 3668, 3633, 1740, 1712, 1617, 1498, 1376, 1232, 1180, 1004, 858, 804, 736, 620 cm^−1^; ^1^H NMR (400 MHz, DMSO-*d*_6_) δ 8.62 (s, 1H), 7.82 (d, *J* = 7.6 Hz, 1H), 7.66 (d, *J* = 8.8 Hz, 2H), 7.62 (d, *J* = 8.8 Hz, 2H), 7.40 (t, *J* = 7.8 Hz, 1H), 7.32 (ψt, *J* = 7.6 Hz, 1H), 7.28 (ψt, *J* = 7.6 Hz, 1H), 7.13 (ψt, *J* = 8.4 Hz, 2H), 7.05 (t, *J* = 7.4 Hz, 2H), 6.90 (ψt, *J* = 7.0 Hz, 1H), 6.69 (d, *J* = 7.6 Hz, 1H), 6.52 (d, *J* = 6.8 Hz, 2H), 6.38 (d, *J* = 7.6 Hz, 1H), 5.72 (s, 1H), 4.87 (d, *J* = 16.2 Hz, 1H), 4.65 (s, 1H), 4.51 (d, *J* = 16.2 Hz, 1H), 3.64 (q, *J* = 7.0 Hz, 2H), 0.70 (t, *J* = 7.1 Hz, 3H); ^13^C NMR (100 MHz, DMSO-*d*_6_): δ 175.0, 167.2, 164.7, 150.8, 144.1, 137.6, 135.7, 131.2, 131.0, 130.7, 128.9, 128.4, 127.5, 126.7, 126.2, 125.0, 124.9, 123.7, 121.4, 117.5, 115.0, 109.9, 77.3, 71.8, 63.1, 59.1, 47.1, 42.7, 13.5 (One carbon atom is missing in the ^13^C NMR spectrum. The reason may be that the NMR signals of one carbon atom of the benzene ring overlap with those of other carbon atoms of the benzene ring.); HRMS (ESI): *m*/*z* calcd for C_34_H_27_N_2_O_6_BrNa [M+Na]^+^ 661.0950, found 661.0924.

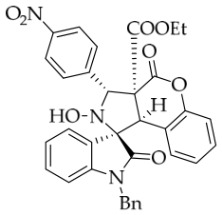

*(±)-Ethyl (1R,3R,3aS,9bR)-1′-benzyl-2-hydroxy-3-(4-nitrophenyl)-2′,4-dioxo-2,3-dihydro-4H-spiro[chromeno[3,4-c]pyrrole-1,3′-indoline]-3a(9bH)-carboxylate* (**3ai**). 182.1 mg, 60%, a white solid, >20:1 *dr,* mp: 202.1–203.2 °C; IR (thin film): *ν*_max_ 3854, 3736, 3639, 3612, 3396, 3070, 2987, 2931, 1728, 1610, 1348, 1235, 1178, 1009, 754, 699, 610, 551 cm^−1^; ^1^H NMR (400 MHz, DMSO-*d*_6_) δ 8.79 (s, 1H), 8.31 (d, *J* = 8.8 Hz, 2H), 8.00 (d, *J* = 8.8 Hz, 2H), 7.85 (d, *J* = 7.2 Hz, 1H), 7.42 (t, *J* = 8.0 Hz, 1H), 7.34 (t, *J* = 7.6 Hz, 1H), 7.30 (t, *J* = 7.6 Hz, 1H), 7.17 (d, *J* = 8.0 Hz, 1H), 7.12 (d, *J* = 6.8 Hz, 1H), 7.06 (ψt, *J* = 7.4 Hz, 2H), 6.92 (ψt, *J* = 7.4 Hz, 1H), 6.71 (d, *J* = 7.6 Hz, 1H), 6.53 (d, *J* = 7.6 Hz, 2H), 6.40 (d, *J* = 7.6 Hz, 1H), 5.86 (s, 1H), 4.88 (d, *J* = 16.2 Hz, 1H), 4.66 (s, 1H), 4.53 (d, *J* = 16.2 Hz, 1H), 3.62 (q, *J* = 7.0 Hz, 2H), 0.69 (t, *J* = 7.0 Hz, 3H); ^13^C NMR (100 MHz, DMSO-*d*_6_): δ 175.0, 167.2, 164.7, 150.7, 147.6, 146.0, 144.2, 135.6, 130.8, 130.0, 128.9, 128.4 127.5, 126.7, 125.9, 125.1, 125.0, 123.8, 123.5, 117.6, 114.8, 110.0, 77.5, 71.8, 63.3, 59.2, 47.3, 42.7, 13.5 (One carbon atom is missing in the ^13^C NMR spectrum. The reason may be that the NMR signals of one carbon atom of the benzene ring overlap with those of other carbon atoms of thte benzene ring.); HRMS (ESI): *m*/*z* calcd for C_34_H_27_N_3_O_8_Na [M+Na]^+^ 628.1696, found 628.1690.

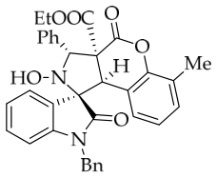

*(±)-Ethyl (1R,3R,3aS,9bR)-1′-benzyl-2-hydroxy-6-methyl-2′,4-dioxo-3-phenyl-2,3-dihydro-4H-spiro[chromeno[3,4-c]pyrrole-1,3′-indoline]-3a(9bH)-carboxylate* (**4a**). 188.6 mg, 65%, a white solid; >20:1 *dr,* mp: 182.6–183.9 °C; IR (thin film): *ν*_max_ 3460, 3061, 3035, 2981, 2915, 1755, 1733, 1615, 1468, 1366, 1273, 1207, 1184, 1031, 1006, 837, 782, 752, 631, 551, 530 cm^−1^; ^1^H NMR (400 MHz, DMSO-*d*_6_) δ 8.51 (s, 1H), 7.80 (d, *J* = 6.8 Hz, 1H), 7.72 (d, *J* = 7.6 Hz, 2H), 7.40 (ψt, *J* = 7.6 Hz, 2H), 7.34–7.23 (m, 4H), 7.13 (t, *J* = 7.4 Hz, 1H), 7.05 (ψt, *J* = 7.5 Hz, 2H), 6.76 (ψt, *J* = 7.4 Hz, 1H), 6.70 (d, *J* = 7.2 Hz, 1H), 6.53 (d, *J* = 7.6 Hz, 2H), 6.19 (d, *J* = 7.2 Hz, 1H), 5.77 (s, 1H), 4.87 (d, *J* = 16.2 Hz, 1H), 4.65 (s, 1H), 4.51 (d, *J* = 16.2 Hz, 1H), 3.60–3.48 (m, 2H), 2.22 (s, 3H), 0.65 (t, *J* = 7.2 Hz, 3H); ^13^C NMR (100 MHz, DMSO-*d*_6_): δ 175.1, 167.2, 164.6, 149.1, 144.2, 138.3, 135.8, 131.8, 130.6, 128.9, 128.8, 128.2, 128.1, 127.4, 126.7, 126.6, 126.1, 125.9, 124.9, 124.2, 123.6, 114.8, 109.8, 77.4, 72.5, 62.9, 59.2, 47.2, 42.7, 15.9, 13.5; HRMS (ESI): *m*/*z* calcd for C_35_H_30_N_2_O_6_Na [M+Na]^+^ 597.2002, found 597.2003.

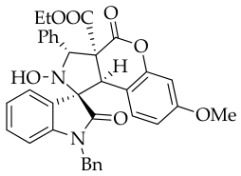

*(±)-Ethyl (1R,3R,3aS,9bR)-1′-benzyl-2-hydroxy-7-methoxy-2′,4-dioxo-3-phenyl-2,3-dihydro-4H-spiro[chromeno[3,4-c]pyrrole-1,3′-indoline]-3a(9bH)-carboxylate* (**4b**). 64.7 mg, 22%, a white solid, >20:1 *dr,* mp: 169.6–171.1 °C; IR (thin film): *ν*_max_ 3478, 2958, 2924, 2853, 1770, 1710, 1613, 1493, 1467, 1361, 1194, 1081, 1013, 955, 856, 747, 595, 643 cm^−1^; ^1^H NMR (400 MHz, DMSO-*d*_6_) δ 8.53 (s, 1H), 7.79 (d, *J* = 6.4 Hz, 1H), 7.71 (d, *J* = 7.2 Hz, 2H), 7.39 (ψt, *J* = 7.4 Hz, 2H), 7.33–7.25 (m, 3H), 7.15 (ψt, *J* = 7.2 Hz, 1H), 7.03 (t, *J* = 7.6 Hz, 2H), 6.74 (d, *J* = 2.0 Hz, 1H), 6.71 (d, *J* = 7.6 Hz, 1H), 6.57 (d, *J* = 7.6 Hz, 2H), 6.48 (dd, *J* = 8.6, 2.2 Hz, 1H), 6.24 (d, *J* = 8.8 Hz, 1H), 5.75 (s, 1H), 4.91 (d, *J* = 16.0 Hz, 1H), 4.59 (s, 1 H), 4.51 (d, *J* = 16.0 Hz, 1H), 3.74 (s, 3H), 3.59–3.52 (m, 2H), 0.65 (t, *J* = 7.0 Hz, 3H); ^13^C NMR (100 MHz, DMSO-*d*_6_): δ 175.2, 167.2, 164.8, 160.9, 151.8, 144.1, 138.3, 135.8, 130.5, 129.1, 128.8, 128.6, 128.2, 128.1, 127.5, 126.8, 126.5, 124.9, 123.6, 111.6, 109.8, 106.8, 102.4, 77.4, 72.3, 62.9, 59.3, 56.0, 46.7, 42.6, 13.5; HRMS (ESI): *m*/*z* calcd for C_35_H_30_N_2_O_7_Na [M+Na]^+^ 613.1952, found 613.1949.

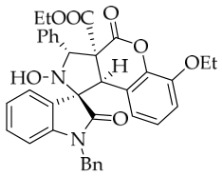

*(±)-Ethyl (1R,3R,3aS,9bR)-1′-benzyl-6-ethoxy-2-hydroxy-2′,4-dioxo-3-phenyl-2,3-dihydro-4H-spiro[chromeno[3,4-c]pyrrole-1,3′-indoline]-3a(9bH)-carboxylate* (**4c**). 261.0 mg, 86%, a white solid, >20:1 *dr,* mp: 143.2–145.1 °C; IR (thin film): *ν*_max_ 3471, 3068, 3039, 2981, 2927, 1774, 1715, 1615, 1587, 1369, 1243, 1181, 1069, 953, 866, 754, 700, 664, 604, 540 cm^−1^; ^1^H NMR (400 MHz, DMSO-*d*_6_) δ 8.51 (s, 1H), 7.80 (d, *J* = 6.8 Hz, 1H), 7.71 (d, *J* = 7.6 Hz, 2H), 7.40 (ψt, *J* = 7.6 Hz, 2H), 7.31 (ψt, *J* = 7.8 Hz, 2H), 7.27 (t, *J* = 7.6 Hz, 1H), 7.14 (ψt, *J* = 7.2 Hz, 1H), 7.12–7.05 (m, 3H), 6.81 (ψt, *J* = 8.0 Hz, 1H), 6.68 (d, *J* = 7.2 Hz, 1H), 6.54 (d, *J* = 7.6 Hz, 2H), 5.91 (d, *J* = 7.6 Hz, 1H), 5.74 (s, 1H), 4.89 (d, *J* = 16.4 Hz, 1H), 4.67 (s, 1H), 4.52 (d, *J* = 16.4 Hz, 1H), 4.11–3.97 (m, 2H), 3.61–3.48 (m, 2H), 1.35 (t, *J* = 7.0 Hz, 3H), 0.65 (t, *J* = 7.0 Hz, 3H); ^13^C NMR (100 MHz, DMSO-*d*_6_): δ 175.1, 167.1, 164.4, 146.8, 144.2, 140.0, 138.3, 135.7, 130.6, 128.9, 128.8, 128.2, 128.1, 127.4, 126.6, 126.5, 124.9, 124.8, 123.7, 119.2, 115.9, 113.8, 109.9, 77.3, 72.5, 64.6, 63.0, 59.0, 47.1, 42.6, 15.0, 13.4; HRMS (ESI): *m*/*z* calcd for C_36_H_32_N_2_O_7_Na [M+Na]^+^ 627.2107, found 627.2110.

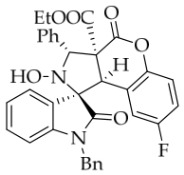

*(±)-Ethyl (1R,3R,3aS,9bR)-1′-benzyl-8-fluoro-2-hydroxy-2′,4-dioxo-3-phenyl-2,3-dihydro-4H-spiro[chromeno[3,4-c]pyrrole-1,3′-indoline]-3a(9bH)-carboxylate* (**4d**).236.4 mg, 82%, a yellow solid, >20:1 *dr*, mp: 177.4–178.2 °C; IR (thin film): *ν*_max_ 3471, 3068, 3018, 2977, 2909, 1770, 1731, 1615, 1495, 1467, 1369, 1250, 1183, 1007, 874, 753, 700, 623, 542 cm^−1^; ^1^H NMR (400 MHz, DMSO-*d*_6_) δ 8.59 (s, 1H), 7.82 (d, *J* = 7.2 Hz, 1H), 7.70 (d, *J* = 7.2 Hz, 2H), 7.40 (ψt, *J* = 7.4 Hz, 2H), 7.35–7.26 (m, 4H), 7.22 (dd, *J* = 8.8, 4.8 Hz, 1H), 7.16 (ψt, *J* = 7.2 Hz, 1H), 7.09 (ψt, *J* = 7.4 Hz, 2H), 6.76 (d, *J* = 7.6 Hz, 1H), 6.60 (d, *J* = 7.6 Hz, 2H), 6.06 (d, *J* = 8.0 Hz, 1H), 5.74 (s, 1H), 4.90 (d, *J* = 16.0 Hz, 1H), 4.69 (s, 1H), 4.53 (d, *J* = 16.0 Hz, 1H), 3.62–3.49 (m, 2H), 0.65 (t, *J* = 7.2 Hz, 3H); ^13^C NMR (100 MHz, DMSO-*d*_6_): δ 175.0, 167.0, 164.4, 147.2 (d, *J* = 2.0 Hz), 144.1, 138.1, 135.8, 130.8, 128.9, 128.2, 127.6, 126.8, 126.0, 125.1, 123.8, 119.5 (d, *J* = 9.6 Hz), 117.8, 117.6, 116.9, 116.8, 114.5, 114.2, 110.1, 77.3, 72.4, 63.0, 58.8, 46.8, 42.7, 13.4; ^19^F NMR (376 MHz, DMSO-*d*_6_): -118.0; HRMS (ESI): *m*/*z* calcd for C_34_H_27_N_2_O_6_FNa [M+Na]^+^ 601.1751, found 601.1754.

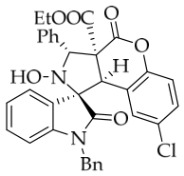

*(±)-Ethyl (1R,3R,3aS,9bR)-1′-benzyl-8-chloro-2-hydroxy-2′,4-dioxo-3-phenyl-2,3-dihydro-4H-spiro[chromeno[3,4-c]pyrrole-1,3′-indoline]-3a(9bH)-carboxylate* (**4e**). 204.2 mg, 67%, a white solid, >20:1 *dr,* mp: 168.8–170.0 °C; IR (thin film): *ν*_max_ 3448, 3098, 3059, 3022, 2977, 2913, 1774, 1725, 1707, 1489, 1468, 1381, 1255, 1255, 1161, 1138, 1007, 885, 750, 701, 615, 557, 534 cm^−1^; ^1^H NMR (400 MHz, DMSO-*d*_6_) δ 8.58 (s, 1H), 7.83 (d, *J* = 7.2 Hz, 1H), 7.70 (d, *J* = 7.6 Hz, 2H), 7.46 (dd, *J* = 8.6, 2.6 Hz, 1H), 7.39 (ψt, *J* = 7.4 Hz, 2H), 7.36–7.24 (m, 3H), 7.19 (d, *J* = 8.8 Hz, 1H), 7.17 (ψt, *J* = 7.2 Hz, 1H), 7.10 (ψt, *J* = 7.6 Hz, 2H), 6.75 (d, *J* = 7.6 Hz, 1H), 6.58 (d, *J* = 7.6 Hz, 2H), 6.32 (d, *J* = 2.4 Hz, 1H), 5.73 (s, 1H), 4.91 (d, *J* = 16.0 Hz, 1H), 4.69 (s, 1H), 4.53 (d, *J* = 16.0 Hz, 1H), 3.62–3.50 (m, 2H), 0.66 (t, *J* = 7.2 Hz, 3H); ^13^C NMR (100 MHz, DMSO-*d*_6_): δ 175.0, 166.9, 164.2, 149.6, 144.1, 138.0, 135.7, 130.8, 130.6, 128.9, 128.8, 128.6, 128.2 (2C), 127.8, 127.6, 126.6, 125.9, 125.1, 123.8, 119.5, 117.2, 110.0, 77.3, 72.4, 63.1, 58.9, 46.6, 42.7, 13.4; HRMS (ESI): *m*/*z* calcd for C_34_H_27_N_2_O_6_ClNa [M+Na]^+^ 617.1455, found 617.1450.

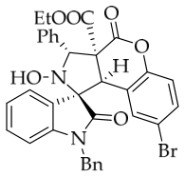

*(±)-Ethyl (1R,3R,3aS,9bR)-1′-benzyl-8-bromo-2-hydroxy-2′,4-dioxo-3-phenyl-2,3-dihydro-4H-spiro[chromeno[3,4-c]pyrrole-1,3′-indoline]-3a(9bH)-carboxylate* (**4f**). 214.3 mg, 67%, a white solid, >20:1 *dr,* mp: 159.1–161.2 °C; IR (thin film): *ν*_max_ 3453, 3064, 3026, 2983, 2930, 1776, 1725, 1615, 1467, 1227, 1163, 1075, 999, 824, 749, 700, 614, 535 cm^−1^; ^1^H NMR (400 MHz, DMSO-*d*_6_) δ 8.58 (s, 1H), 7.83 (d, *J* = 7.2 Hz, 1H), 7.70 (d, *J* = 7.6 Hz, 2H), 7.58 (dd, *J* = 8.8, 2.0 Hz, 1H), 7.40 (ψt, *J* = 7.4 Hz, 2H), 7.37 (ψt, *J* = 8.0 Hz, 1H), 7.33–7.29 (m, 2 H), 7.17 (ψt, *J* = 7.2 Hz, 1H), 7.12 (s, 1 H), 7.11 (ψt, *J* = 7.2 Hz, 2H), 6.75 (d, *J* = 7.6 Hz, 1H), 6.57 (d, *J* = 7.6 Hz, 2H), 6.45 (d, *J* = 2.4 Hz, 1H), 5.73 (s, 1H), 4.91 (d, *J* = 16.0 Hz, 1H), 4.69 (s, 1H), 4.53 (d, *J* = 16.0 Hz, 1H), 3.62–3.50 (m, 2H), 0.66 (t, *J* = 7.0 Hz, 3H); ^13^C NMR (100 MHz, DMSO-*d*_6_): δ 175.0, 166.9, 164.2, 150.1, 144.1, 138.0, 135.7, 133.4, 130.8, 130.7, 128.9, 128.8, 128.2 (2C), 127.5, 126.5, 125.9, 125.1, 123.8, 119.8, 117.7, 116.5, 110.0, 77.3, 72.4, 63.1, 58.9, 46.6, 42.7, 13.4; HRMS (ESI): *m*/*z* calcd for C_34_H_27_N_2_O_6_BrNa [M+Na]^+^ 661.0950, found 661.0933.

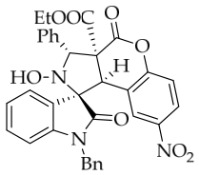

*(±)-Ethyl (1R,3R,3aS,9bR)-1′-benzyl-2-hydroxy-8-nitro-2′,4-dioxo-3-phenyl-2,3-dihydro-4H-spiro[chromeno[3,4-c]pyrrole-1,3’-indoline]-3a(9bH)-carboxylate* (**4g**). 228.0 mg, 75%, a white solid, >20:1 *dr*, mp: 142.8–143.6 °C; IR (thin film): *ν*_max_ 3904, 3740, 3079, 1784, 1714, 1613, 1528, 1343, 1246, 1149, 996, 844, 742, 699 cm^−1^; ^1^H NMR (400 MHz, DMSO-*d*_6_) δ 8.66 (s, 1H), 8.23 (dd, *J* = 9.2, 2.8 H, 1H), 7.89 (dd, *J* = 7.0, 1.0 Hz, 1H), 7.71 (d, *J* = 7.6 Hz, 2H), 7.44–7.31 (m, 6H), 7.14 (d, *J* = 2.4 Hz, 1H), 7.11 (d, *J* = 7.6 Hz, 1H), 7.02 (ψt, *J* = 7.6 Hz, 2H), 6.82 (d, *J* = 8.0 Hz, 1H), 6.60 (d, *J* = 7.2 Hz, 2H), 5.76 (s, 1H), 4.85 (d, *J* = 16.0 Hz, 2H), 4.82 (s, 1 H), 4.51 (d, *J* = 16.0 Hz, 1H), 3.63–3.50 (m, 2H), 0.67 (t, *J* = 7.2 Hz, 3H); ^13^C NMR (100 MHz, DMSO-*d*_6_): δ 175.0, 166.6, 163.7, 155.3, 144.0, 143.5, 137.8, 135.9, 131.0, 128.9, 128.7, 128.3, 127.7, 127.0, 126.4, 125.5, 125.3, 124.0, 123.9, 119.0, 116.6, 110.2, 77.3, 72.5, 63.2, 58.7, 46.4, 42.8, 13.4 (One carbon atom is missing in the ^13^C NMR spectrum. The reason may be that the NMR signals of one carbon atom of the benzene ring overlap with those of other carbon atoms of the benzene ring.); HRMS (ESI): *m*/*z* calcd for C_34_H_27_N_3_O_8_Na [M+Na]^+^ 628.1696, found 6281.1693.

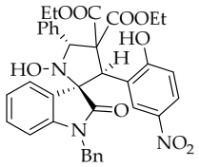

*(±)-Diethyl (3R,3′R,5′R)-1-benzyl-1′-hydroxy-3′-(2-hydroxy-5-nitrophenyl)-2-oxo-5′-phenylspiro[indoline-3,2′-pyrrolidine]-4′,4′-dicarboxylate* (**4g’**). 77.4 mg, 24%, a white solid, >20:1 *dr*, mp: 178.1–180.0 °C; IR (thin film): *ν*_max_ 3852, 3732, 3676, 3649, 3622, 1725, 1618, 1496, 1339, 1289, 1195, 1098, 1020, 803, 753, 702, 611 cm^−1^; ^1^H NMR (400 MHz, DMSO-*d*_6_) δ 11.02 (s, 1H), 8.32 (d, *J* = 2.8 Hz, 1H), 8.20 (s, 1H), 7.97 (dd, *J* = 9.2, 2.8 Hz, 1H), 7.71 (dd, *J* = 5.8, 2.2 Hz, 1H), 7.51 (d, *J* = 7.6 Hz, 2H), 7.35 (ψt, *J* = 7.4 Hz, 2H), 7.28 (t, *J* = 7.2 Hz, 1H), 7.18–7.11 (m, 3H), 7.05 (ψt, *J* = 7.4 Hz, 2H), 6.84 (d, *J* = 7.6 Hz, 2H), 6.75 (d, *J* = 9.2 Hz, 1H), 6.60 (dd, *J* = 6.8, 1.6 Hz, 1H), 6.46 (s, 1H), 5.85 (s, 1H), 4.90 (d, *J* = 15.8 Hz, 1H), 4.55 (d, *J* = 15.8 Hz, 1H), 3.88–3.80 (m, 1H), 3.72–3.64 (m, 1H), 3.60–3.52 (m, 1H), 3.22–3.14 (m, 1H), 0.67 (t, *J* = 7.0 Hz, 3H), 0.59 (t, *J* = 7.2 Hz, 3H); ^13^C NMR (100 MHz, DMSO-*d*_6_): δ 174.8, 169.2, 168.4, 162.7, 143.7, 139.3, 138.6, 136.3, 129.8, 128.6, 128.5, 128.1, 128.0, 127.9, 127.6, 127.1, 125.4, 125.1, 122.9, 122.8, 115.6, 109.0, 76.9, 70.7, 65.6, 61.7, 61.2, 43.3, 42.6, 13.5, 13.4 (One carbon atom is missing in the ^13^C NMR spectrum. The reason may be that the NMR signals of one carbon atom of the benzene ring overlap with those of other carbon atoms of the benzene ring.); HRMS (ESI): *m*/*z* calcd for C_36_H_33_N_3_O_9_Na [M+Na]^+^ 674.2114, found 674.2107.

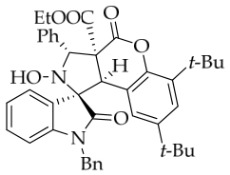

*(±)-Ethyl (1R,3R,3aS,9bR)-1′-benzyl-6,8-di-tert-butyl-2-hydroxy-2′,4-dioxo-3-phenyl-2,3-dihydro-4H-spiro[chromeno[3,4-c]pyrrole-1,3′-indoline]-3a(9bH)-carboxylate* (**4h**). 246.1 mg, 73%, a white solid, >20:1 *dr*, mp: 145.1–146.8 °C; IR (thin film): *ν*_max_ 3065, 3035, 2962, 2907, 2867, 1774, 1720, 1615, 1468, 1365, 1225, 1163, 1124, 1007, 750, 699, 631, 546 cm^−1^; ^1^H NMR (400 MHz, DMSO-*d*_6_) δ 8.53 (s, 1H), 7.82 (d, *J* = 6.8 Hz, 1H), 7.73 (d, *J* = 7.6 Hz, 2H), 7.40 (ψt, *J* = 7.2 Hz, 2H), 7.36–7.29 (m, 3H), 7.24 (s, 1H), 7.14 (ψt, *J* = 7.4 Hz, 1H), 7.01 (ψt, *J* = 7.4 Hz, 2H), 6.74 (d, *J* = 7.2 Hz, 1H), 6.45 (d, *J* = 7.6 Hz, 2H), 6.12 (s, 1H), 5.83 (s, 1H), 4.94 (d, *J* = 16.0 Hz, 1H), 4.52 (s, 1H), 4.35 (d, *J* = 16.0 Hz, 1H), 3.63–3.50 (m, 2H), 1.36 (s, 9H), 0.73 (s, 9H), 0.68 (t, *J* = 7.0 Hz, 3H); ^13^C NMR (100 MHz, DMSO-*d*_6_): δ 175.0, 167.4, 126.6, 164.3, 147.1, 145.4, 144.3, 138.4, 136.4, 136.1, 130.4, 128.9, 128.7, 128.2, 128.0, 127.3, 126.7, 124.9, 124.0, 123.5, 123.3, 114.5, 109.6, 77.6, 72.4, 62.7, 58.6, 48.1, 42.9, 35.1, 34.1, 31.0, 30.0, 13.6; HRMS (ESI): *m*/*z* calcd for C_42_H_44_N_2_O_6_Na [M+Na]^+^ 695.3097, found 695.3082.

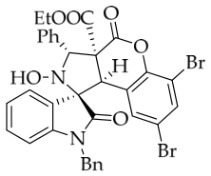

*(±)-Ethyl (1R,3R,3aS,9bR)-1′-benzyl-6,8-dibromo-2-hydroxy-2′,4-dioxo-3-phenyl-2,3-dihydro-4H-spiro[chromeno[3,4-c]pyrrole-1,3′-indoline]-3a(9bH)-carboxylate* (**4j**). 109.1 mg, 30%, a yellow solid; >20:1 *dr*, mp: 147.9–149.1 °C; IR (thin film): *v*_max_ 3472, 3070, 3035, 2983, 2940, 1789, 1714, 1614, 1495, 1369, 1238, 1155, 1125, 1000, 862, 752, 699, 546 cm^−1^; ^1^H NMR (400 MHz, DMSO-*d*_6_) δ 8.61 (s, 1H), 7.93 (d, *J* = 2.0 Hz, 1H), 7.83 (d, *J* = 7.2 Hz, 1H), 7.69 (d, *J* = 7.2 Hz, 2H), 7.40 (ψt, *J* = 7.6 Hz, 2H), 7.39 (t, *J* = 7.4 Hz, 1H), 7.32 (ψt, *J* = 6.6 Hz, 1H), 7.31 (ψt, *J* = 7.0 Hz, 1H), 7.19 (ψt, *J* = 7.2 Hz, 1H), 7.13 (ψt, *J* = 7.4 Hz, 2H), 6.83 (d, *J* = 7.6 Hz, 1H), 6.76 (d, *J* = 7.2 Hz, 2H), 6.41 (d, *J* = 2.0 Hz, 1H), 5.73 (s, 1H), 4.91 (d, *J* = 16.0 Hz, 1H), 4.72 (s, 1H), 4.55 (d, *J* = 15.7 Hz, 1H), 3.63–3.50 (m, 2H), 0.66 (t, *J* = 7.2 Hz, 3H). ^13^C NMR (100 MHz, DMSO-*d*_6_): δ 175.0, 166.5, 163.5, 147.1, 144.1, 137.8, 136.0, 135.8, 130.9, 130.2, 128.9, 128.3, 127.7, 126.7, 125.6, 125.2, 123.9, 118.9, 116.5, 111.6, 110.1, 77.2, 72.5, 63.2, 58.9, 46.7, 42.8, 13.4 (Two carbon atoms are missing in the ^13^C NMR spectrum. The reason may be that the NMR signals of two carbon atoms of the benzene ring overlap with those of other carbon atoms of the benzene ring.); HRMS (ESI): *m*/*z* calcd for C_34_H_26_N_2_O_6_Br_2_Na [M+Na]^+^ 741.0035, found 741.0034.

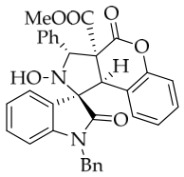

*(±)-Methyl (1R,3R,3aS,9bR)-1′-benzyl-2-hydroxy-2′,4-dioxo-3-phenyl-2,3-dihydro-4H-spiro[chromeno[3,4-c]pyrrole-1,3′-indoline]-3a(9bH)-carboxylate* (**4k**). 81.3 mg, 30%, a white solid, >20:1 *dr*, mp: 158.8–159.6 °C; IR (thin film): *ν*_max_ 3854, 3555, 3474, 3185, 3068, 2915, 1744, 1709, 1610, 1491, 1460, 1253, 1178, 948, 756, 705, 604, 526 cm^−1^; ^1^H NMR (400 MHz, DMSO-*d*_6_) δ 8.56 (s, 1H), 7.83 (d, *J* = 6.6 Hz, 1H), 7.70 (d, *J* = 7.6 Hz, 2H), 7.40 (ψt, *J* = 7.4 Hz, 3H), 7.32 (ψt, *J* = 7.2 Hz, 2H), 7.28 (t, *J* = 7.6 Hz, 1H), 7.13 (d, *J* = 8.4 Hz, 1H), 7.12 (d, *J* = 7.2 Hz, 1H), 7.05 (ψt, *J* = 7.6 Hz, 2H), 6.90 (ψt, *J* = 7.6 Hz, 1H), 6.69 (d, *J* = 7.6 Hz, 1H), 6.53 (d, *J* = 7.6 Hz, 2H), 6.38 (d, *J* = 7.6 Hz, 1H), 5.76 (s, 1H), 4.88 (d, *J* = 16.0 Hz, 1H), 4.68 (s, 1H), 4.51 (d, *J* = 16.0 Hz, 1H), 3.07 (s, 3H); ^13^C NMR (100 MHz, DMSO-*d*_6_): δ 175.1, 167.7, 164.5, 150.8, 144.1, 138.2, 135.7, 130.6, 128.9, 128.7, 128.4, 128.2, 128.1, 127.4, 126.7, 126.3, 125.0, 124.9, 123.7, 117.5, 115.1, 109.9, 77.4, 72.4, 59.5, 53.5, 46.9, 42.6 (One carbon atom is missing in the ^13^C NMR spectrum. The reason may be that the NMR signals of one carbon atom of the benzene ring overlap with those of other carbon atoms of the benzene ring.); HRMS (ESI): *m*/*z* calcd for C_33_H_26_N_2_O_6_Na [M+Na]^+^ 569.1689, found 569.1700.

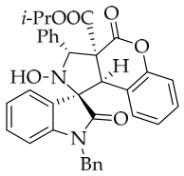

*(±)-Isopropyl (1R,3R,3aS,9bR)-1′-benzyl-2-hydroxy-2′,4-dioxo-3-phenyl-2,3-dihydro-4H-spiro[chromeno[3,4-c]pyrrole-1,3′-indoline]-3a(9bH)-carboxylate* (**4l**). 193.2 mg, 67%, a white solid, >20:1 *dr*, mp: 182.0–183.0 °C; IR (thin film): *ν*_max_ 3474, 3065, 3030, 2989, 2907, 1748, 1731, 1617, 1492, 1371, 1261, 1230, 1261, 1181, 1100, 1004, 908, 753, 701, 610, 553 cm^−1^; ^1^H NMR (600 MHz, CDCl_3_) δ 7.84 (d, *J* = 7.2 Hz, 2H), 7.79 (d, *J* = 6.6 Hz, 1H), 7.39 (ψt, *J* = 7.5 Hz, 2H), 7.31 (ψt, *J* = 7.2 Hz, 1H), 7.27 (td, *J* = 7.8, 1.2 Hz, 2H), 7.25 (td, *J* = 8.1, 0.9 Hz, 1H), 7.12 (ψt, *J* = 7.5 Hz, 1H), 7.09 (dd, *J* = 8.4, 0.6 Hz, 1H), 7.05 (ψt, *J* = 7.8 Hz, 2H), 6.79 (td, *J* = 7.5, 0.9 Hz, 1H), 6.58 (d, *J* = 7.2 Hz, 2H), 6.56 (dd, *J* = 7.2, 1.2 Hz, 1H), 6.42 (d, *J* = 7.2 Hz, 1H), 6.10 (s, 1H), 5.03 (d, *J* = 16.2 Hz, 1H), 4.80 (s, 1H), 4.72 (s, 1H), 4.46 (Sept, *J* = 6.0 Hz, 1H), 4.38 (d, *J* = 16.2 Hz, 1H), 0.95 (d, *J* = 6.0 Hz, 3H), 0.57 (d, *J* = 6.0 Hz, 3H); ^13^C NMR (150 MHz, CDCl_3_): δ 174.4, 166.4, 164.6, 151.1, 144.2, 137.3, 134.9, 130.4, 129.7, 129.1, 128.6, 128.2, 128.1, 127.2, 126.6, 126.0, 124.1, 124.0, 123.3, 117.6, 114.9, 109.8, 77.0, 72.4, 71.3, 59.0, 47.2, 43.4, 21.2, 20.5 (One carbon atom is missing in the ^13^C NMR spectrum. The reason may be that the NMR signals of one carbon atom of the benzene ring overlap with those of other carbon atoms of the benzene ring.); HRMS (ESI): *m*/*z* calcd for C_35_H_30_N_2_O_6_Na [M+Na]^+^ 597.2002, found 597.1975.

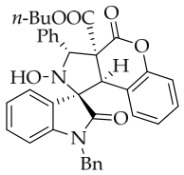

*(±)-n-Butyl (1R,3R,3aS,9bR)-1′-benzyl-2-hydroxy-2′,4-dioxo-3-phenyl-2,3-dihydro-4H-spiro[chromeno[3,4-c]pyrrole-1,3′-indoline]-3a(9bH)-carboxylate* (**4m**). 197.1 mg, 67%, a white solid, >20:1 *dr*, mp: 144.6–145.9 °C; IR (thin film): *ν*_max_ 3474, 3063, 3035, 2958, 2872, 1777, 1716, 1615, 1491, 1455, 1369, 1226, 1174, 1035, 753, 700, 610, 528 cm^−1^; ^1^H NMR (400 MHz, DMSO-*d*_6_) δ 8.53 (s, 1H), 7.80 (d, *J* = 7.2 Hz, 1H), 7.42–7.37 (m, 3H), 7.34–7.27 (m, 3H), 7.14 (d, *J* = 8.0 Hz, 1H), 7.13 (ψt, *J* = 7.2 Hz, 1H), 7.05 (ψt, *J* = 7.4 Hz, 2H), 6.90 (ψt, *J* = 7.4 Hz, 1H), 6.69 (d, *J* = 7.2 Hz, 1H), 6.53 (d, *J* = 7.2 Hz, 2H), 6.38 (d, *J* = 7.6 Hz, 1H), 5.76 (s, 1H), 4.88 (d, *J* = 16.4 Hz, 1H), 4.66 (s, 1H), 4.51 (d, *J* = 16.4 Hz, 1H), 3.57–3.51 (m, 1H), 3.47–3.41 (m, 1H), 1.10–0.99 (m, 4H), 0.73 (t, *J* = 6.8 Hz, 3H); ^13^C NMR (100 MHz, DMSO-*d*_6_): δ 175.1, 167.2, 164.7, 150.3, 144.1, 138.2, 135.7, 130.6, 128.9, 128.8, 128.3, 128.2, 128.1, 127.4, 126.7, 126.4, 124.9, 124.9, 123.7, 117.5, 115.1, 109.9, 77.4, 72.4, 66.5, 59.4, 47.2, 42.6, 29.8, 18.7, 13.9 (One carbon atom is missing in the ^13^C NMR spectrum. The reason may be that the NMR signals of one carbon atom of the benzene ring overlap with those of other carbon atoms of the benzene ring.); HRMS (ESI): *m*/*z* calcd for C_36_H_32_N_2_O_6_Na [M+Na]^+^ 611.2158, found 611.2149.

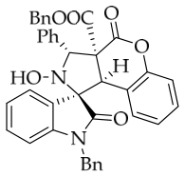

*(±)-Benzyl (1R,3R,3aS,9bR)-1′-benzyl-2-hydroxy-2′,4-dioxo-3-phenyl-2,3-dihydro-4H-spiro[chromeno[3,4-c]pyrrole-1,3′-indoline]-3a(9bH)-carboxylate* (**4n**). 276.9 mg, 89%, a white solid, >20:1 *dr*, mp: 131.9–132.5 °C; IR (thin film): *ν*_max_ 3648, 3415, 3063, 3020, 1968, 1742, 1708, 1613, 1461, 1374, 1217, 1175, 1004, 910, 739, 695, 608, 528 cm^−1^; ^1^H NMR (400 MHz, DMSO-*d*_6_) δ 8.55 (s, 1H), 7.80 (d, *J* = 8.0 Hz, 1H), 7.71 (d, *J* = 7.2 Hz, 2H), 7.40 (t, *J* = 7.2 Hz, 1H), 7.37–7.30 (m, 4H), 7.29–7.27 (m, 4H), 7.13 (t, *J* = 8.4 Hz, 1H), 7.12 (t, *J* = 6.8 Hz, 1H), 7.05 (ψt, *J* = 7.6 Hz, 2H), 6.99–6.96 (m, 2H), 6.90 (ψt, *J* = 7.4 Hz, 1H), 6.69 (d, *J* = 7.6 Hz, 1H), 6.53 (d, *J* = 7.6 Hz, 2H), 6.39 (d, *J* = 7.6 Hz, 1H), 5.78 (s, 1H), 4.88 (d, *J* = 16.2 Hz, 1H), 4.70 (s, 1H), 4.64 (d, *J* = 12.4 Hz, 1H), 4.51 (d, *J* = 16.2 Hz, 1H), 4.37 (d, *J* = 12.4 Hz, 1H); ^13^C NMR (100 MHz, DMSO-*d*_6_): δ 175.1, 167.1, 164.6, 150.8, 144.1, 138.1, 135.7, 134.9, 130.7, 128.9, 128.8, 128.6, 128.4, 128.3, 128.2, 127.9, 127.4, 126.7, 126.3, 125.0, 124.9, 123.7, 117.5, 115.1, 109.9, 77.4, 72.5, 68.1, 59.4, 47.3, 42.6 (Two carbon atoms are missing in the ^13^C NMR spectrum. The reason may be that the NMR signals of two carbon atoms of the benzene ring overlap with those of other carbon atoms of the benzene ring.); HRMS (ESI): *m*/*z* calcd for C_39_H_30_N_2_O_6_Na [M+Na]^+^ 645.2002, found 645.1997.

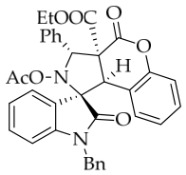

*(±)-Ethyl (1R,3R,3aS,9bR)-2-acetoxy-1′-benzyl-2′,4-dioxo-3-phenyl-2,3-dihydro-4H-spiro[chromeno[3,4-c]pyrrole-1,3′-indoline]-3a(9bH)-carboxylate* (**6a**). 260.7 mg, 87%, a white solid, mp: 194.2–196.0 °C; IR (thin film): *ν*_max_ 3523, 3445, 3210, 3059, 2931, 2859, 1773, 1737, 1707, 1610, 1492, 1367, 1165, 998, 758, 697, 596,495 cm^−1^; ^1^H NMR (400 MHz, DMSO-*d*_6_) δ 7.90 (d, *J* = 6.4 Hz, 1H), 7.80 (d, *J* = 7.2 Hz, 1H), 7.43–7.31 (m, 5H),7.26 (t, *J* = 7.2 Hz, 1H), 7.17–7.08 (m, 4 H), 6.92 (td, *J* = 7.6, 0.8 Hz, 1H), 6.77 (d, *J* = 7.6 Hz, 1H), 6.63 (d, *J* = 7.2 Hz, 2H), 6.39 (d, *J* = 7.2 Hz, 1H), 6.03 (s, 1H), 4.86 (s, 1H), 4.84 (d, *J* = 16.0 Hz, 1H), 4.60 (d, *J* = 16.0 Hz, 1H), 3.64–3.50 (m, 2H), 1.53 (s, 3H), 0.67 (t, *J* = 7.0 Hz, 3H). ^13^C NMR (100 MHz, DMSO-*d*_6_): δ 173.5, 167.8, 166.5, 164.1, 150.8, 143.6, 136.0, 135.6, 131.4, 131.0, 129.0, 128.8, 128.4, 128.3, 127.7, 126.9, 126.5, 125.1, 123.9, 123.3, 117.7, 114.1, 110.2, 76.7, 71.3, 63.3, 59.2, 46.7, 42.8, 18.6, 13.4 (One carbon atom is missing in the ^13^C NMR spectrum. The reason may be that the NMR signals of one carbon atom of the benzene ring overlap with those of other carbon atoms of the benzene ring.); HRMS (ESI): *m*/*z* calcd for C_36_H_30_N_2_O_7_Na [M+Na]^+^ 625.1951, found 625.1953.

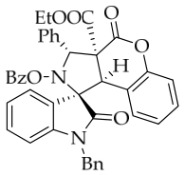

*(±)-Ethyl (1R,3R,3aS,9bR)-2-(benzoyloxy)-1′-benzyl-2′,4-dioxo-3-phenyl-2,3-dihydro-4H-spiro[chromeno[3,4-c]pyrrole-1,3′-indoline]-3a(9bH)-carboxylate* (**6b**). 263.0 mg, 79%, a white solid, mp: 160.4–162.1 °C; IR (thin film): *ν*_max_ 3847, 3648, 3567, 3511, 3237, 2975, 2929, 1766, 1715, 1610, 1459, 1233, 1167, 1017, 753, 701, 554 cm^−1^; ^1^H NMR (400 MHz, DMSO-*d*_6_) δ 7.99 (dd, *J* = 6.4, 2.4 Hz, 1H), 7.89 (d, *J* = 7.2 Hz, 2H), 7.59–7.50 (m, 3H), 7.45–7.36 (m, 5H), 7.32–7.25 (m, 3H), 7.20 (d, *J* = 8.0 Hz, 1H), 7.11 (ψt, *J* = 7.4 Hz, 1H), 6.98–6.92 (m, 3 H), 6.67 (dd, *J* = 5.6, 3.2 Hz, 1H), 6.57 (d, *J* = 7.2 Hz, 2H), 6.25 (s, 1H), 4.95 (s, 1H), 4.88 (d, *J* = 16.0 Hz, 1H), 4.57 (d, *J* = 16.0 Hz, 1H), 3.67–3.54 (m, 2H), 0.69 (t, *J* = 7.2 Hz, 3H). ^13^C NMR (100 MHz, DMSO-*d*_6_): δ 173.5, 166.5, 164.2, 163.3, 150.8, 143.5, 135.9, 135.4, 134.4, 131.4, 131.2, 129.5, 129.3, 129.0, 128.9, 128.5, 128.4, 127.6, 127.3, 126.8, 126.5, 125.1, 124.0, 123.3, 117.7, 114.0, 110.2, 76.9, 71.6, 63.4, 59.2, 46.9, 42.8, 13.4; HRMS (ESI): *m*/*z* calcd for C_41_H_32_N_2_O_7_Na [M+Na]^+^ 687.2107, found 687.2106.

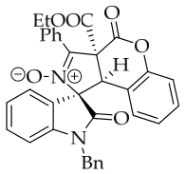

*(±)-(1R,3aS,9bR)-1′-Benzyl-3a-(ethoxycarbonyl)-2′,4-dioxo-3-phenyl-3a,9b-dihydro-4H-spiro [chromeno[3,4-c]pyrrole-1,3′-indoline] 2-oxide* (**8**). 154.0 mg, 55%, a white solid, mp: 295.1–295.8 °C; IR (thin film): *ν*_max_ 3098, 3068, 2975, 2927, 1773, 1746, 1721, 1458, 1374, 1263, 1225, 1150, 1023, 990, 910, 860, 762, 697, 637, 544 cm^−1^; ^1^H NMR (400 MHz, DMSO-*d*_6_) δ 8.43–8.40 (m, 2H), 7.76 (d, *J* = 7.2 Hz, 1H), 7.55–7.51 (m, 4H), 7.46 (t, *J* = 7.4 Hz, 1H), 7.32 (d, *J* = 7.2 Hz, 1H), 7.31 (d, *J* = 8.0 Hz, 1H), 7.17 (ψt, *J* = 7.2 Hz, 1H), 7.10 (ψt, *J* = 7.4 Hz, 1H), 7.06 (ψt, *J* = 7.2 Hz, 1H), 6.90 (d, *J* = 8.0 Hz, 1H), 6.57 (ψd, *J* = 7.2 Hz, 3H), 4.93 (s, 1H), 4.86 (d, *J* = 16.2 Hz, 1H), 4.59 (d, *J* = 16.2 Hz, 1H), 4.29–4.23 (m, 1H), 4.20–4.12 (m, 1H), 0.98 (t, *J* = 7.0 Hz, 3H); ^13^C NMR (100 MHz, DMSO-*d*_6_): δ 170.7, 167.9, 161.3, 150.9, 140.4, 135.1, 132.5, 131.6, 131.4, 129.0, 128.7, 128.4, 127.8, 126.8, 126.4, 125.6, 124.5, 122.8, 117.6, 113.2, 110.8, 85.9, 64.3, 60.3, 47.6, 43.3, 13.9; HRMS (ESI): *m*/*z* calcd for C_34_H_26_N_2_O_6_Na [M+Na]^+^ 581.1689, found 581.1683.

## 4. Conclusions

In summary, we have developed a rapid (3+2)-cycloaddition reaction between in situ generated isatin ketonitrone 1,3-dipoles and coumarins. This method provided access to novel dicyclic spiropyrrolidine oxindole derivatives (>45 examples) in moderate to excellent yields (22–98%). The reaction proceeded under mild conditions and exhibits high regio- and diastereoselectivities (>20:1 *dr*). All synthesized *exo*-type spirooxindole derivatives **3/4** were characterized by ^1^H NMR, ^13^C NMR, ^19^F NMR, IR, and HRMS. The relative stereochemistry of the products was unambiguously confirmed by single-crystal X-ray diffraction analysis of **3b** and **8**. In addition, the successful transformation of a spirooxindole product demonstrated its good synthetic utility. Further exploration and application of this reaction in organic synthesis are ongoing in our laboratory.

## Figures and Tables

**Table 1:** The condition optimization of model reaction *^a^*.

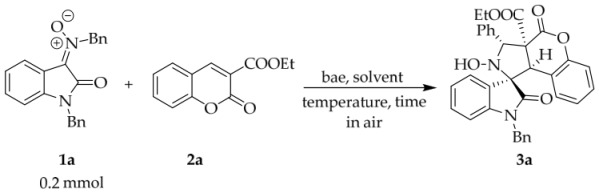		
Entry	Reaction Condition	Yield (%) *^b,c^*
1	DBU, **2a**, DCM, 24 h	- *^d^*
2	DBU, **2a**, DMF, 24 h	complex
3	DBU, **2a**, MeOH, 4 h	58
4	DBU, **2a**, EtOH, 5 h	70
5	DBU, **2a**, IPA, 11 h	60
6	DBU, **2a**, *n*BuOH, 9 h	63
7	DBU, **2a**, *t*BuOH, 4 h	54
8	DABCO, **2a**, EtOH, 4 h	20 *^e^*
9	DMAP, **2a**, EtOH, 8 h	24 *^e^*
10	DBU, **2a**, dry EtOH, 6 h	62
11	DBU, 2.1 eq **2a**, EtOH, 14 h	71
12	50 mol% DBU, **2a**, EtOH, 2.5 h	51
13	100 mol% DBU, **2a**, EtOH, 2.5 h	36
14	DBU, **2a**, 1.0 mL, EtOH, 5 h	74
15	DBU, **2a**, 4.0 mL, EtOH, 9 h	69

*^a^* Unless otherwise indicated, the reaction was performed at the 0.2 mmol scale in a solvent (2 mL) with base (25 mol%) at rt, and the molar ratio of **1a**:**2a** was 1:1.05. *^b^* Isolated yield. *^c^* The selectivity (*dr* > 20:1 in all cases) was determined by ^1^H NMR. *^d^* NR: no reaction. *^e^* The reaction was performed at reflux.

**Table 2:** Synthesis of coumarin-fused spiropyrrolidine oxindoles **3** from isatin ketonitrones **1** and coumarins **2a** *^a^*.

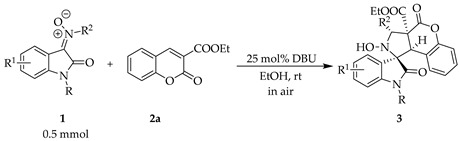					
Entry	Compound	R	R^1^	R^2^	Yield (%) *^b^*
1	**3a**	Bn	H	Ph	74 (74) *^c^*
2	**3b**	Bn	5-Me	Ph	98
3	**3c**	Bn	5-OMe	Ph	82
4	**3d**	Bn	5-F	Ph	82
5	**3** **e**	Bn	5-Cl	Ph	74
6	**3f**	Bn	6-Br	Ph	58
7	**3g**	Bn	5-I	Ph	51
8	**3h**	Bn	5-NO_2_	Ph	- *^d^*
9	**3** **i**	Bn	7-CF_3_	Ph	- *^d^*
10	**3** **j**	H	H	Ph	76
11	**3** **k**	Me	H	Ph	80
12	**3** **l**	Et	H	Ph	78
13	**3** **m**	allyl	H	Ph	85
14	**3** **n**	propargyl	H	Ph	59
15	**3** **o**	H	H	H	- *^e^*
16	**3** **p**	H	H	4-MeC_6_H_4_	66
17	**3q**	H	H	4-OMeC_6_H_4_	54
18	**3** **r**	H	H	4-FC_6_H_4_	78
19	**3** **s**	H	H	4-ClC_6_H_4_	86
20	**3** **t**	H	H	4-BrC_6_H_4_	82
21	**3u**	H	H	4-NO_2_C_6_H_4_	47
22	**3v**	Bn	5-Me	H	- *^d^*
23	**3w**	Bn	5-Me	4-MeC_6_H_4_	77
24	**3x**	Bn	5-Me	4-OMeC_6_H_4_	50
25	**3y**	Bn	5-Me	4-FC_6_H_4_	69
26	**3z**	Bn	5-Me	2-ClC_6_H_4_	48
27	**3a** **a**	Bn	5-Me	4-ClC_6_H_4_	85
28	**3a** **b**	Bn	5-Me	4-BrC_6_H_4_	66
29	**3a** **c**	Bn	5-Me	4-NO_2_C_6_H_4_	52
30	**3ad**	Bn	H	4-MeC_6_H_4_	46
31	**3a** **e**	Bn	H	4-OMeC_6_H_4_	38
32	**3af**	Bn	H	4-FC_6_H_4_	63 *^f^*
33	**3ag**	Bn	H	4-ClC_6_H_4_	76
34	**3ah**	Bn	H	4-BrC_6_H_4_	68
35	**3ai**	Bn	H	4-NO_2_C_6_H_4_	60

*^a^* Reaction conditions: **1a** (0.5 mmol), **2a** (0.525 mmol), and DBU (0.125 mmol), EtOH (2.5 mL), rt, 5–24h. *^b^* Isolated yield via column chromatography. *^c^* The reaction was scaled up to 5 mmol, and the product **3a** was obtained with a quantity of 2.08g. *^d^* The reaction gave a complex mixture. *^e^* Only a trace amount of the product was obtained. *^f^* The reaction was performed in *t*BuOH.

**Table 3:** Synthesis of coumarin-fused spiropyrrolidine oxindoles **4** from istain ketonitrones **1a** and coumarin **2** *^a^*.

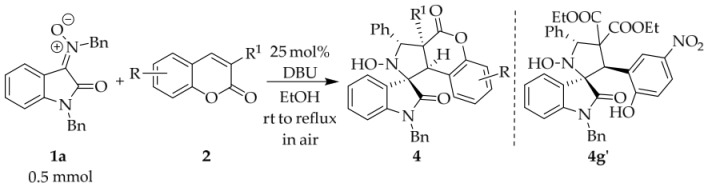				
Entry	Compound	R	R^1^	Yield (%) *^b^*
1	**4a**	8-Me	COOEt	65
2	**4b**	7-OMe	COOEt	22
3	**4c**	8-OEt	COOEt	86
4	**4d**	6-F	COOEt	82
5	**4** **e**	6-Cl	COOEt	67
6	**4f**	6-Br	COOEt	67
7	**4g** (**4g’**)	6-NO_2_	COOEt	75 (24) *^c^*
8	**4h**	6,8-*t*-Bu_2_	COOEt	73
9	**4** **i**	7-Et_2_N	COOEt	- *^d^*
10	**4** **j**	6,8-Br_2_	COOEt	30
11	**4** **k**	H	COOMe	30 *^e^*
12	**4** **l**	H	COO*i*-Pr	67
13	**4m**	H	COO*n*-Bu	67
14	**4n**	H	COOBn	89 *^e^*
15	**4o**	H	H	- *^d^*
16	**4p**	H	Cl	- *^d^*
17	**4q**	H	Br	- *^d^*
18	**4r**	H	Bz	- *^d^*
19	**4s**	H	COOH	- *^d^*
20	**4t**	H	CN	- *^d^*
21	**4u**	H	Ac	- *^d^*
22	**4v**	H	CONH*n*-Bu	- *^d^*
23	**4w**	H	CON*n*-Bu_2_	- *^d^*

*^a^* Reaction conditions: **1** (0.5 mmol), **2a** (0.525 mmol), and DBU (0.125 mmol), EtOH (2.5 mL), rt to reflux. *^b^* Isolated yield via column chromatography. *^c^* The reaction gave the lactone ring-opening product **4g’** at the standard condition, while the reaction could obtain the desired product **4g** using *t*BuOH as a solvent. *^d^* The reaction gave a complex. *^e^* The reaction was performed in *t*BuOH.

## Data Availability

The original contributions presented in this study are included in the article and Supplementary Materials. Further inquiries can be directed to the corresponding author.

## Supplementary Materials

The following supporting information can be downloaded at: https://www.mdpi.com/article/10.3390/molecules31081303/s1. I. Copes of NMR for all new compounds; II. Copes of HRMS for all new compounds; III. Copes of Data of X-ray crystal structure for **3b** (CCDC 2375731); IV. Copes of Data of X-ray crystal structure for **8** (CCDC 2518523).
