# Supplementary material for: DBU-Mediated Diastereoselective (3+2)-Cycloaddition of Isatin Ketonitrones and Coumarins to Construct Coumarin-Fused Spiropyrolidine Oxoindoles
Source: Molecules. 2026 Apr 16;31(8):1303. doi: 10.3390/molecules31081303 (PMC13118662; doi:10.3390/molecules31081303)
Supplement: Supplementary file 1 [file molecules-31-01303-s001.zip › molecules-4221475-supplementary.pdf]

Molecules

Supporting Information

DBU-mediated diastereoselective (3+2)-cycloaddition of isatin ketonitrones and coumarins to construct coumarin-fused spiropyrolidine oxoindoles

Lan Ma [1,†], Qian Zhong [1,†], Zixin Zhang [1], Ruyi Zhou [1], Chunyan Long [3], Wanbing Wu [3], Sicheng Li [1,4], Qiao He [1,5], and Guizhou Yue [1,*]

[1]*College of Science, Sichuan Agricultural University, Ya'an, Sichuan, 625014, China.*
[2]*Sichuan Jisheng Biopharmaceutical Co., Ltd., Leshan, Sichuan, 614000, China.*
[3]*The Yingjing County Emergency Management Agency, Ya'an, Sichuan, 625200, China.*
[4]*Agriculture and Rural Bureau of Daying Country, Suining, Sichuan, 629300, China.*
† These authors contributed equally to this work.

I. Copes of NMR for all new compounds

^{1}H NMR and ^{13}C NMR Spectra for Compound **3a**

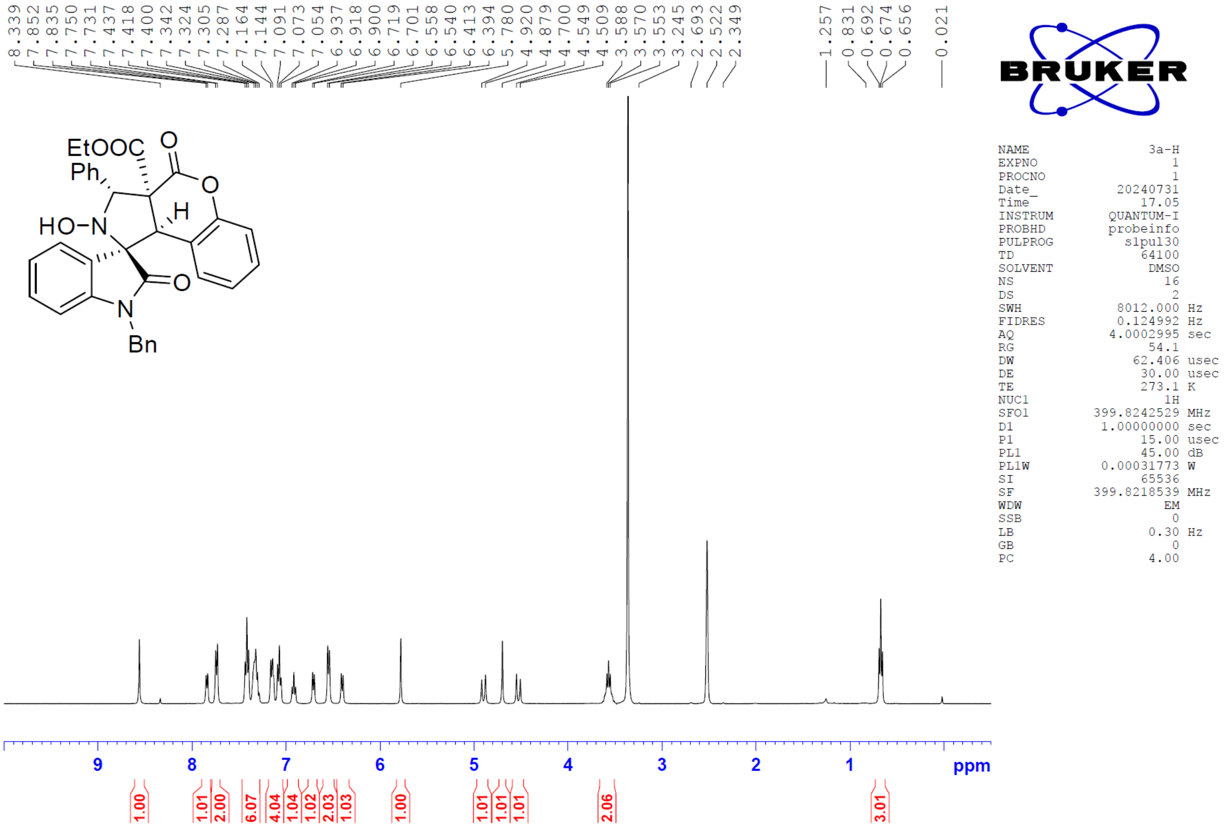

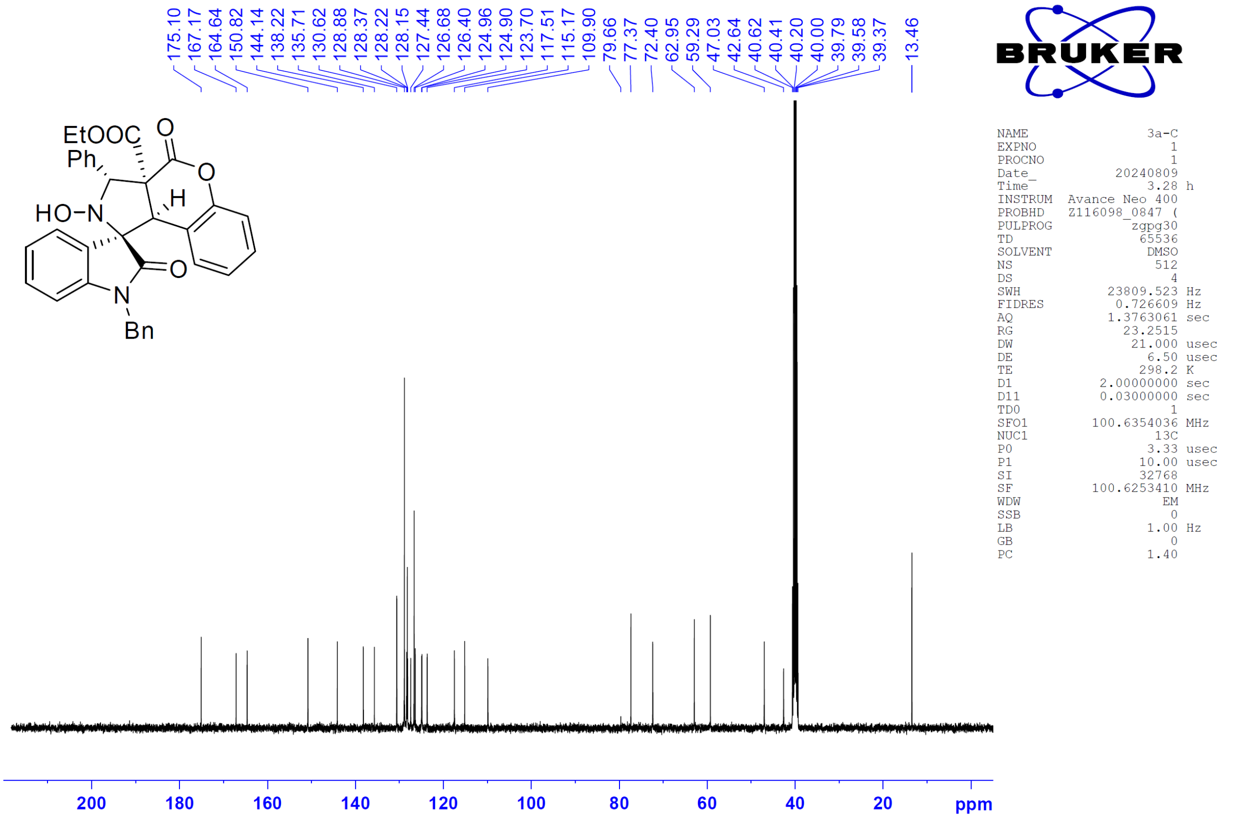

^{1}H NMR Spectra for Compound **3a** (It was obtained during the column chromatography for compound **7**.)

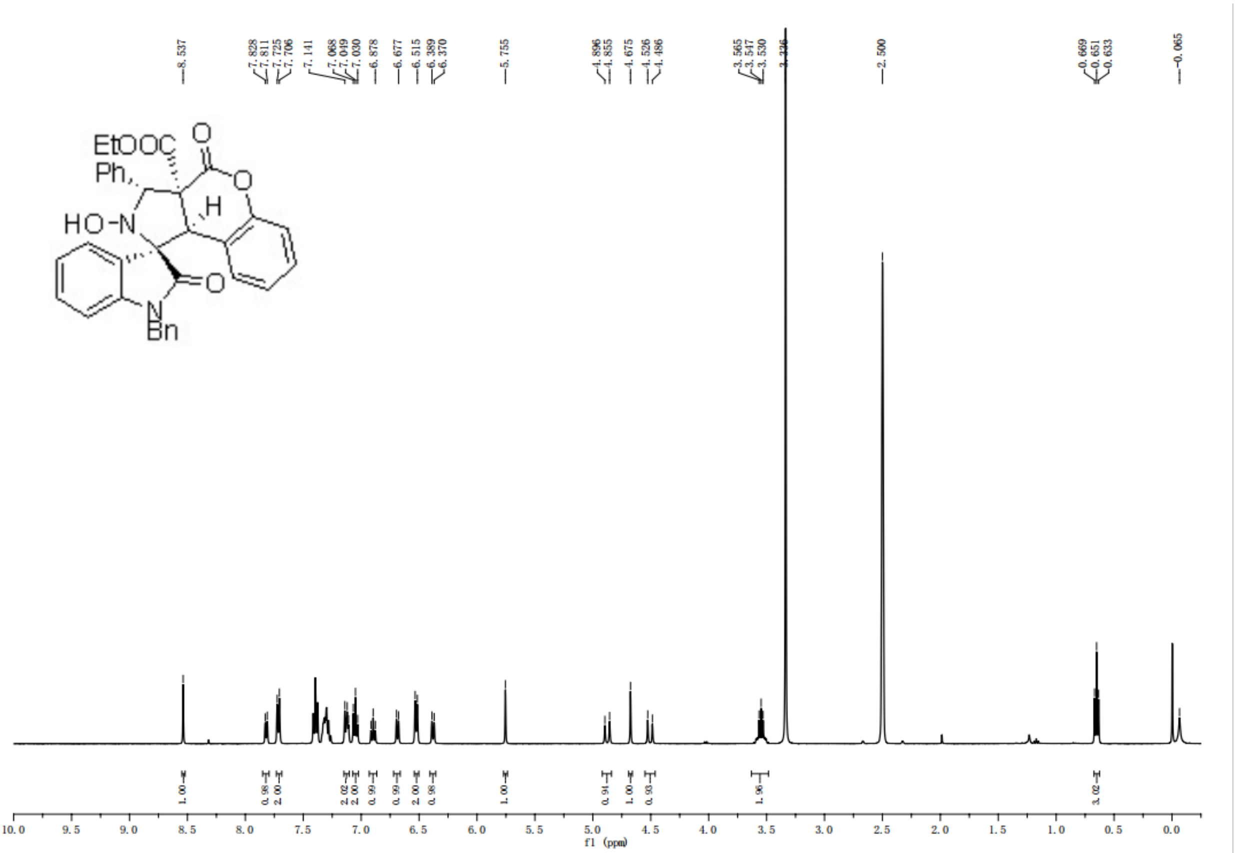

^{1}H NMR Spectrum for Compound **3a** (MeOD)

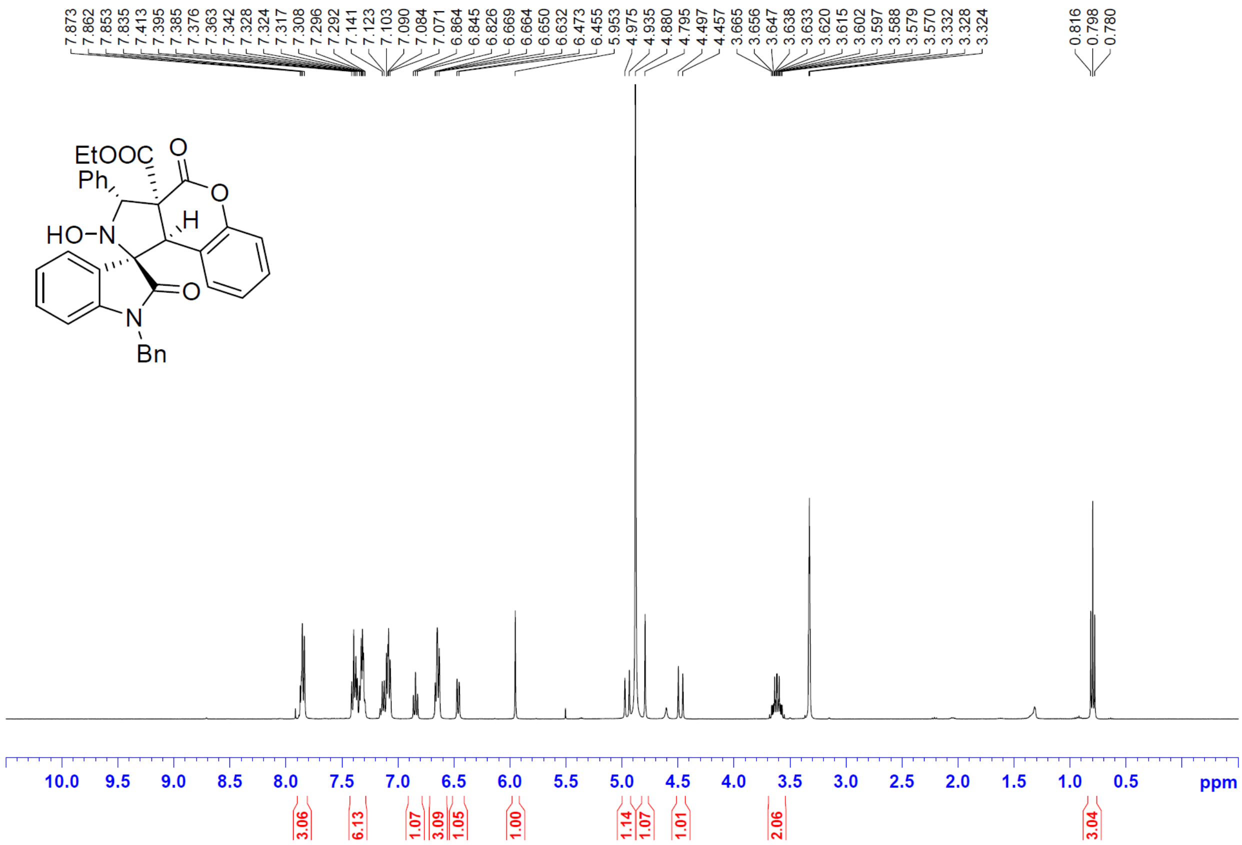

^{1}H NMR Spectrum for Sodium Salt of Compound **7** (MeOD; **3a** was found in the spectrum)

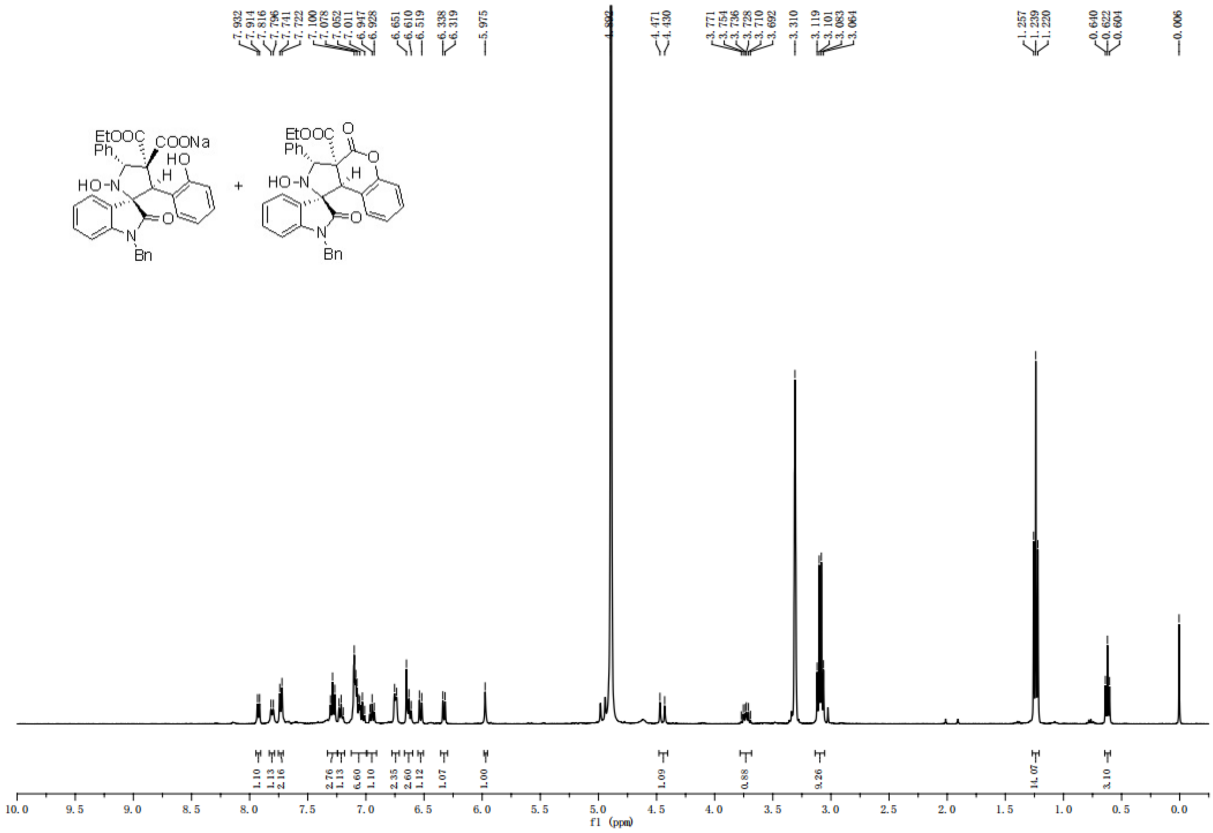

^{1}H NMR and ^{13}C NMR Spectra for Compound **3b**

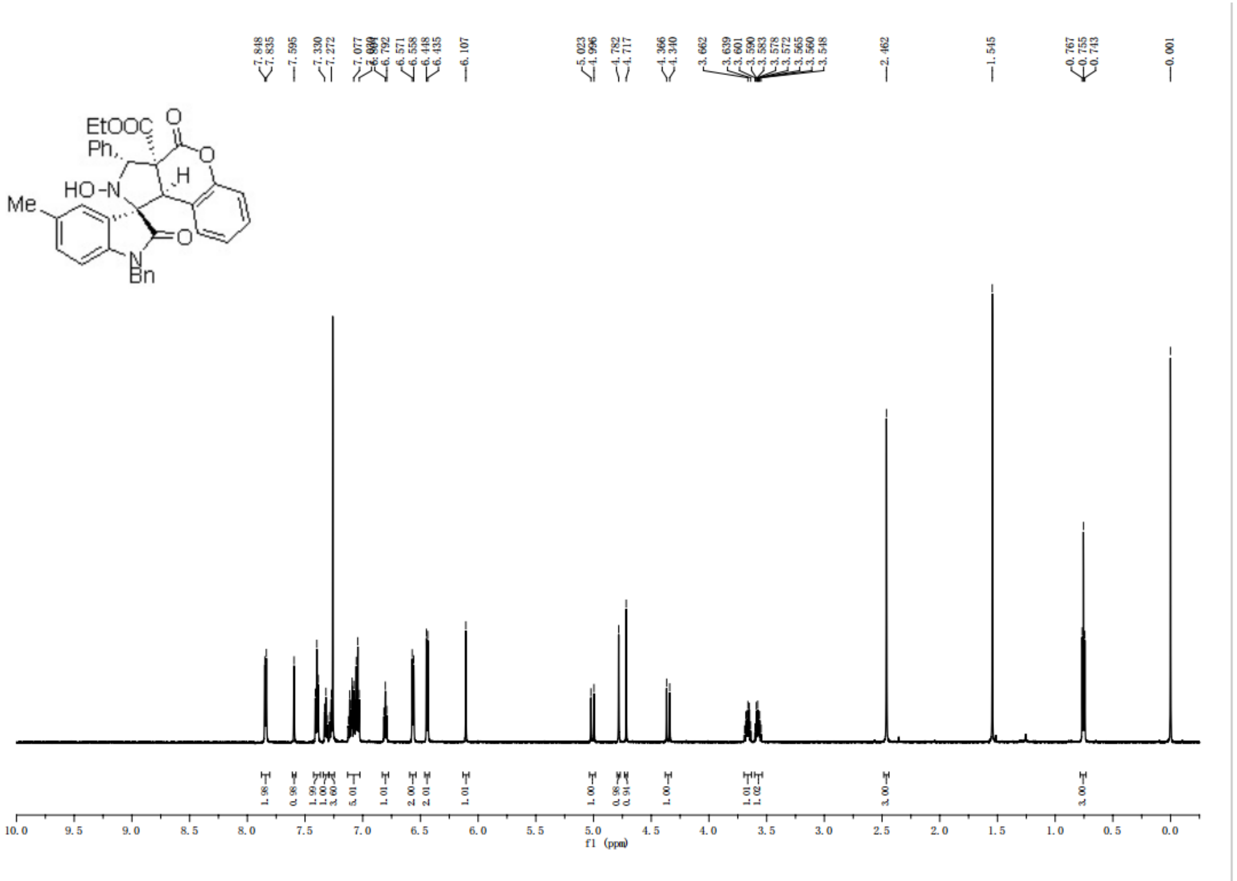

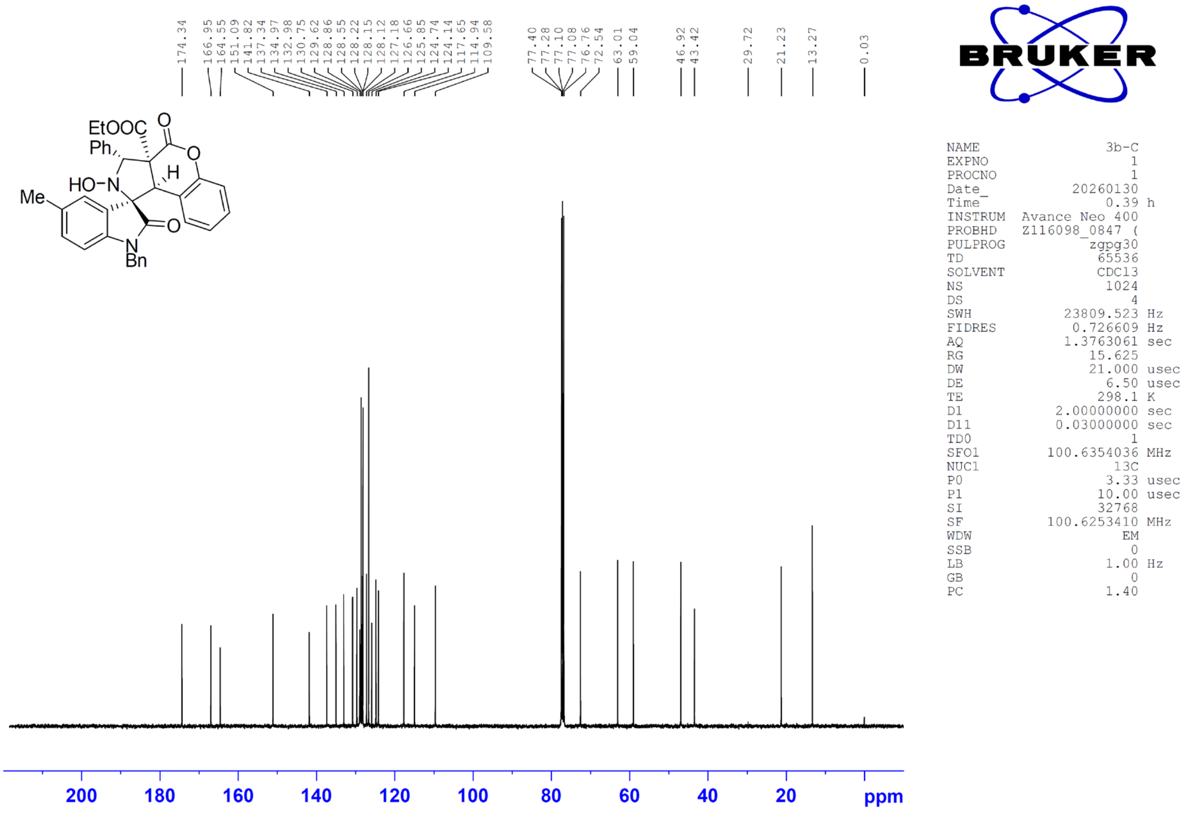

^{1}H NMR and ^{13}C NMR Spectra for Compound **3c**

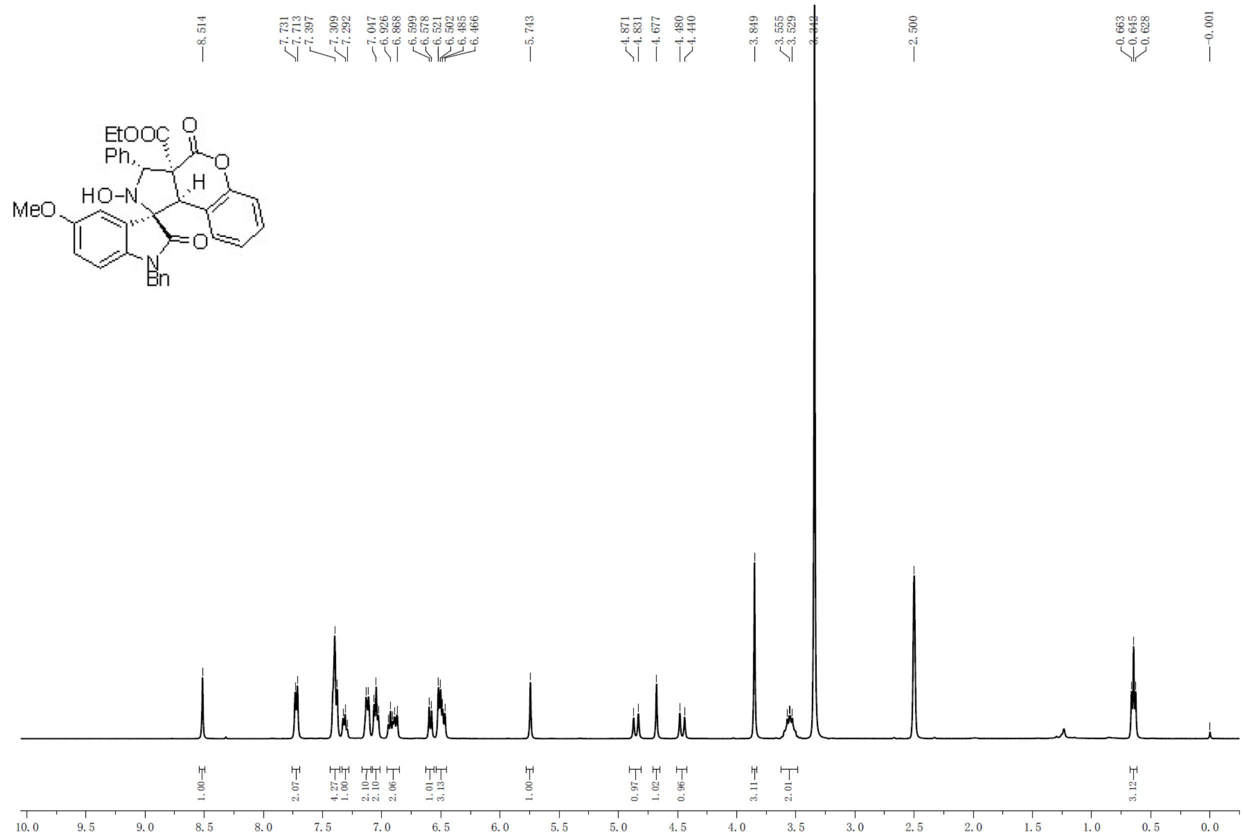

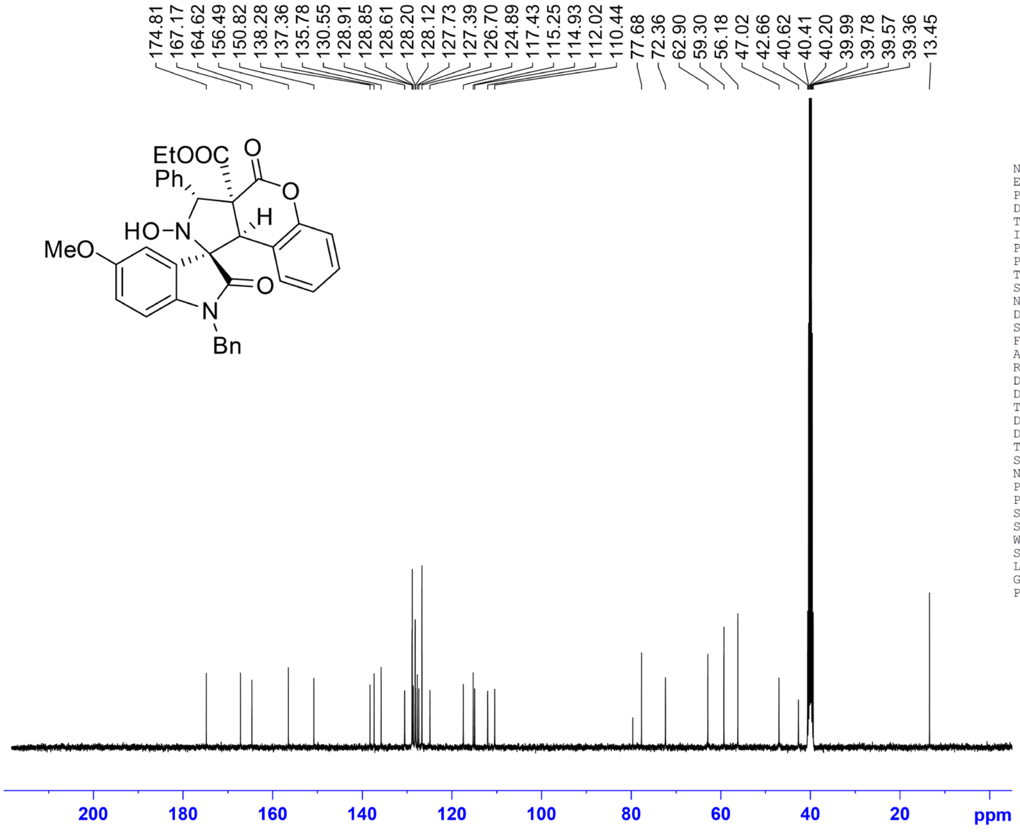


```
NAME       3c-C (5-MeO, N-Bn)
EXPNO                   1
PROCNO                  1
Date_            20250428
Time               6.35 h
INSTRUM    Avance Neo 400
PROBHD     Z116098_0847 (
PULPROG           zgpg30
TD                 65536
SOLVENT             DMSO
NS                   512
DS                     4
SWH            23809.523 Hz
FIDRES          0.726609 Hz
AQ             1.3763061 sec
RG                15.625
DW                21.000 usec
DE                  6.50 usec
TE                 298.2 K
D1            2.00000000 sec
D11           0.03000000 sec
TD0                    1
SFO1         100.6354036 MHz
NUC1                 13C
P0                  3.33 usec
P1                 10.00 usec
SI                 32768
SF           100.6253410 MHz
WDW                   EM
SSB                    0
LB                  1.00 Hz
GB                     0
PC                  1.40
```

^{1}H NMR, ^{13}C NMR and ^{19}F NMR Spectra for Compound **3d**

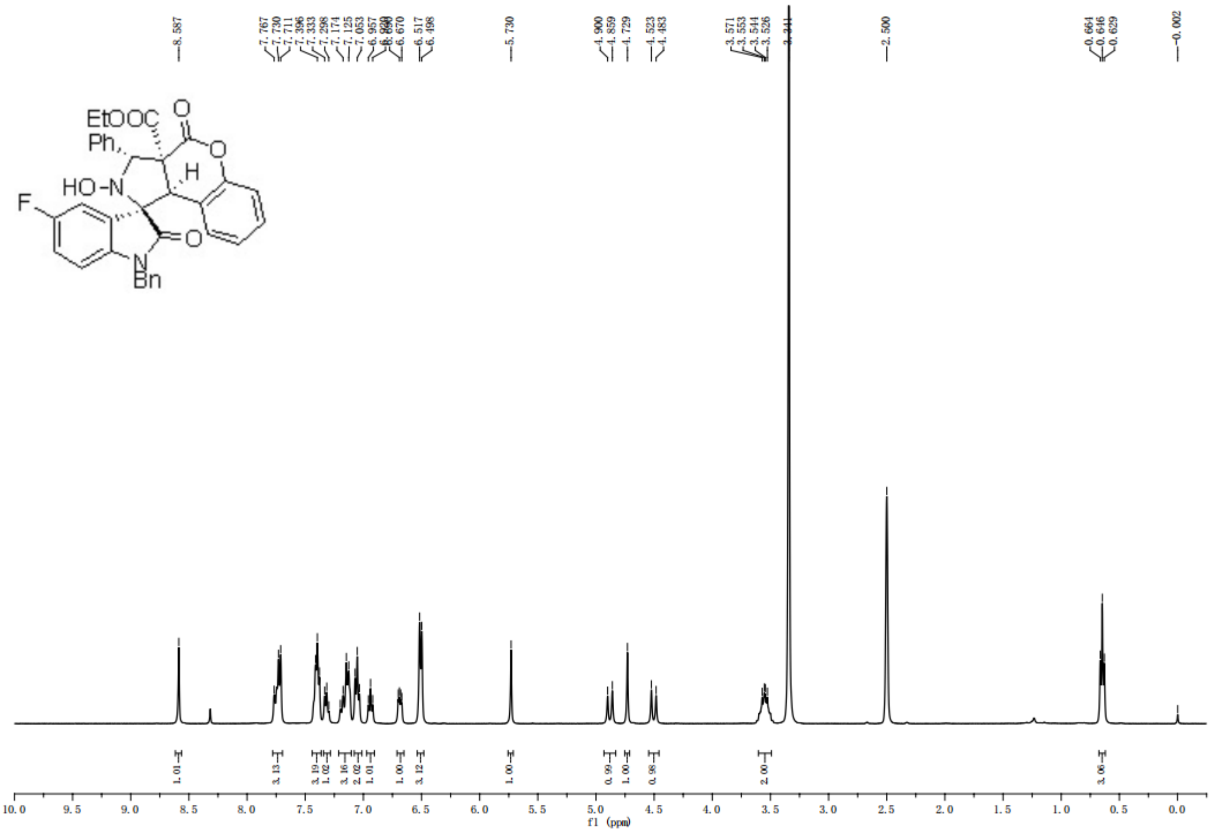

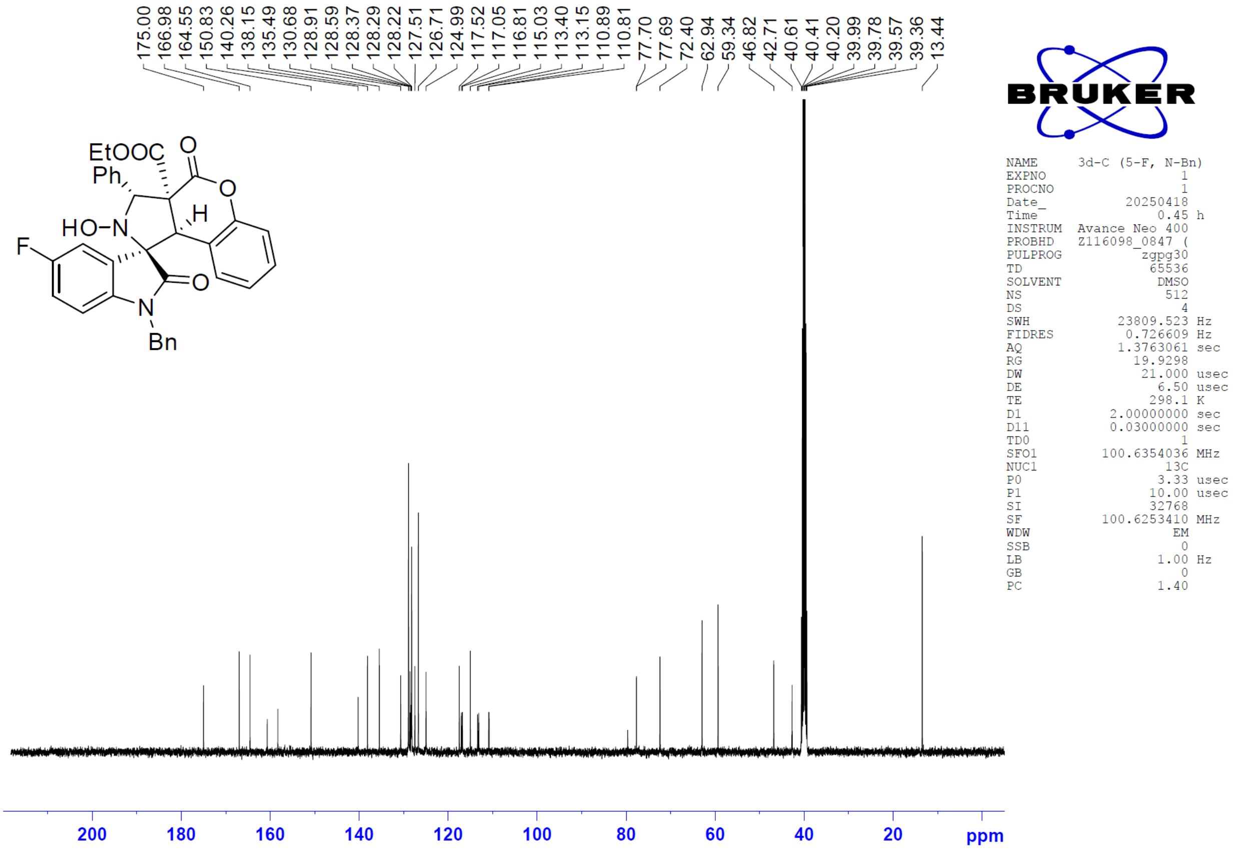

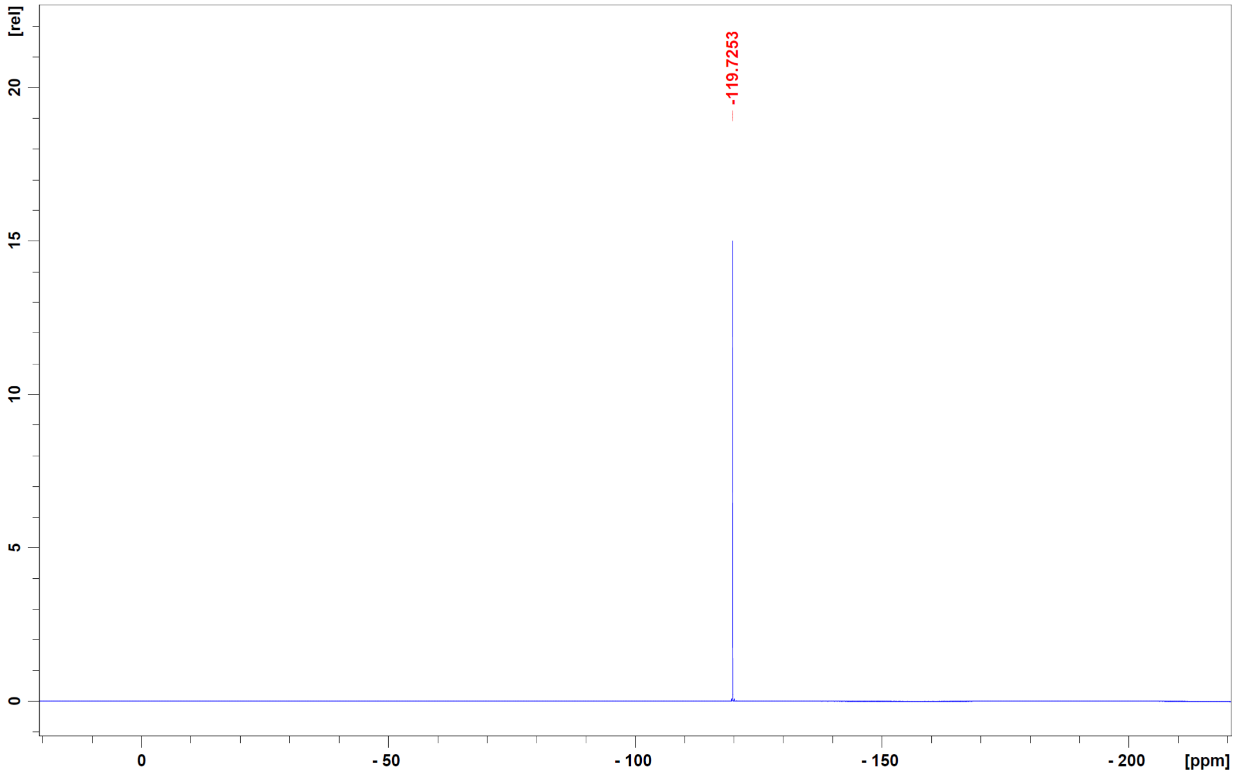

-119.7253
[rel]
20
15
10
5
0
0
- 50
- 100
- 150
- 200
[ppm]

^{1}H NMR, ^{13}C NMR and ^{19}F NMR Spectra for Compound **3e**

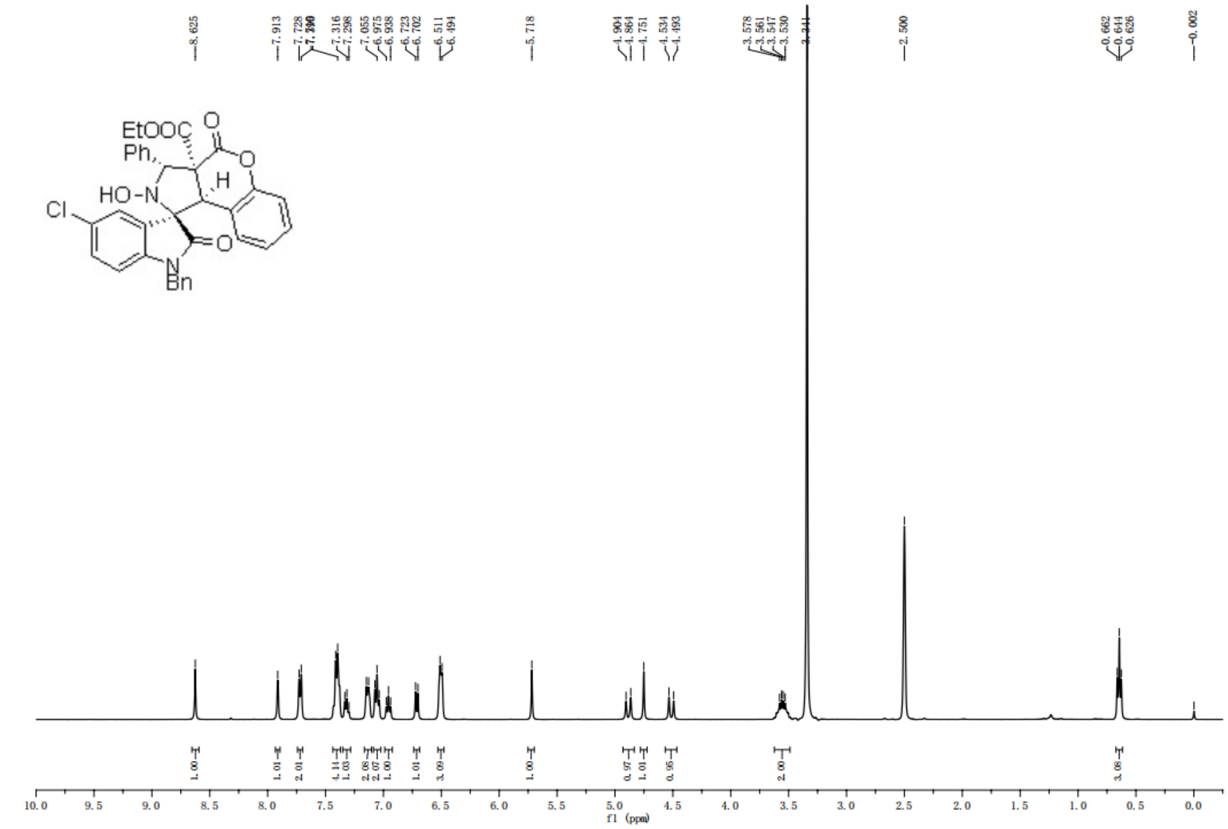

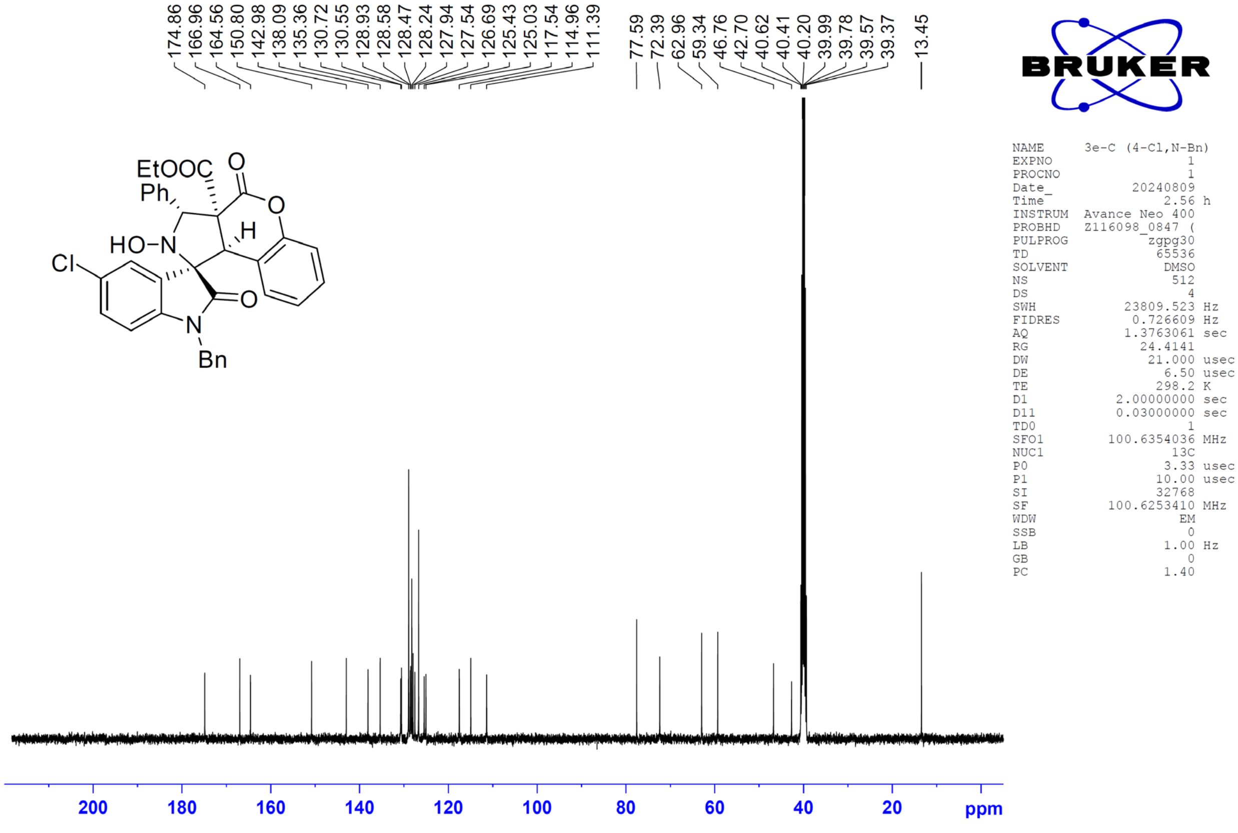

^{1}H NMR and ^{13}C NMR Spectra for Compound **3f**

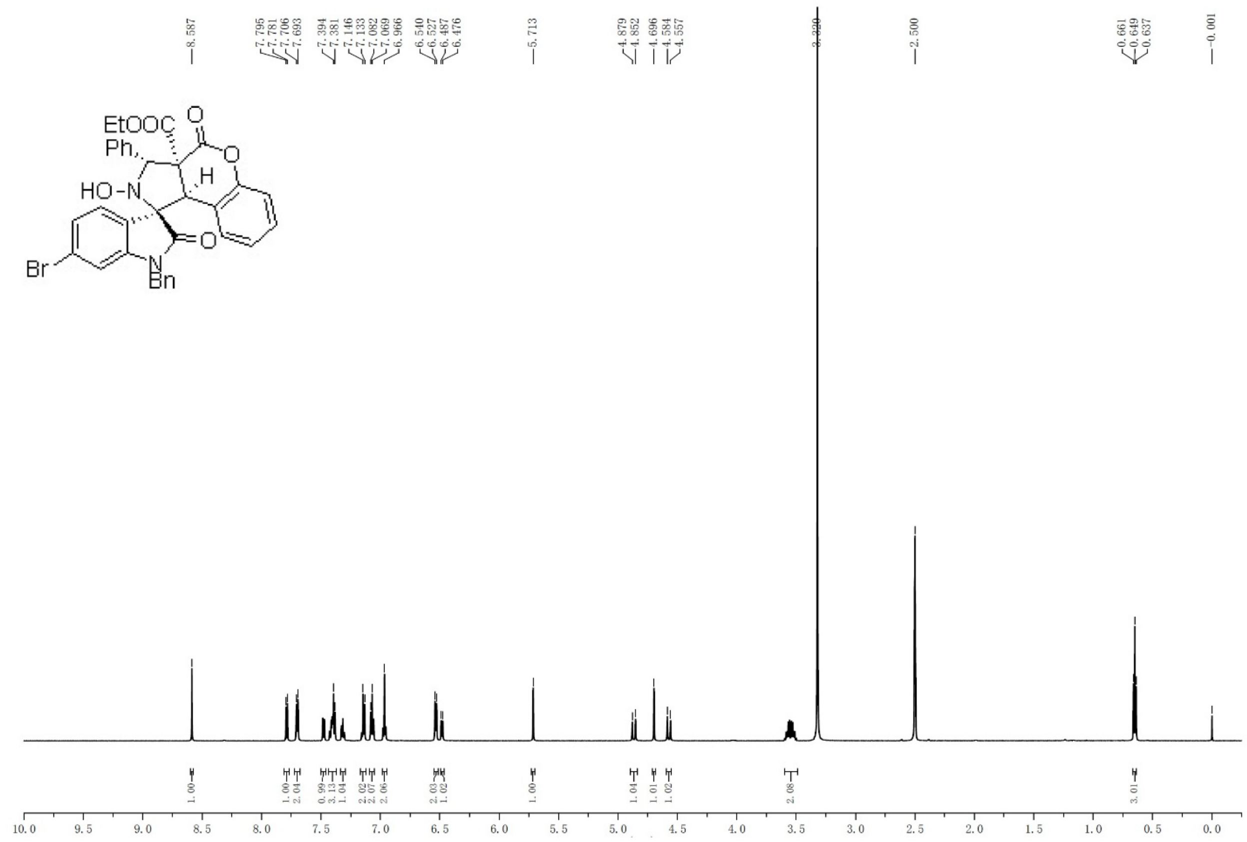

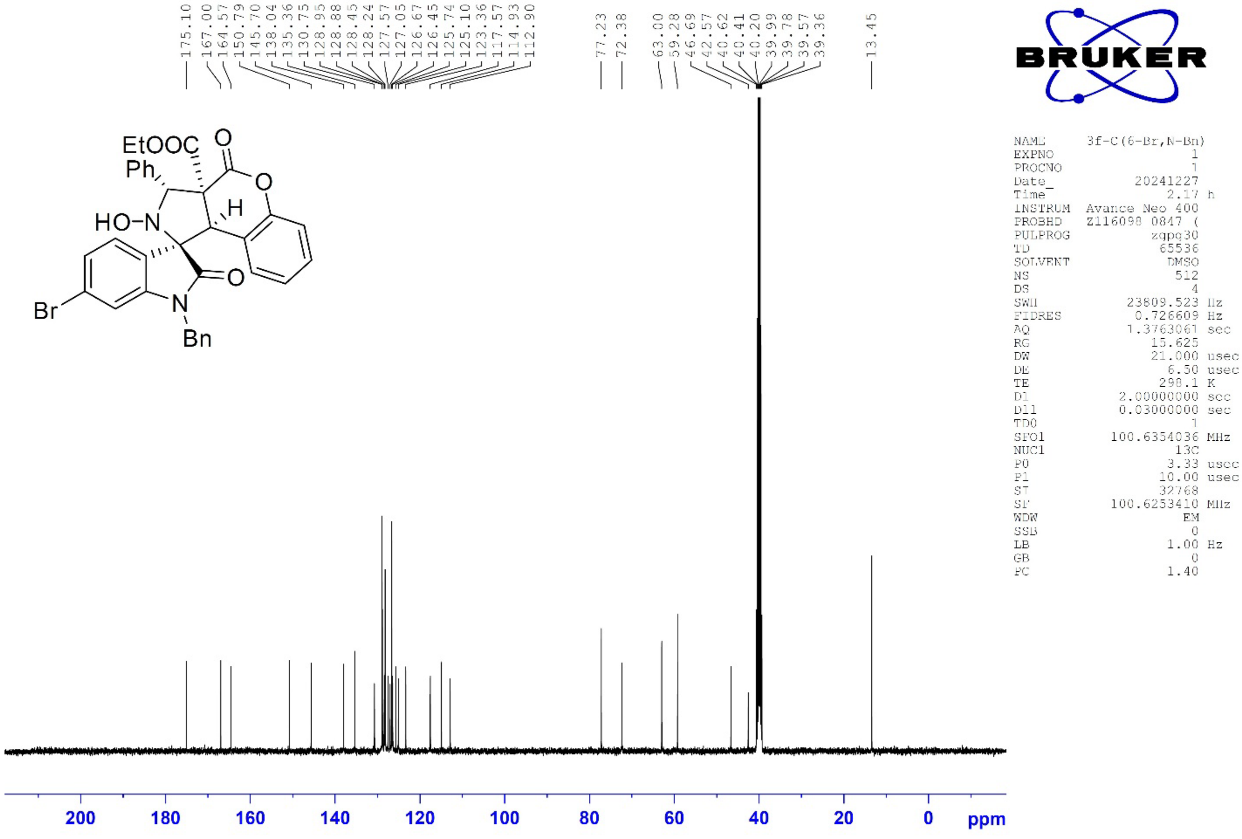

^{1}H NMR and ^{13}C NMR Spectra for Compound **3g**

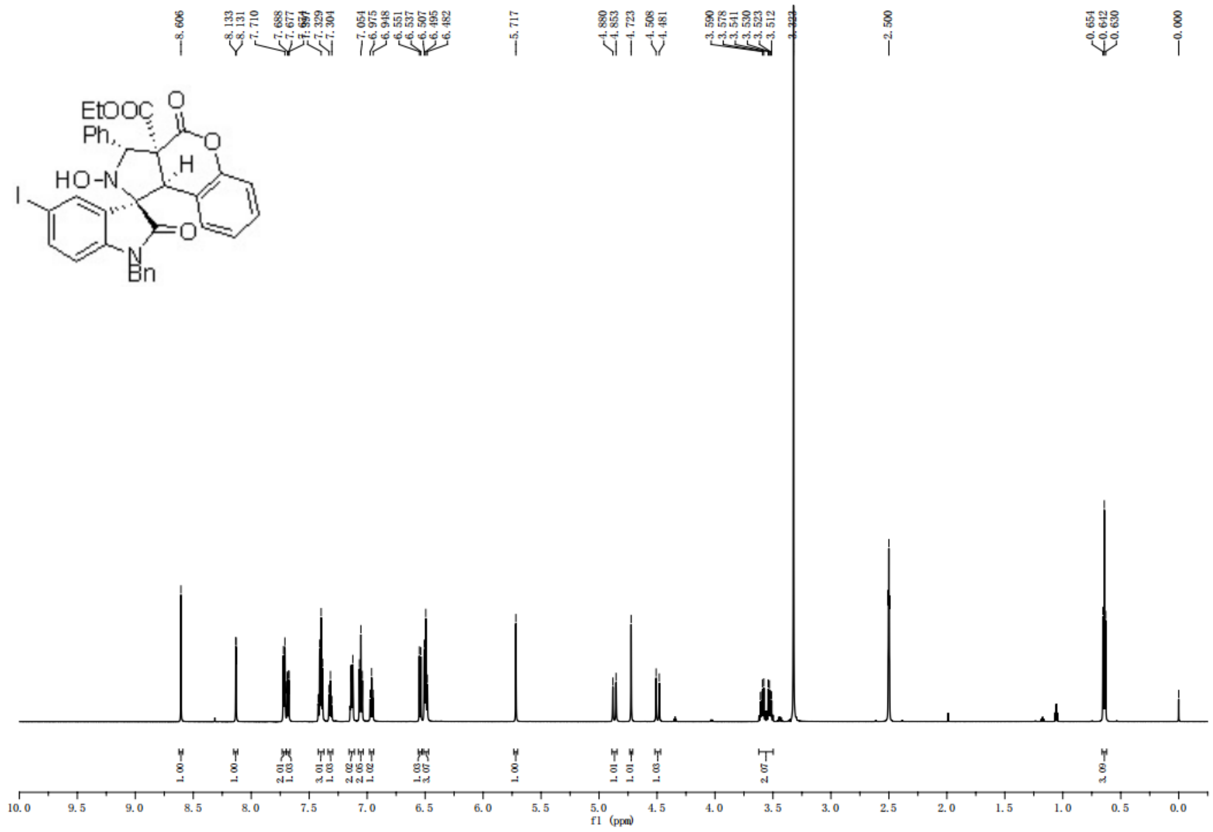

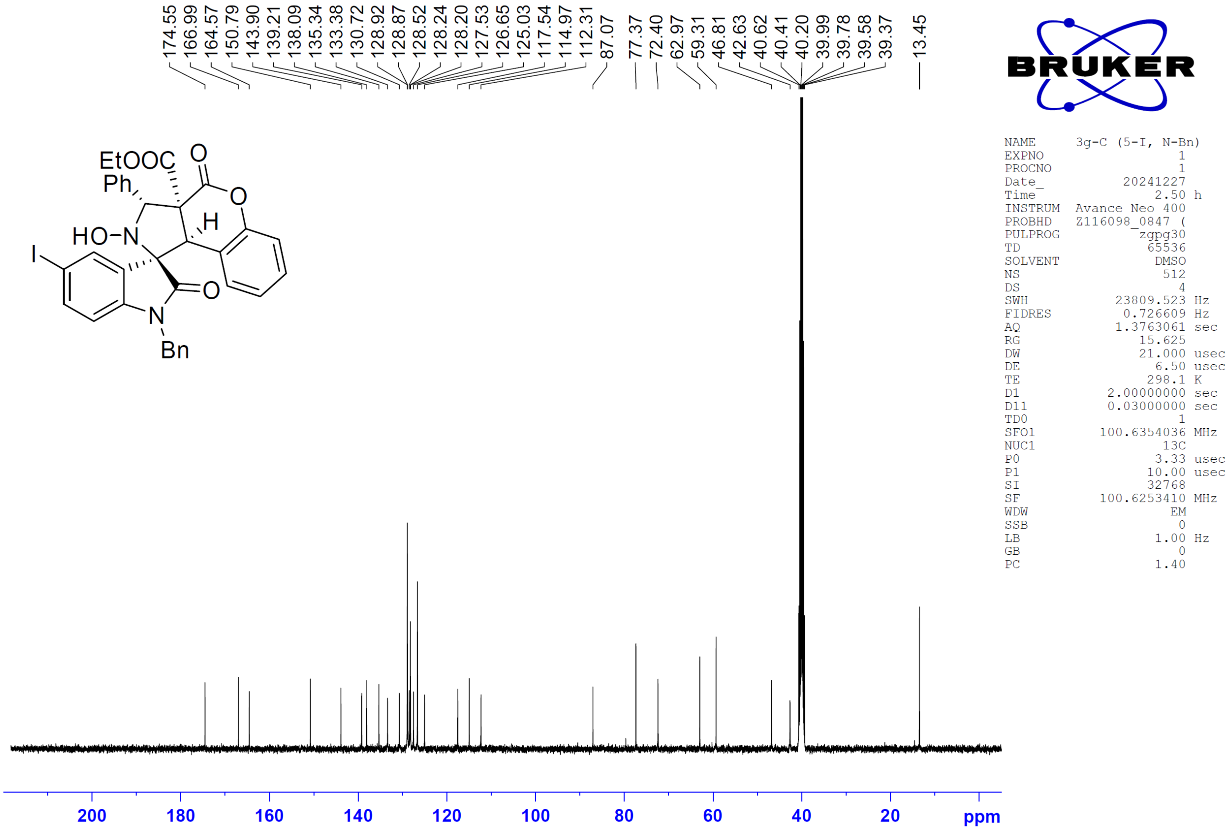

^{1}H NMR and ^{13}C NMR Spectra for Compound **3j**

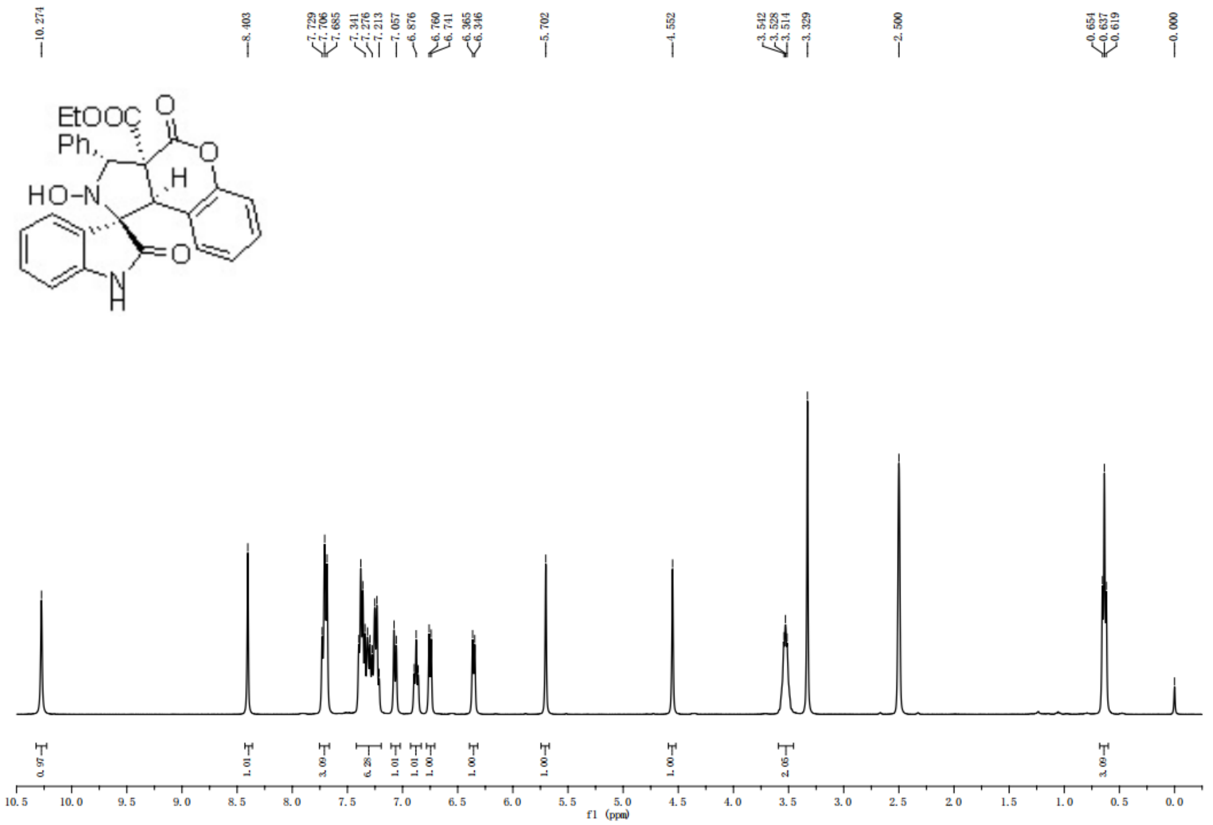

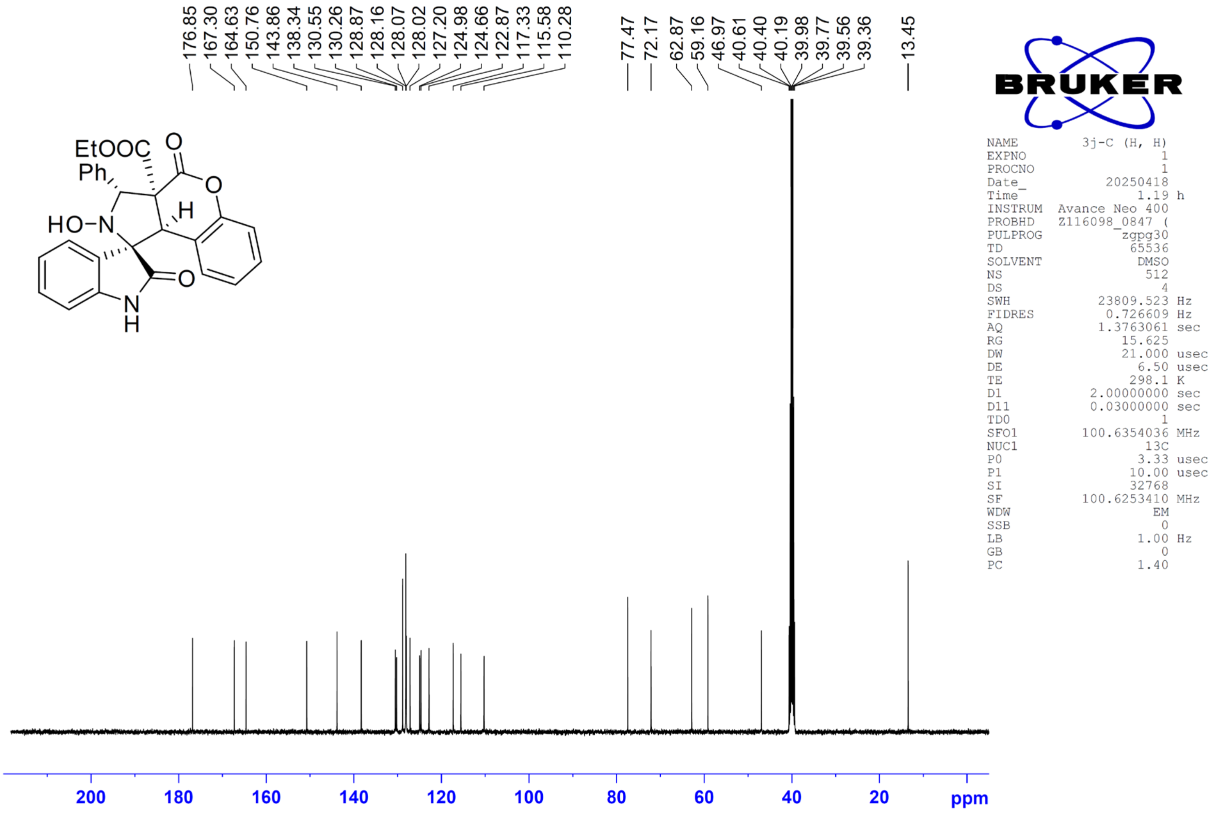

^{1}H NMR and ^{13}C NMR Spectra for Compound **3k**

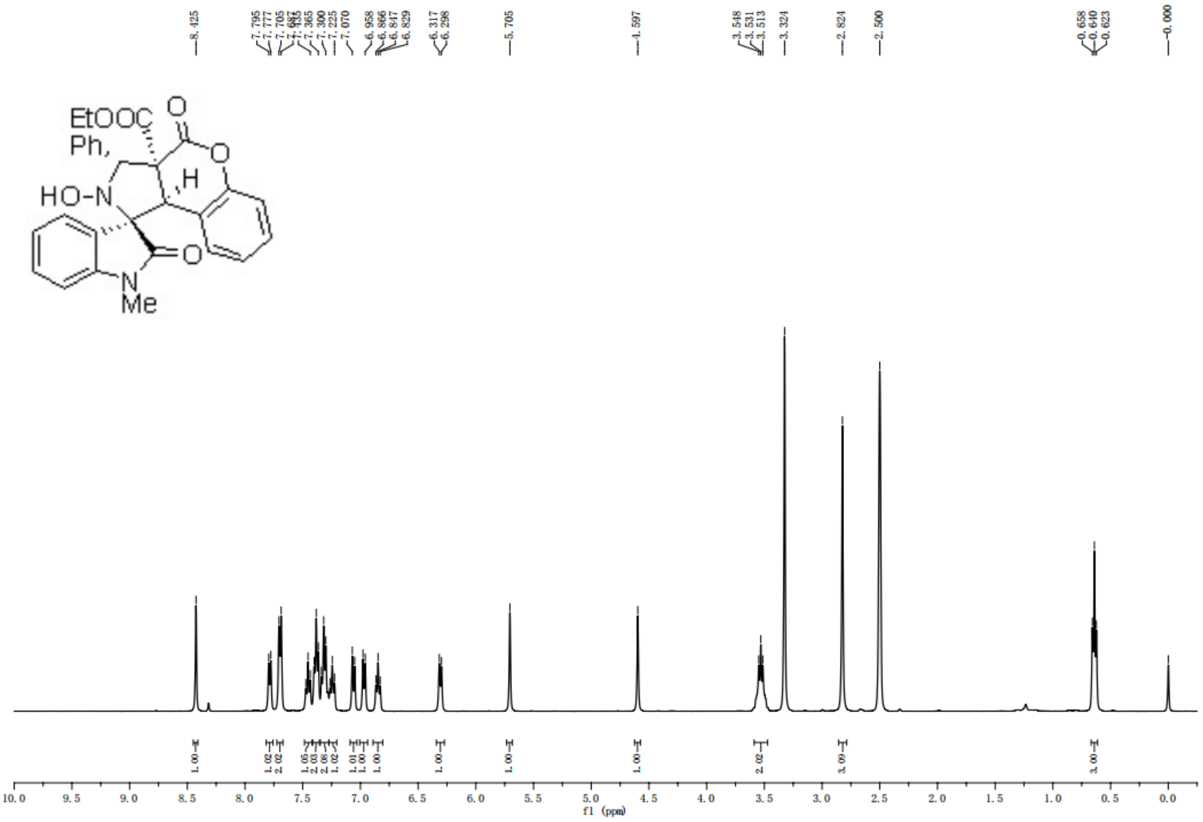

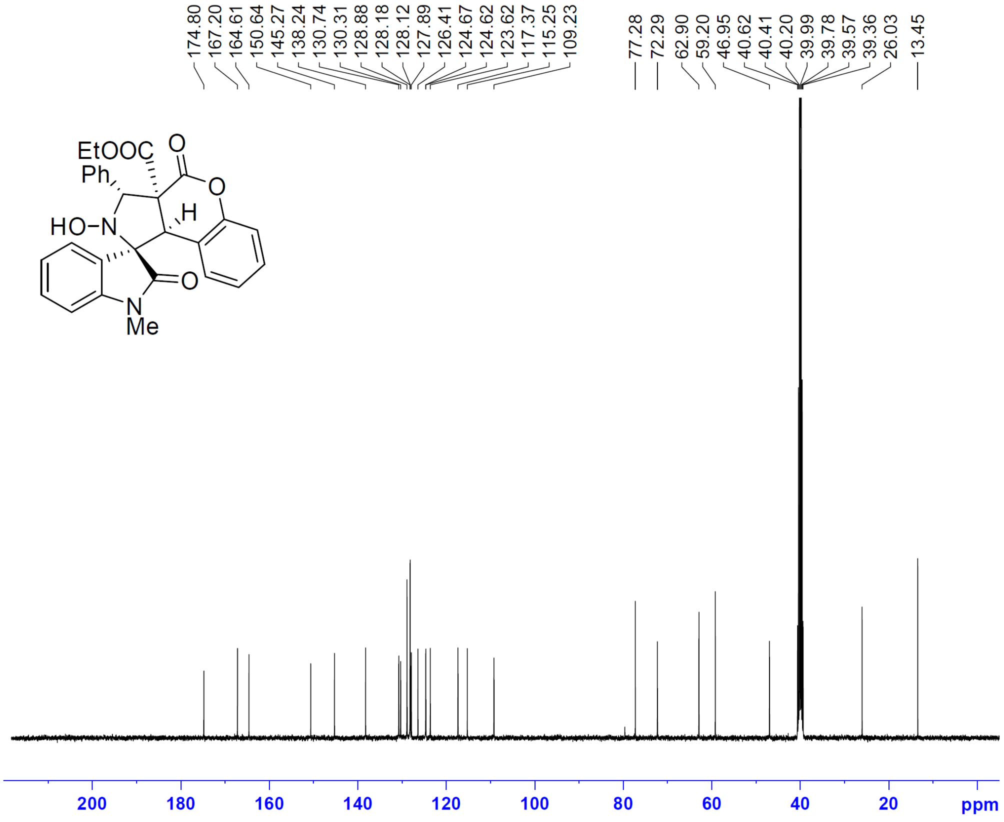

^{1}H NMR and ^{13}C NMR Spectra for Compound **3l**

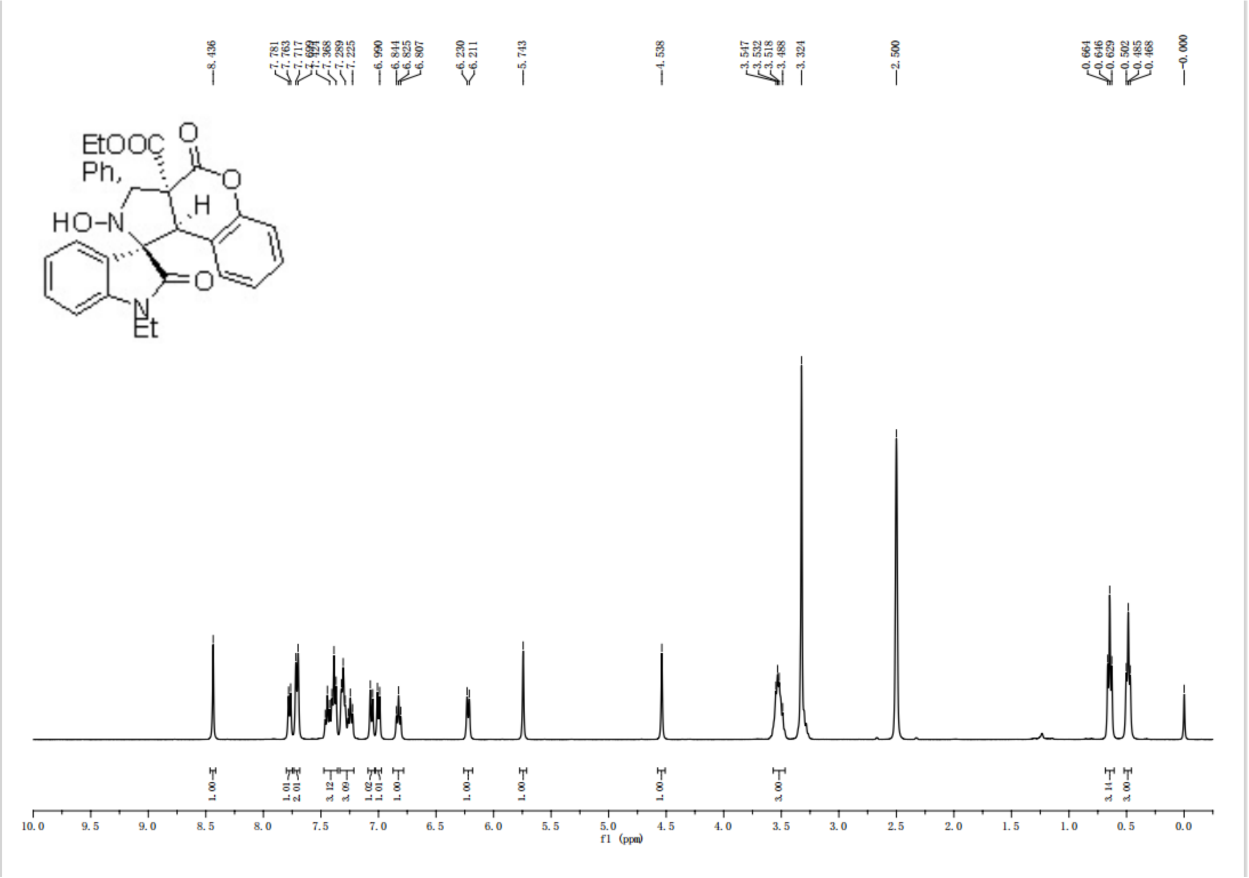

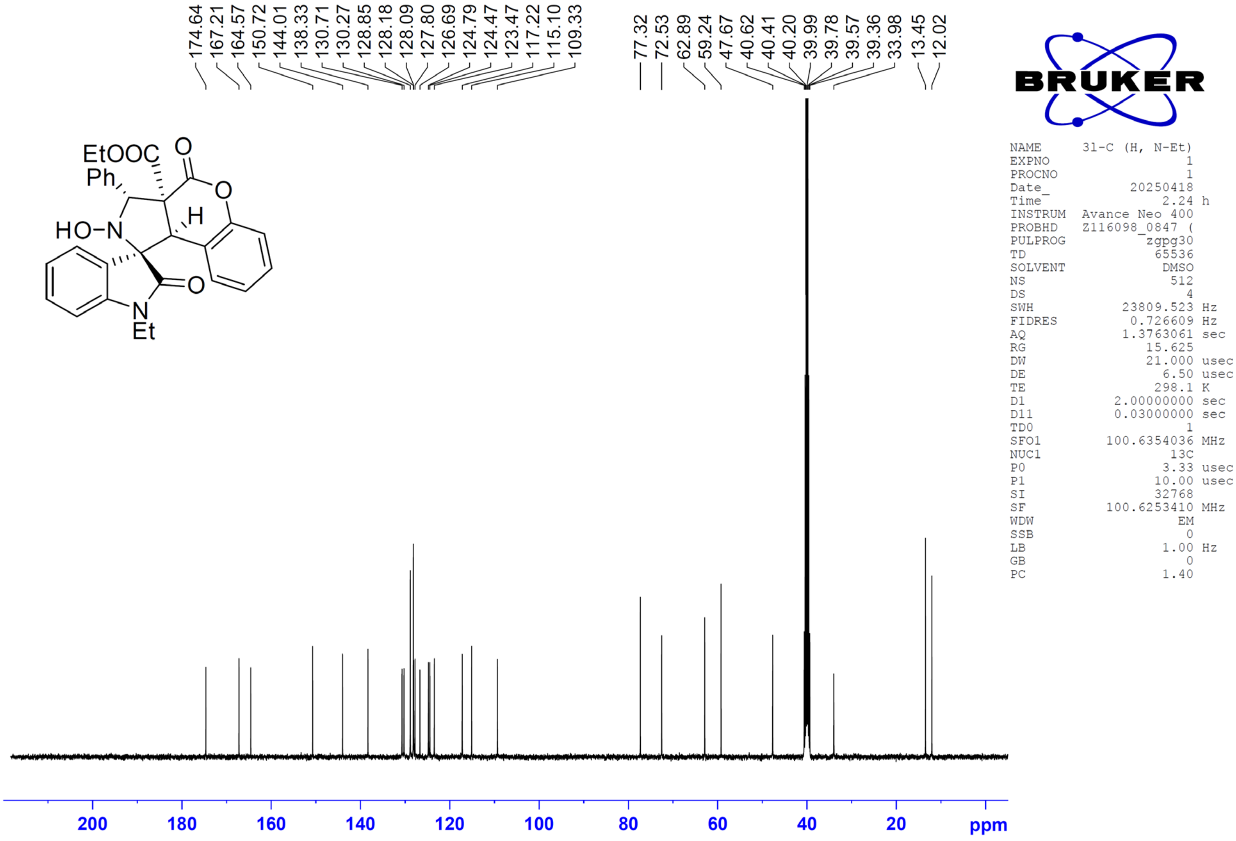

^{1}H NMR and ^{13}C NMR Spectra for Compound **3m**

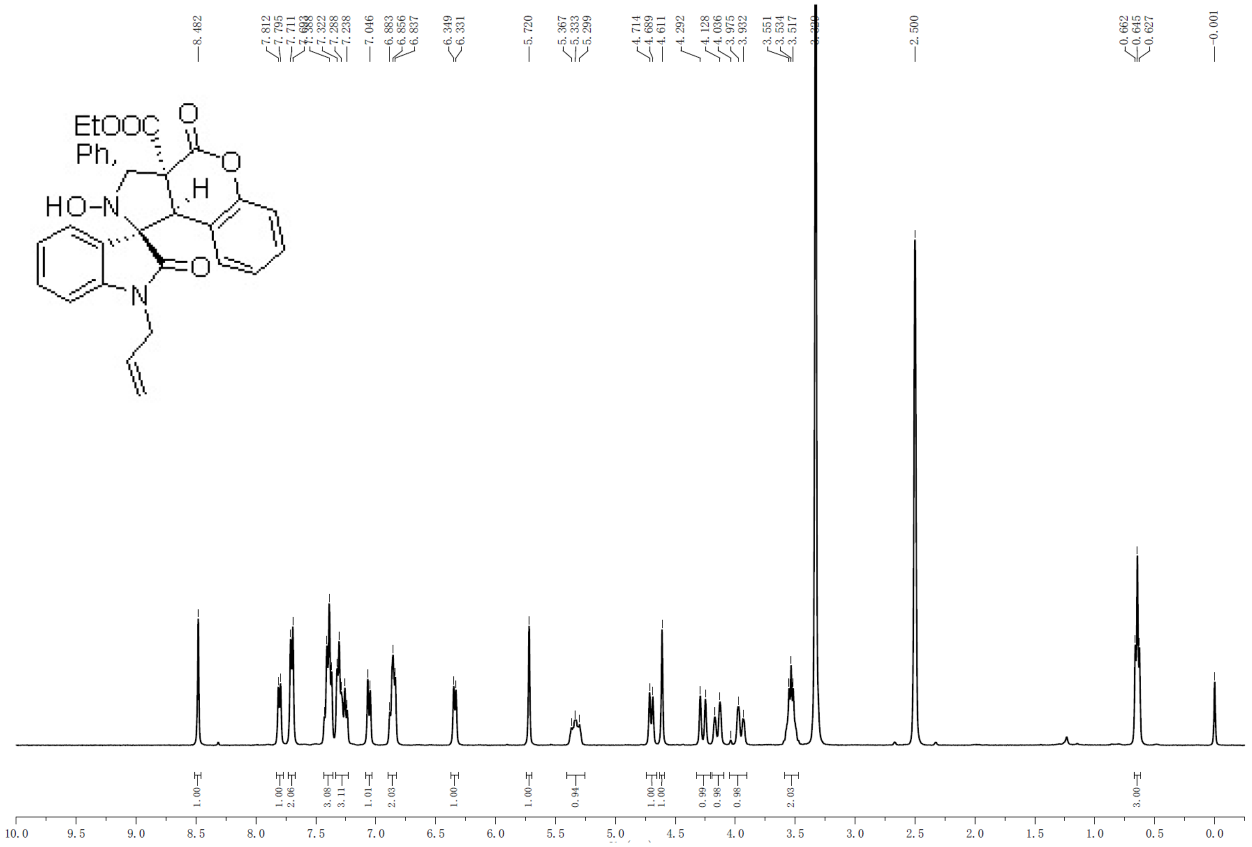

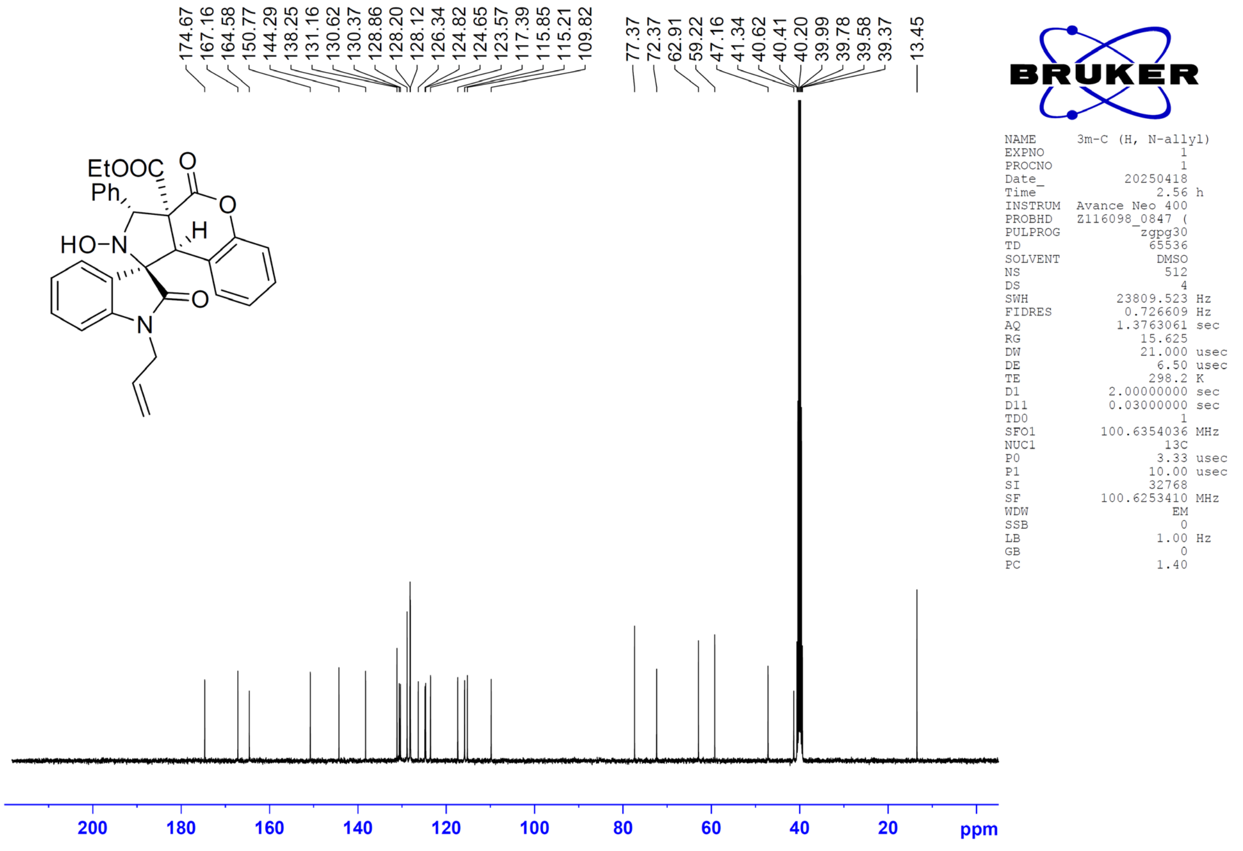

^{1}H NMR and ^{13}C NMR Spectra for Compound **3n**

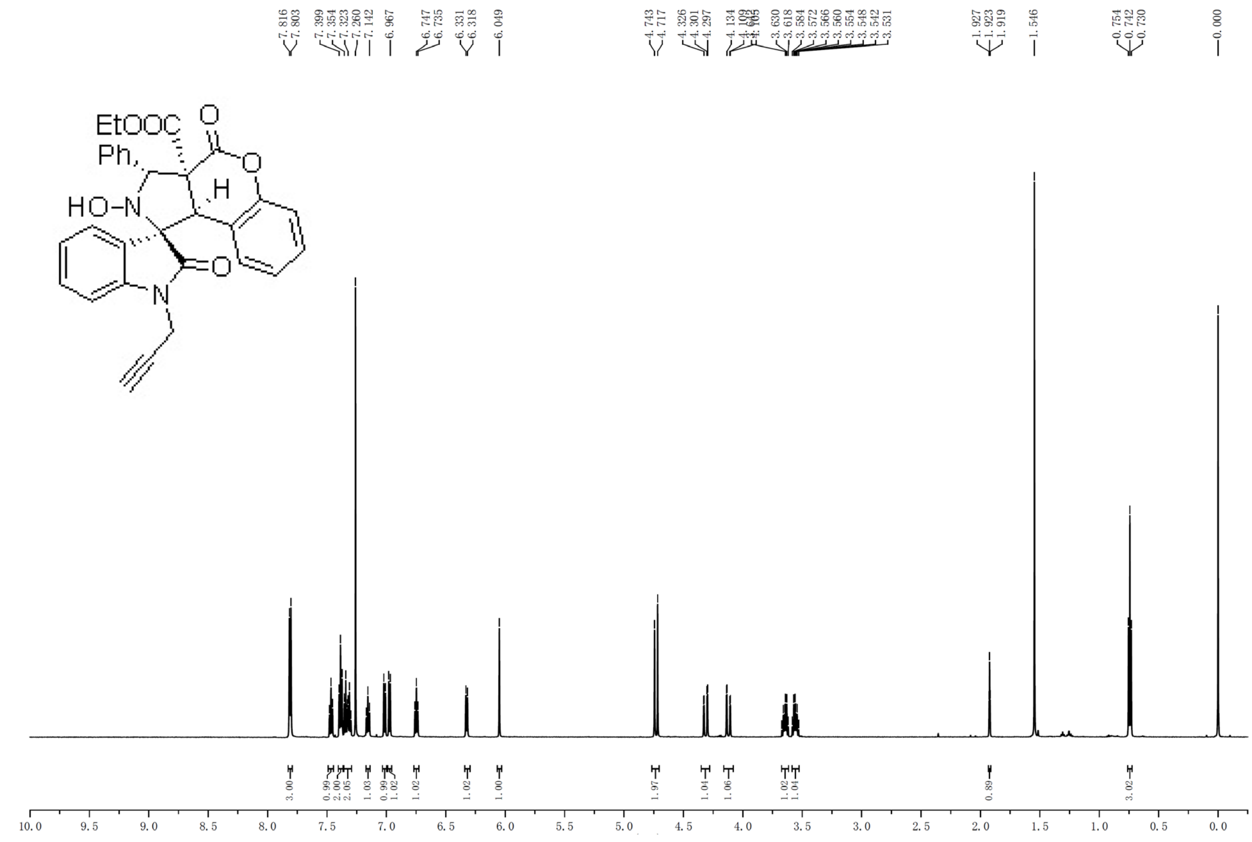

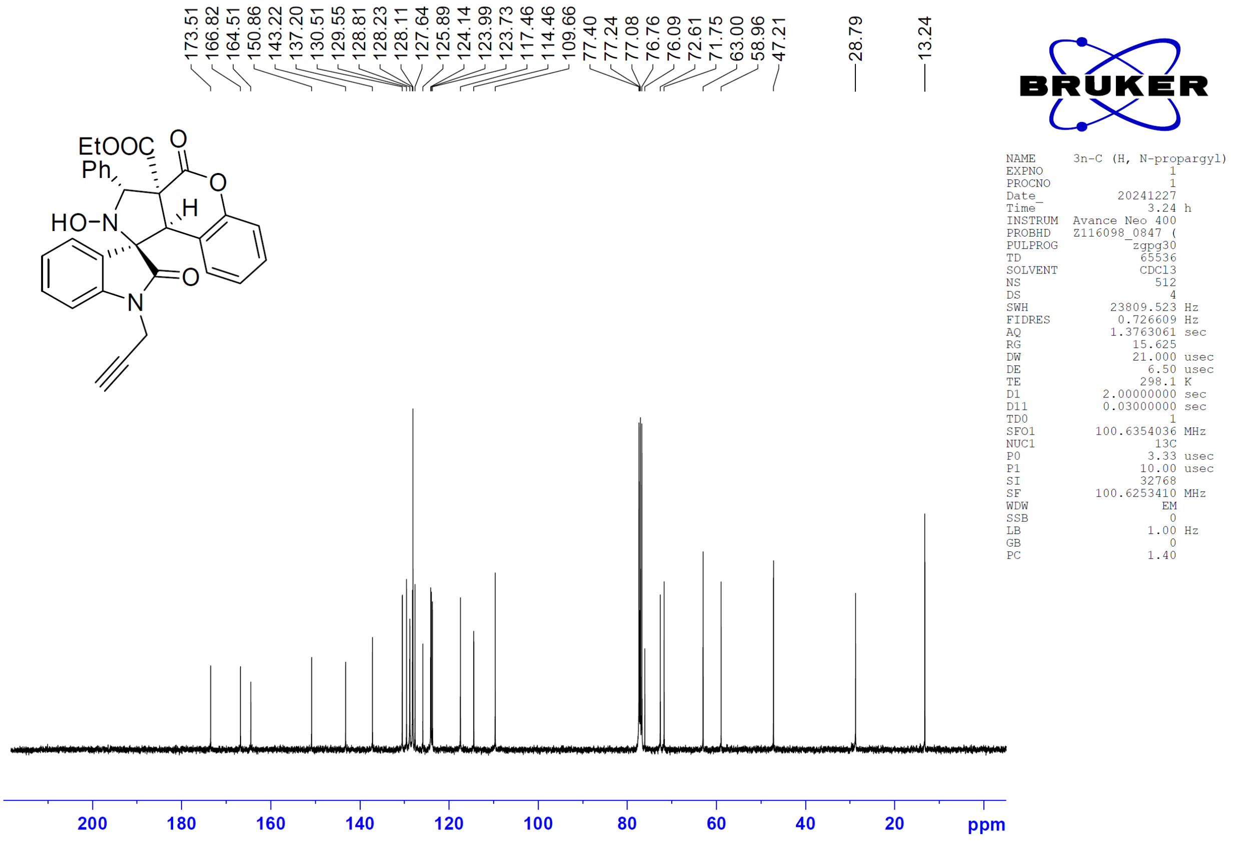

^{1}H NMR and ^{13}C NMR Spectra for Compound **3p**

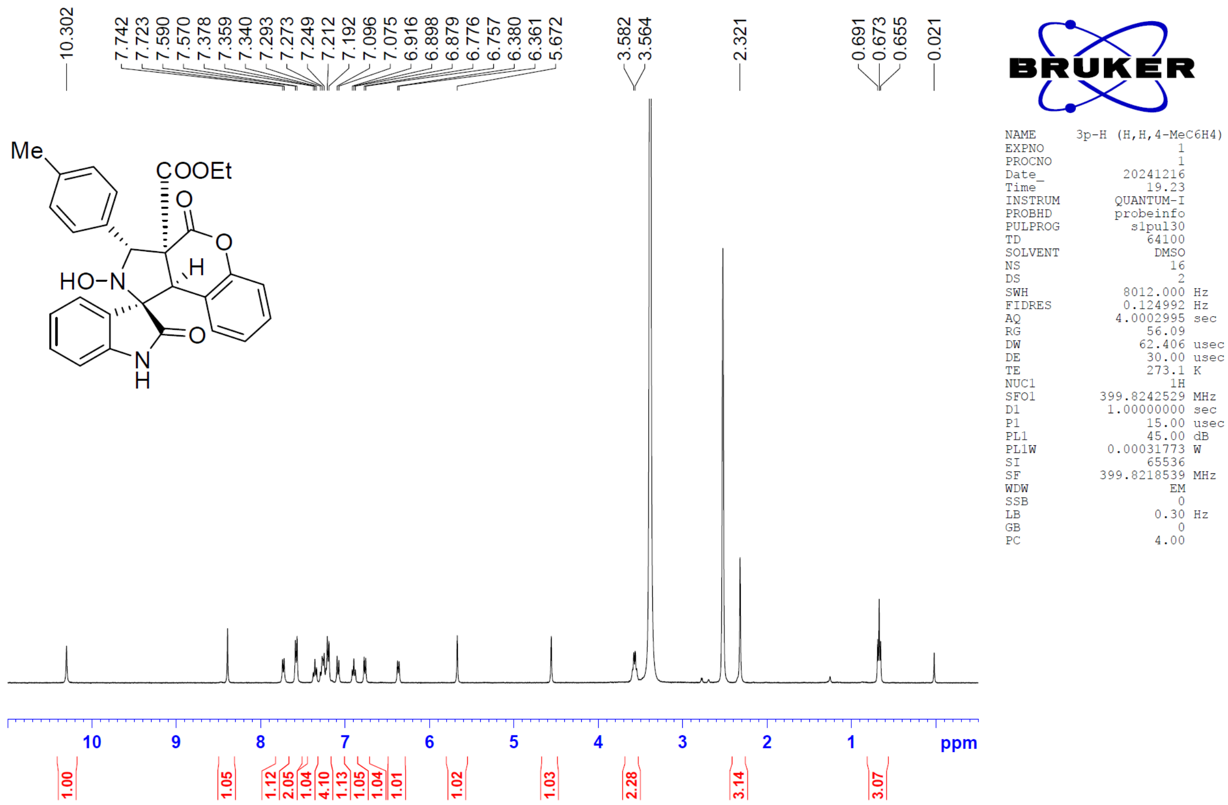

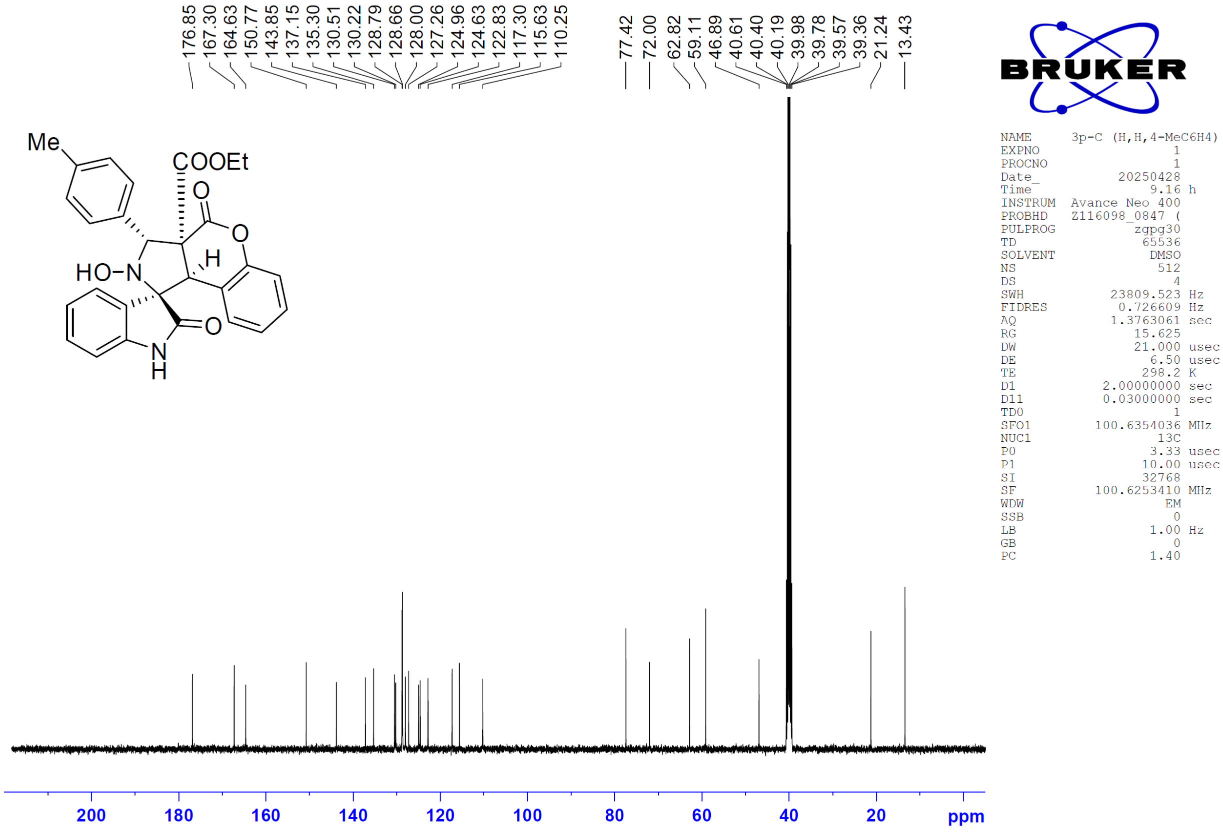

^{1}H NMR and ^{13}C NMR Spectra for Compound **3q**

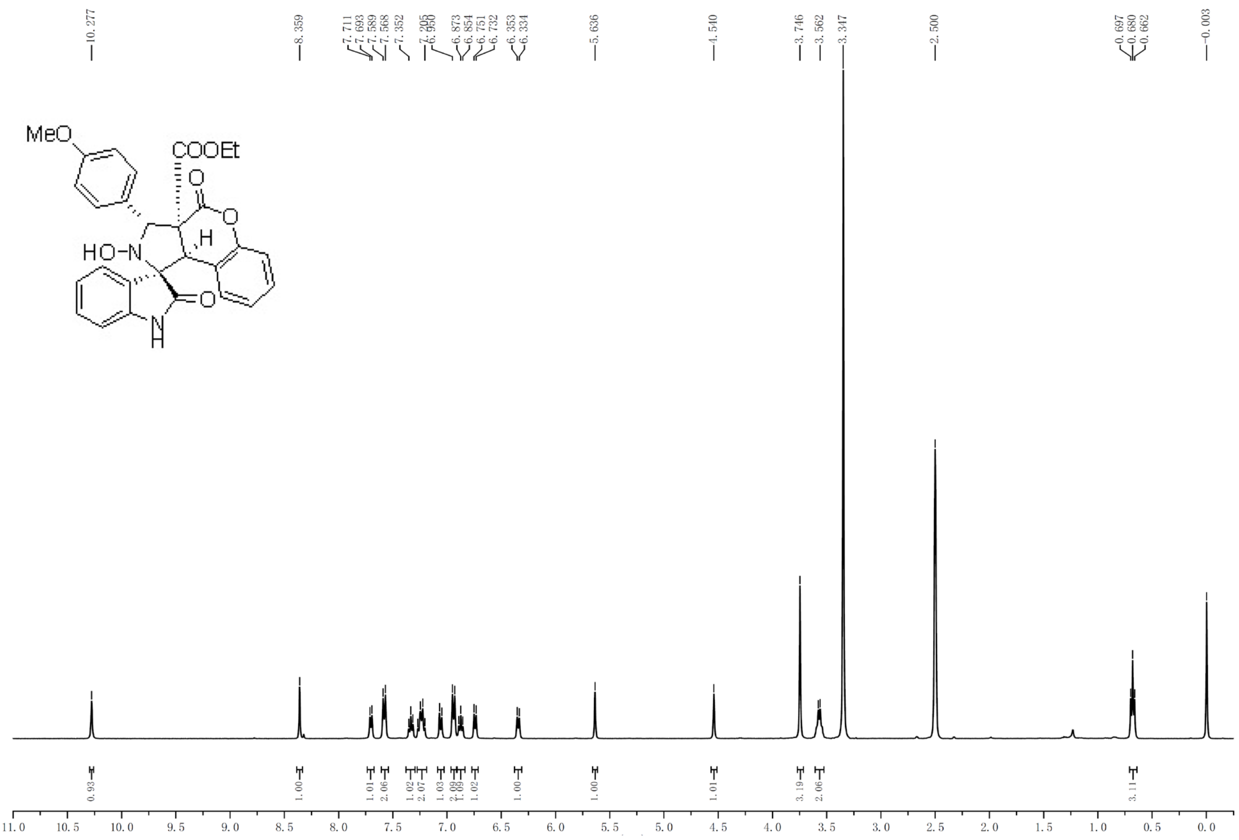

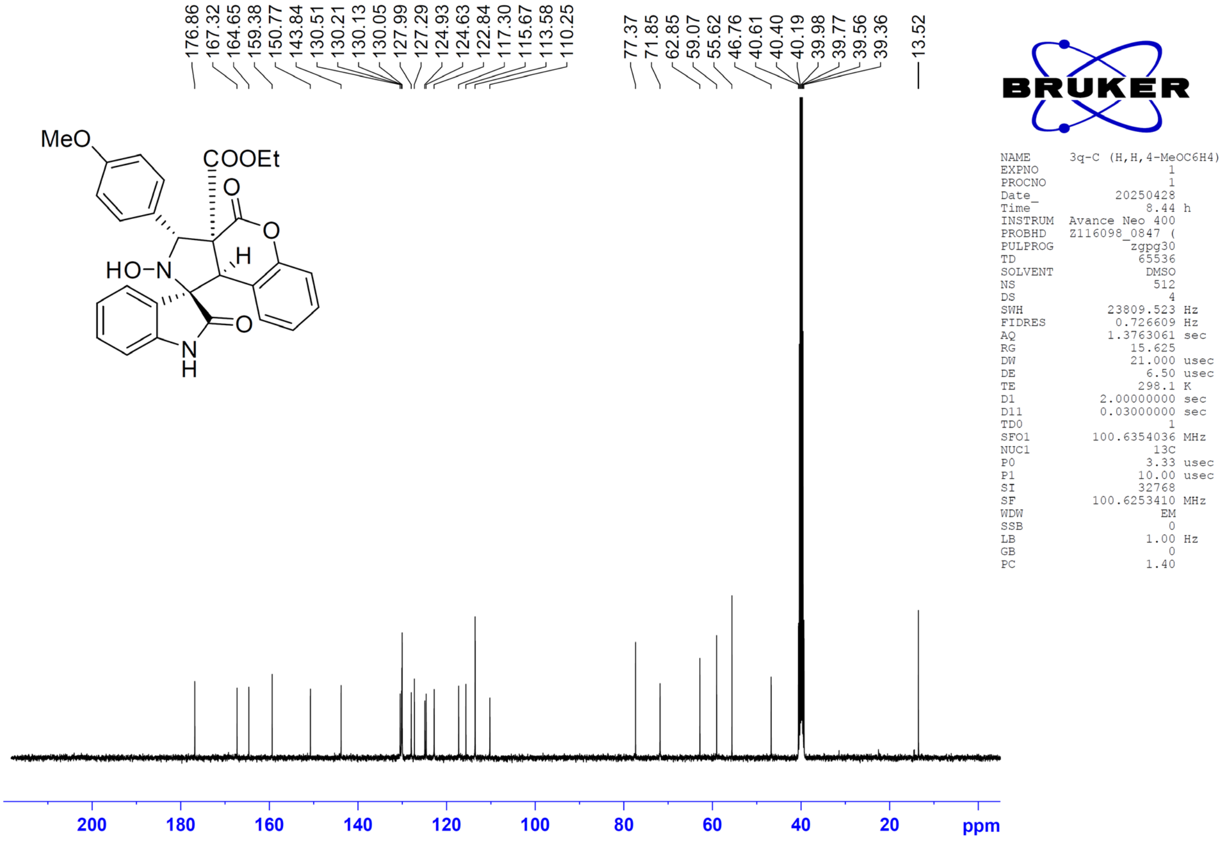

^{1}H NMR, ^{13}C NMR and ^{19}F NMR Spectra for Compound **3r**

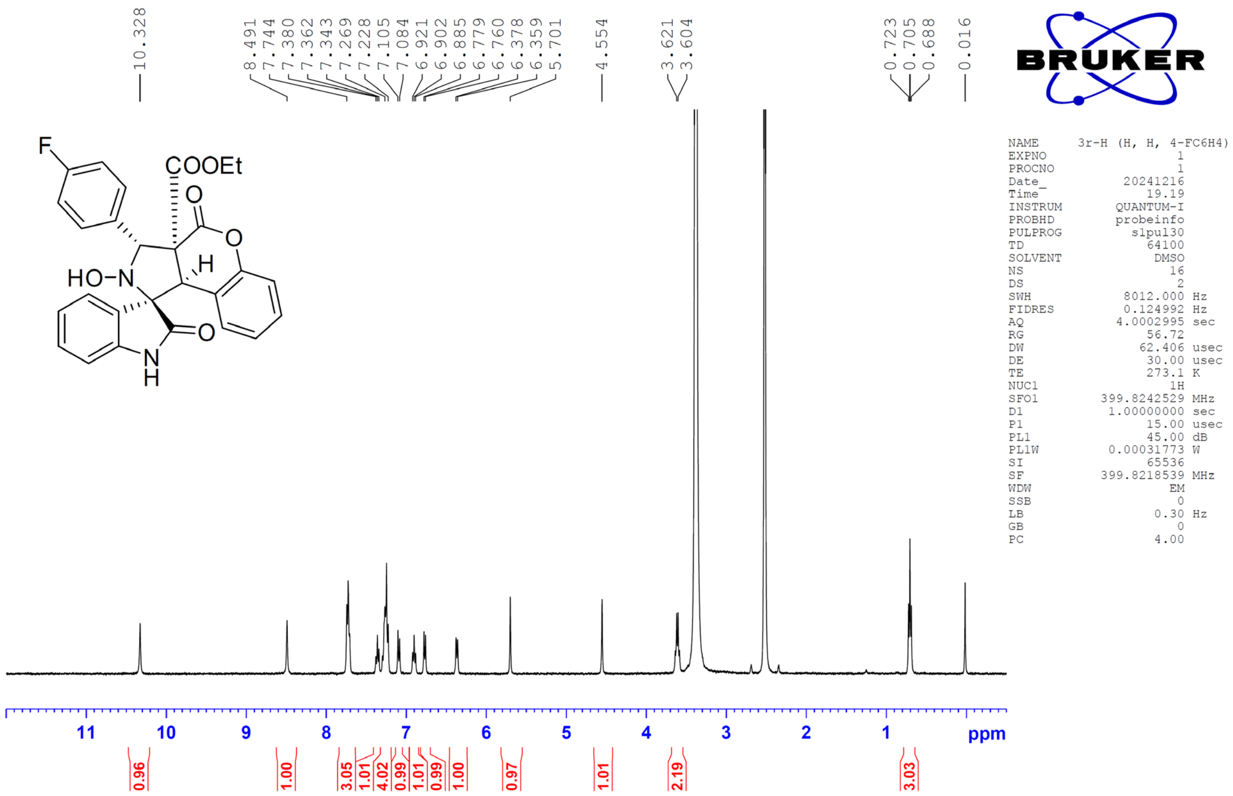

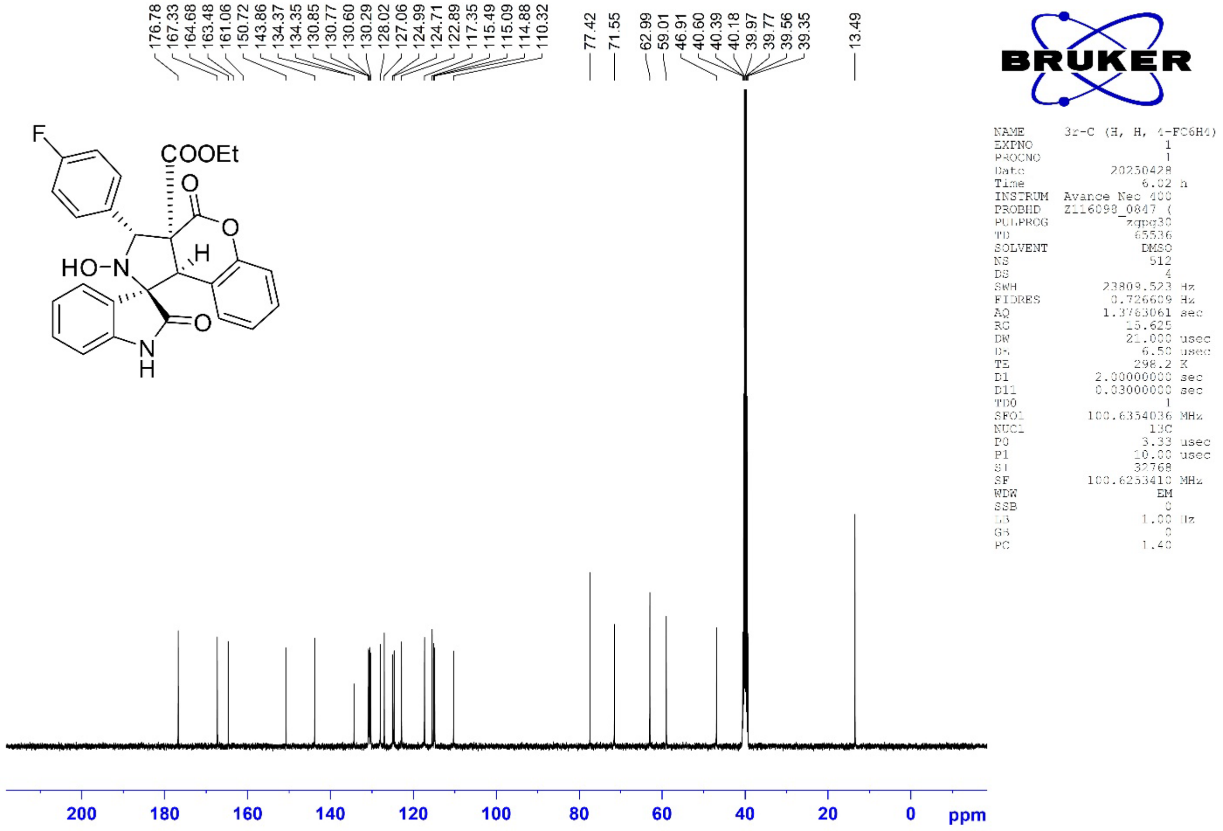

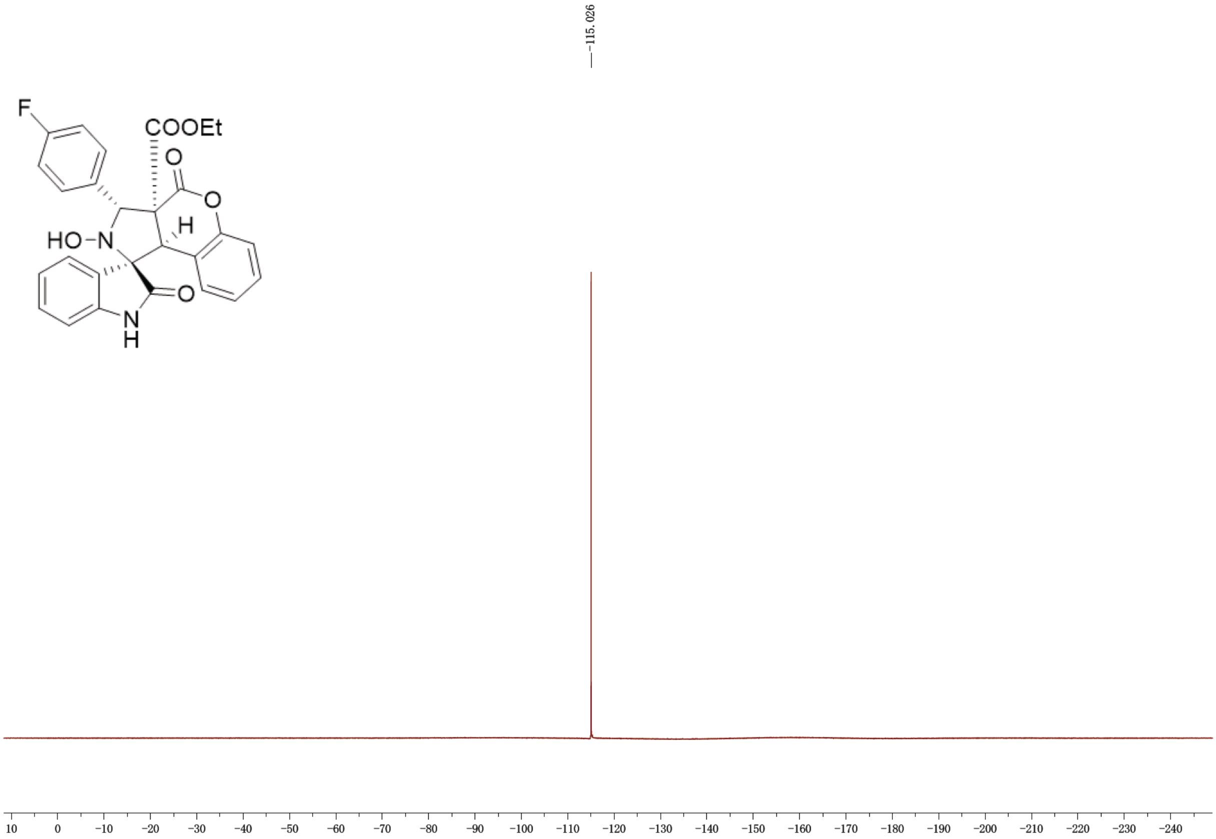
−115.026
F
COOEt
O
O
H
HO
N
O
N
H
10 0 −10 −20 −30 −40 −50 −60 −70 −80 −90 −100 −110 −120 −130 −140 −150 −160 −170 −180 −190 −200 −210 −220 −230 −240

^{1}H NMR and ^{13}C NMR Spectra for Compound **3s**

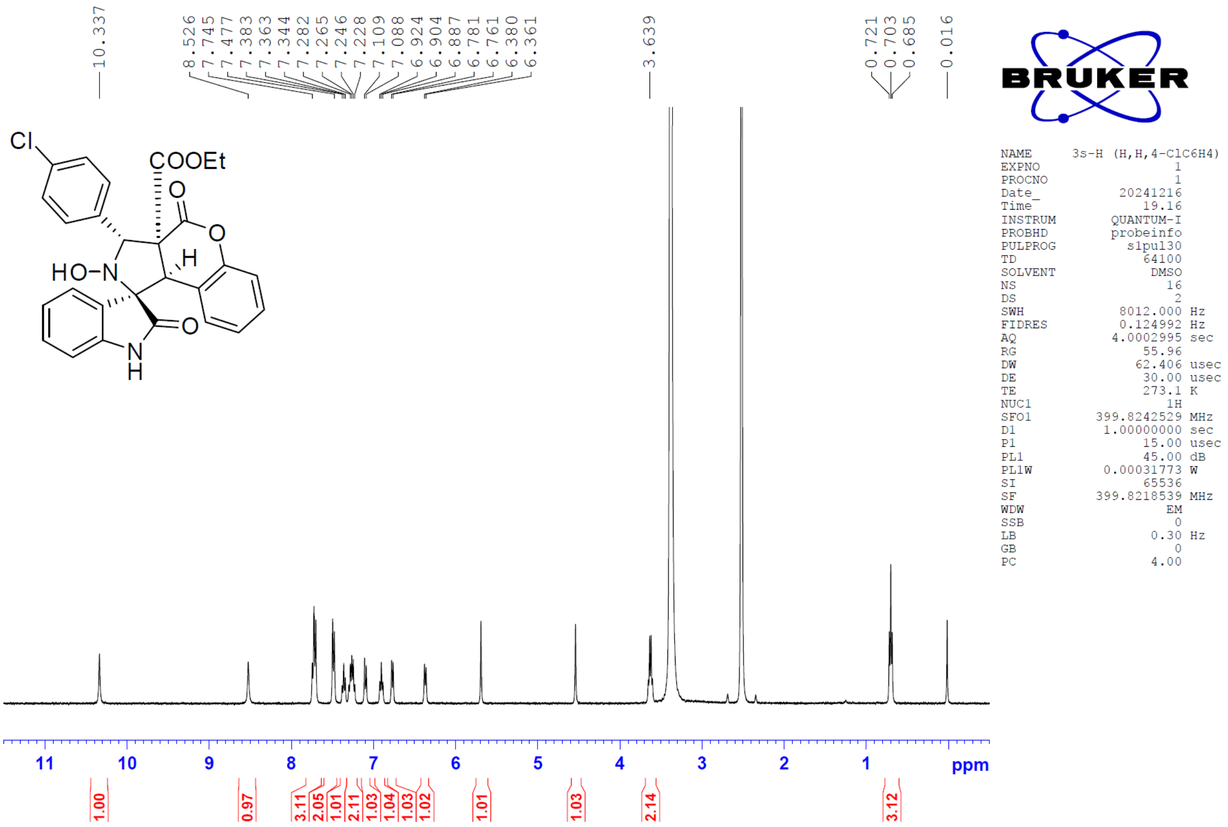

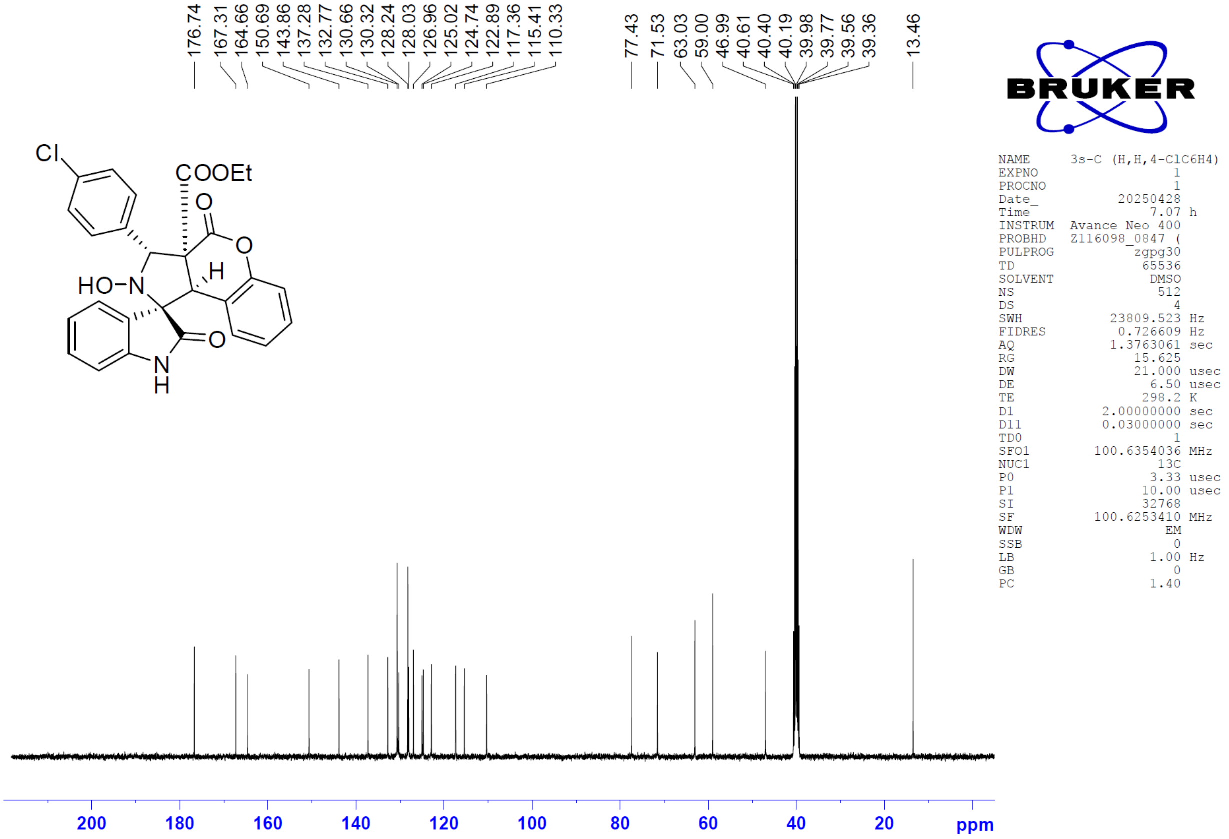

^{1}H NMR and ^{13}C NMR Spectra for Compound **3t**

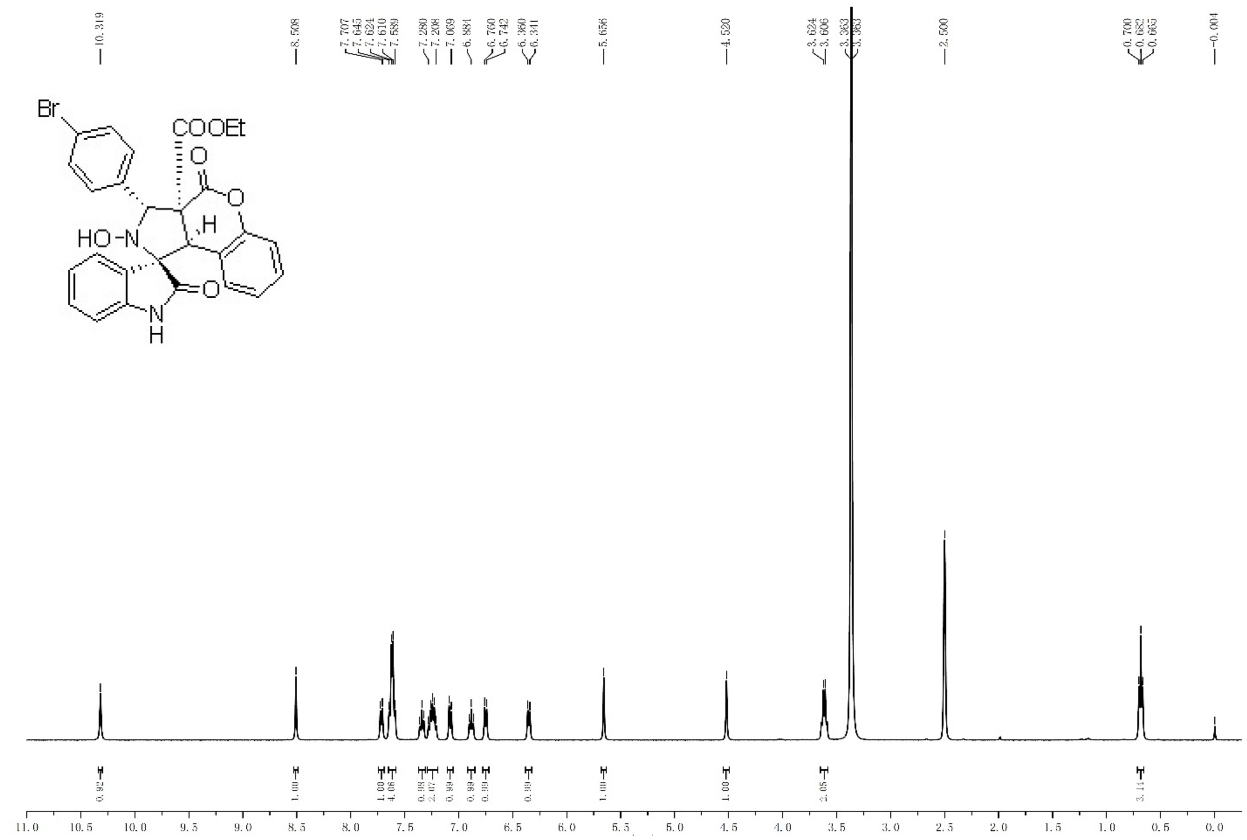

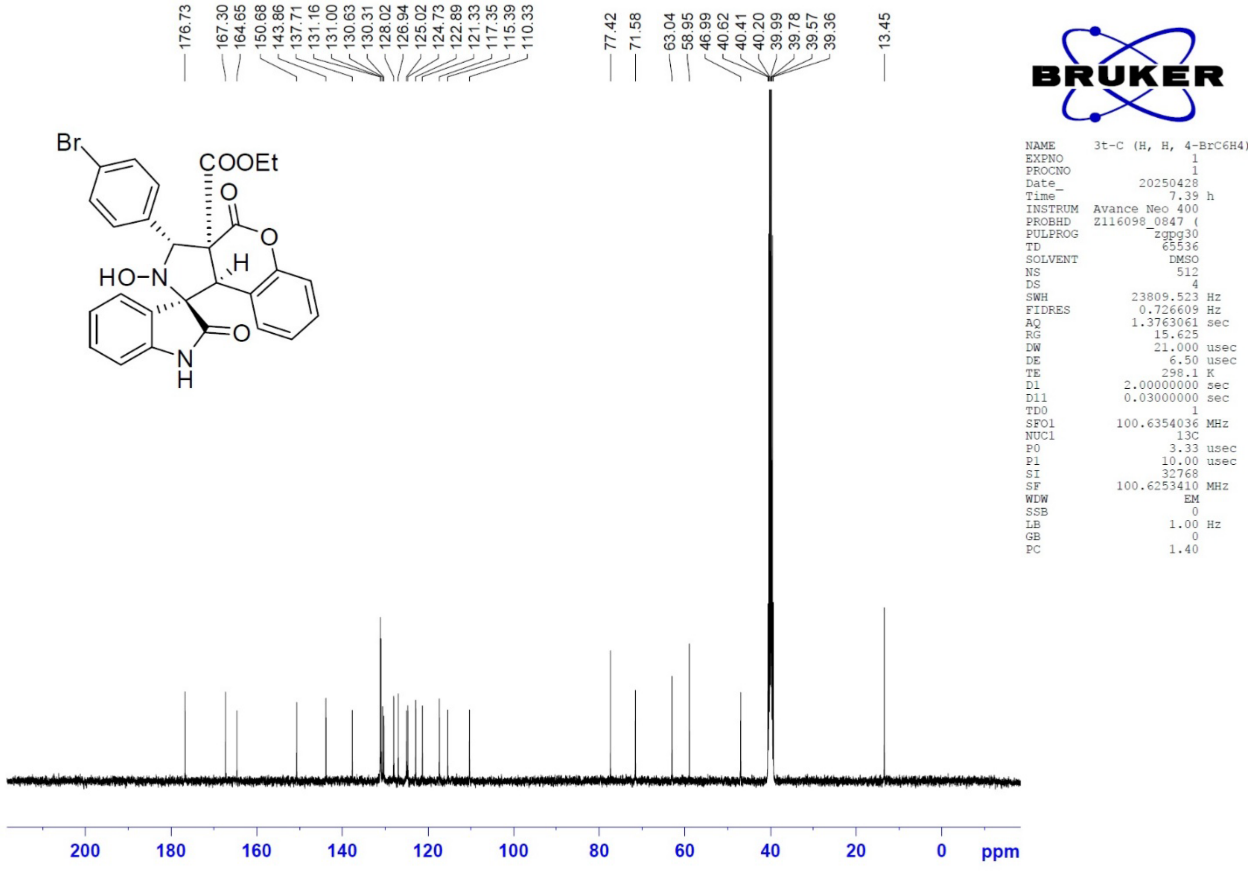

^{1}H NMR and ^{13}C NMR Spectra for Compound **3u**

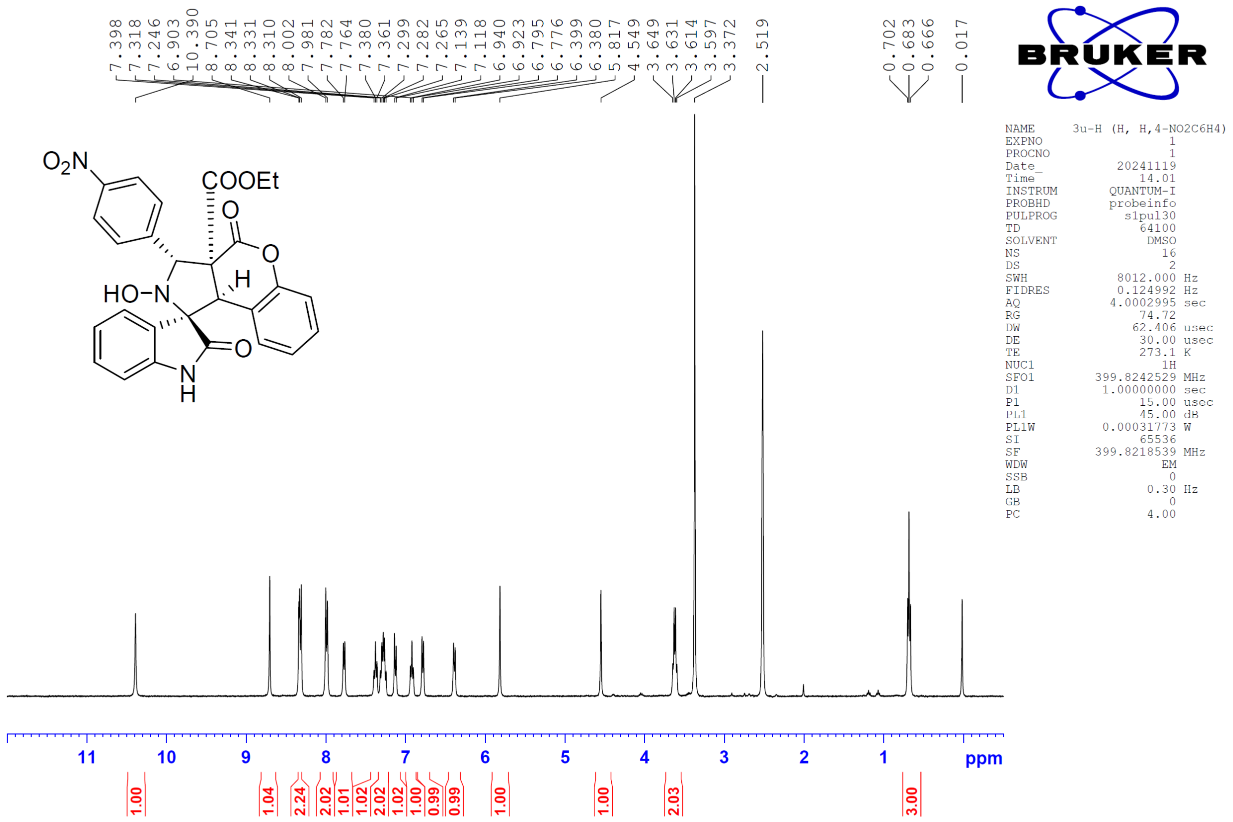

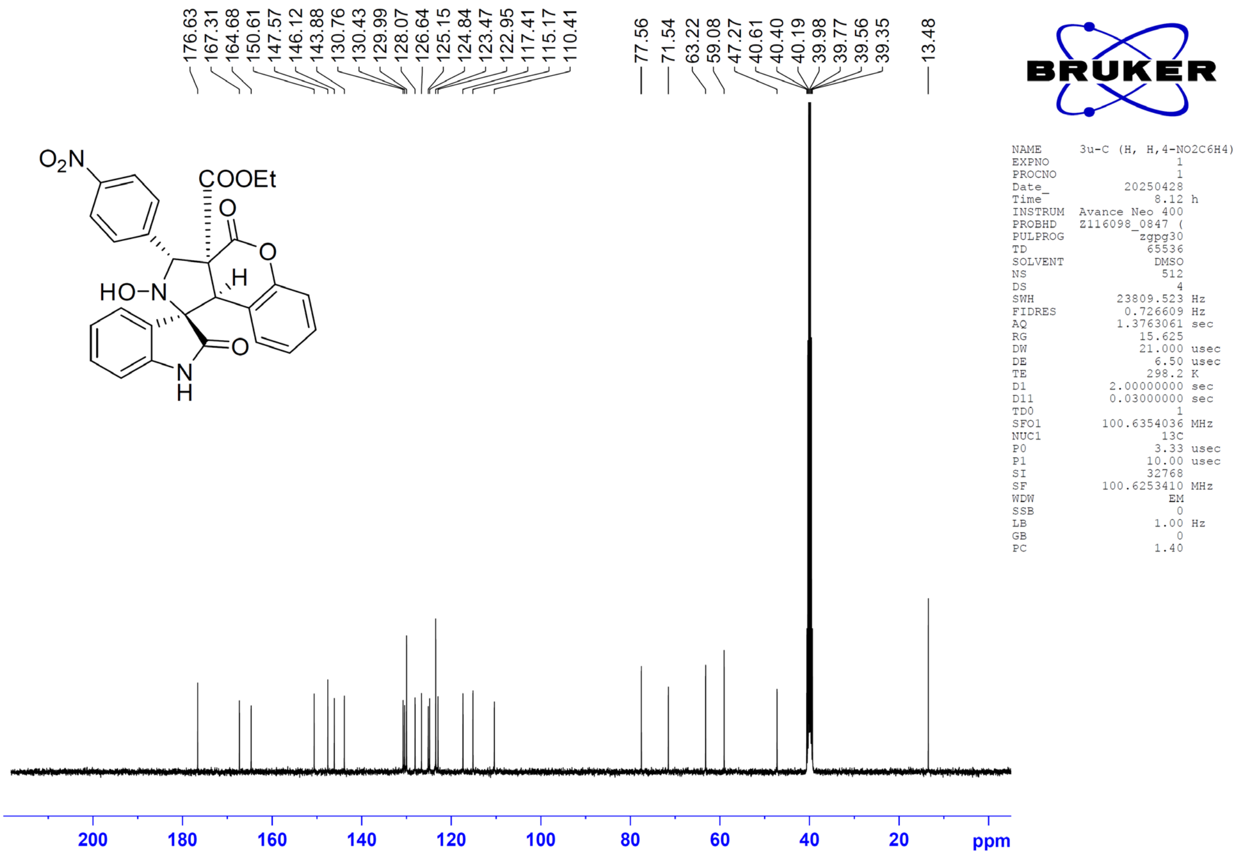

^{1}H NMR and ^{13}C NMR Spectra for Compound **3w**

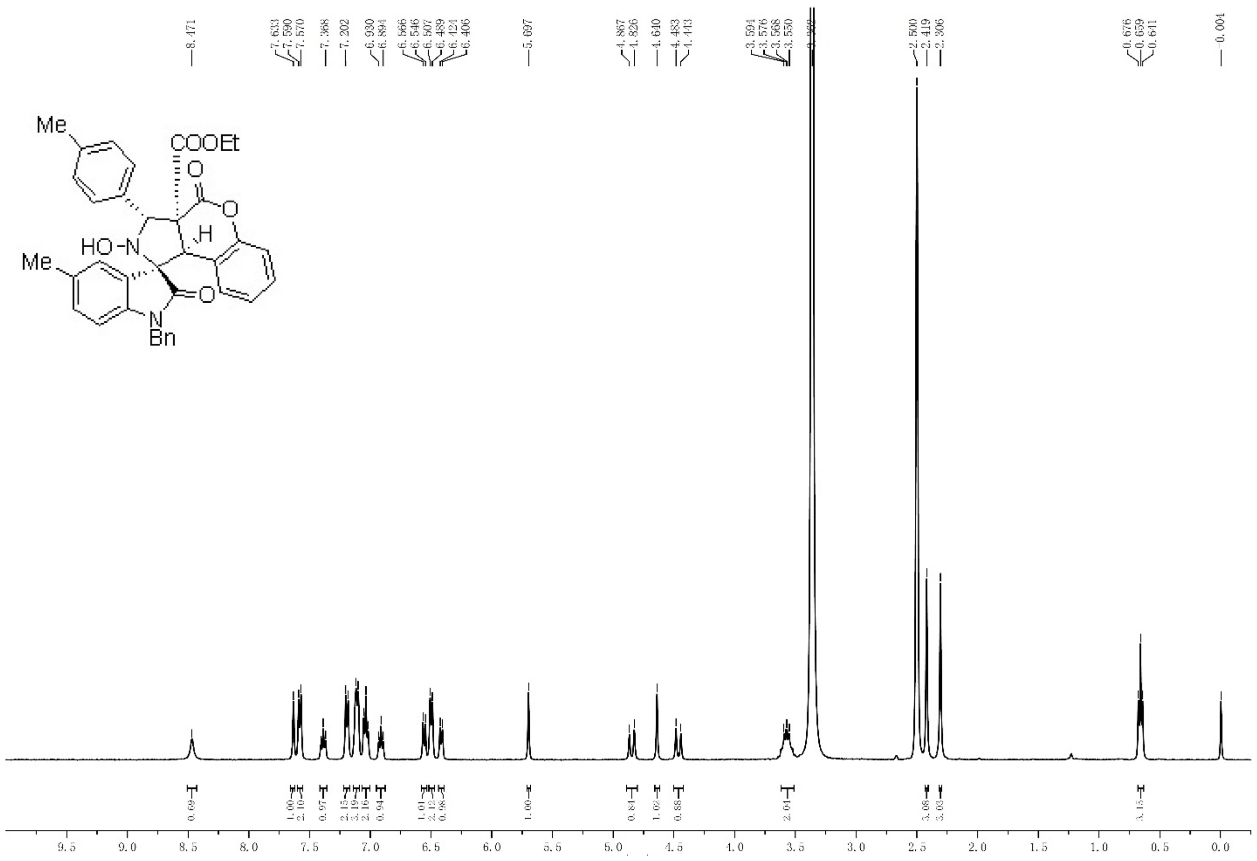

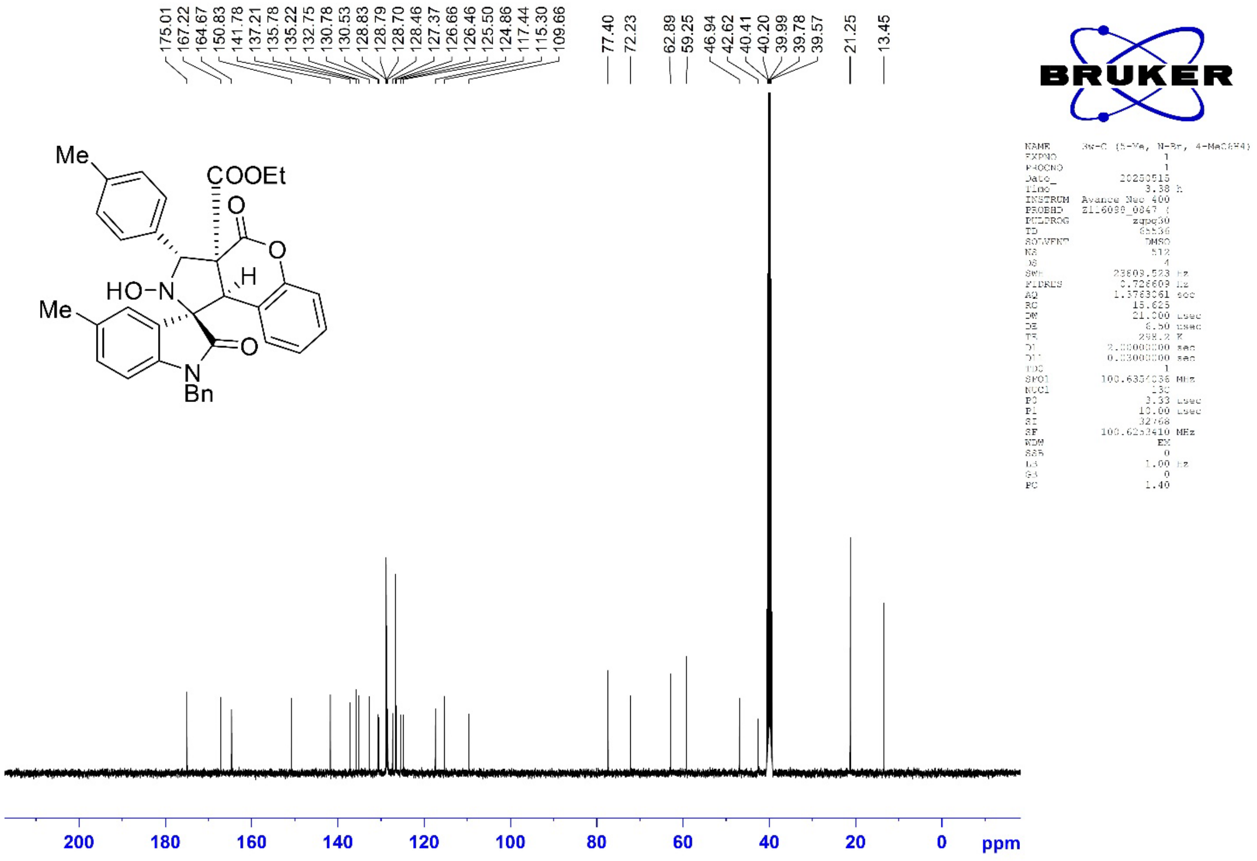

^{1}H NMR and ^{13}C NMR Spectra for Compound **3x**

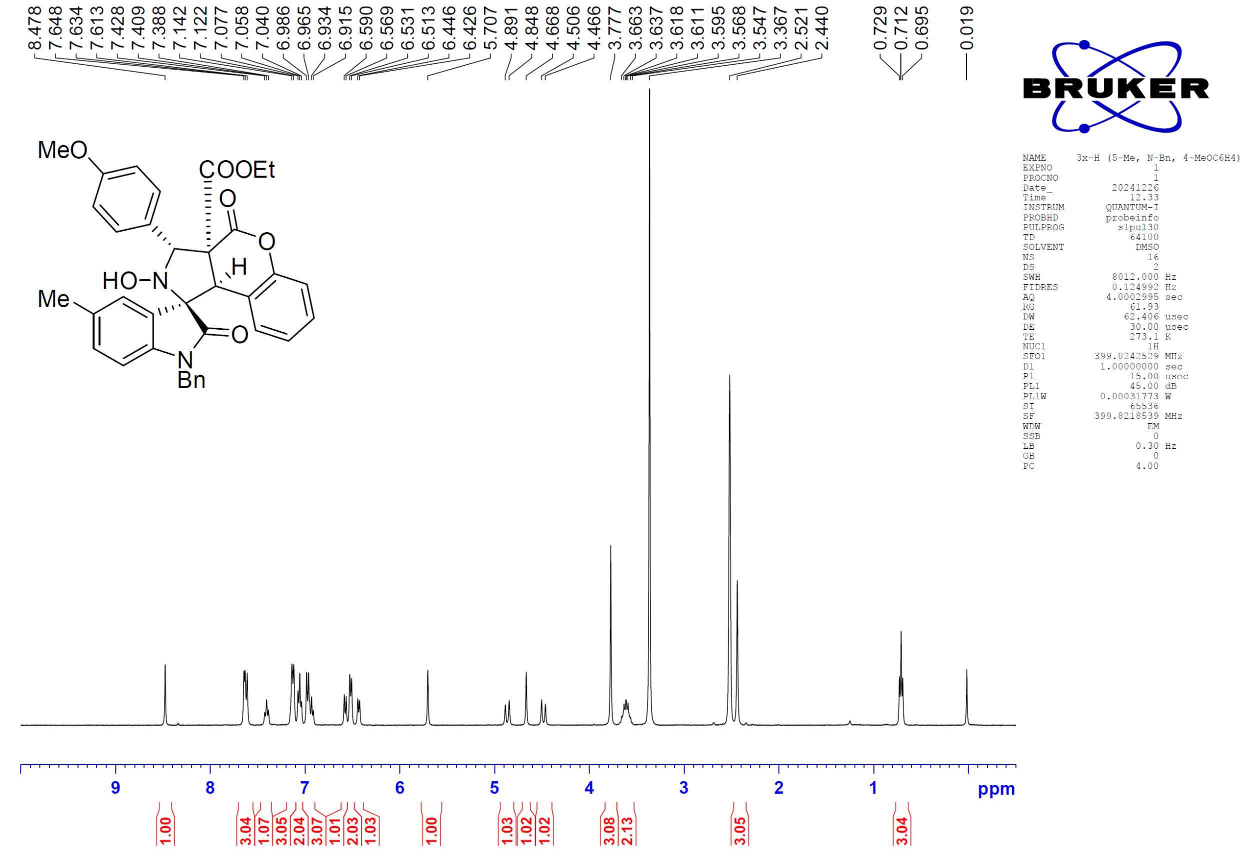

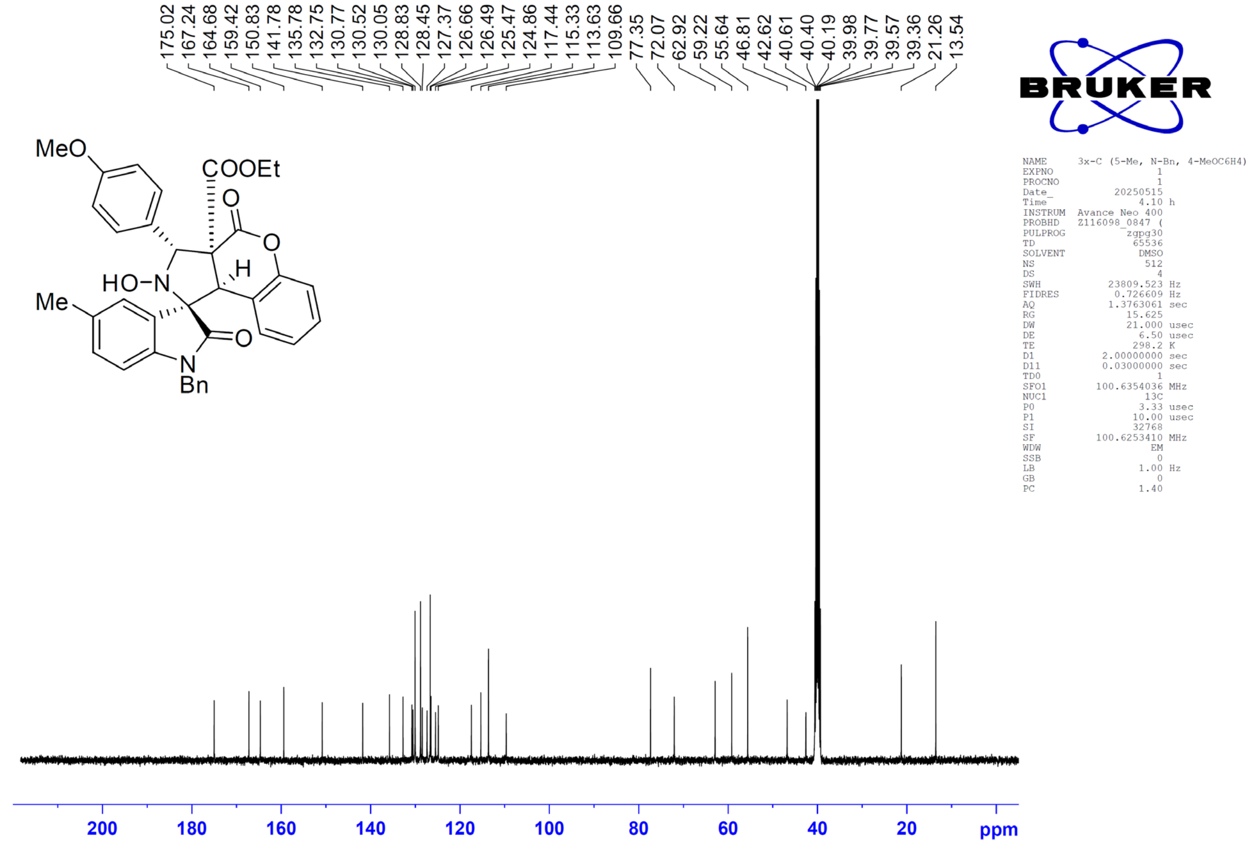

^{1}H NMR and ^{13}C NMR Spectra for Compound **3y**

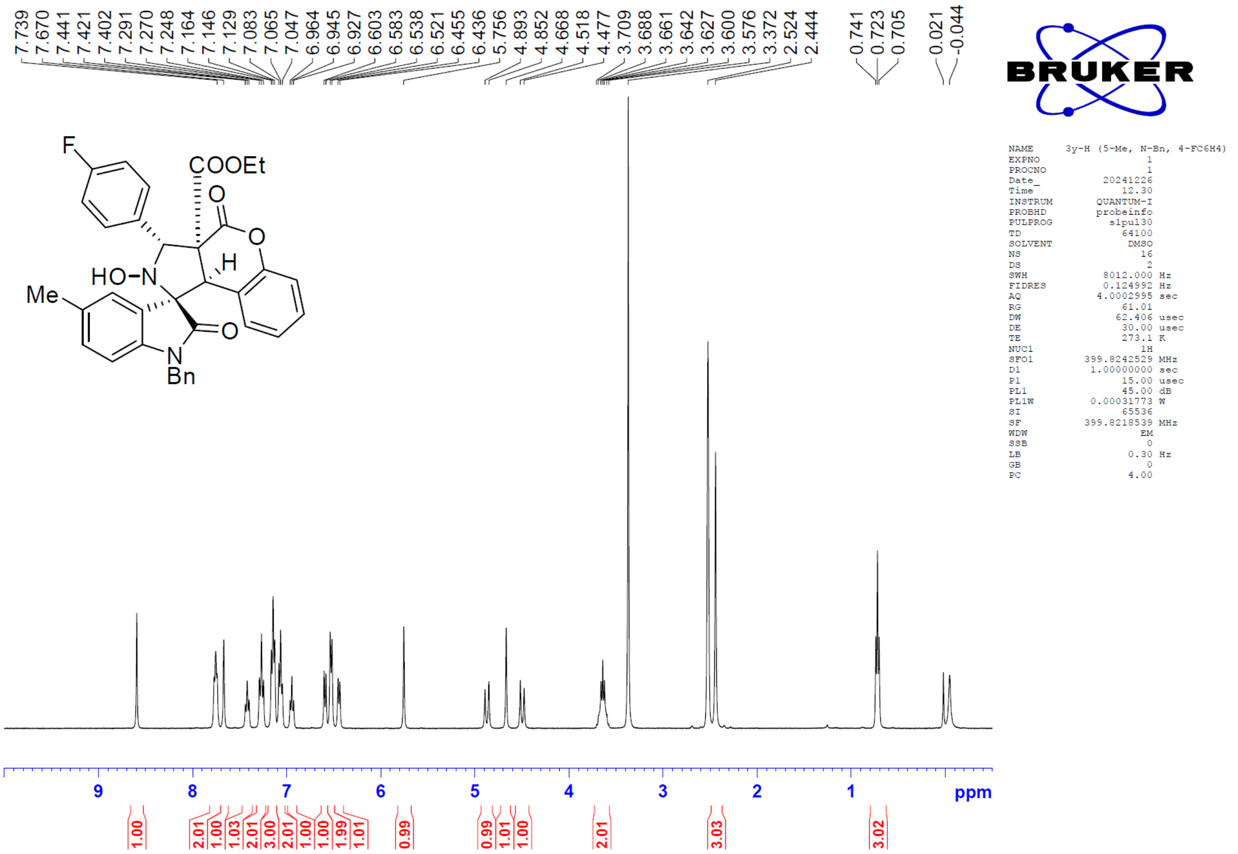

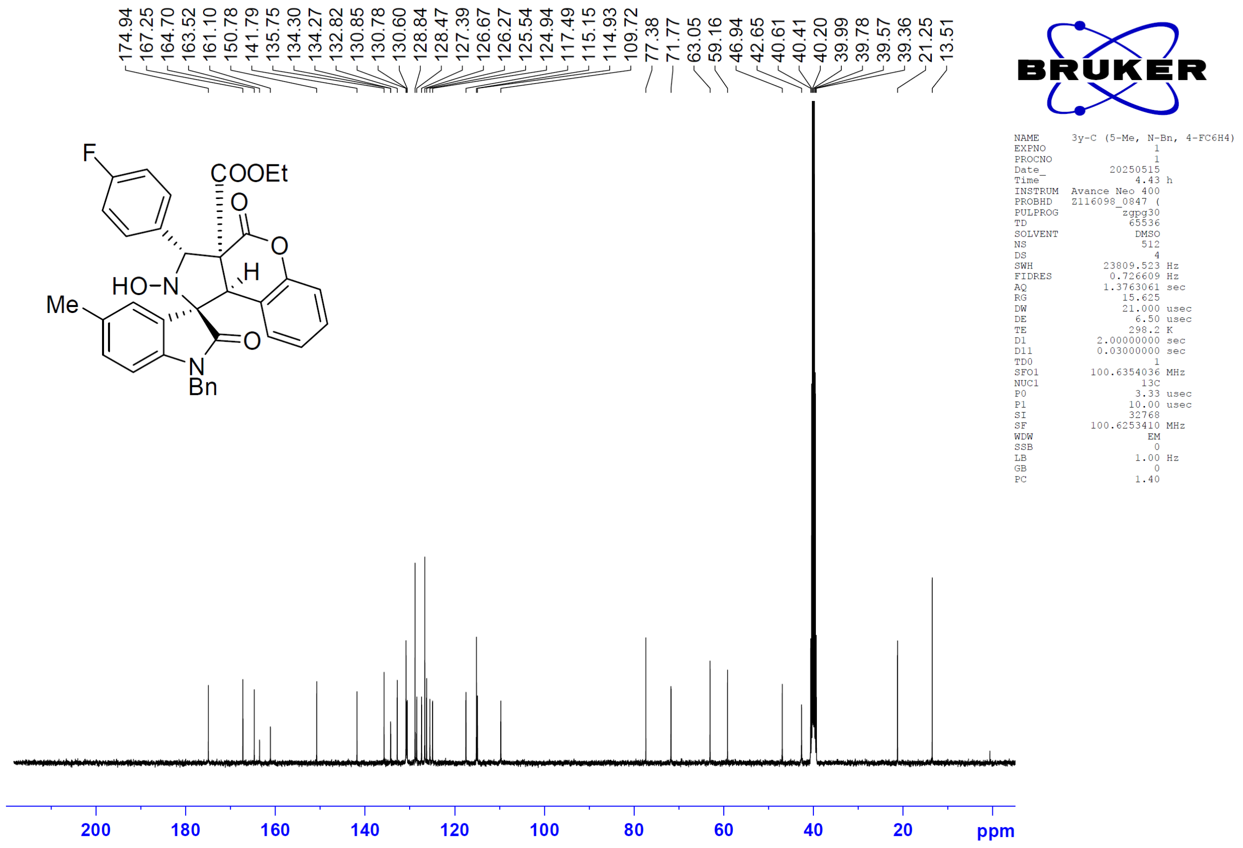

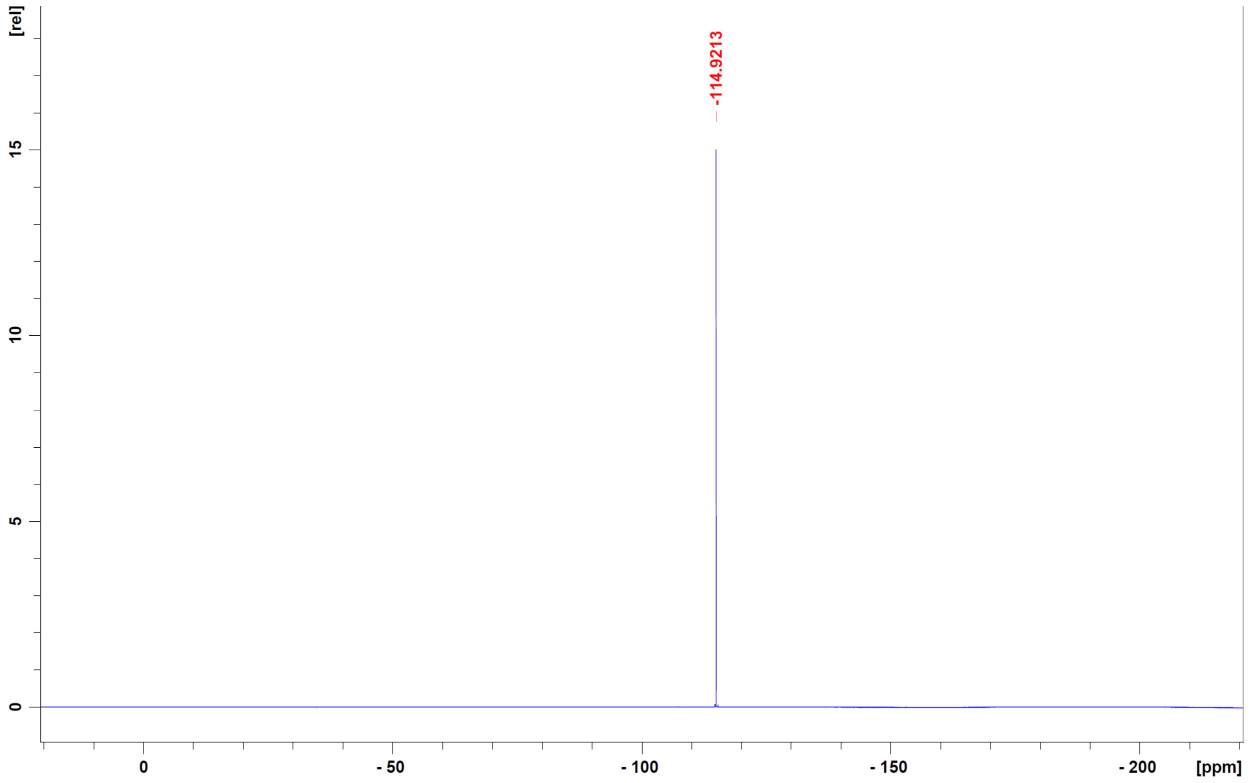

-114.9213
[rel]
15
10
5
0
0
- 50
- 100
- 150
- 200
[ppm]

^{1}H NMR and ^{13}C NMR Spectra for Compound **3z**

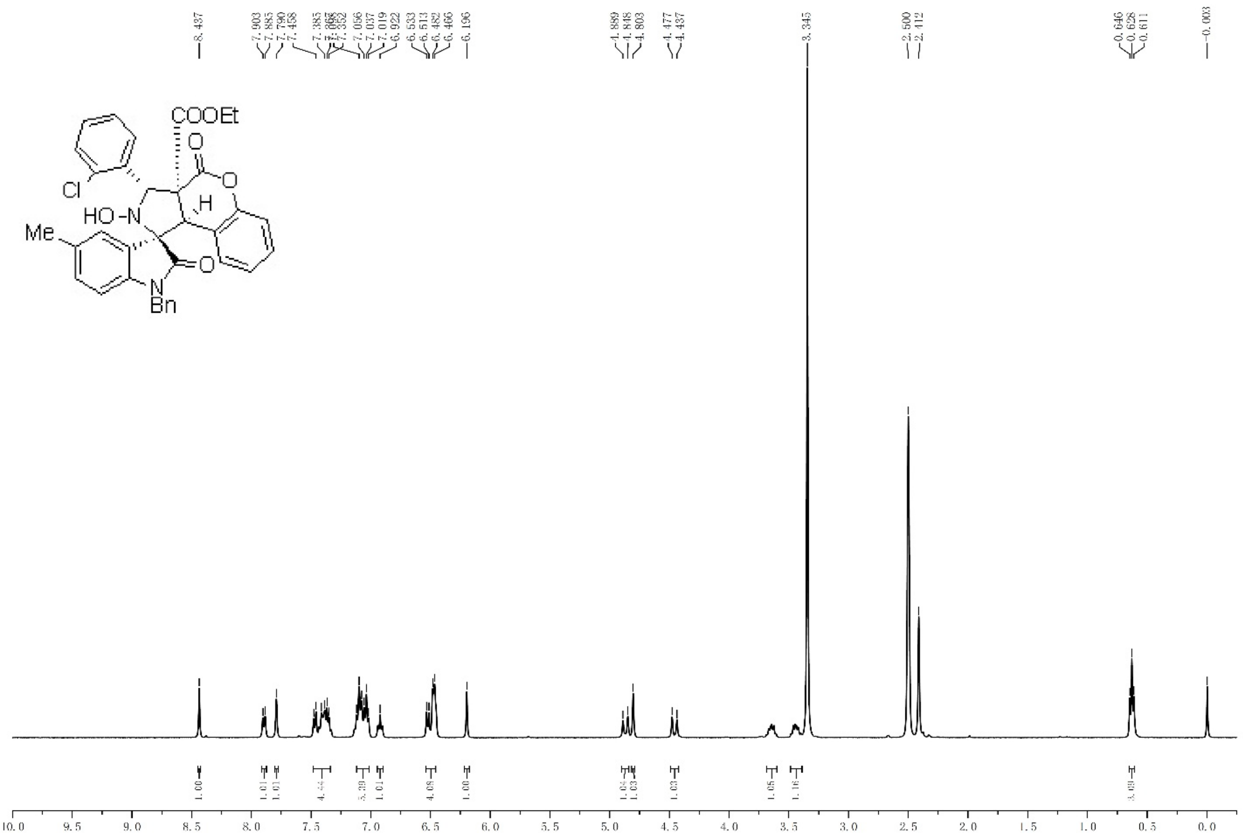

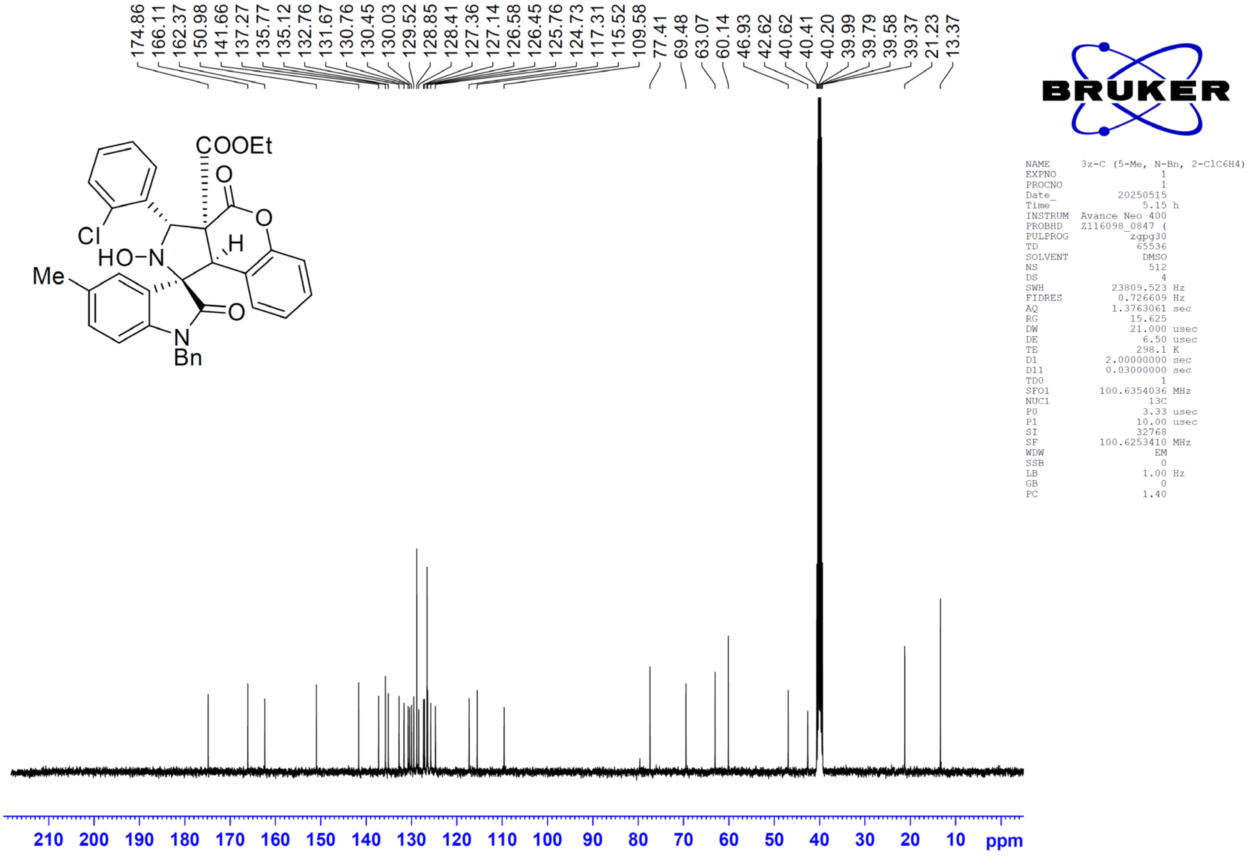

^{1}H NMR and ^{13}C NMR Spectra for Compound **3aa**

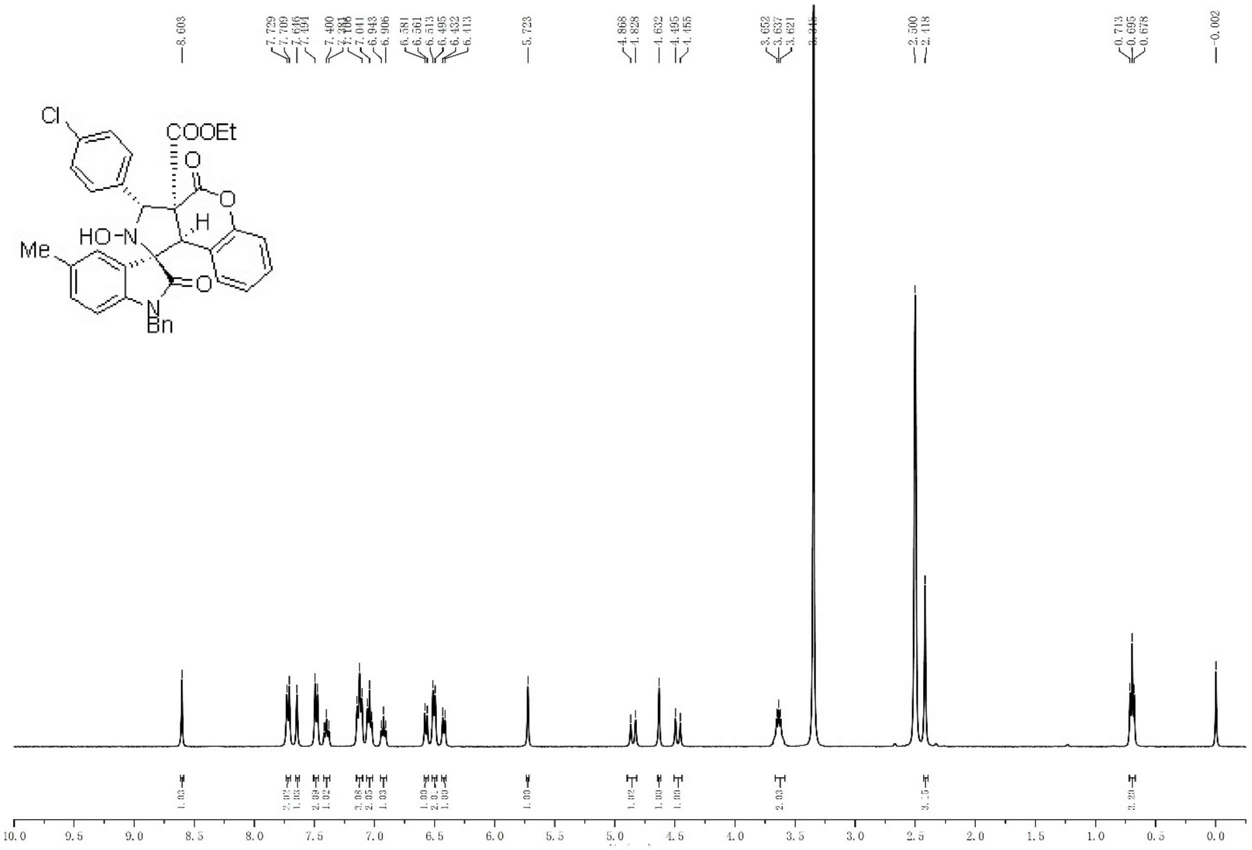

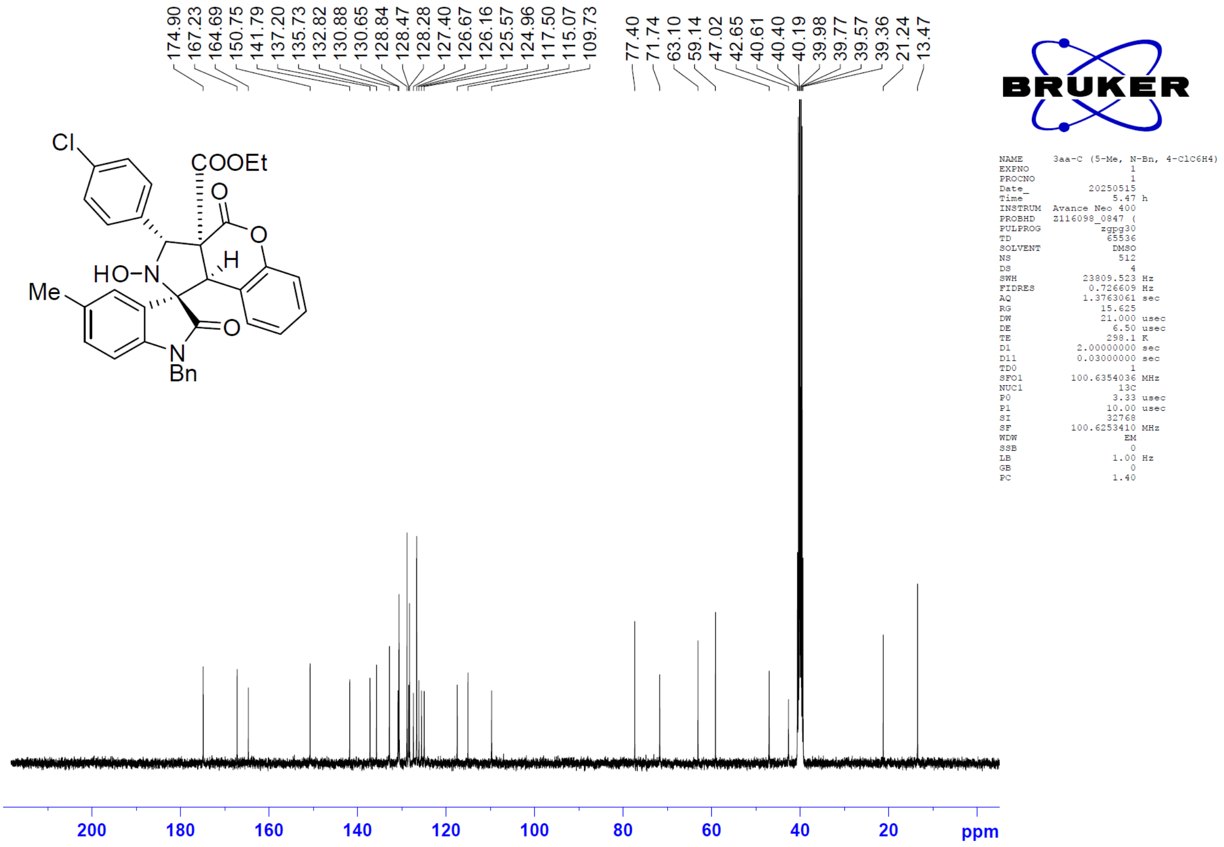

^{1}H NMR and ^{13}C NMR Spectra for Compound **3ab**

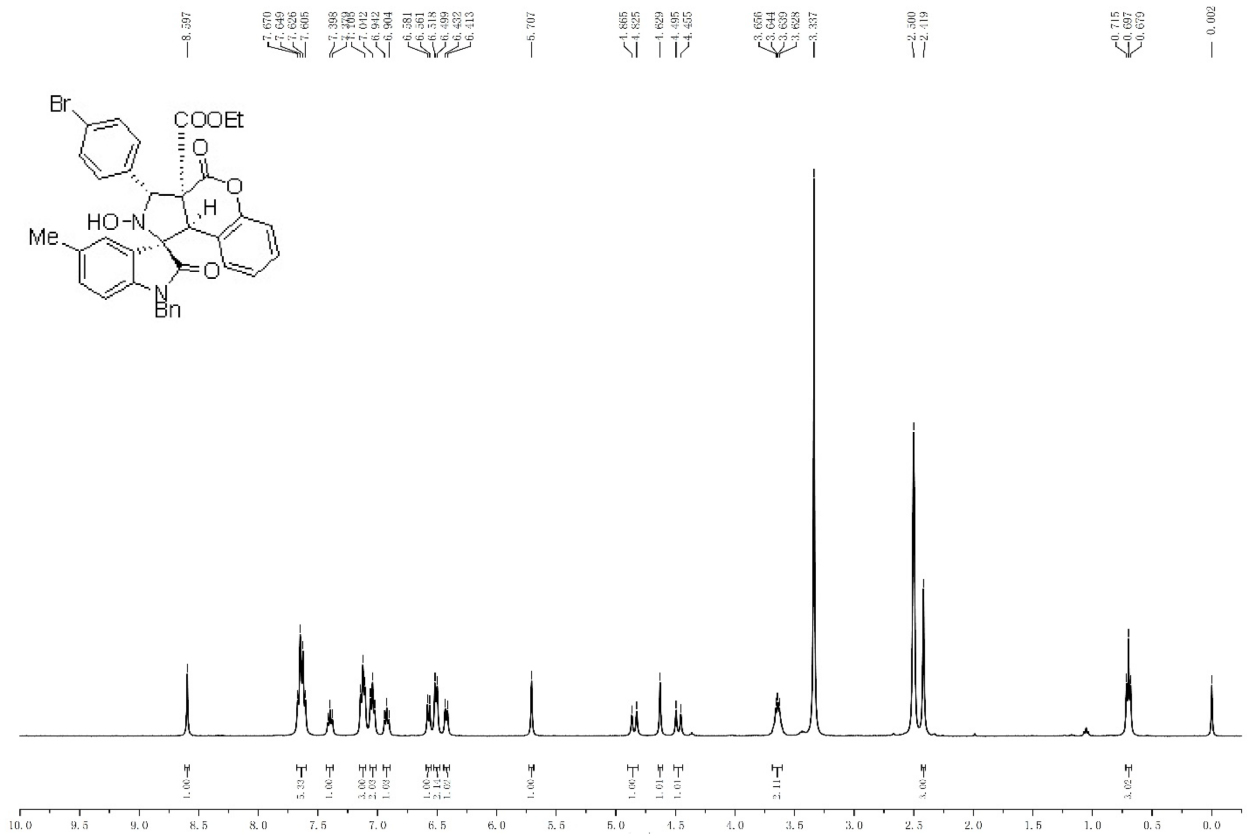

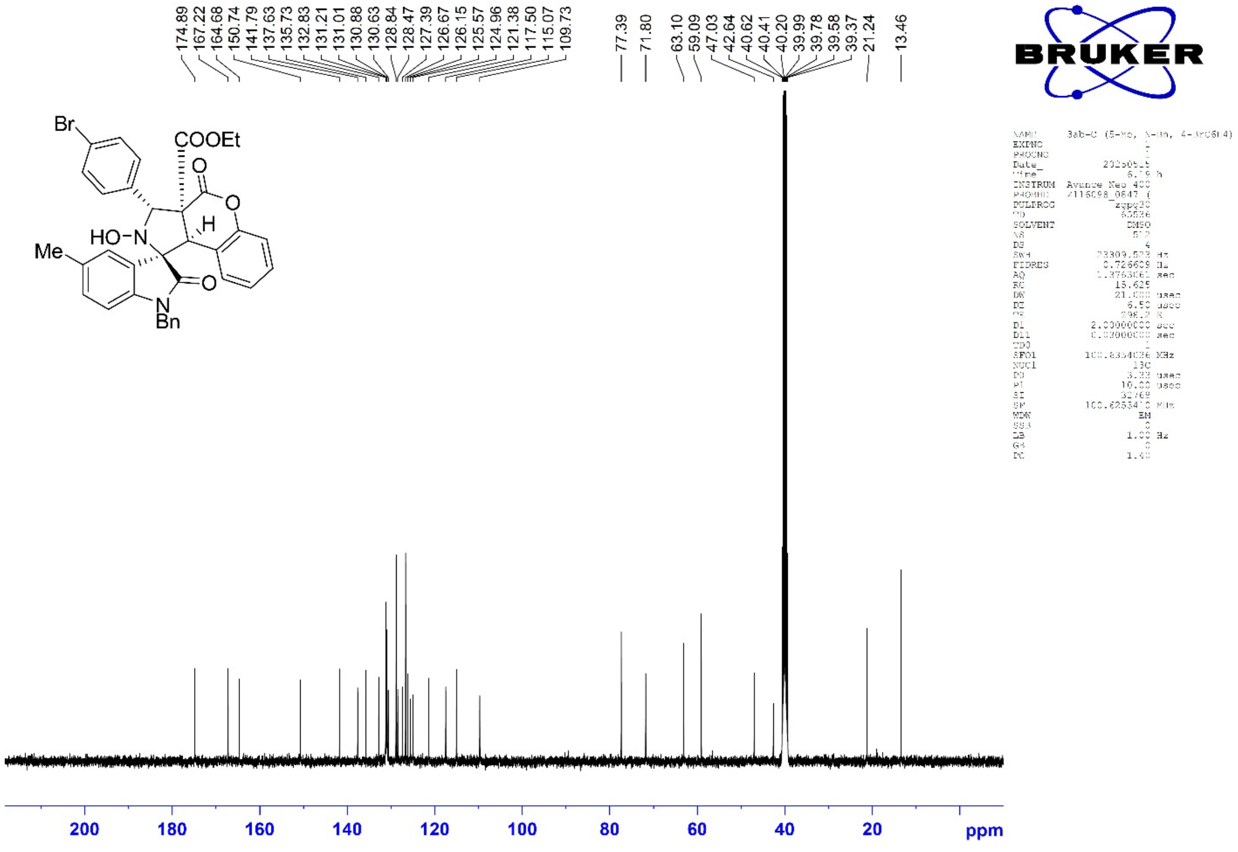

^{1}H NMR and ^{13}C NMR Spectra for Compound **3ac**

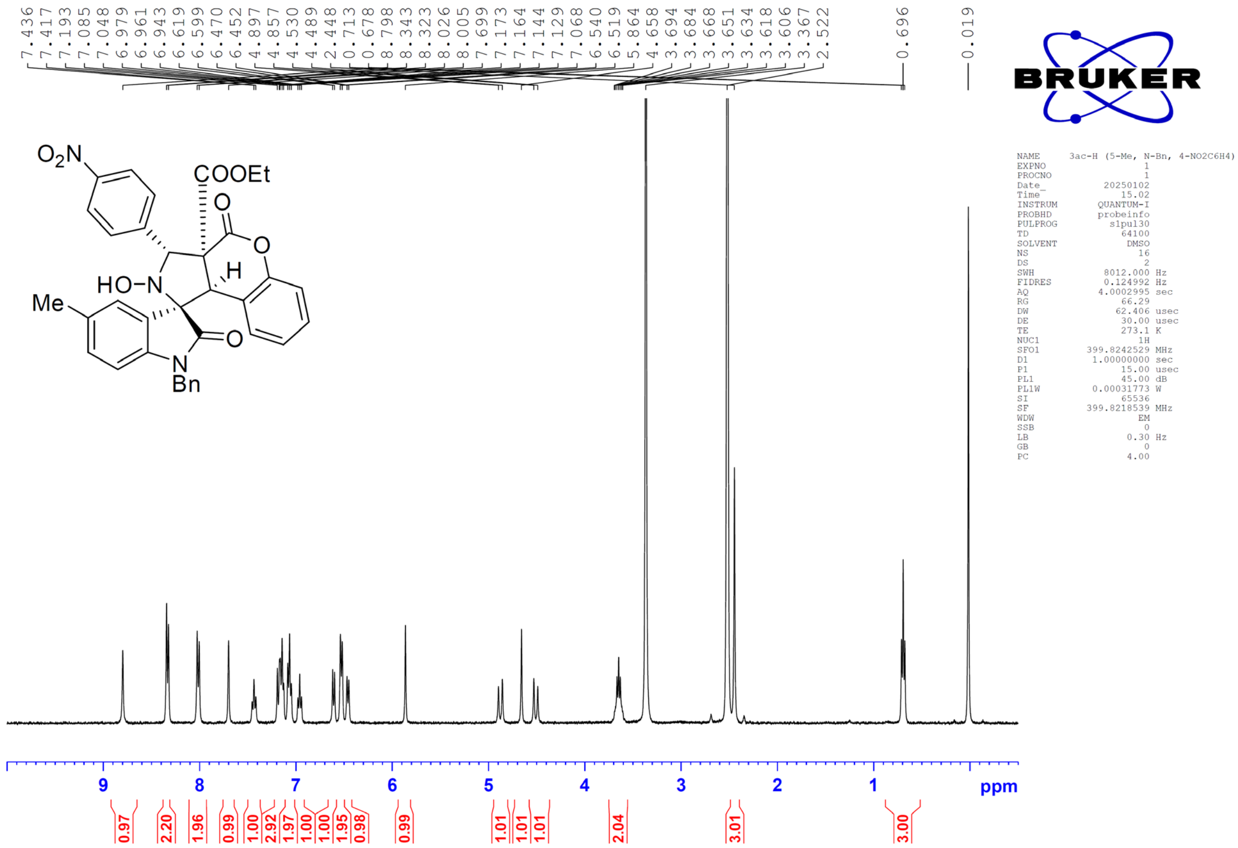

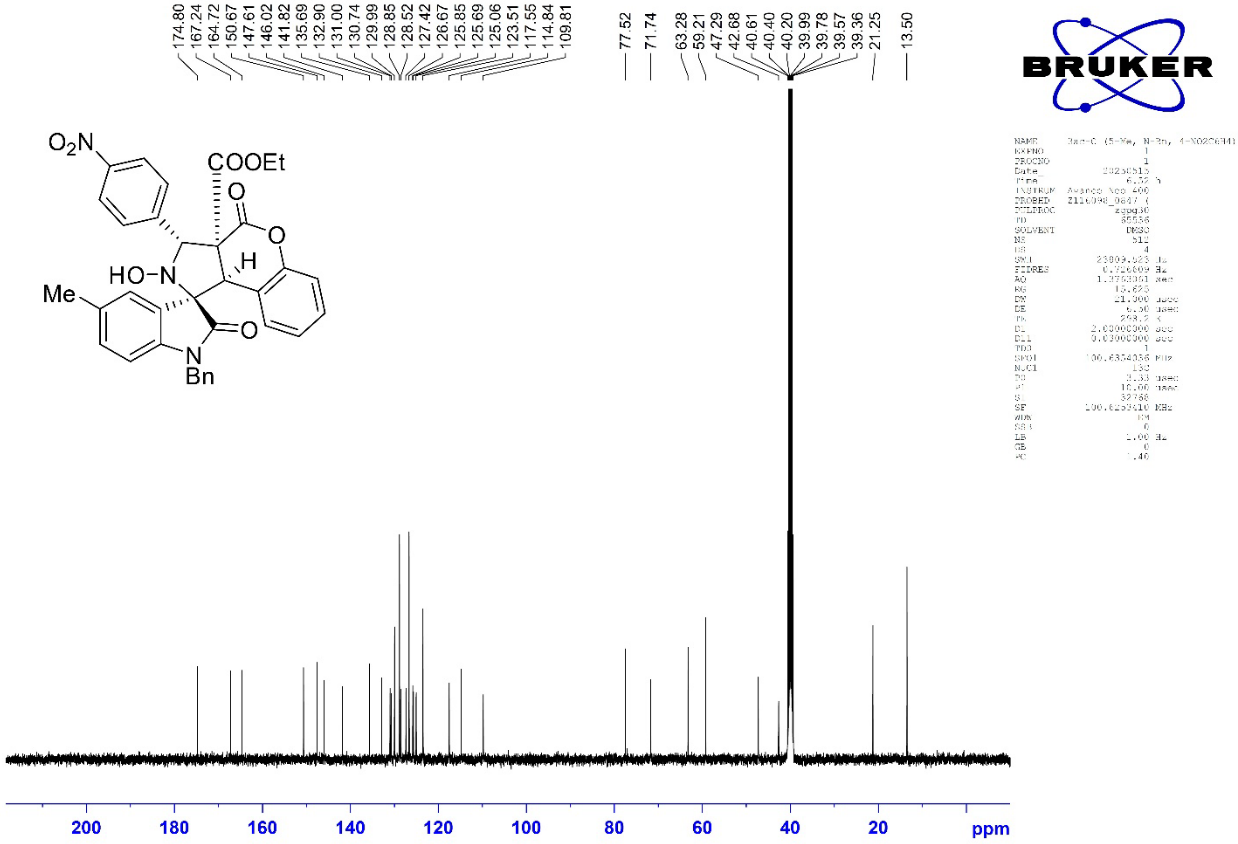

^{1}H NMR and ^{13}C NMR Spectra for Compound **3ad**

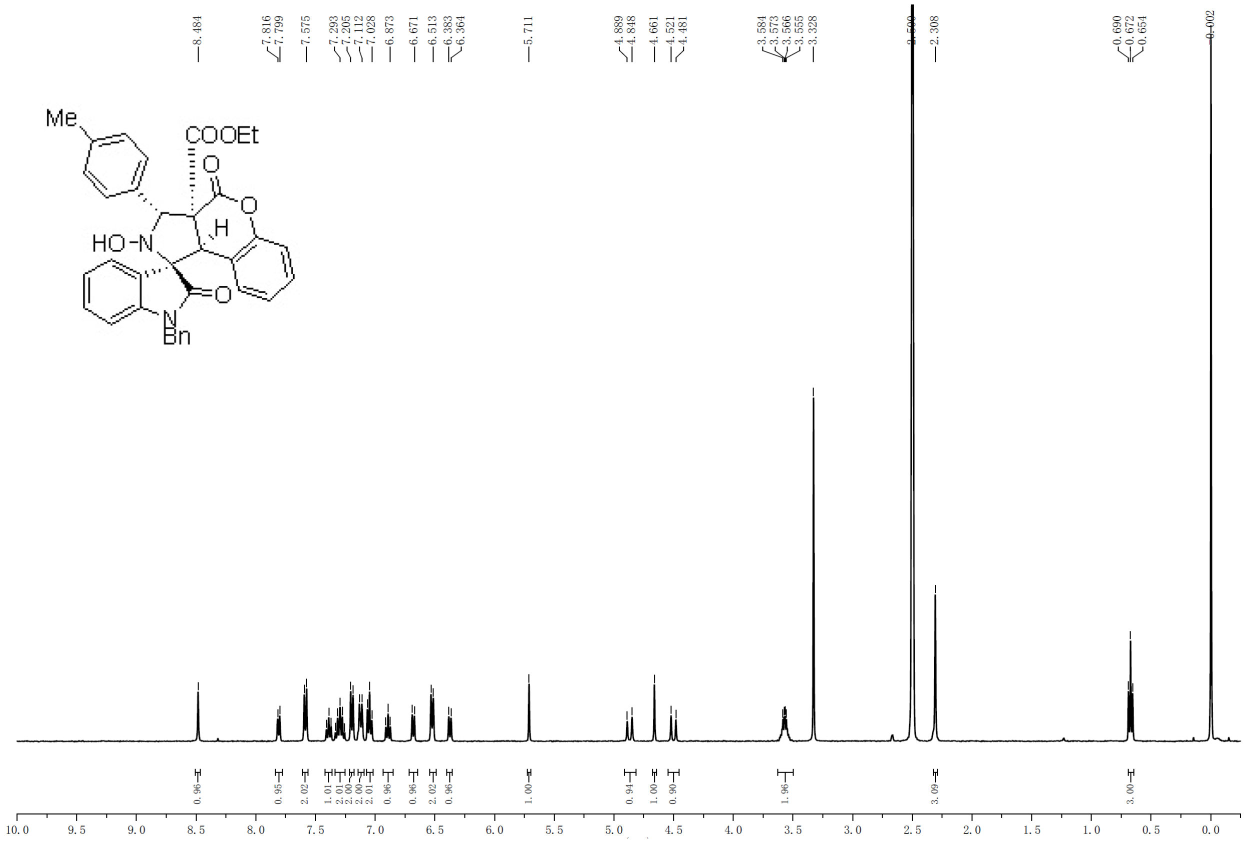

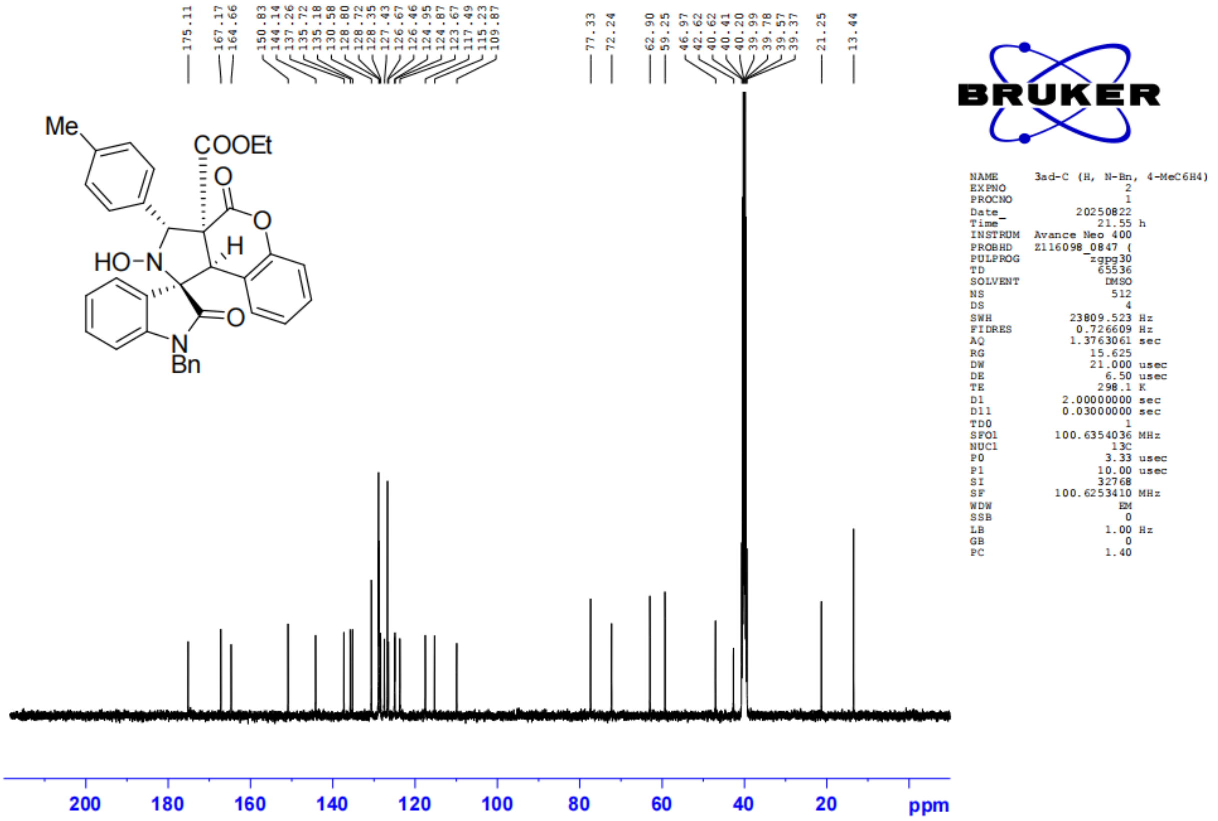

^{1}H NMR and ^{13}C NMR Spectra for Compound **3ae**

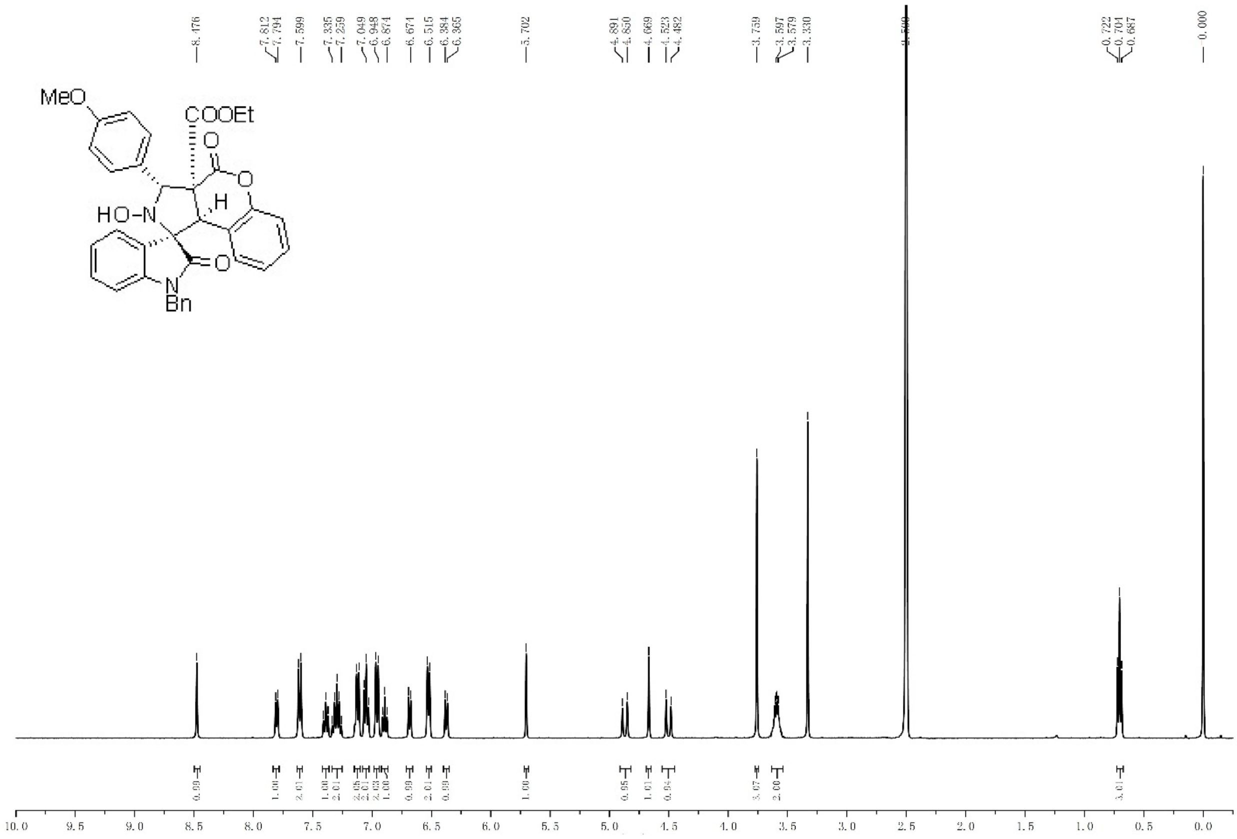

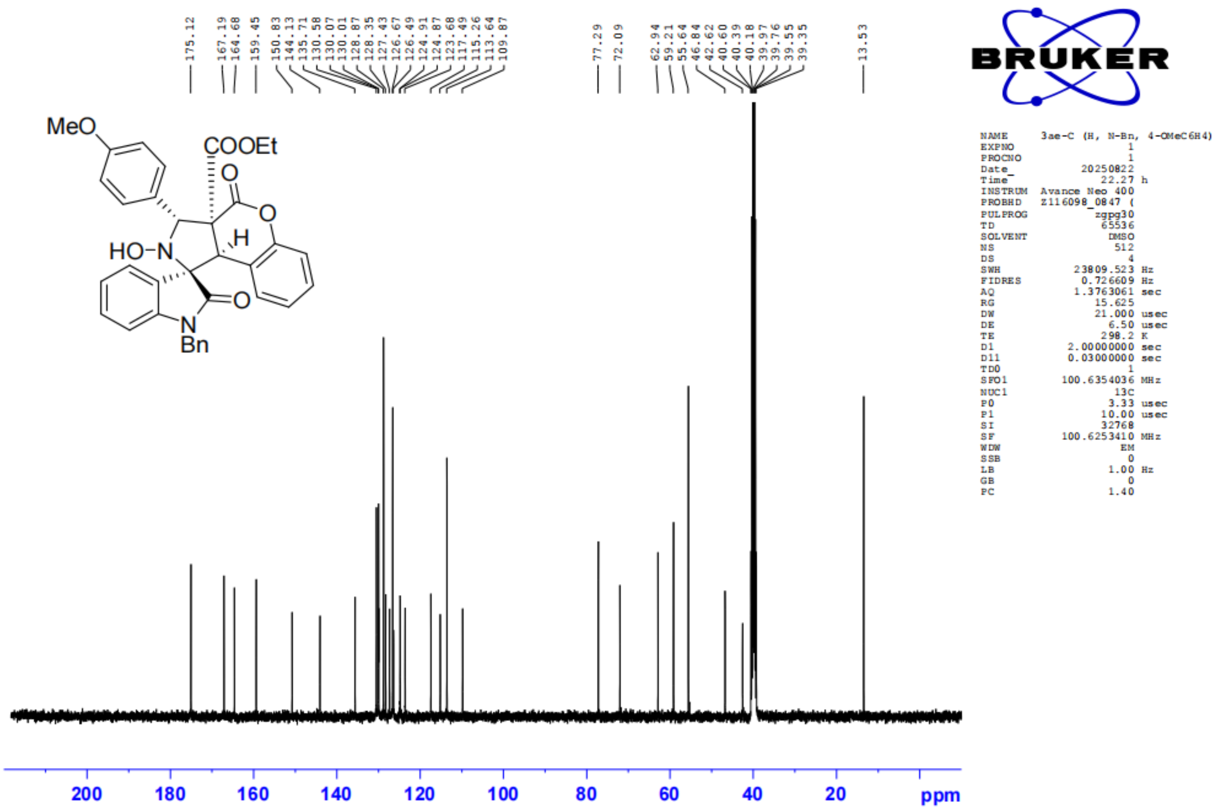

^{1}H NMR and ^{13}C NMR Spectra for Compound **3af**

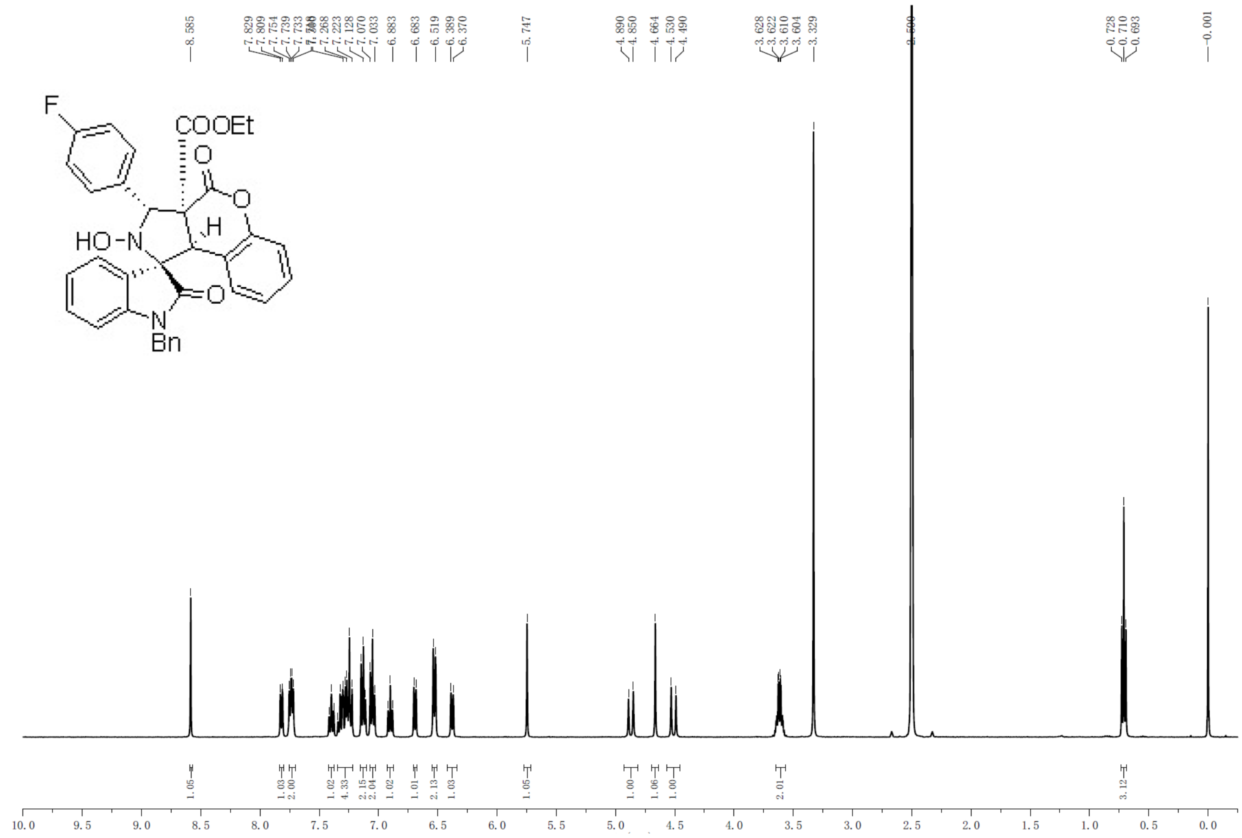

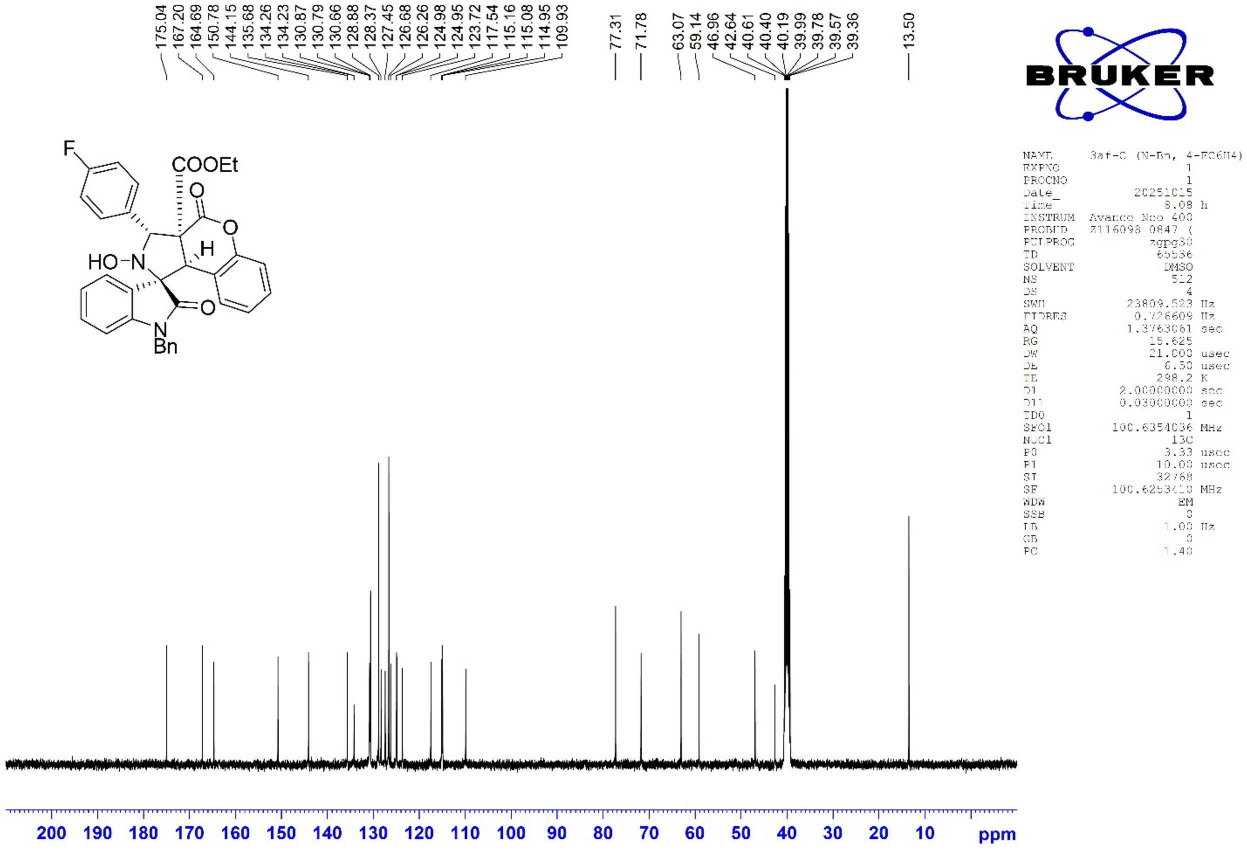

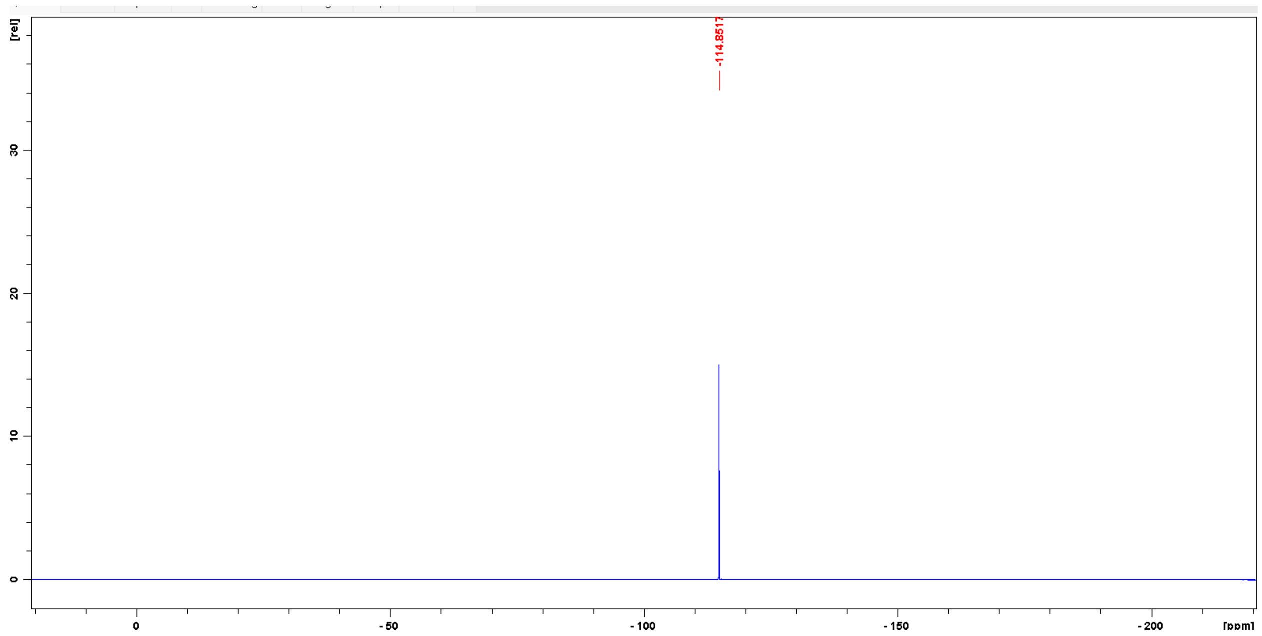
-114.8517
[rel]
30
20
10
0
0
-50
-100
-150
-200
[ppm]

^{1}H NMR and ^{13}C NMR Spectra for Compound **3ag**

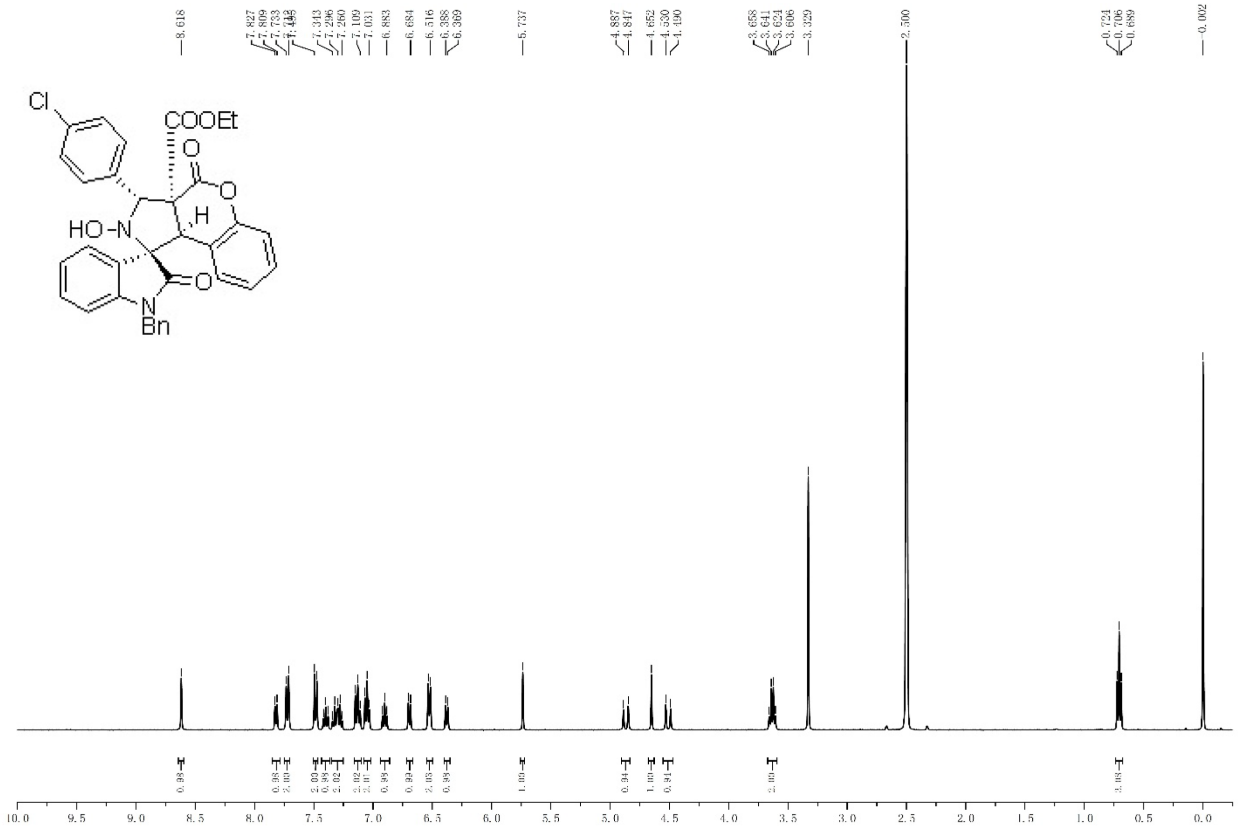

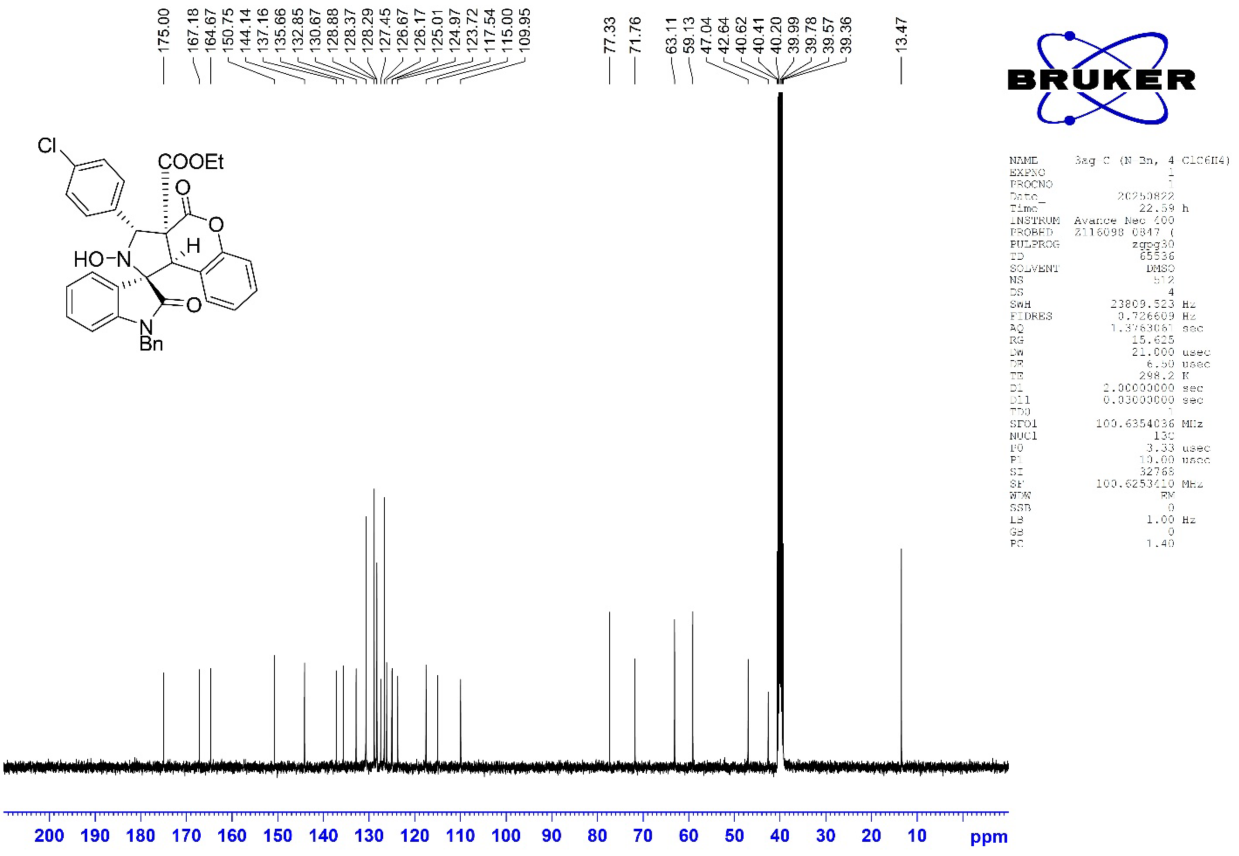

^{1}H NMR and ^{13}C NMR Spectra for Compound **3ah**

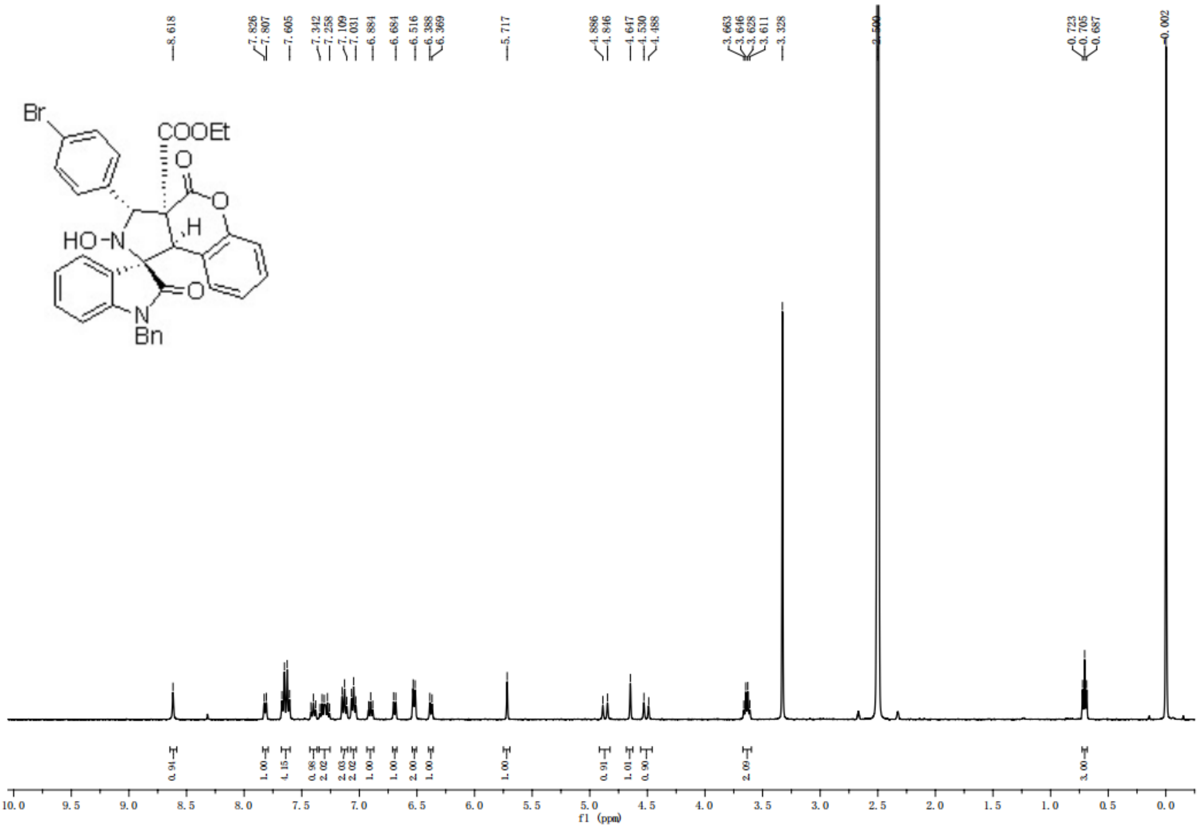

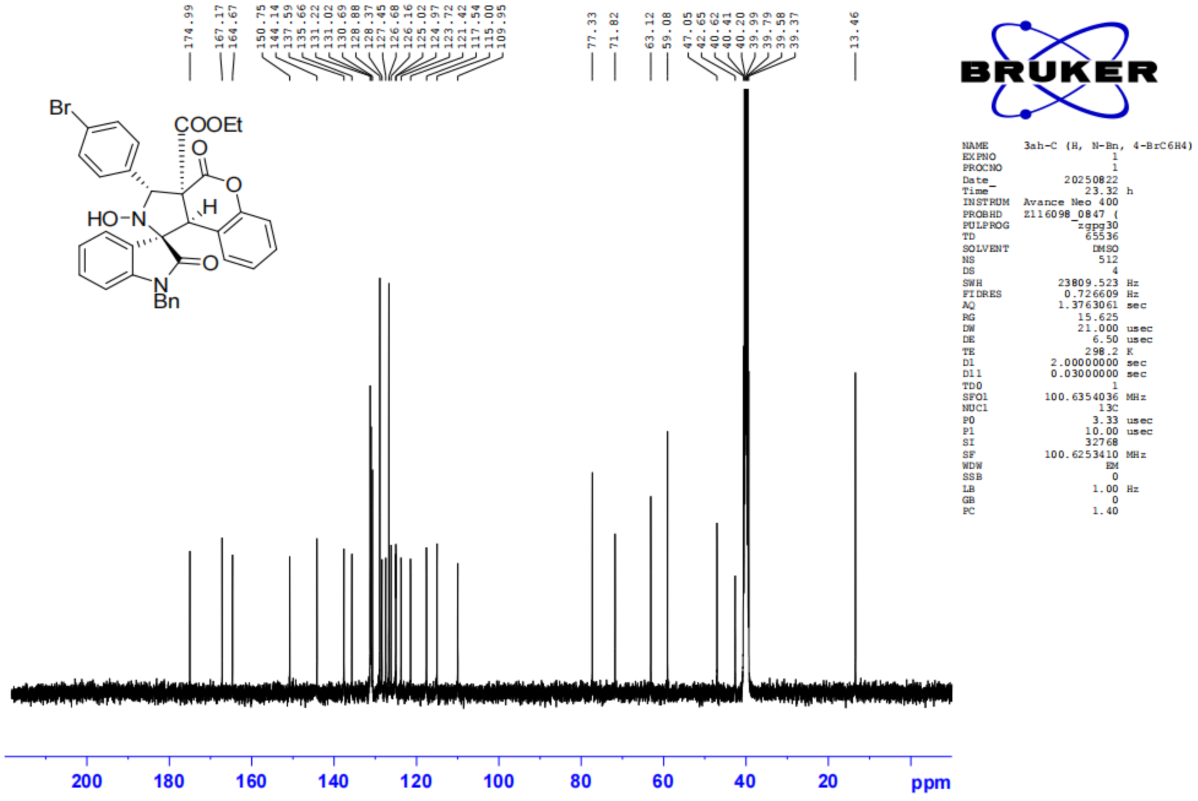

^{1}H NMR and ^{13}C NMR Spectra for Compound **3ai**

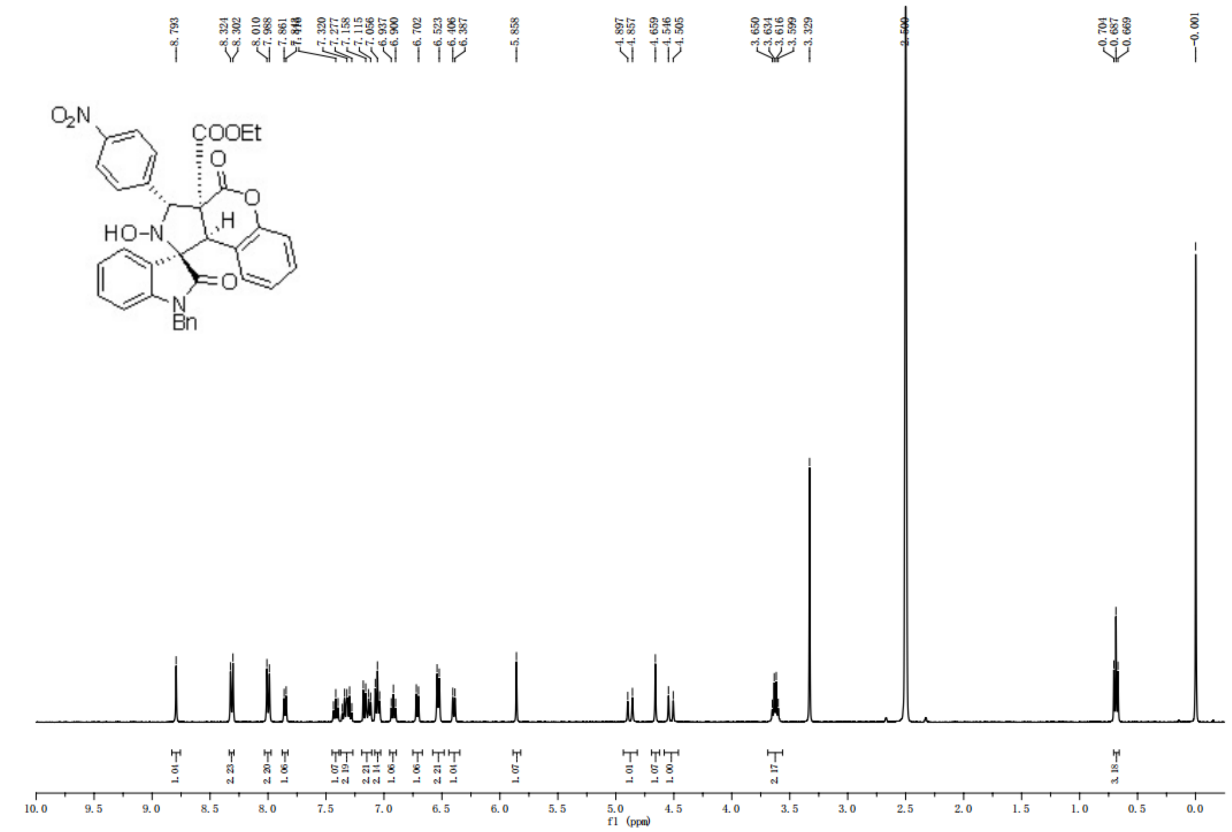

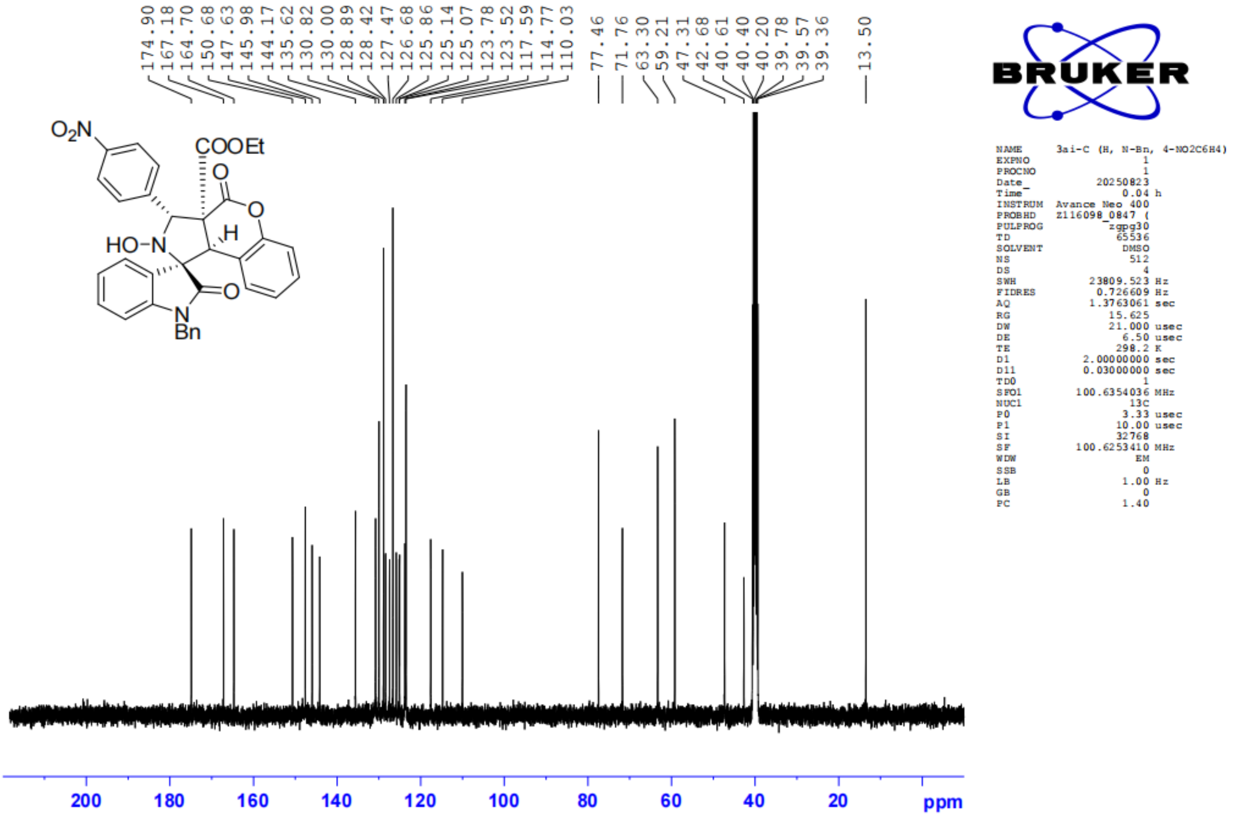

^{1}H NMR and ^{13}C NMR Spectra for Compound **4a**

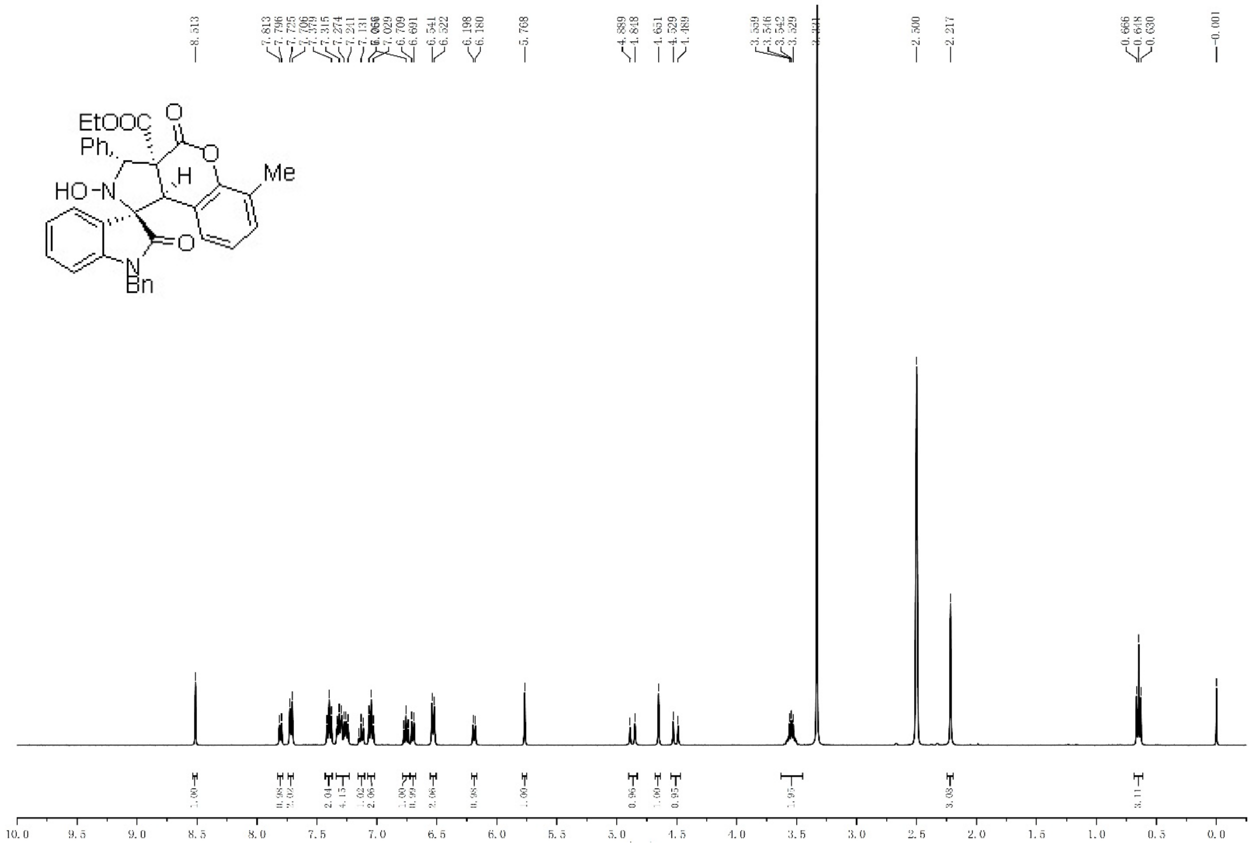

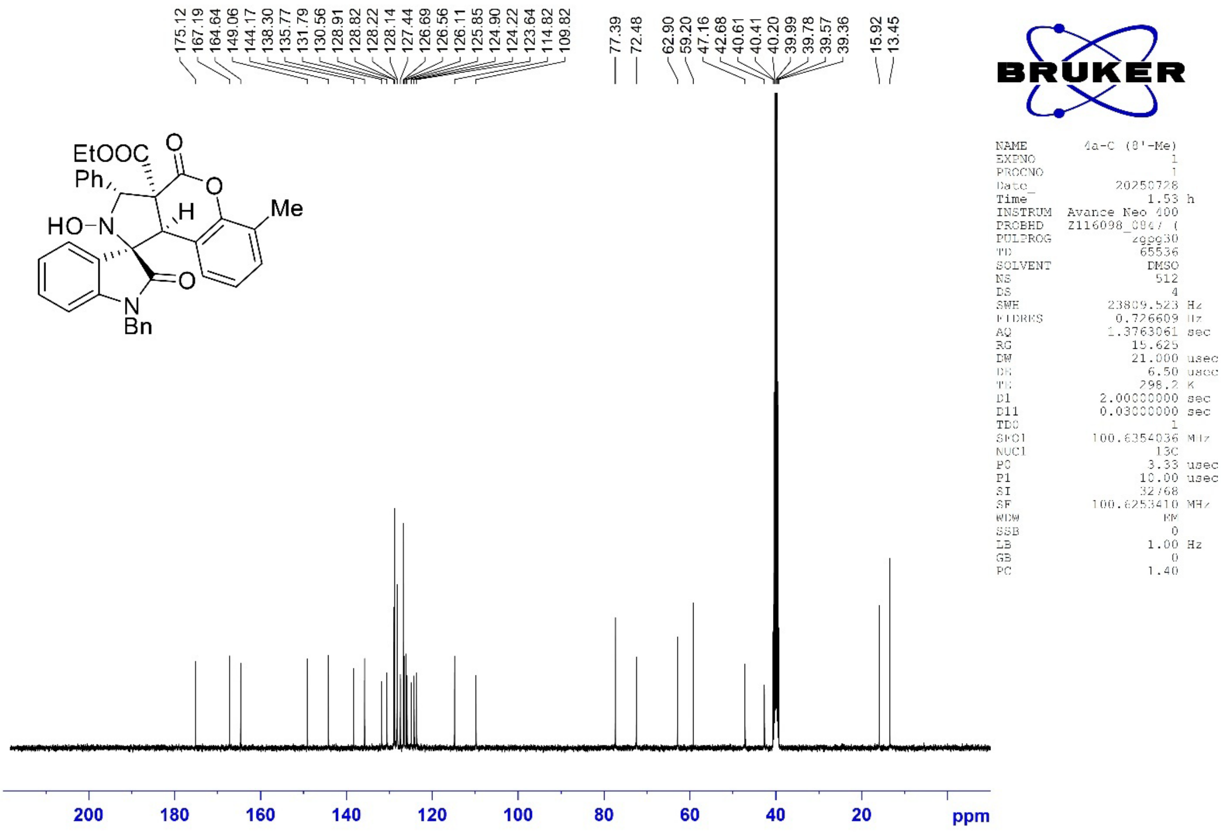

^{1}H NMR and ^{13}C NMR Spectra for Compound **4b**

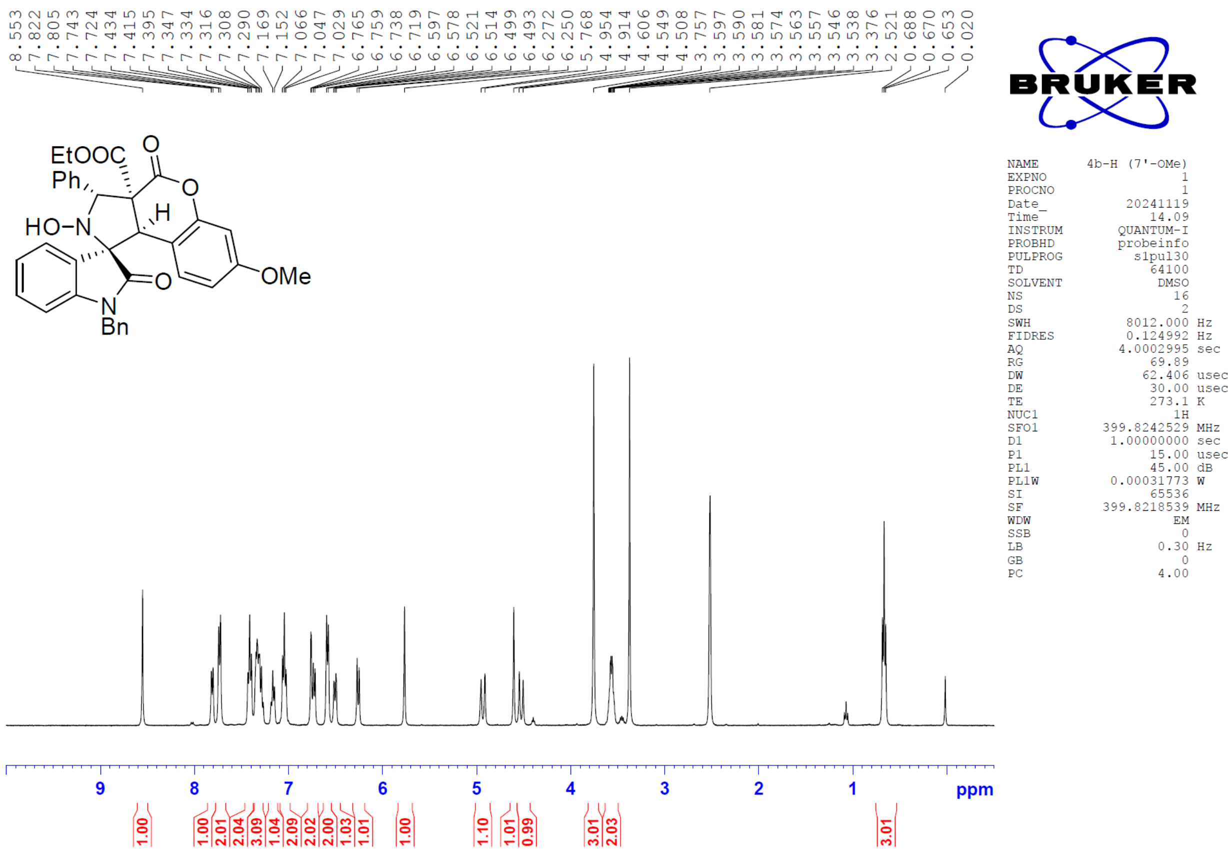

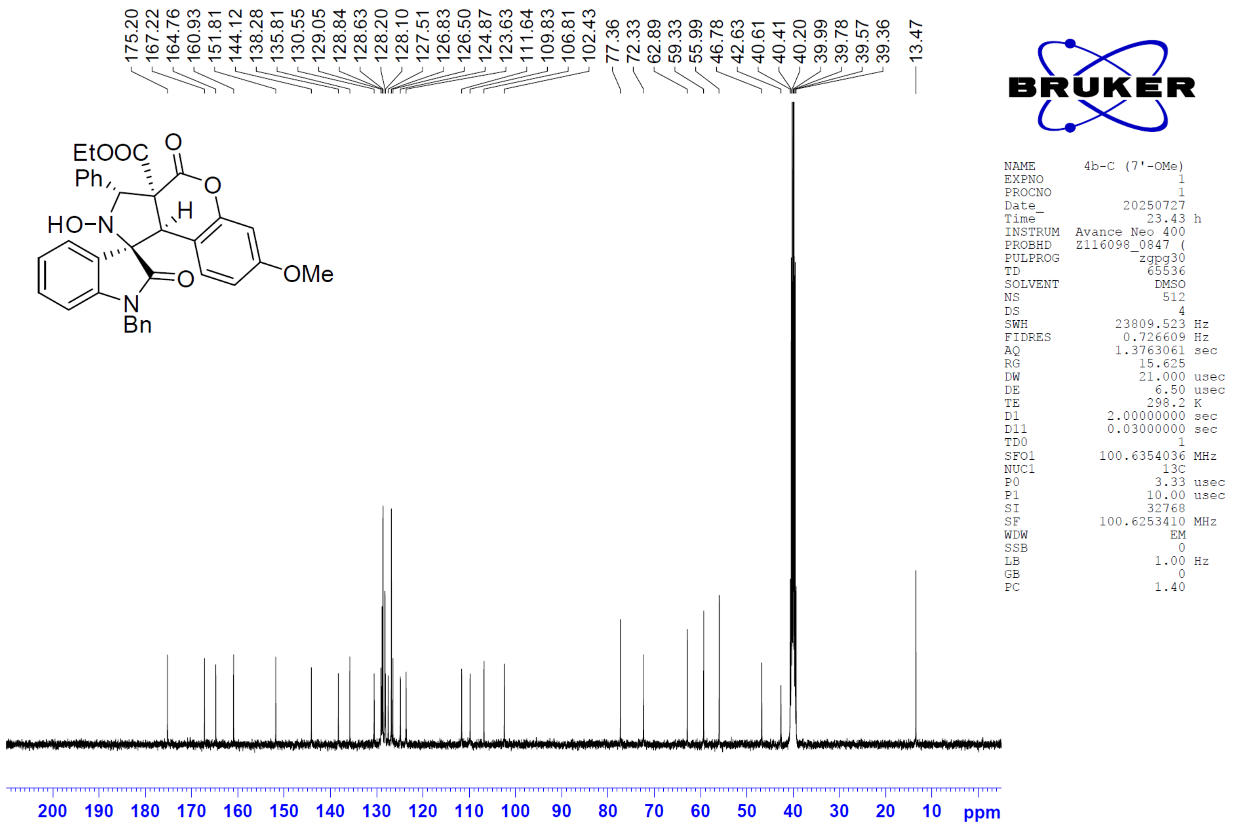

^{1}H NMR and ^{13}C NMR Spectra for Compound **4c**

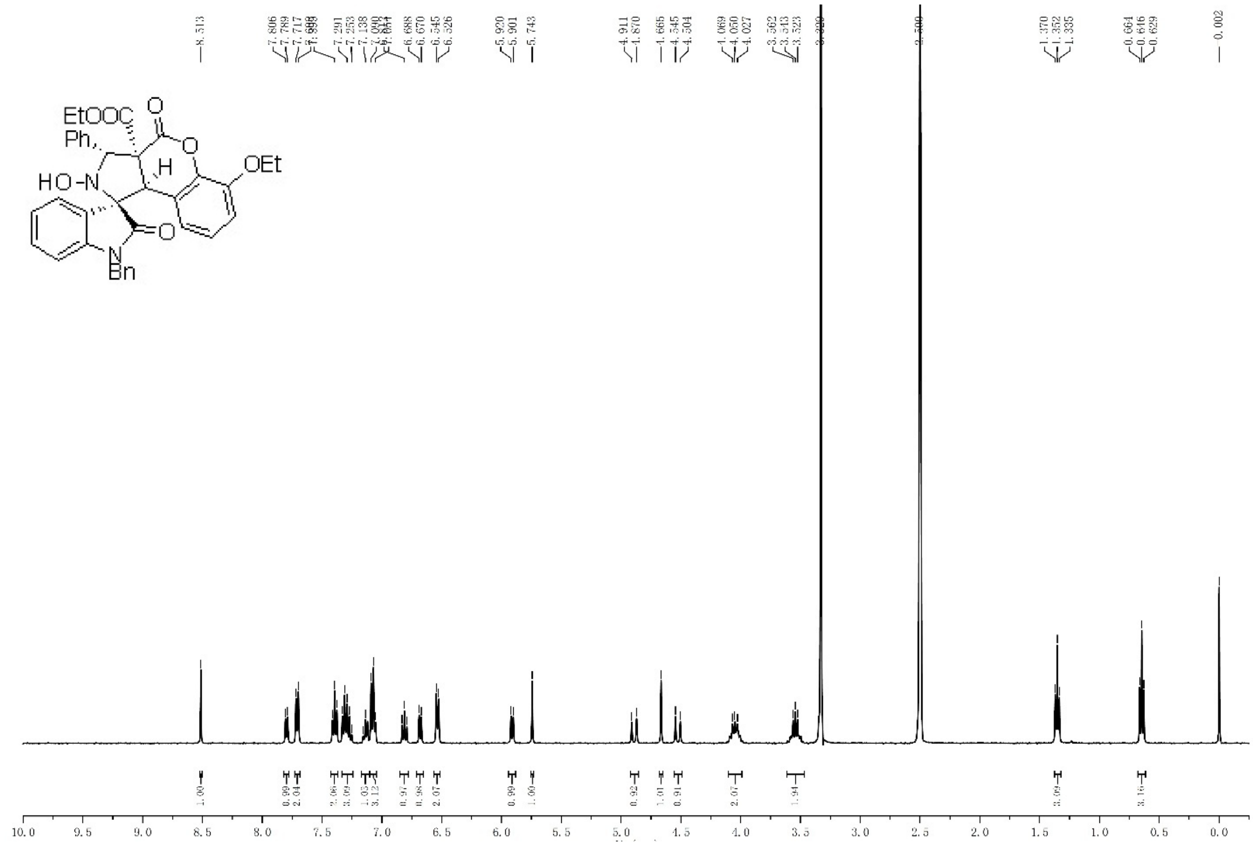

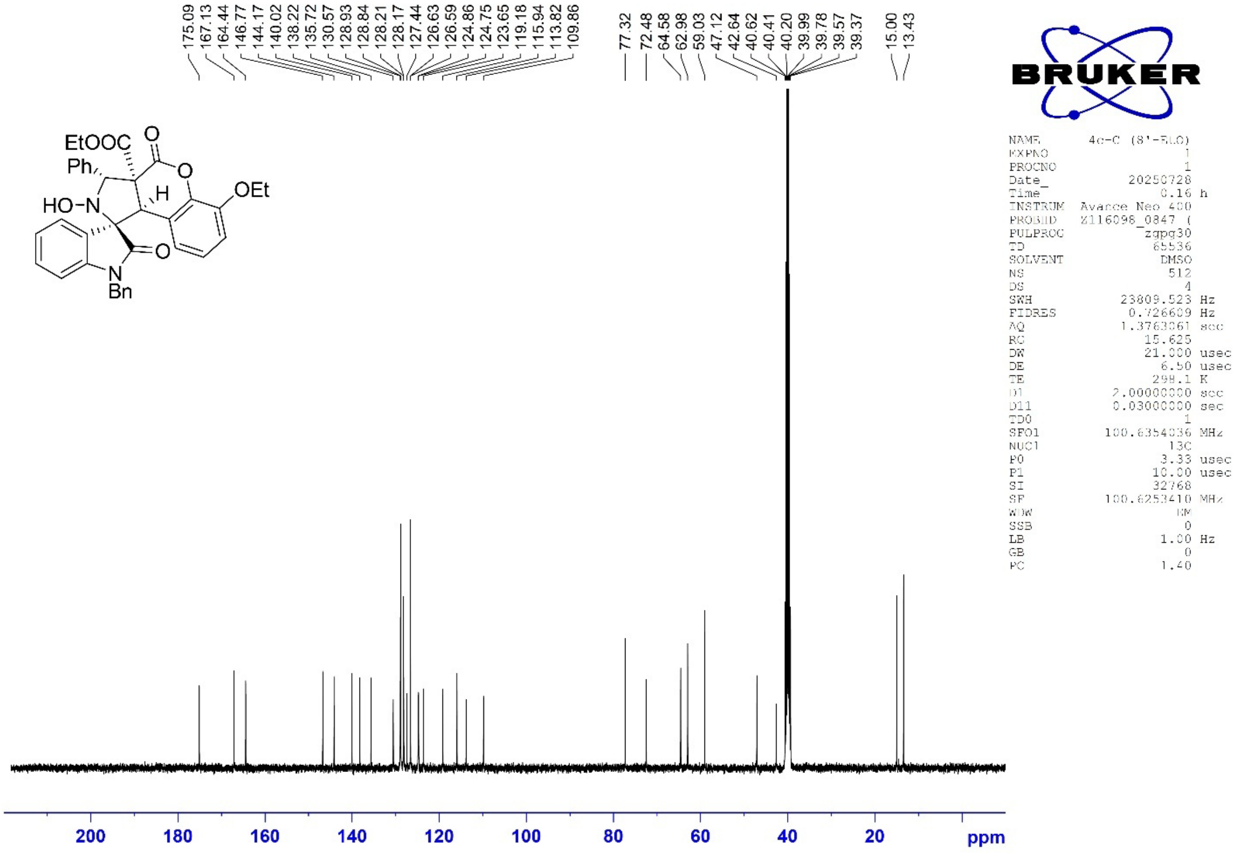

^{1}H NMR, ^{13}C NMR and ^{19}F NMR Spectra for Compound **4d**

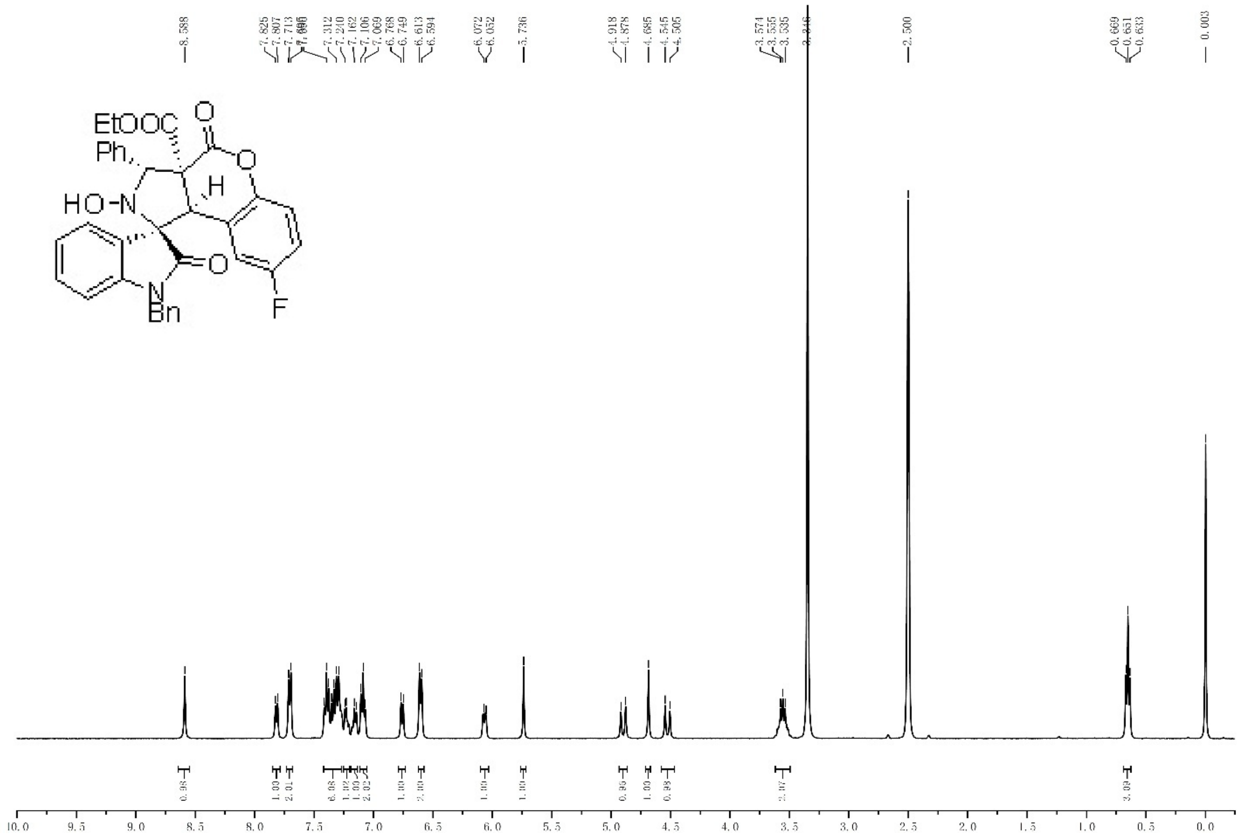

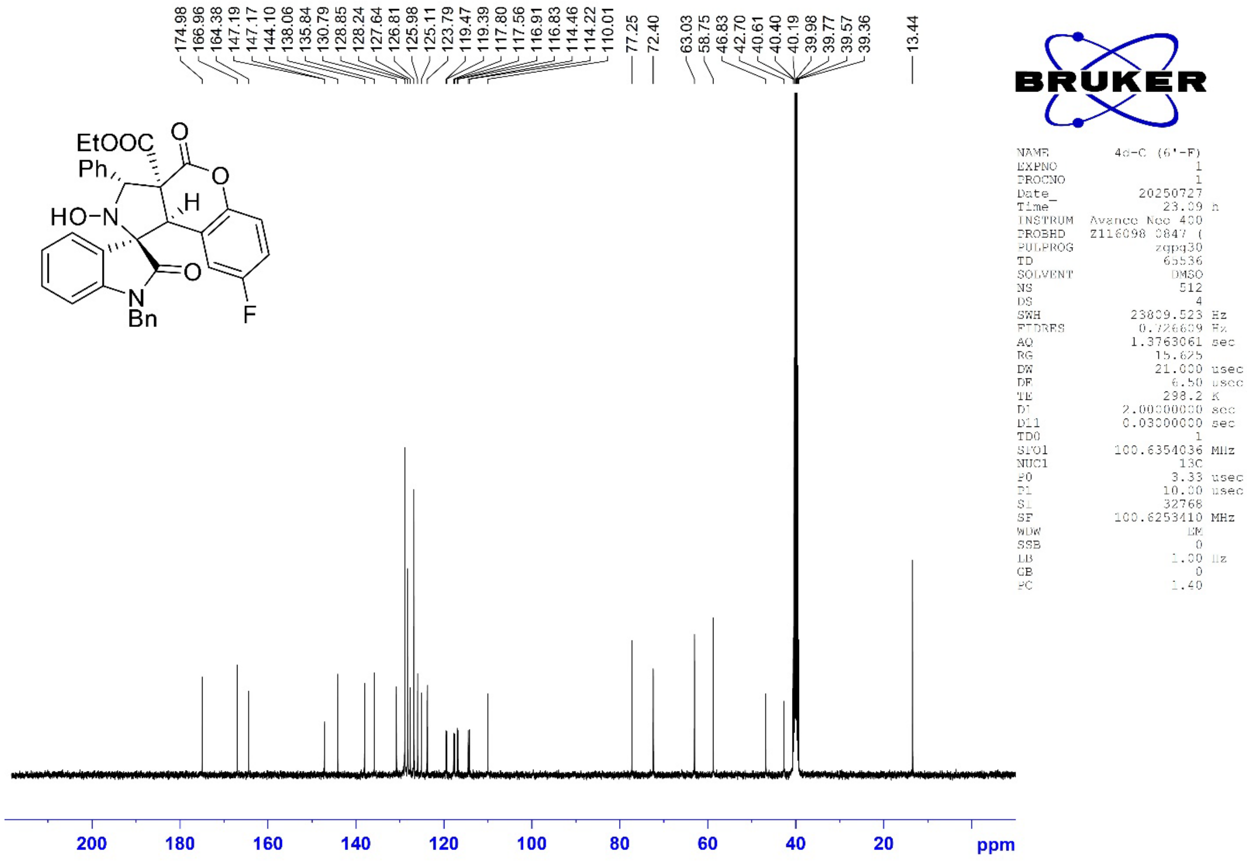

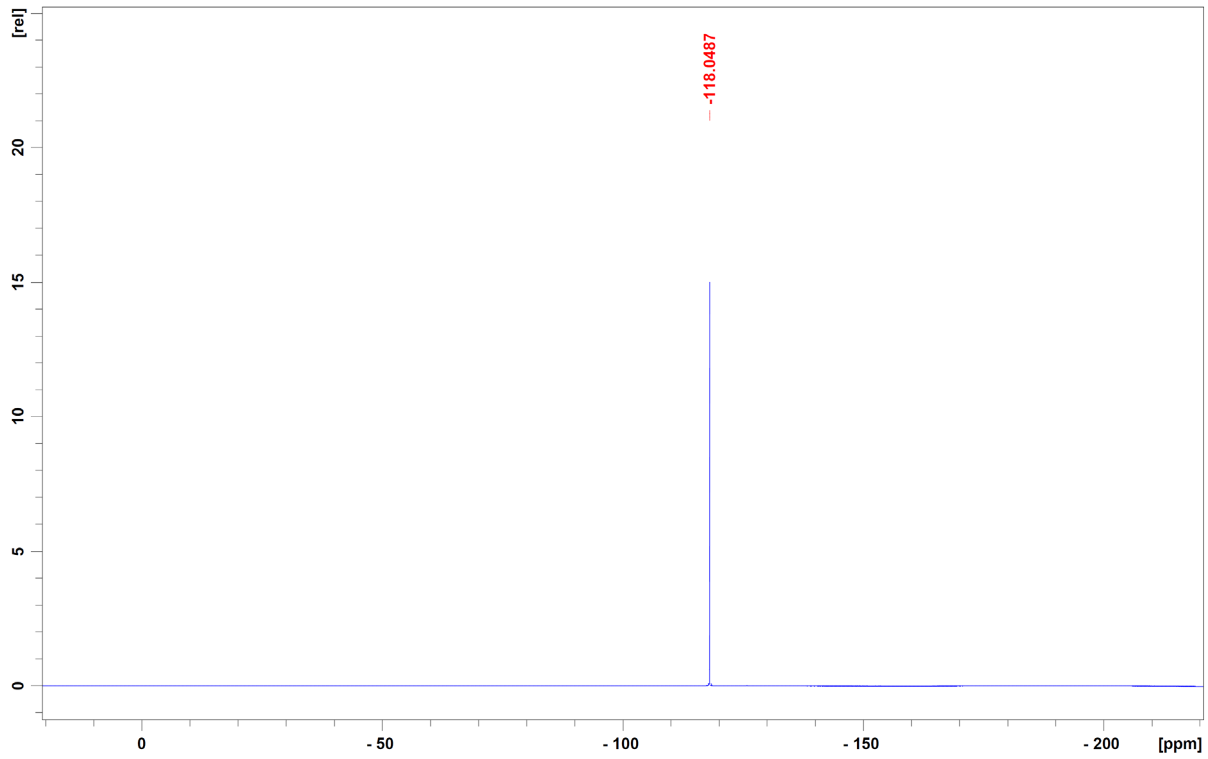
-118.0487
[rel]
20
15
10
5
0
0
- 50
- 100
- 150
- 200
[ppm]

^{1}H NMR and ^{13}C NMR Spectra for Compound **4e**

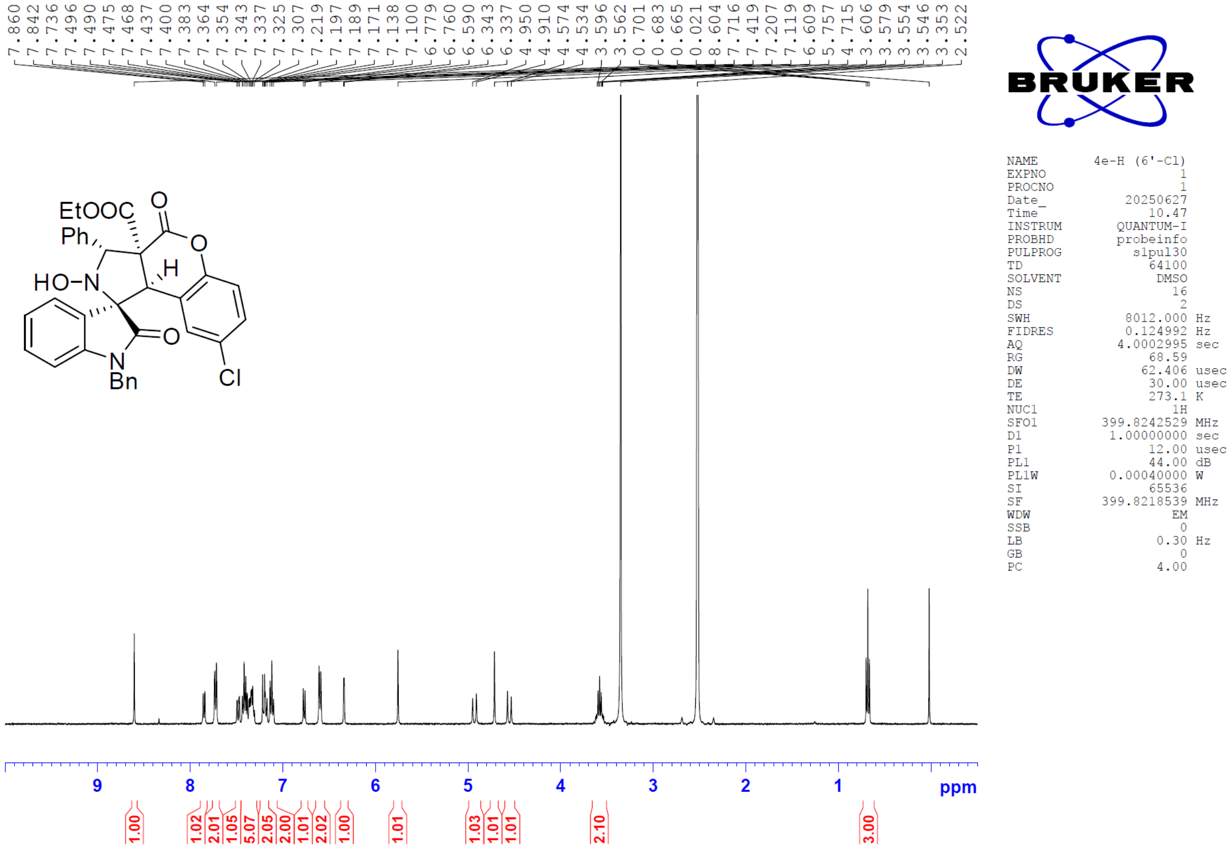

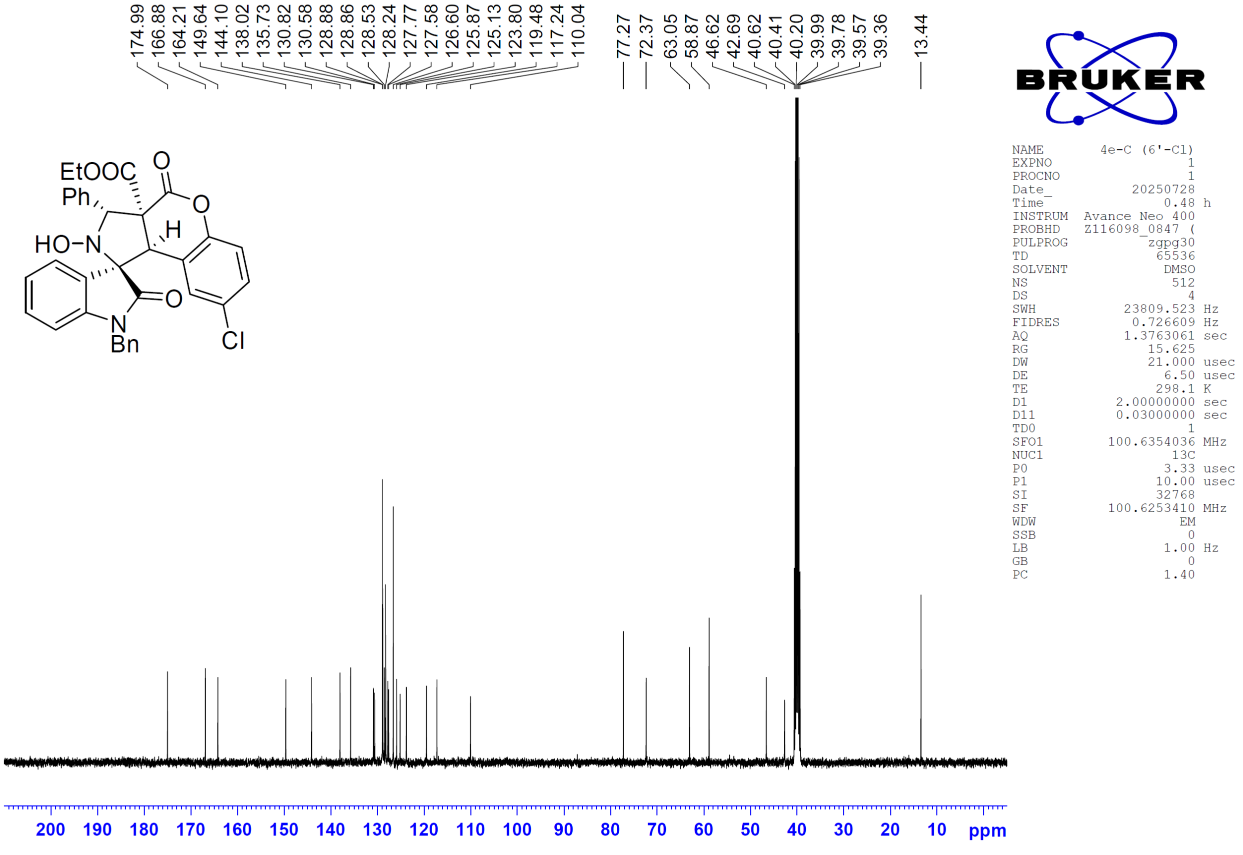

^{1}H NMR and ^{13}C NMR Spectra for Compound **4f**

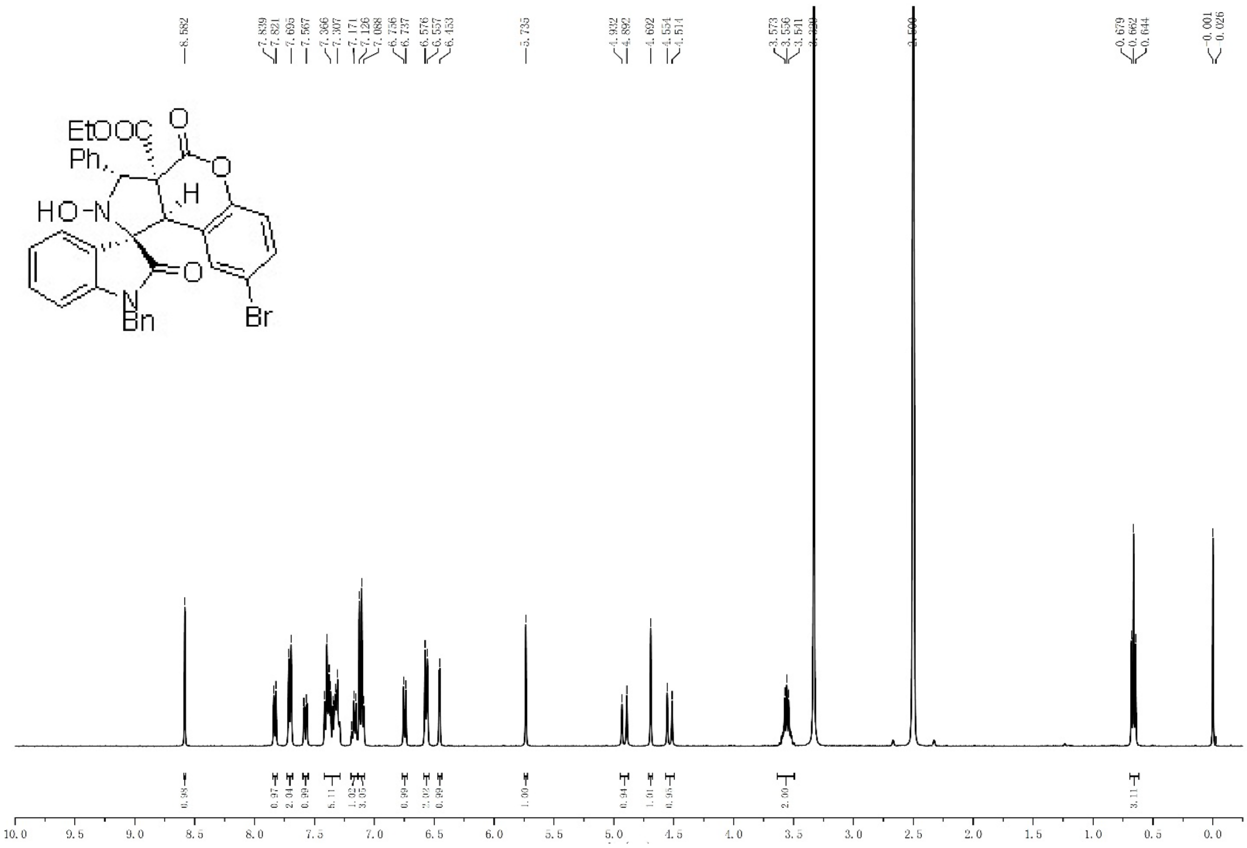

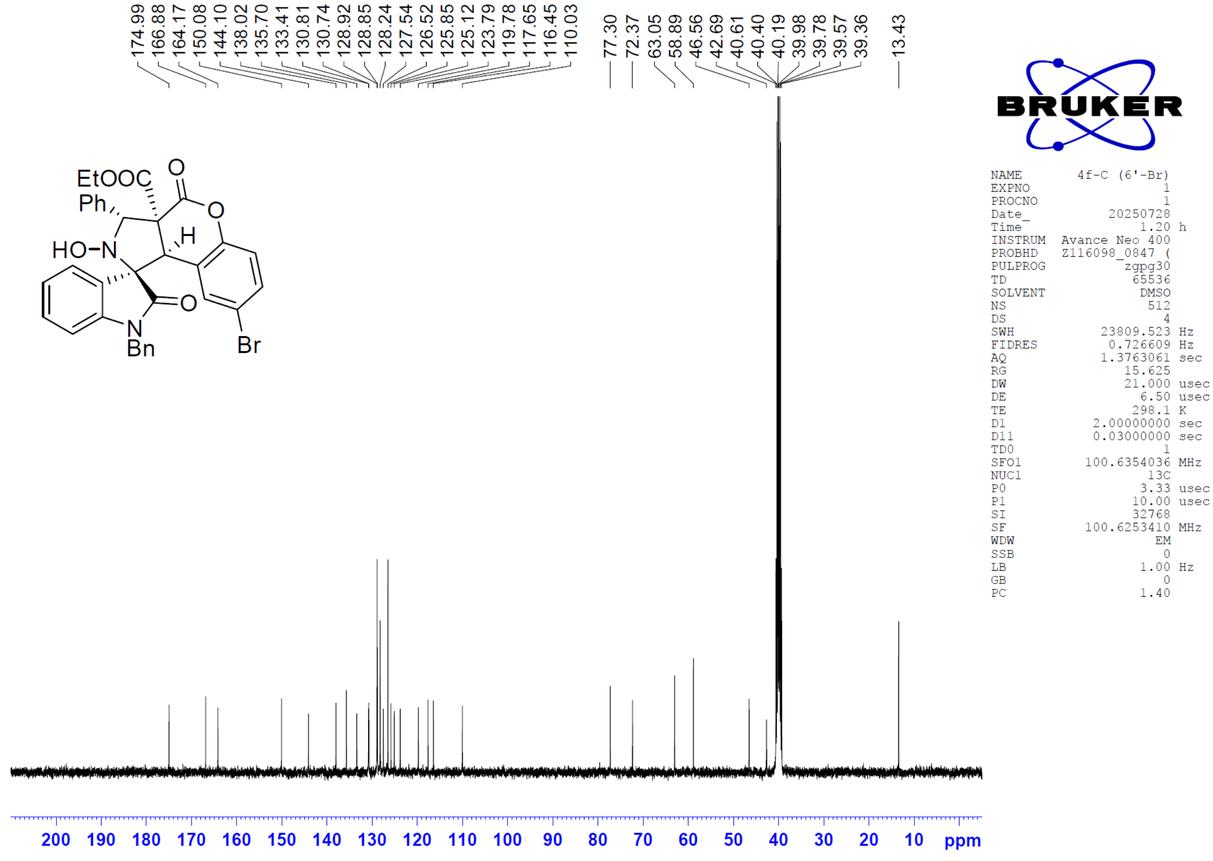

^{1}H NMR and ^{13}C NMR Spectra for Compound **4g**

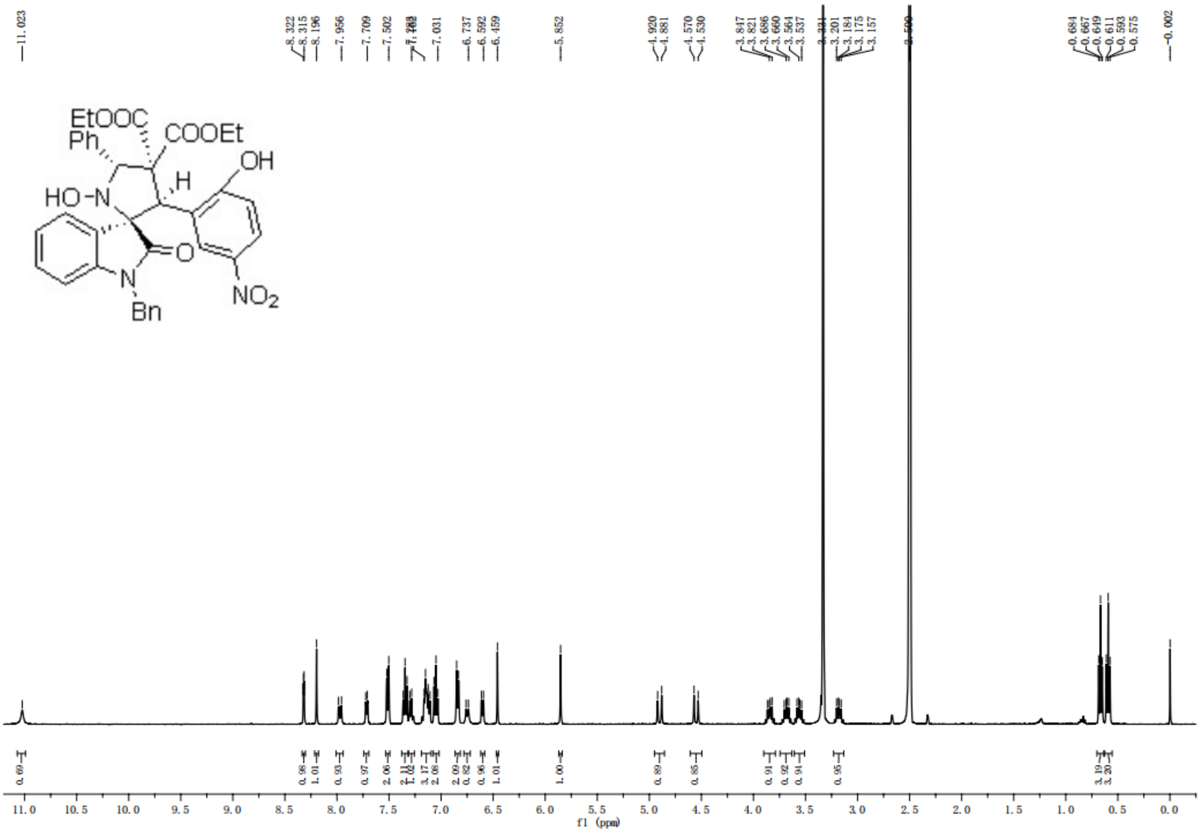

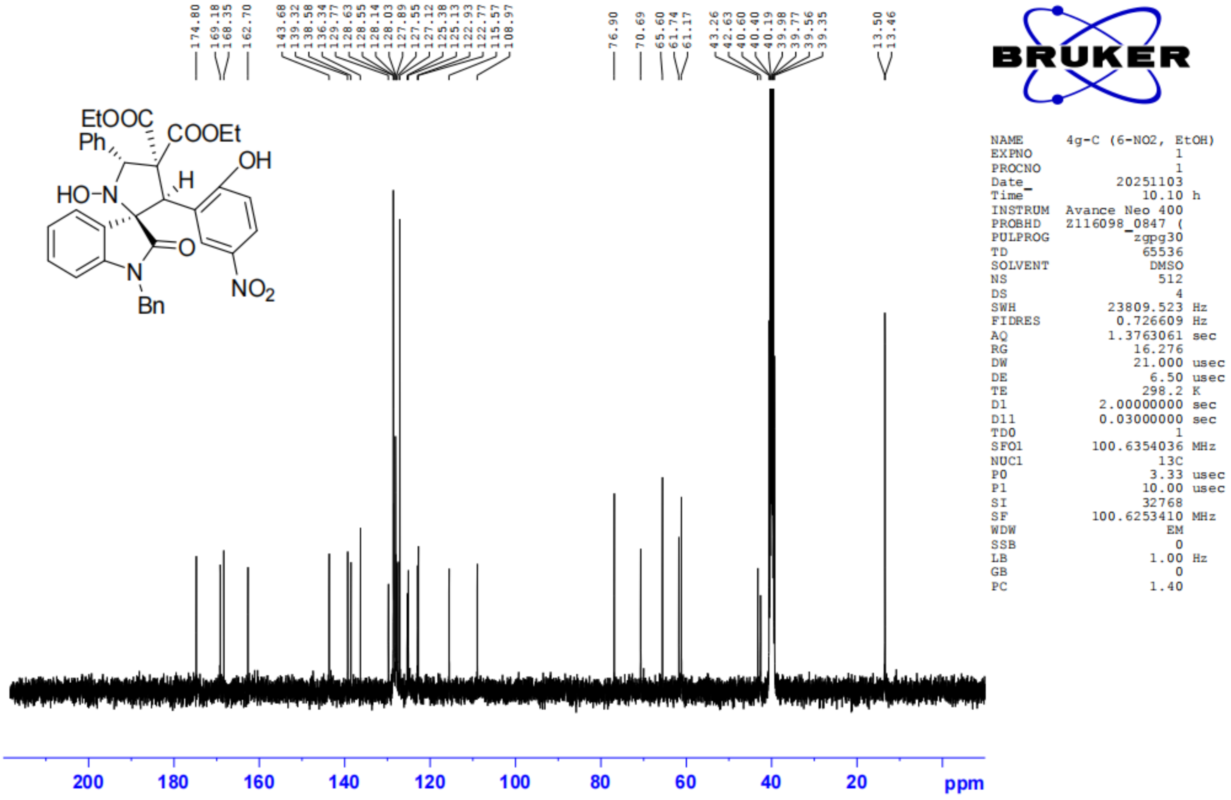

^{1}H NMR and ^{13}C NMR Spectra for Compound **4g′**

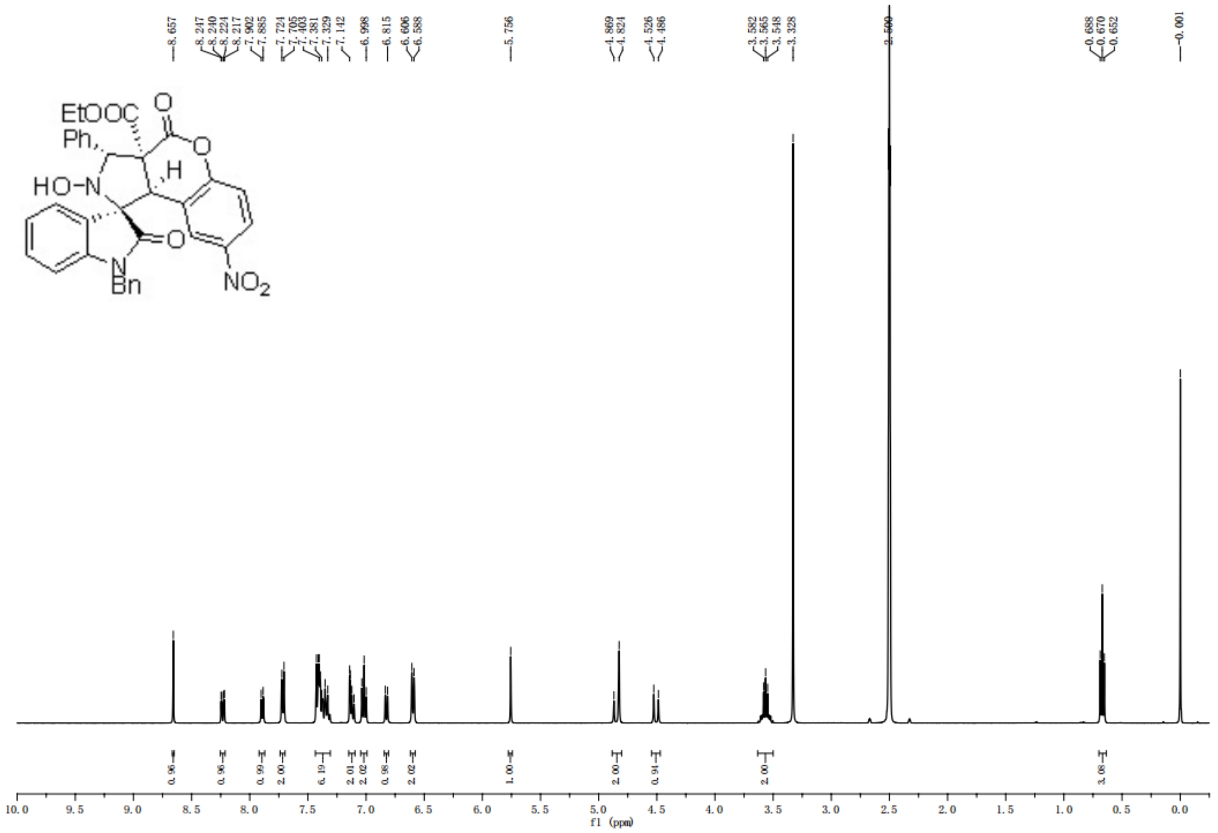

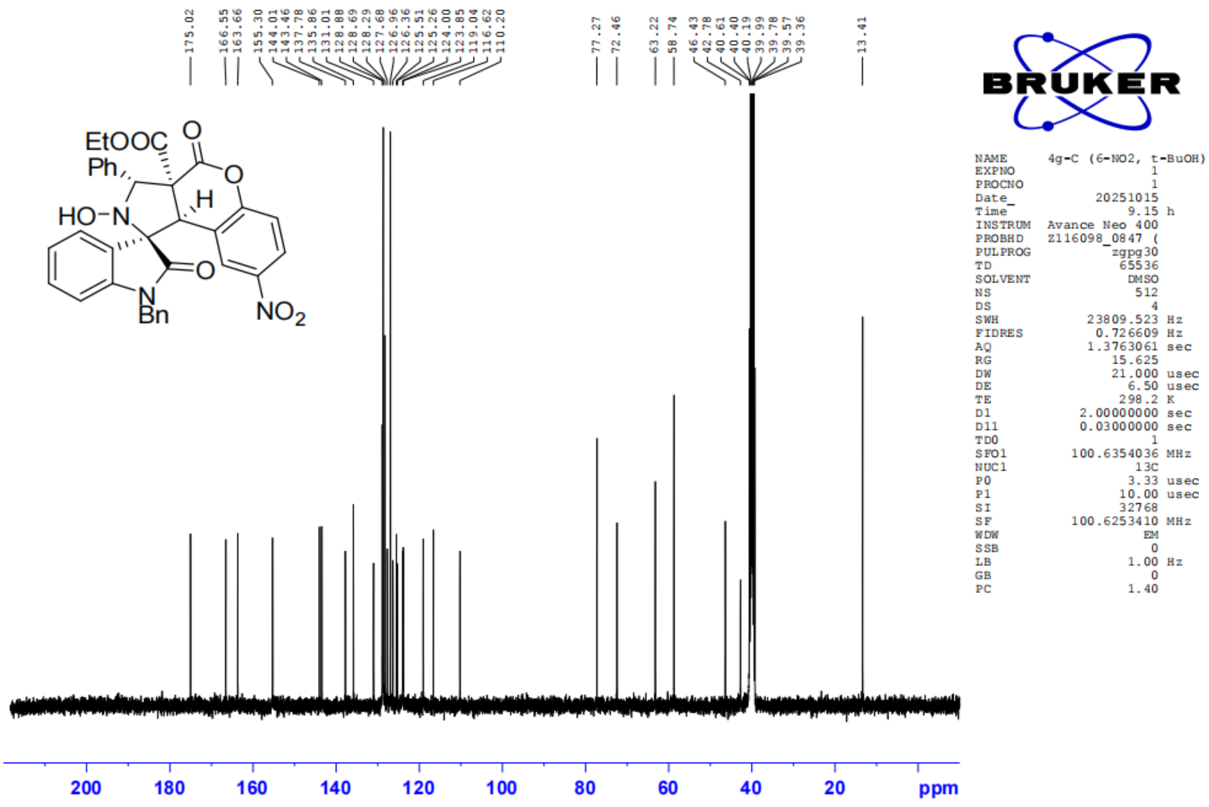

^{1}H NMR and ^{13}C NMR Spectra for Compound **4h**

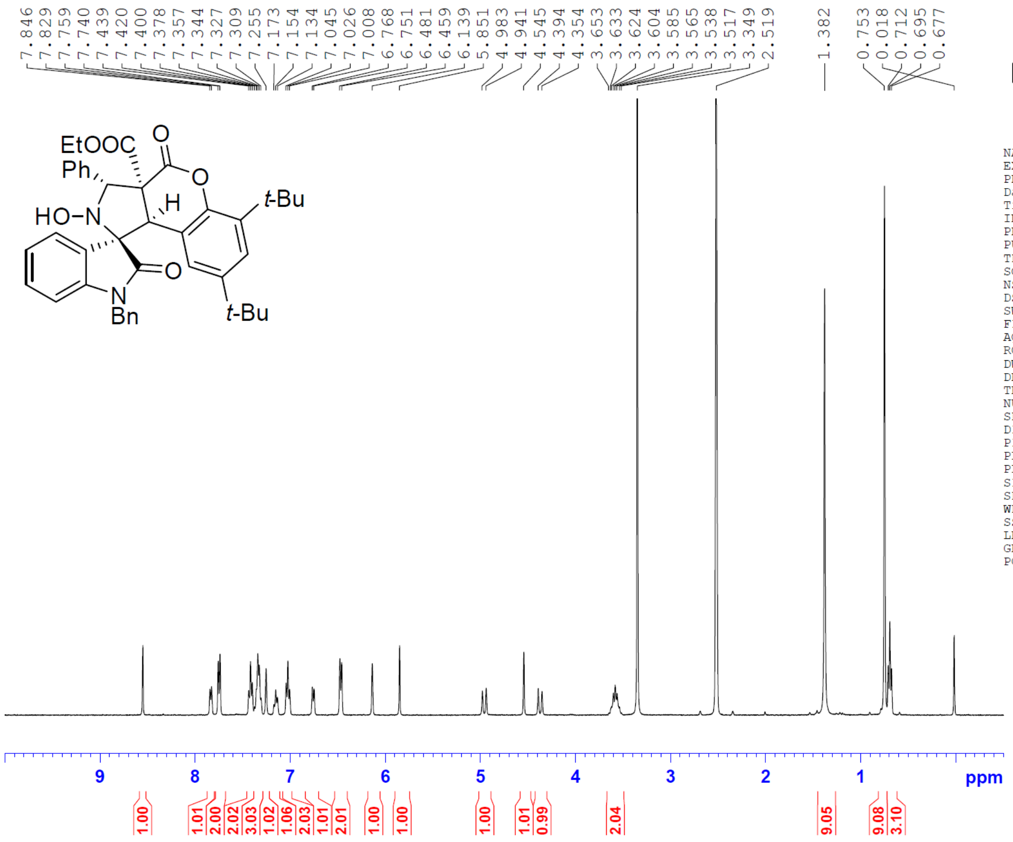

NAME	4h-H (6',8'-tBu2)
EXPNO	1
PROCNO	1
Date_	20250627
Time	10.35
INSTRUM	QUANTUM-I
PROBHD	probeinfo
PULPROG	s1pul30
TD	64100
SOLVENT	DMSO
NS	16
DS	2
SWH	8012.000 Hz
FIDRES	0.124992 Hz
AQ	4.0002995 sec
RG	61.98
DW	62.406 usec
DE	30.00 usec
TE	273.1 K
NUC1	1H
SFO1	399.8242529 MHz
D1	1.00000000 sec
P1	12.00 usec
PL1	44.00 dB
PL1W	0.00040000 W
SI	65536
SF	399.8218539 MHz
WDW	EM
SSB	0
LB	0.30 Hz
GB	0
PC	4.00

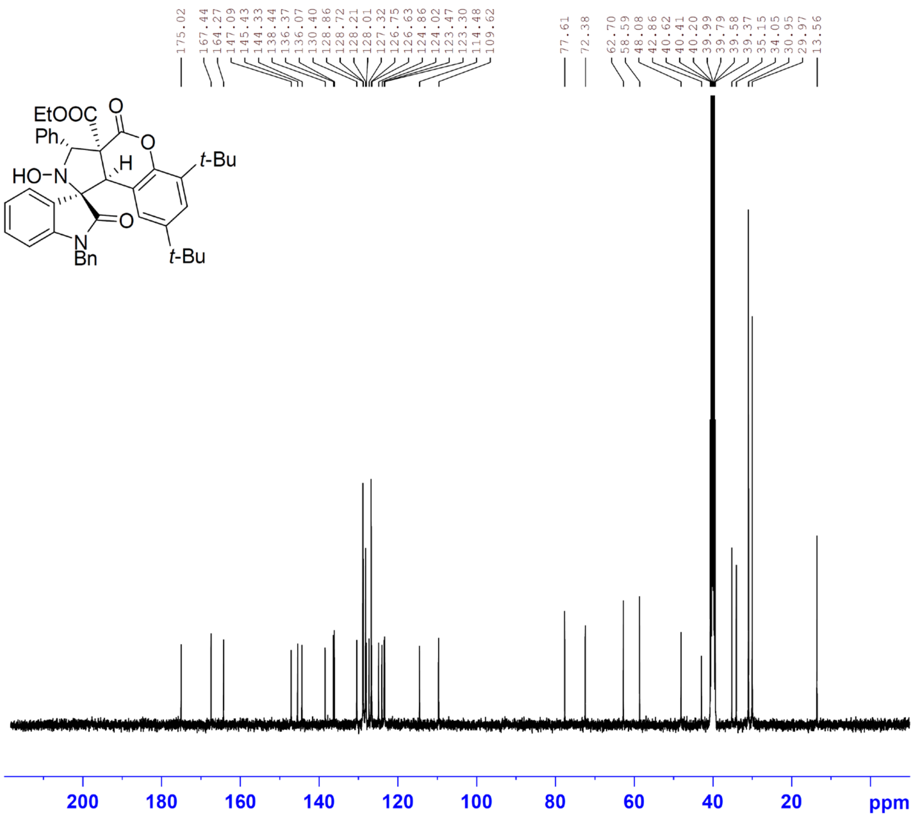

NAME	4h-C
EXPNO	1
PROCNO	1
Date_	20260128
Time	17.20 h
INSTRUM	Avance Neo 400
PROBHD	Z116098_0847 (
PULPROG	zgpg30
TD	65536
SOLVENT	DMSO
NS	1024
DS	4
SWH	23809.523 Hz
FIDRES	0.726609 Hz
AQ	1.3763061 sec
RG	15.625
DW	21.000 usec
DE	6.50 usec
TE	298.1 K
D1	2.00000000 sec
D11	0.03000000 sec
TD0	1
SFO1	100.6354036 MHz
NUC1	13C
P0	3.33 usec
P1	10.00 usec
SI	32768
SF	100.6253410 MHz
WDW	EM
SSB	0
LB	1.00 Hz
GB	0
PC	1.40

^{1}H NMR and ^{13}C NMR Spectra for Compound **4j**

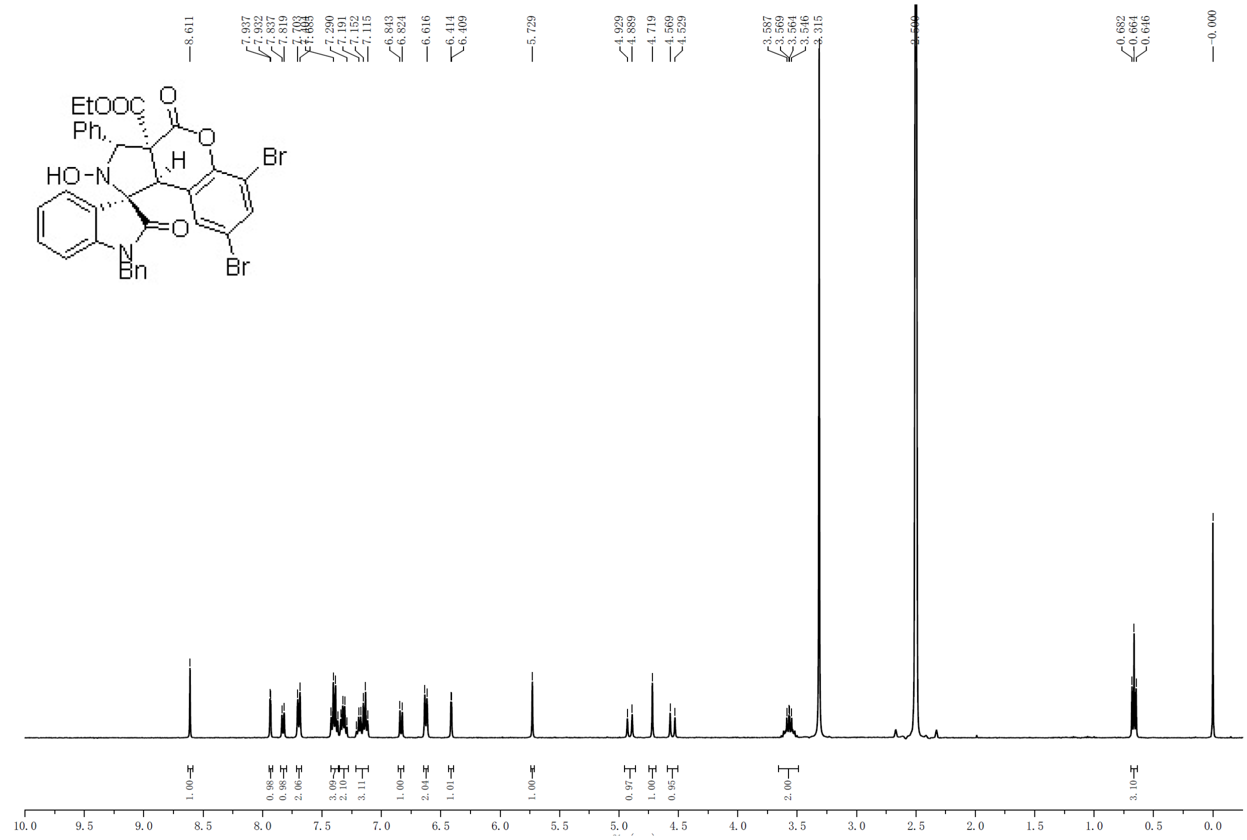

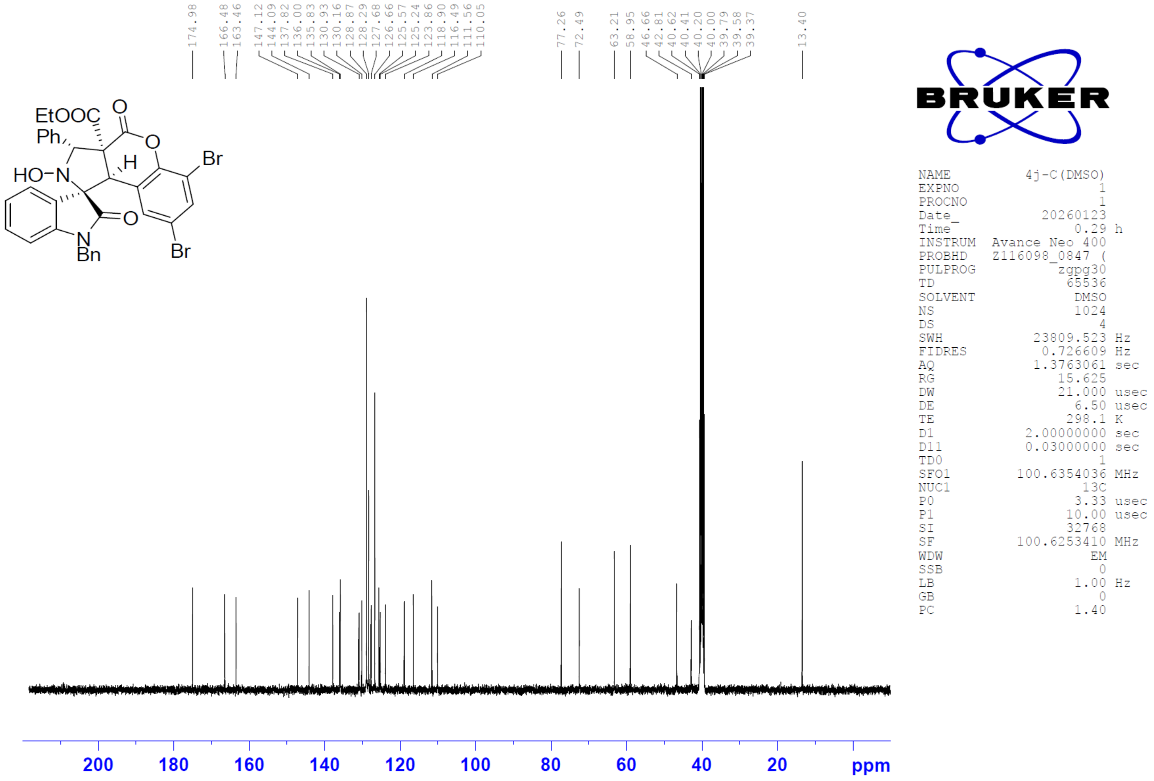

^{1}H NMR and ^{13}C NMR Spectra for Compound **4k**

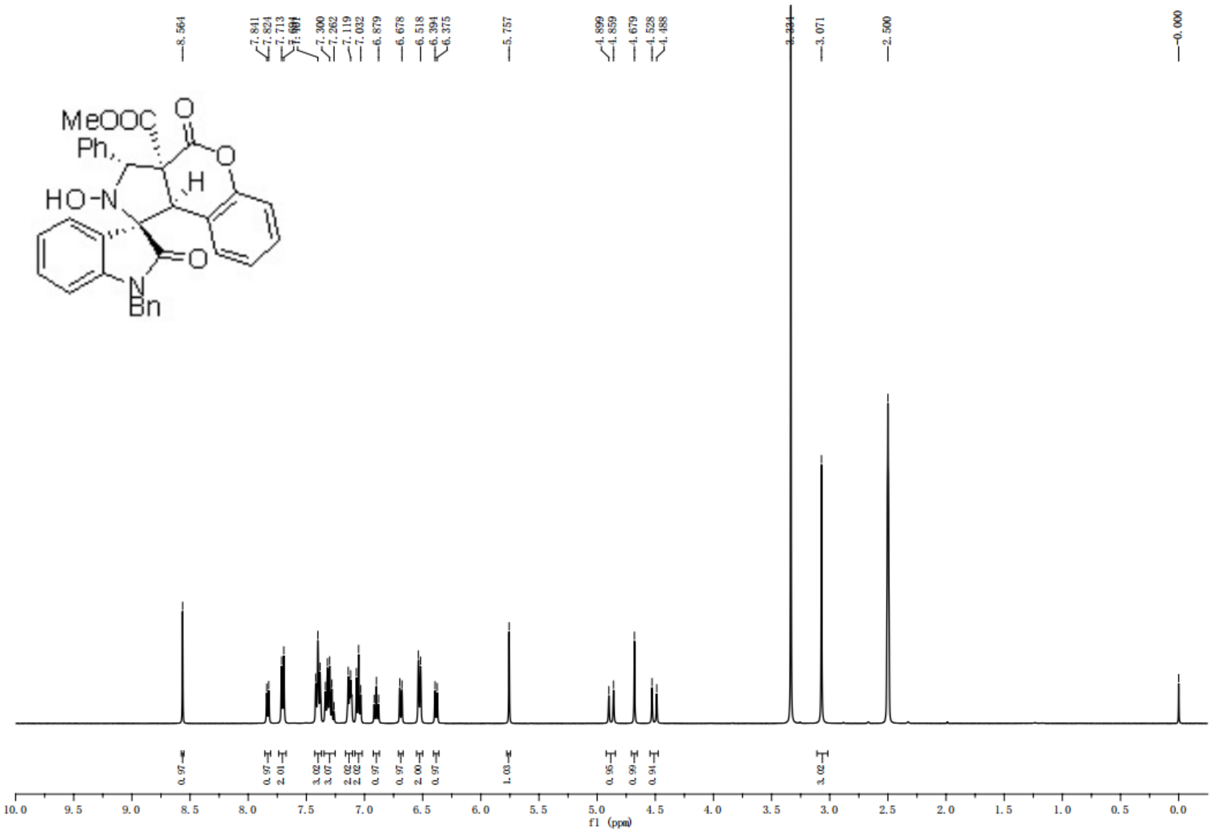

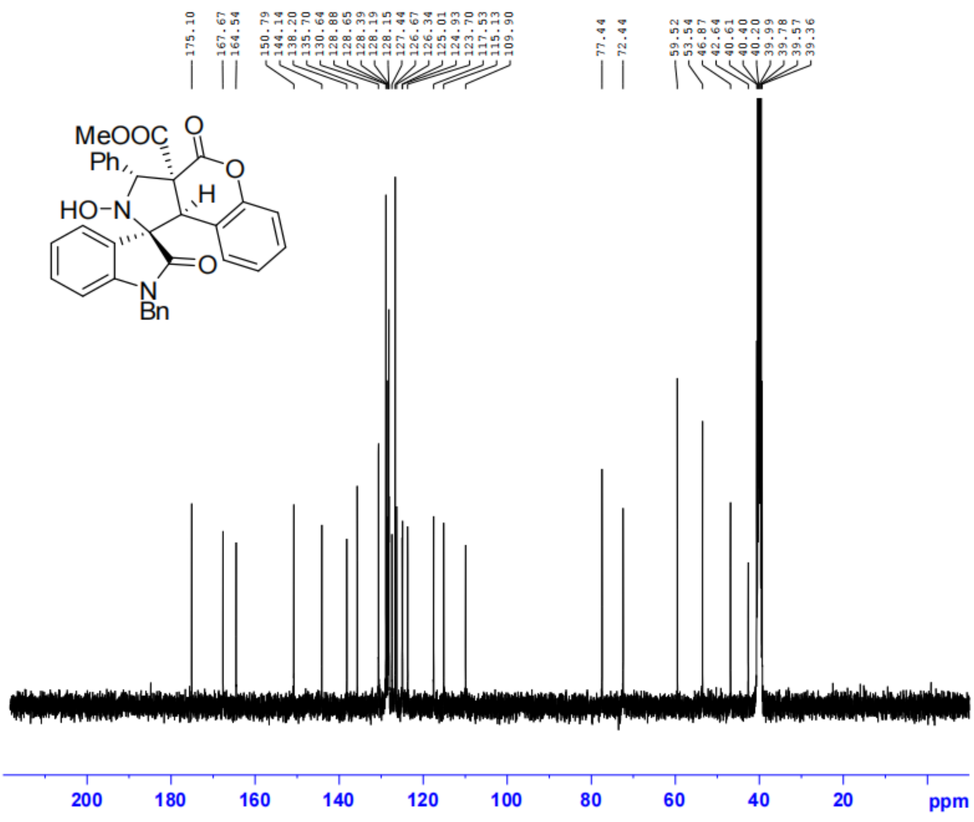

NAME	4k-C (COOMe)
EXPNO	1
PROCNO	1
Date_	20250929
Time	18.48 h
INSTRUM	Avance Neo 400
PROBHD	Z116098_0847 (
PULPROG	zgpg30
TD	65536
SOLVENT	DMSO
NS	512
DS	4
SWH	23809.523 Hz
FIDRES	0.726609 Hz
AQ	1.3763061 sec
RG	19.9298
DW	21.000 usec
DE	6.50 usec
TE	298.2 K
D1	2.00000000 sec
D11	0.03000000 sec
TD0	1
SFO1	100.6354036 MHz
NUC1	13C
P0	3.33 usec
P1	10.00 usec
SI	32768
SF	100.6253410 MHz
WDW	EM
SSB	0
LB	1.00 Hz
GB	0
PC	1.40

^{1}H NMR and ^{13}C NMR Spectra for Compound **4l**

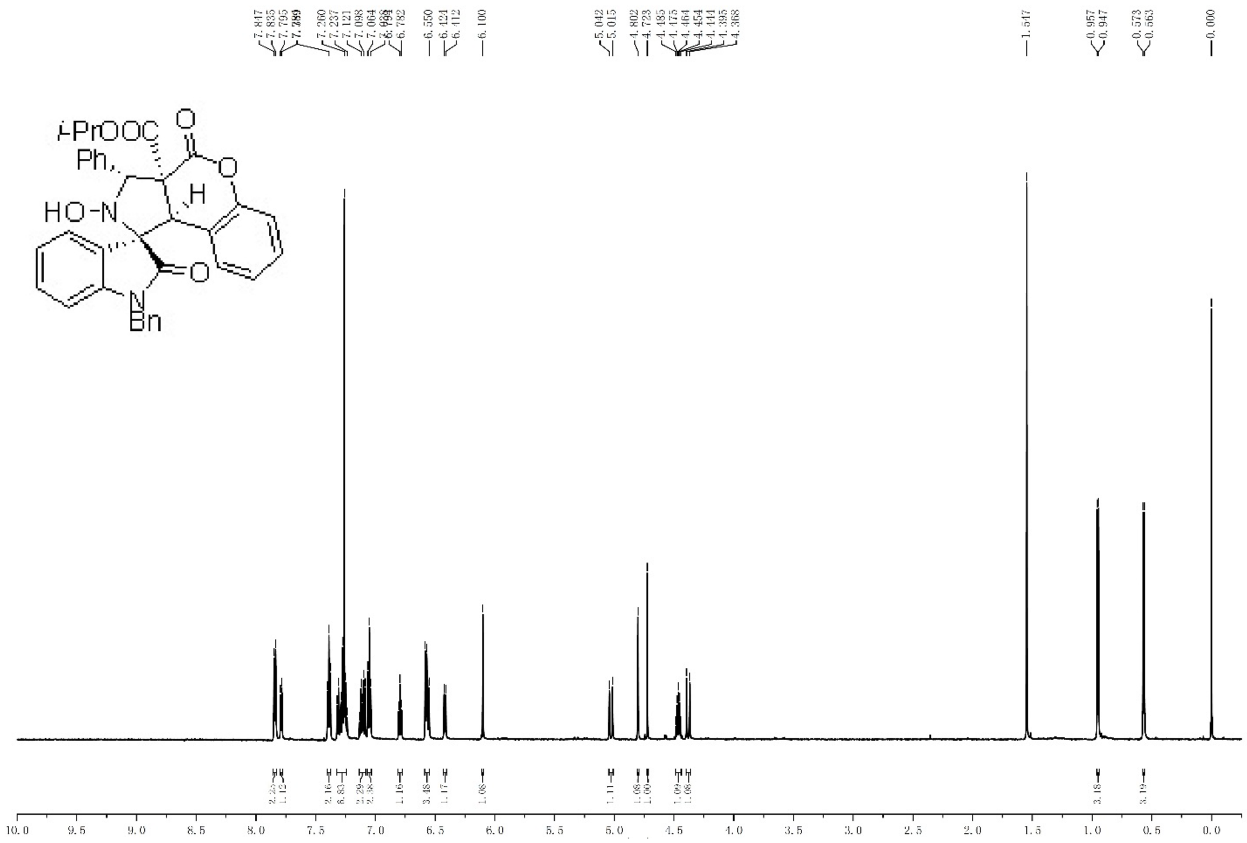

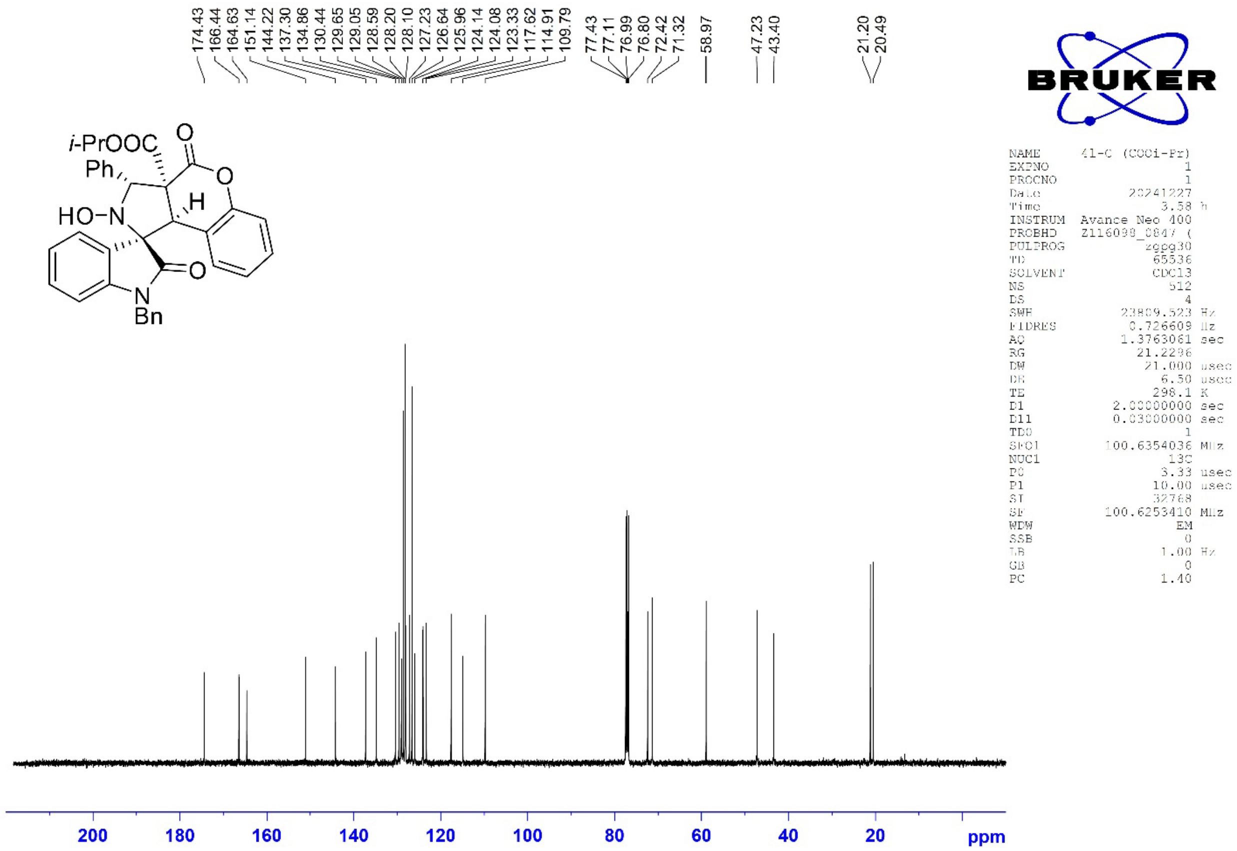

^{1}H NMR and ^{13}C NMR Spectra for Compound **4m**

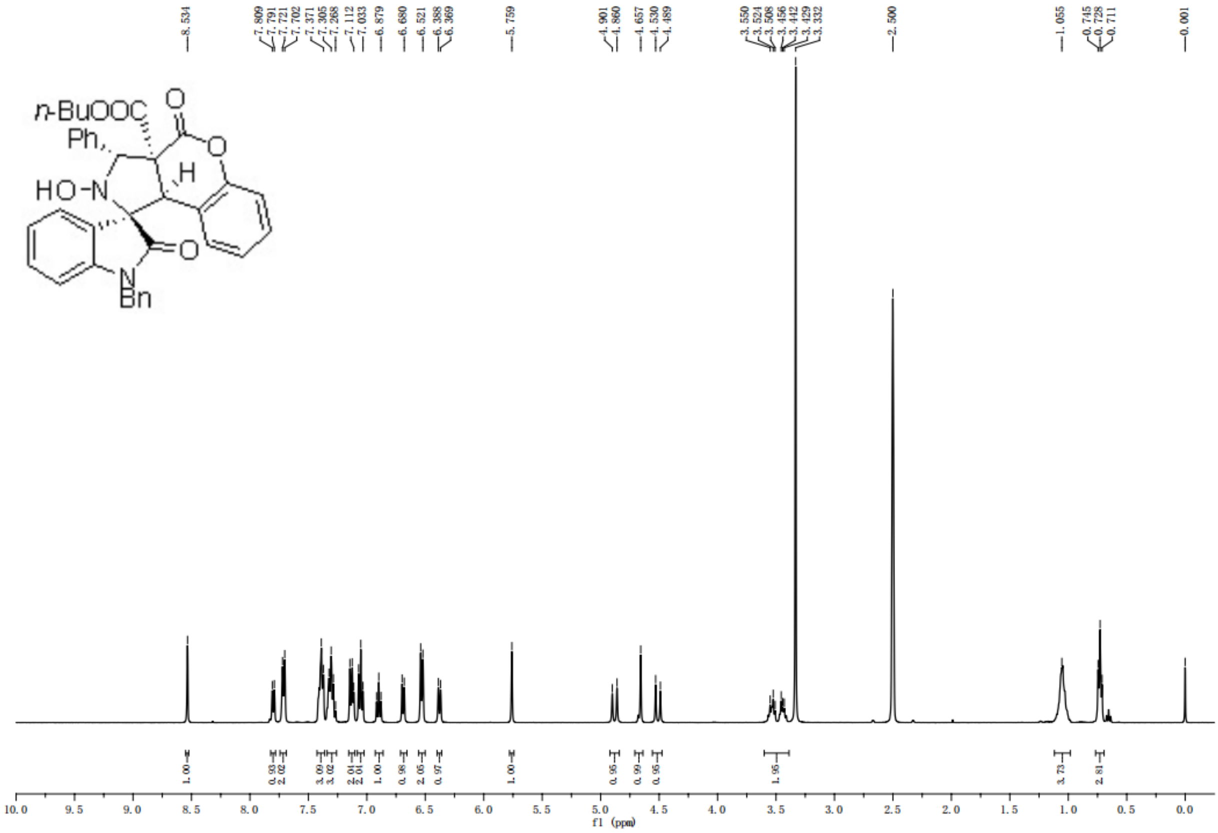

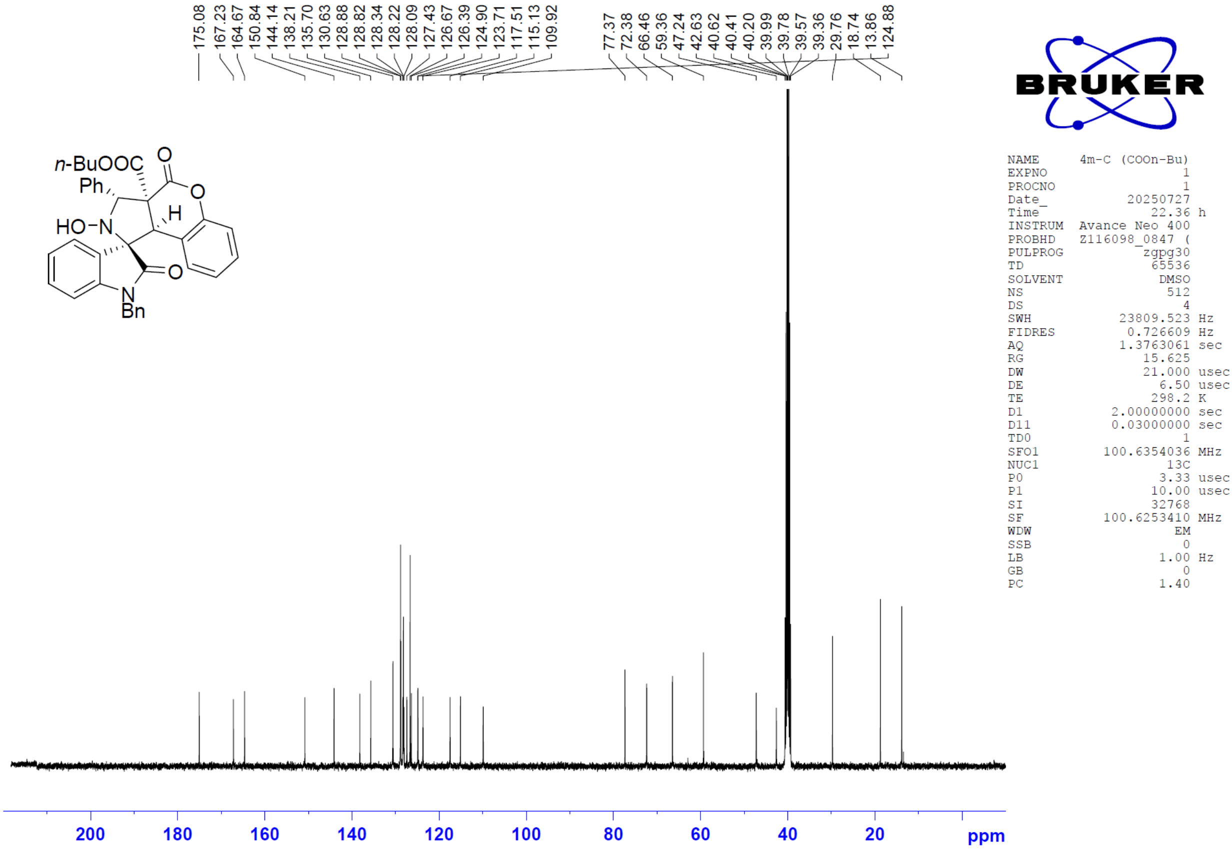

BRUKER

NAME	4m-C (COOn-Bu)
EXPNO	1
PROCNO	1
Date_	20250727
Time	22.36 h
INSTRUM	Avance Neo 400
PROBHD	Z116098_0847 (
PULPROG	zgpg30
TD	65536
SOLVENT	DMSO
NS	512
DS	4
SWH	23809.523 Hz
FIDRES	0.726609 Hz
AQ	1.3763061 sec
RG	15.625
DW	21.000 usec
DE	6.50 usec
TE	298.2 K
D1	2.00000000 sec
D11	0.03000000 sec
TD0	1
SFO1	100.6354036 MHz
NUC1	13C
P0	3.33 usec
P1	10.00 usec
SI	32768
SF	100.6253410 MHz
WDW	EM
SSB	0
LB	1.00 Hz
GB	0
PC	1.40

^{1}H NMR and ^{13}C NMR Spectra for Compound **4n**

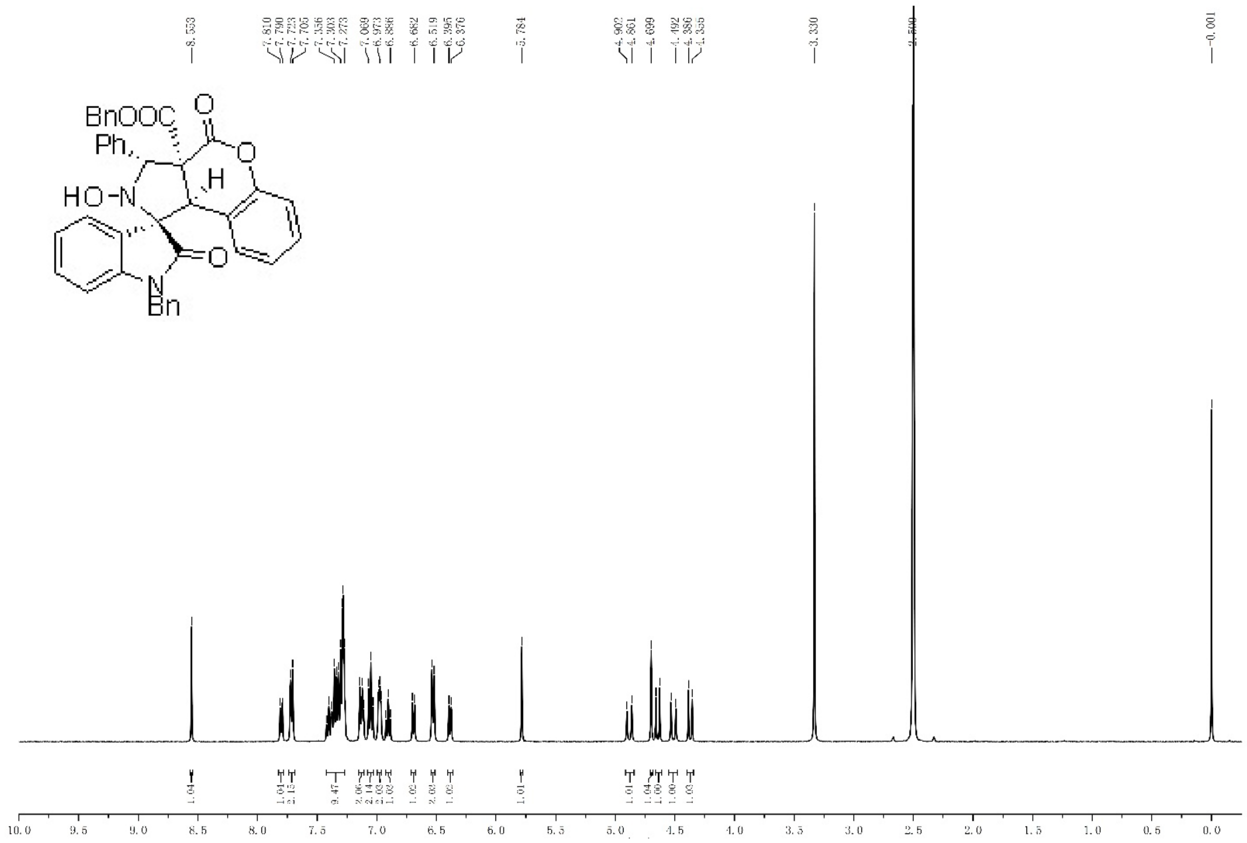

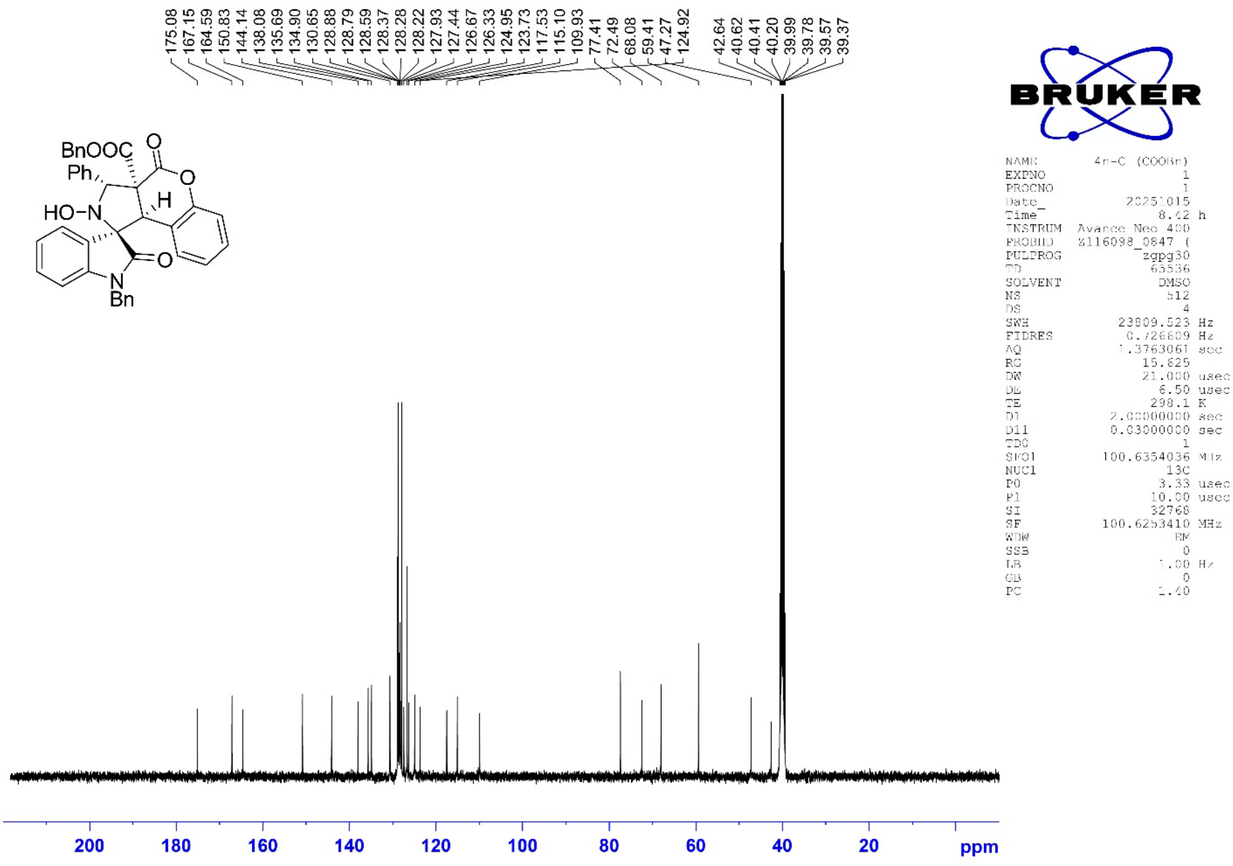

^{1}H NMR and ^{13}C NMR Spectra for Compound **6a**

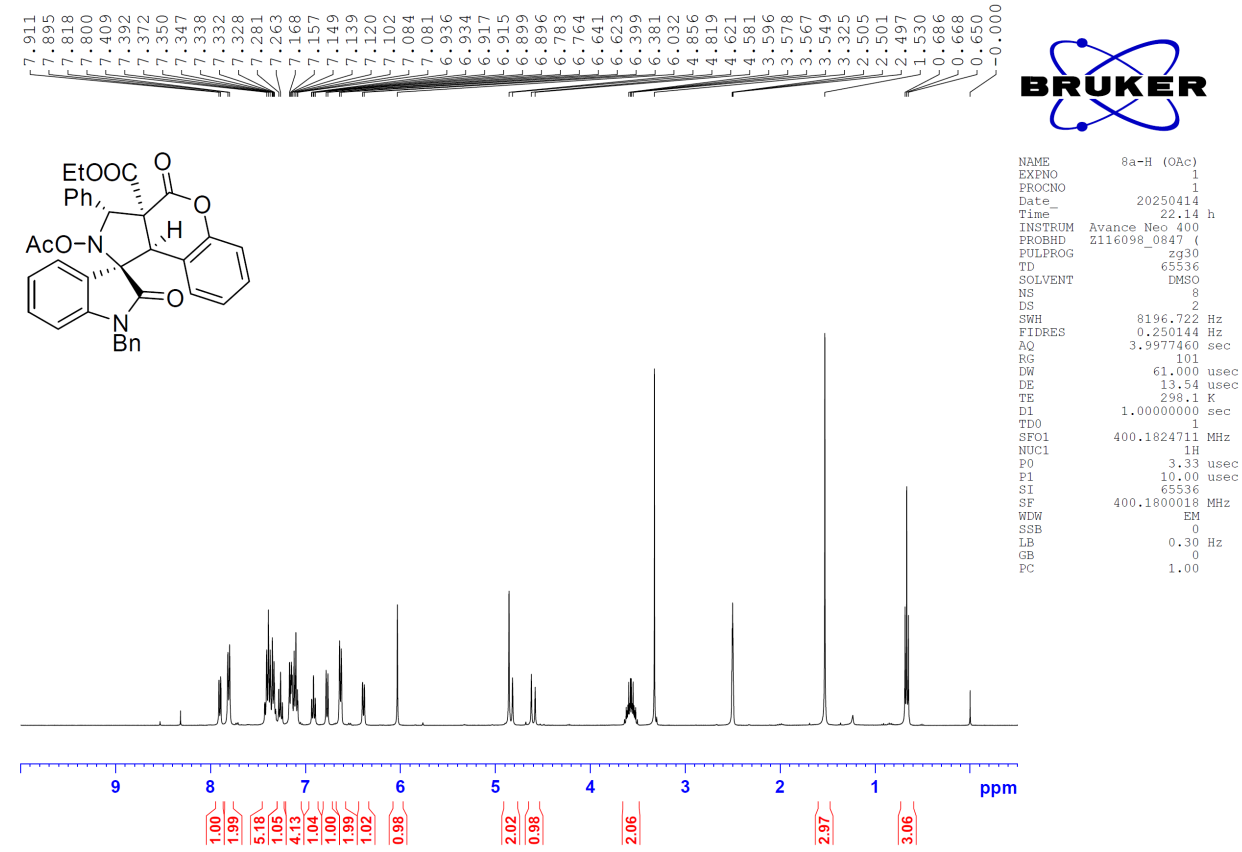

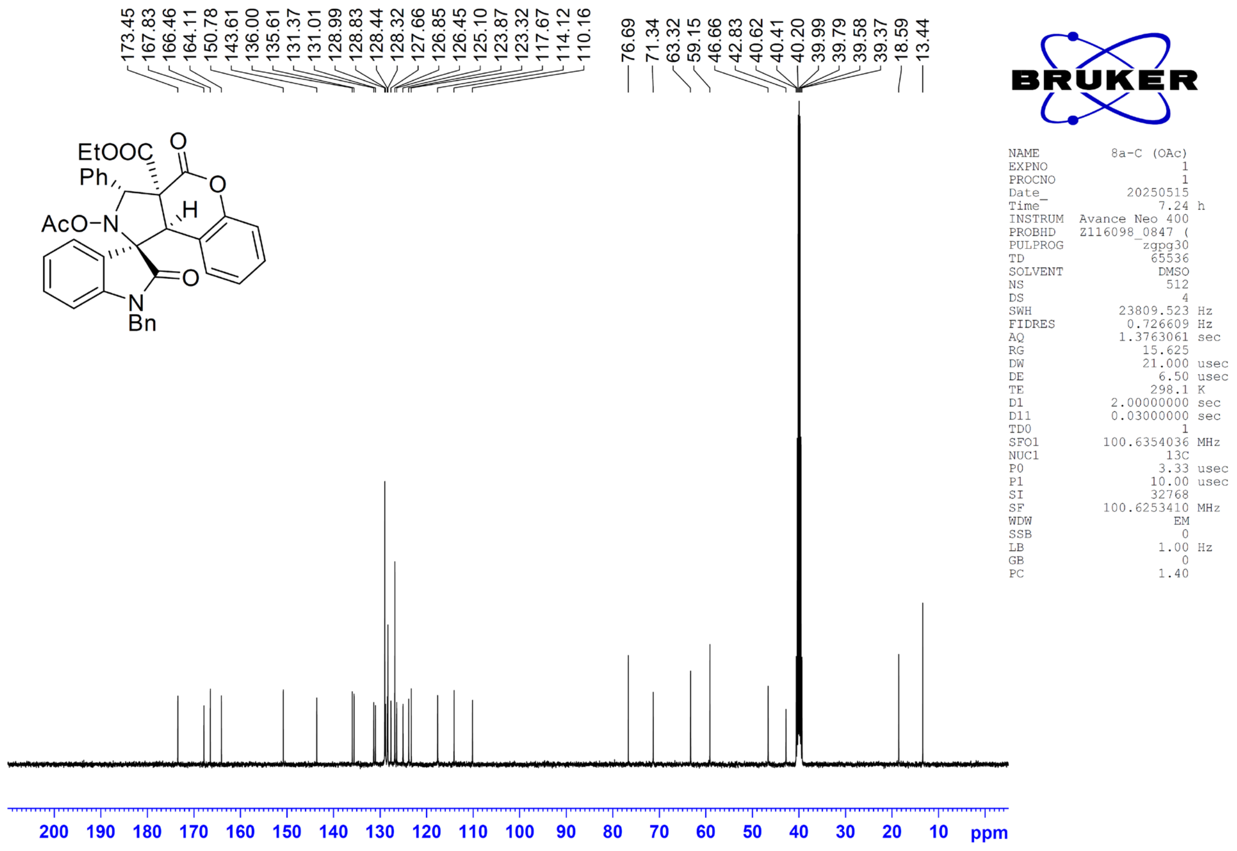

^{1}H NMR and ^{13}C NMR Spectra for Compound **6b**

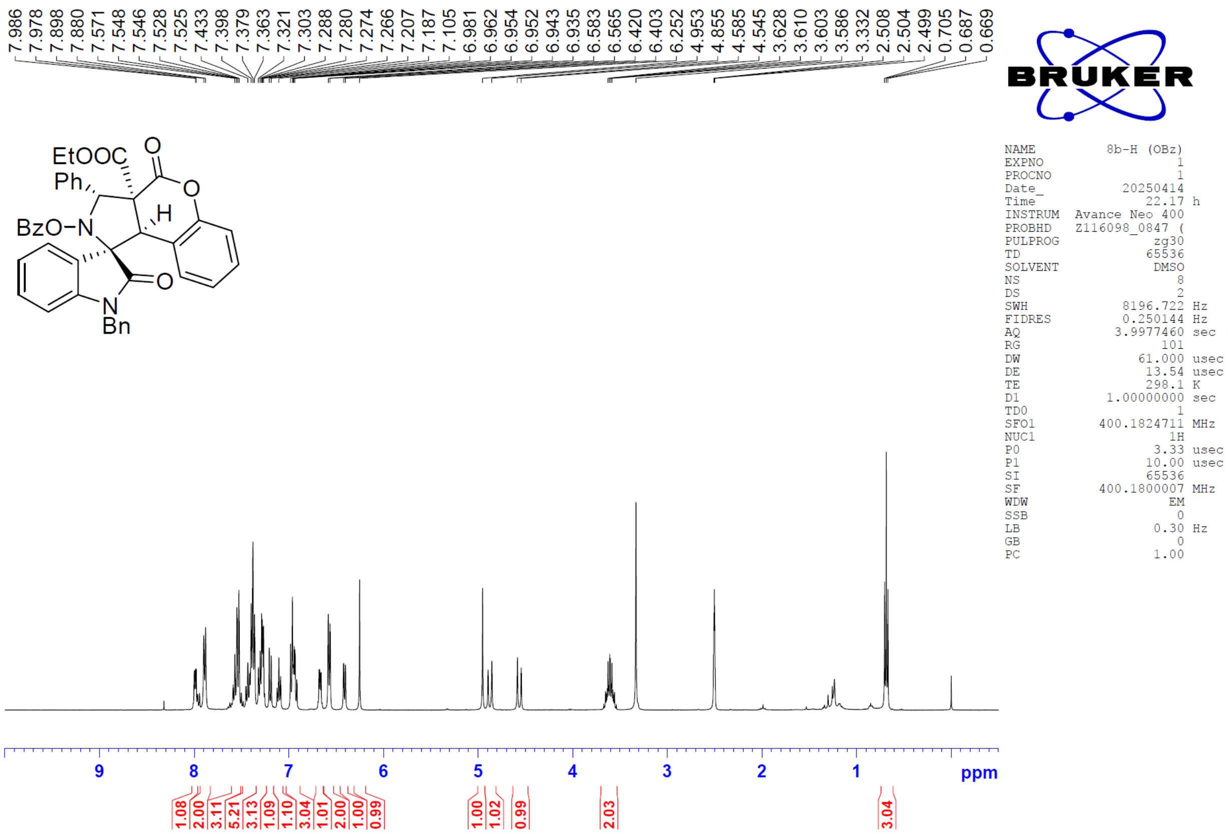

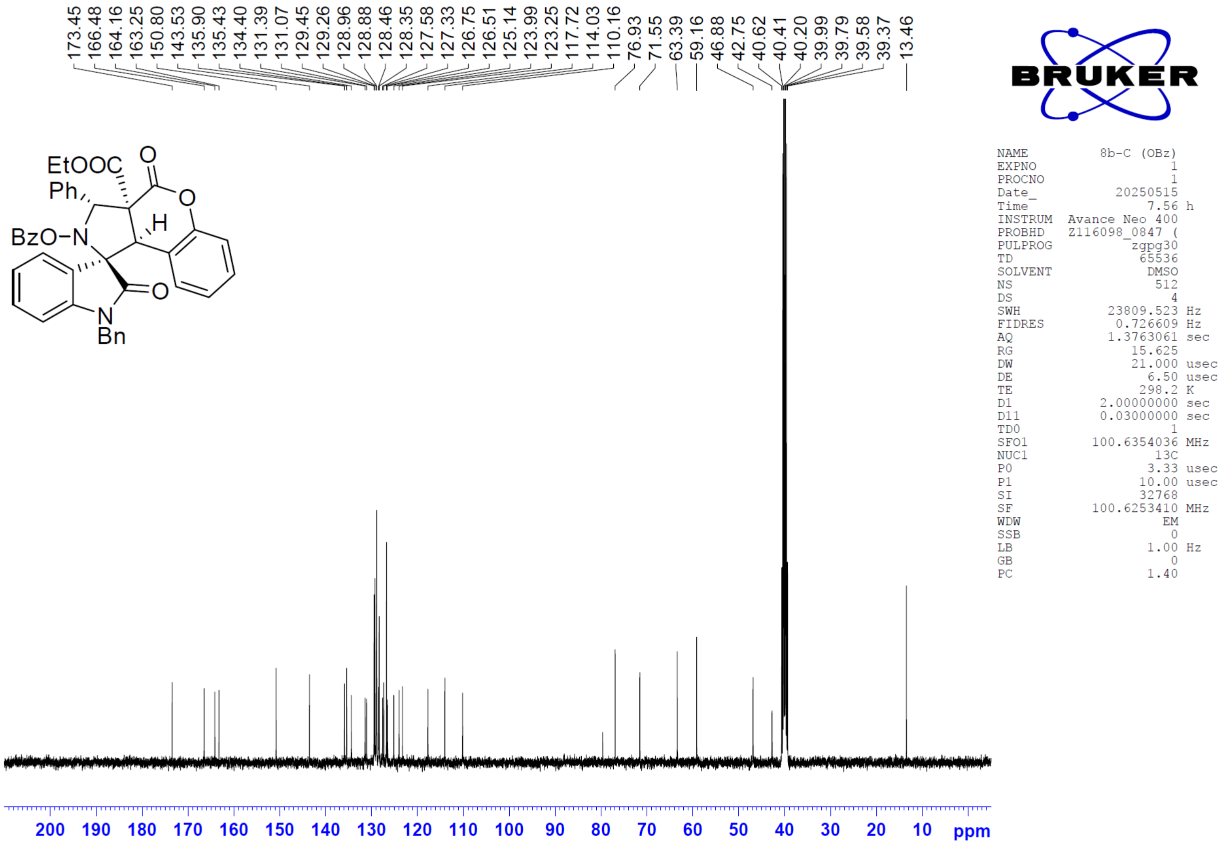

^{1}H NMR and ^{13}C NMR Spectra for Compound **8**

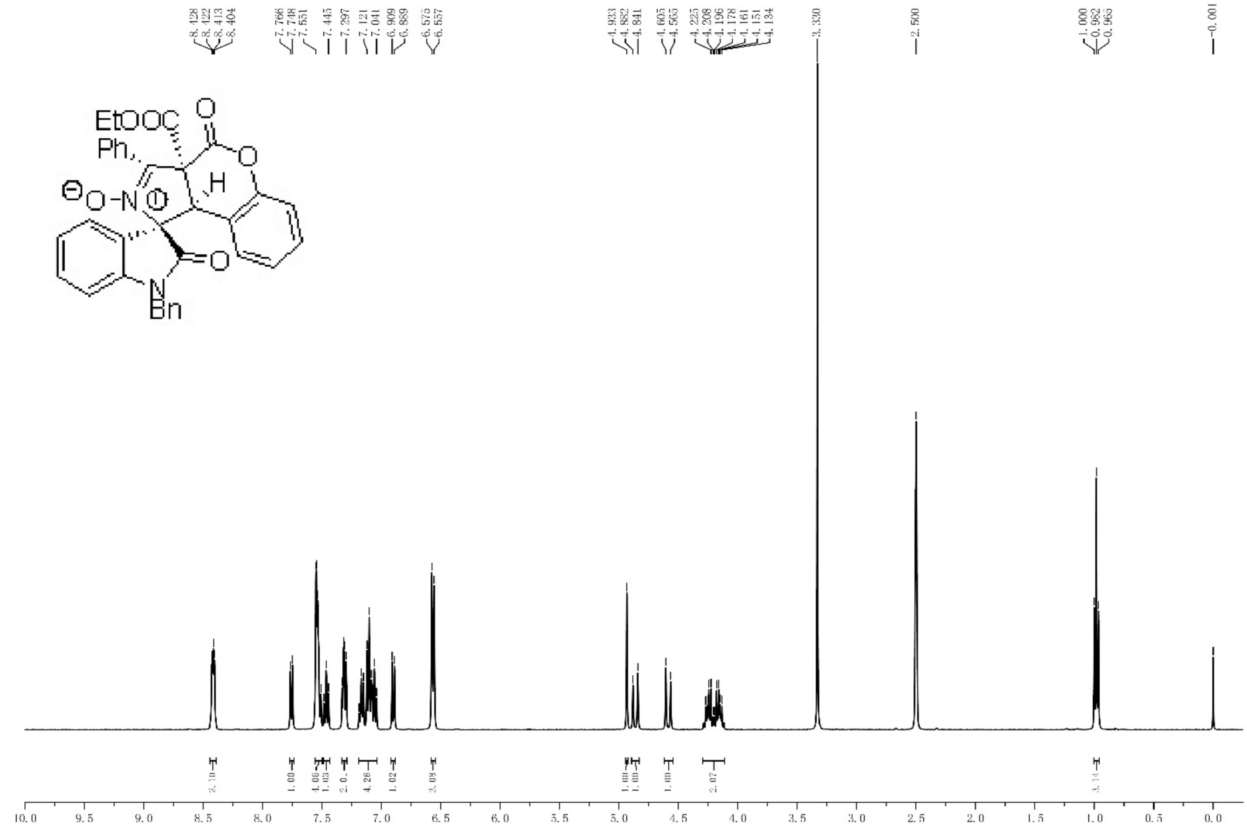

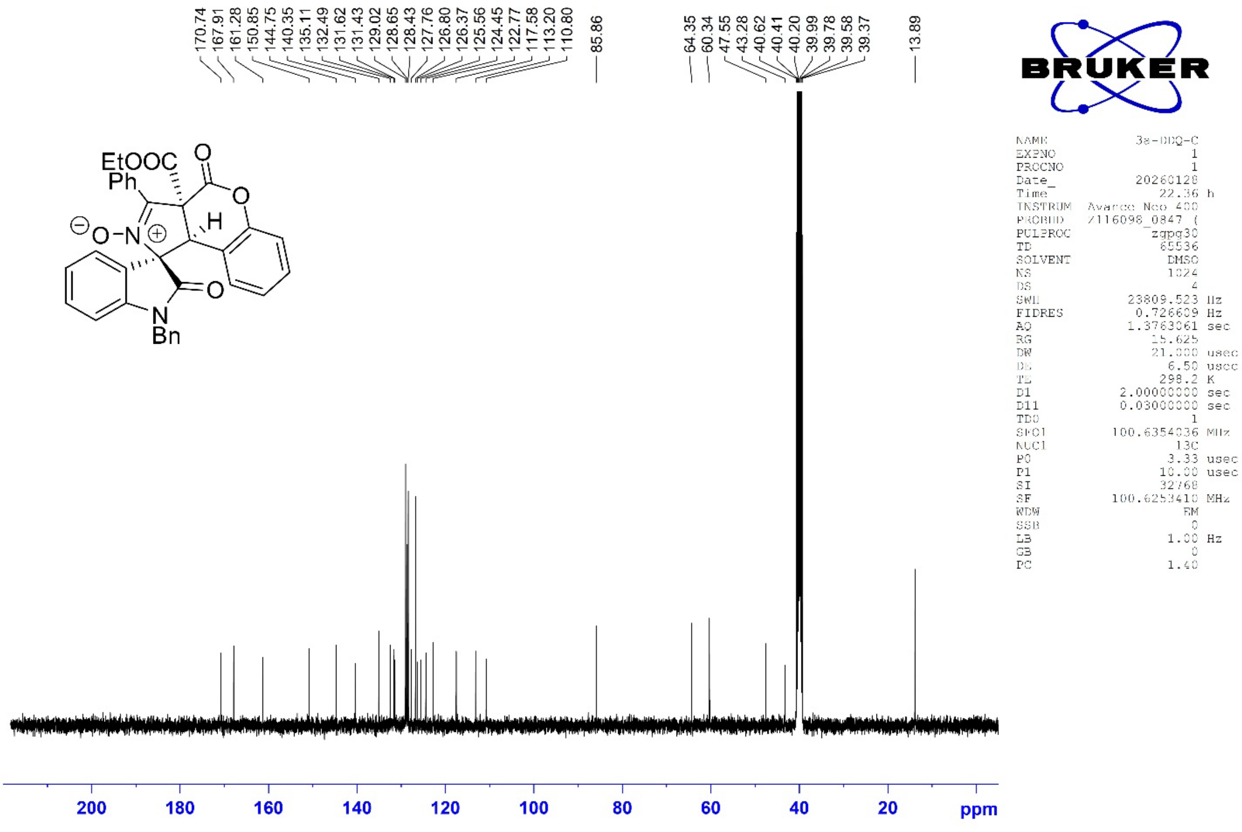

II. Copes of HRMS for all new compounds

HRMS for Compound **3a**

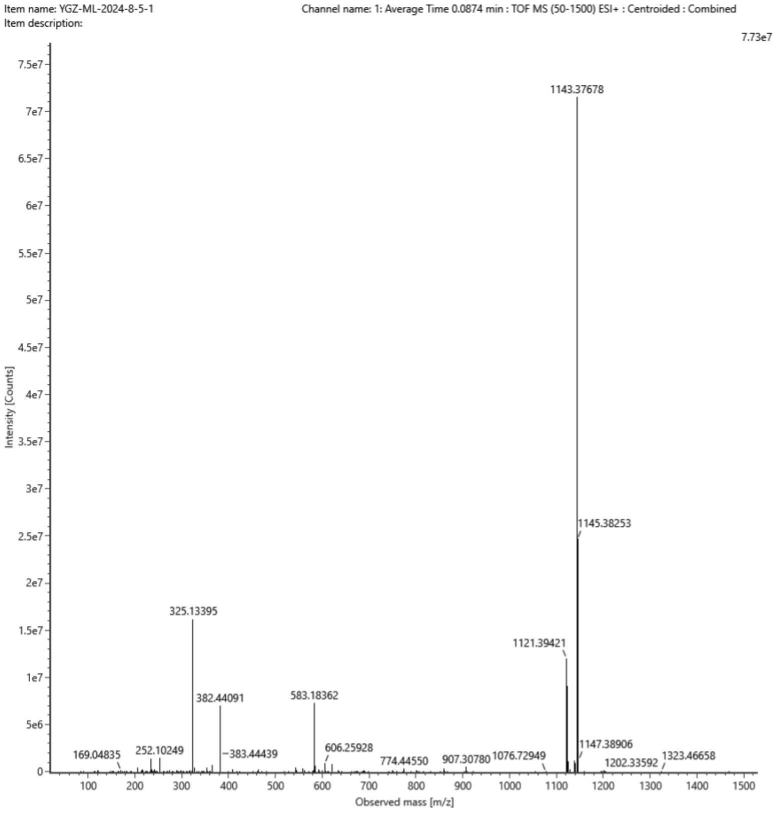

HRMS for Compound **3b**

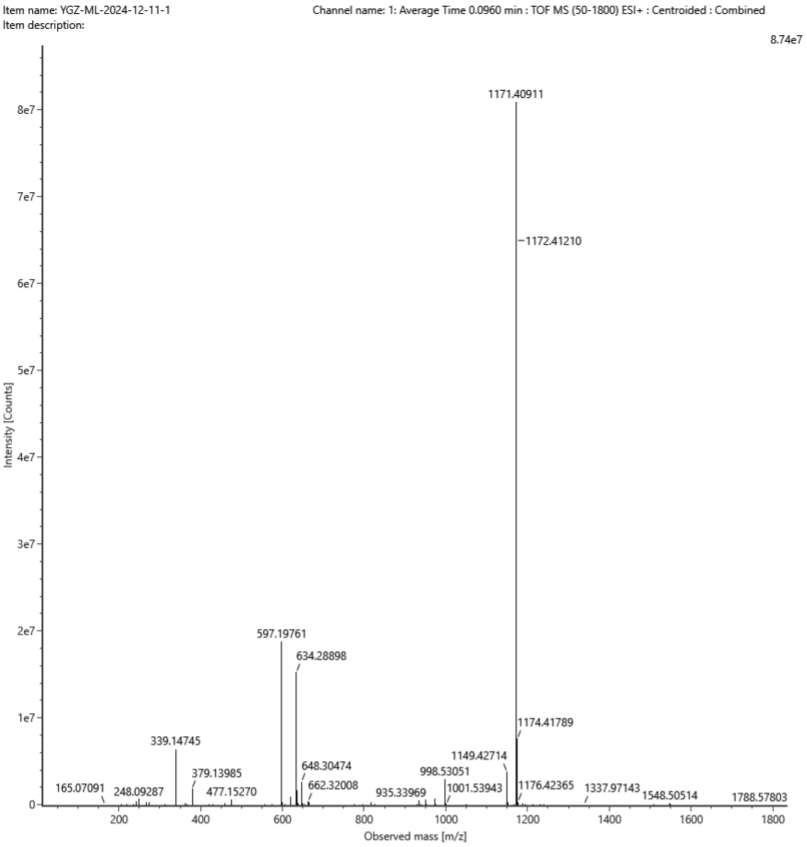

HRMS for Compound **3c**

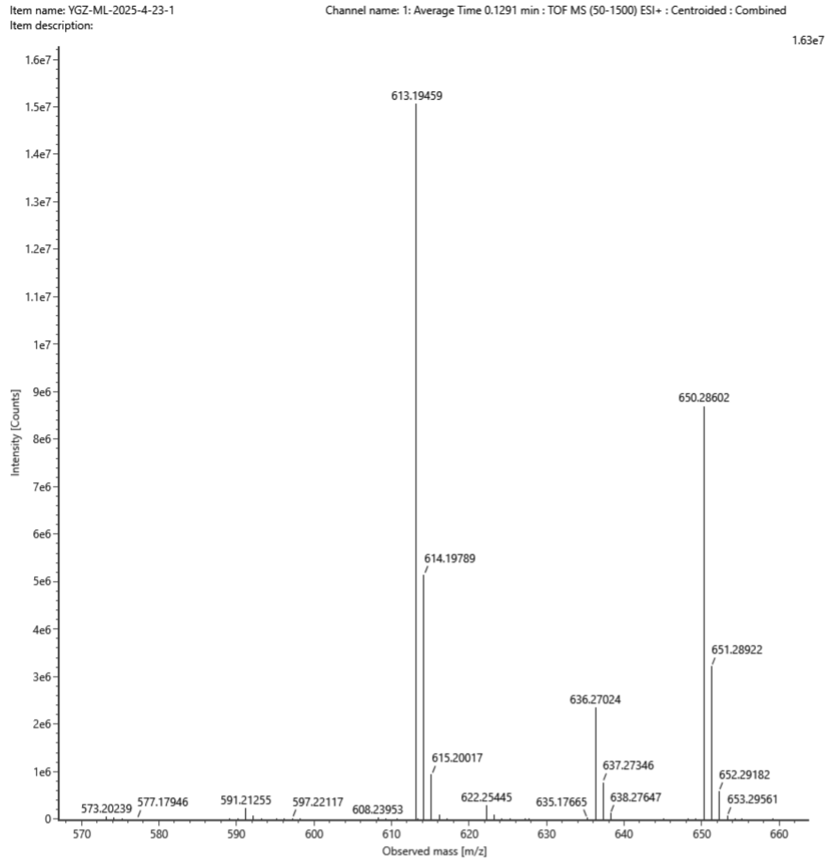

HRMS for Compound **3d**

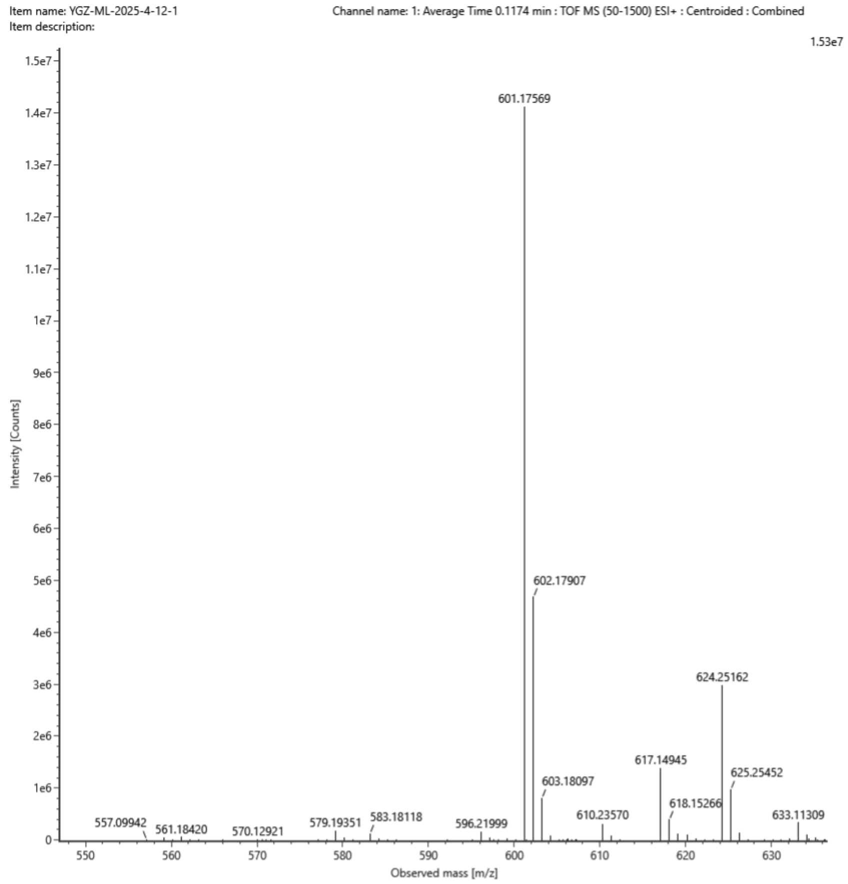

HRMS for Compound **3e**

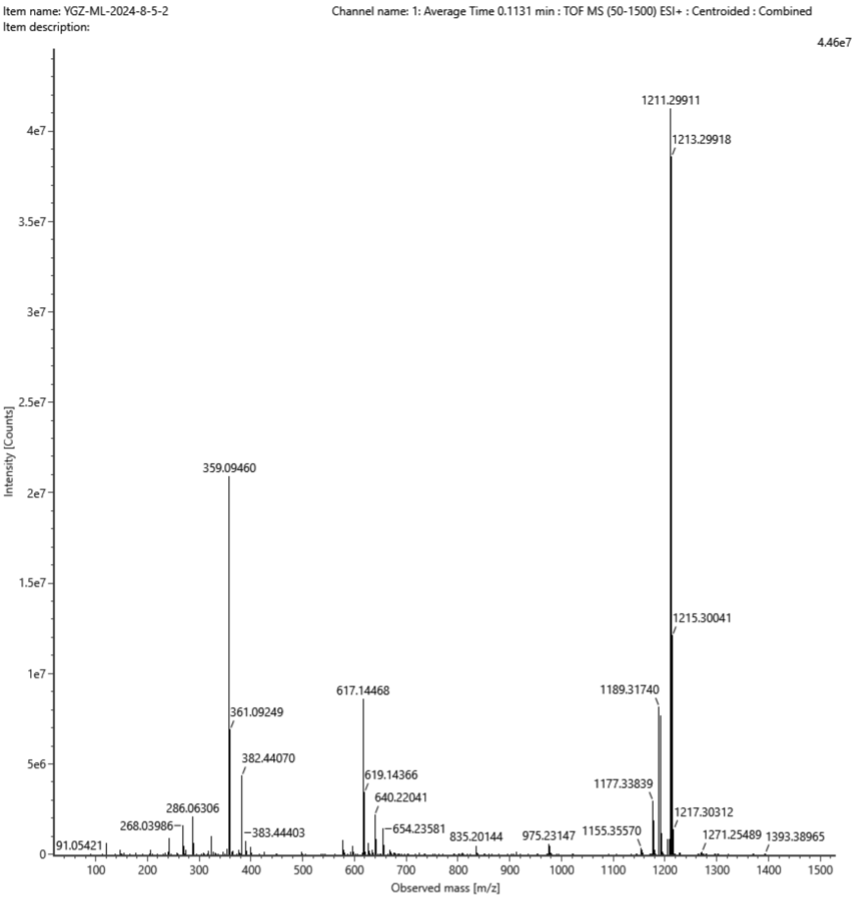

HRMS for Compound **3f**

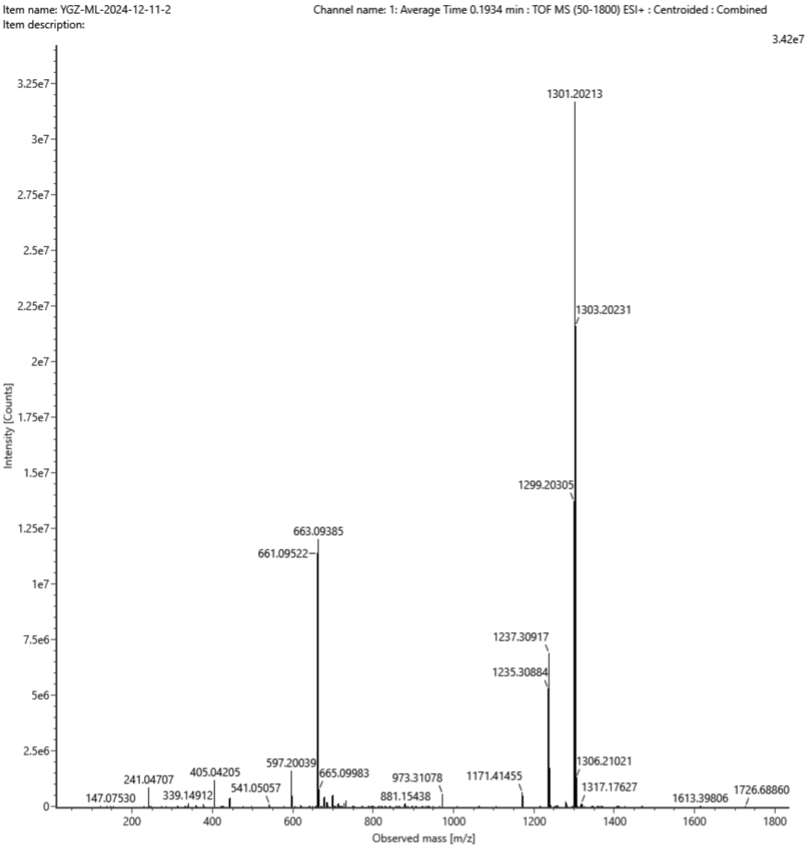

HRMS for Compound **3g**

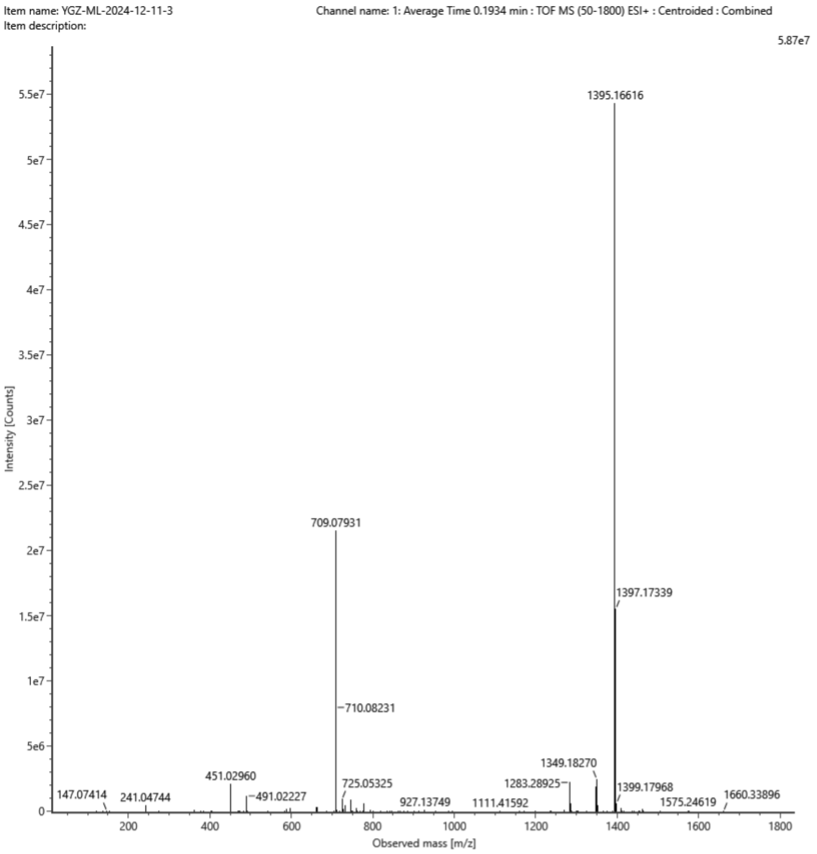

HRMS for Compound **3J**

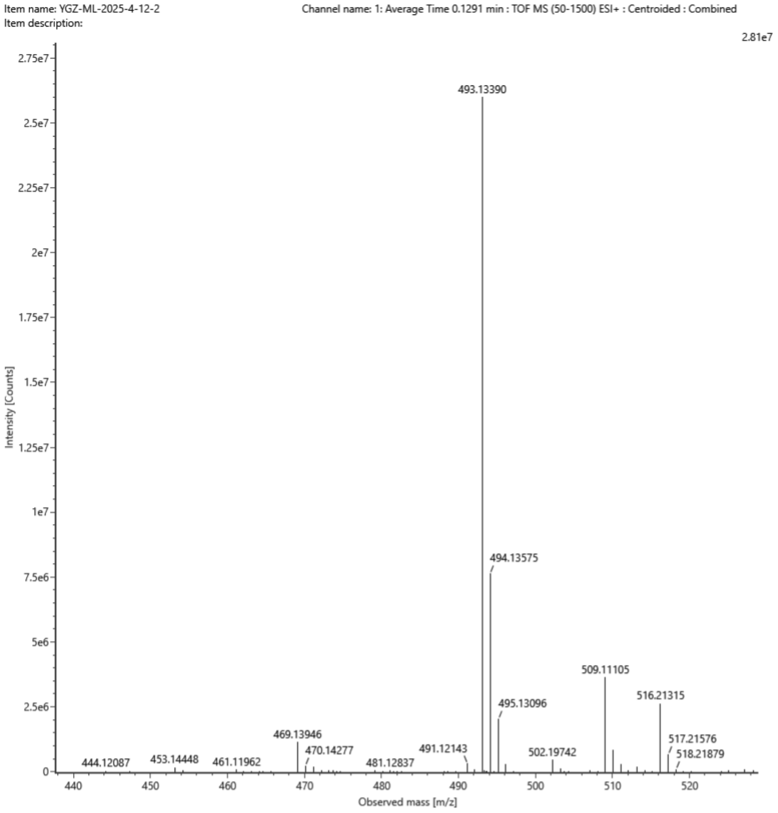

HRMS for Compound **3k**

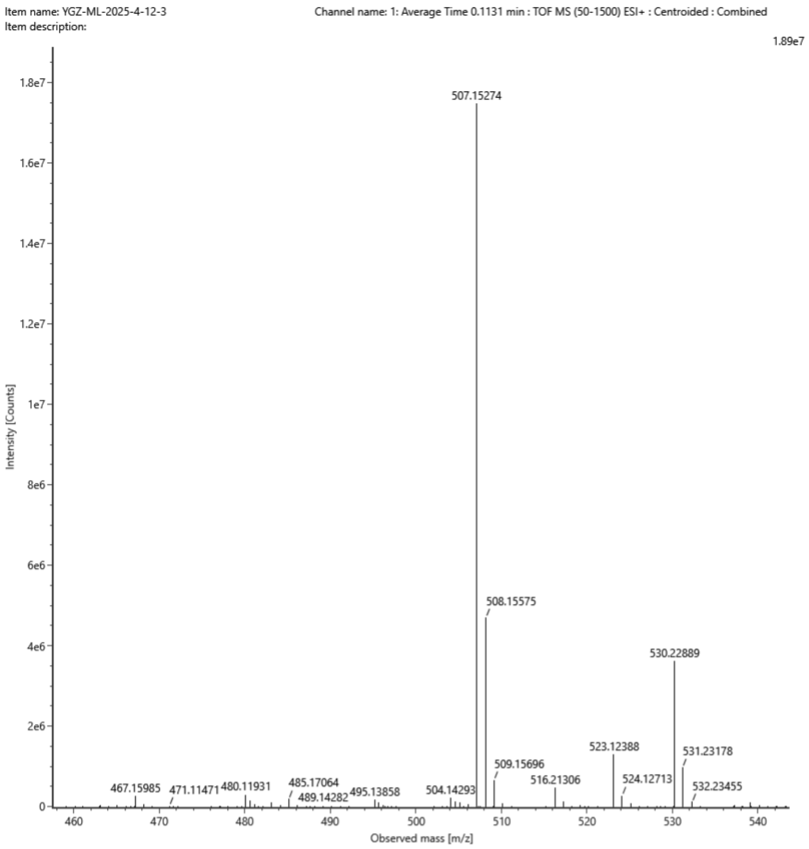

HRMS for Compound **3l**

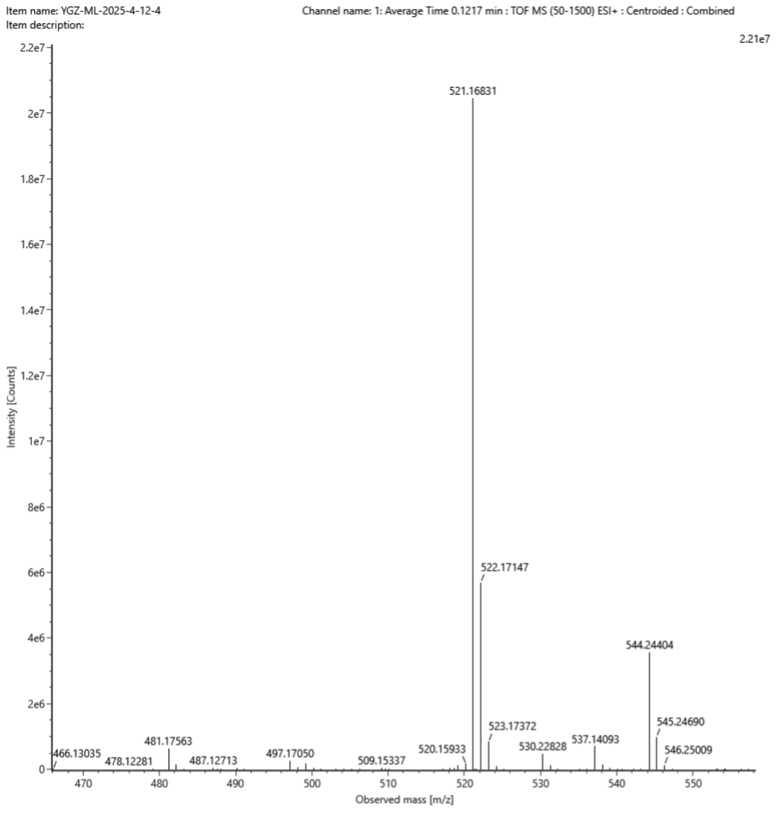

HRMS for Compound **3m**

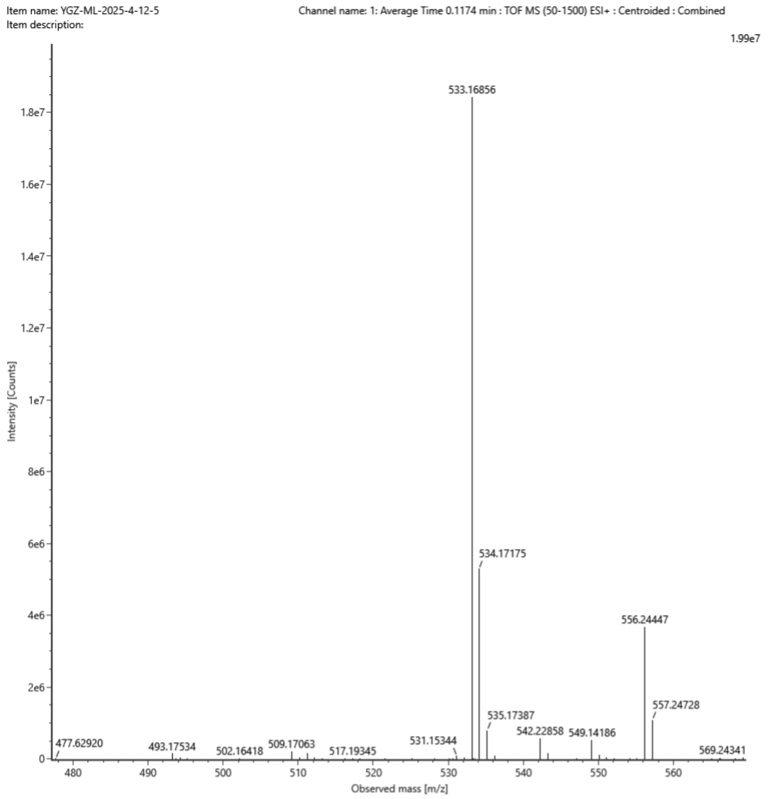

HRMS for Compound **3n**

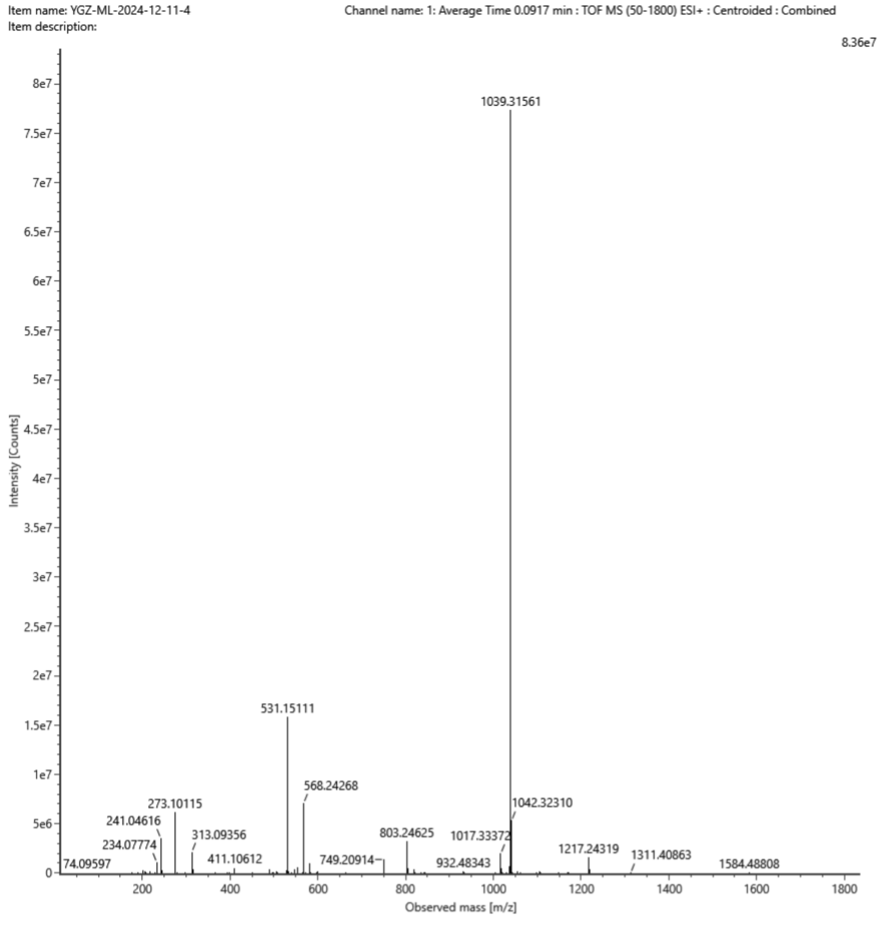

HRMS for Compound **3p**

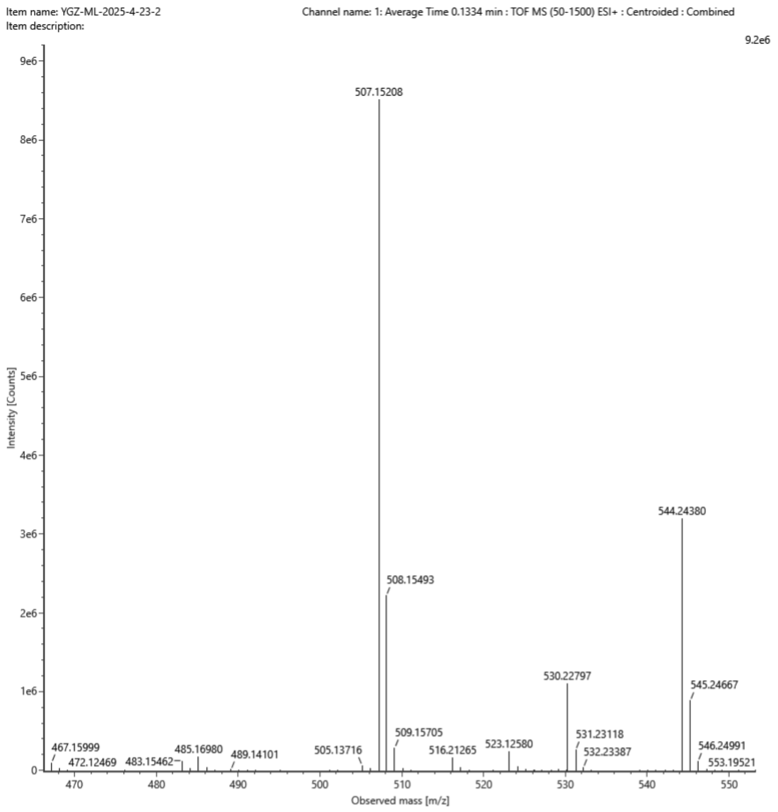

HRMS for Compound **3q**

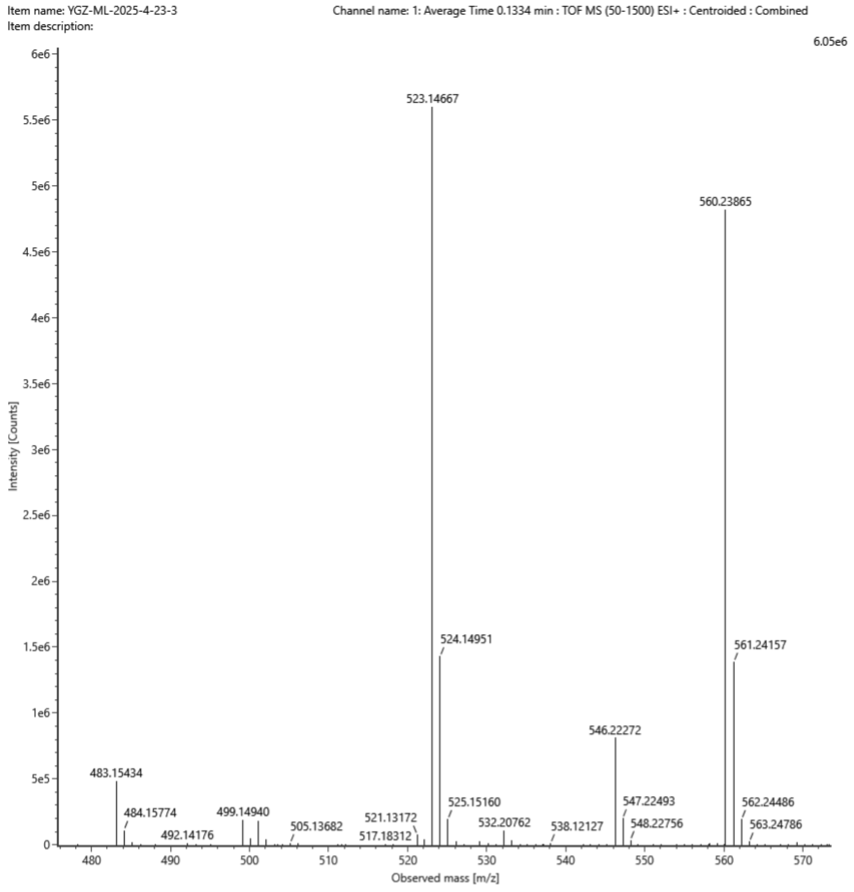

HRMS for Compound **3r**

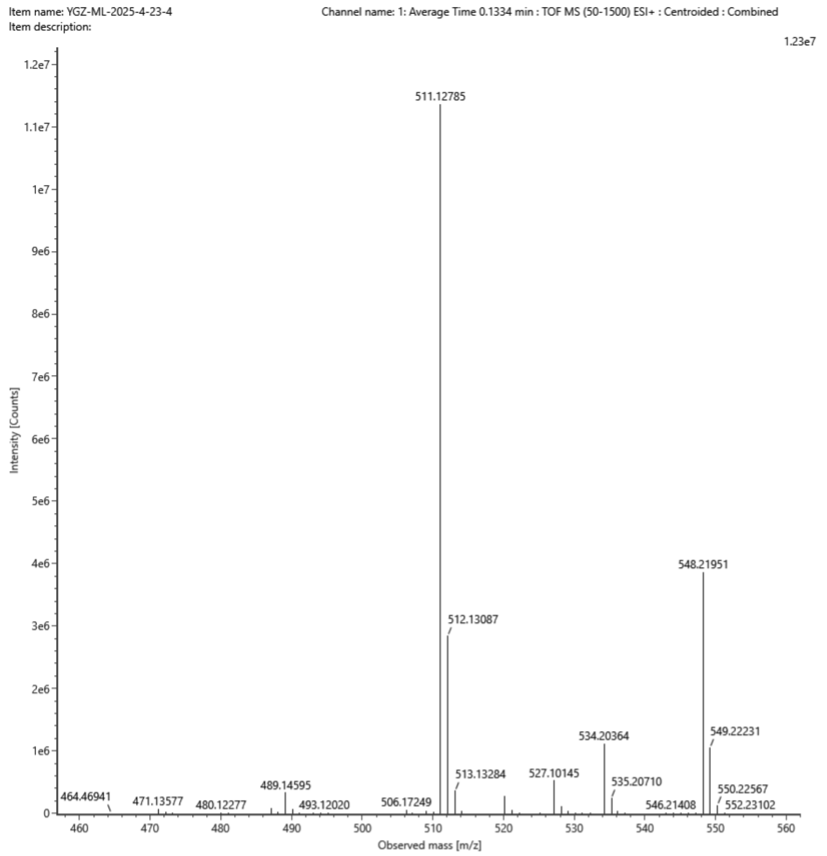

HRMS for Compound **3s**

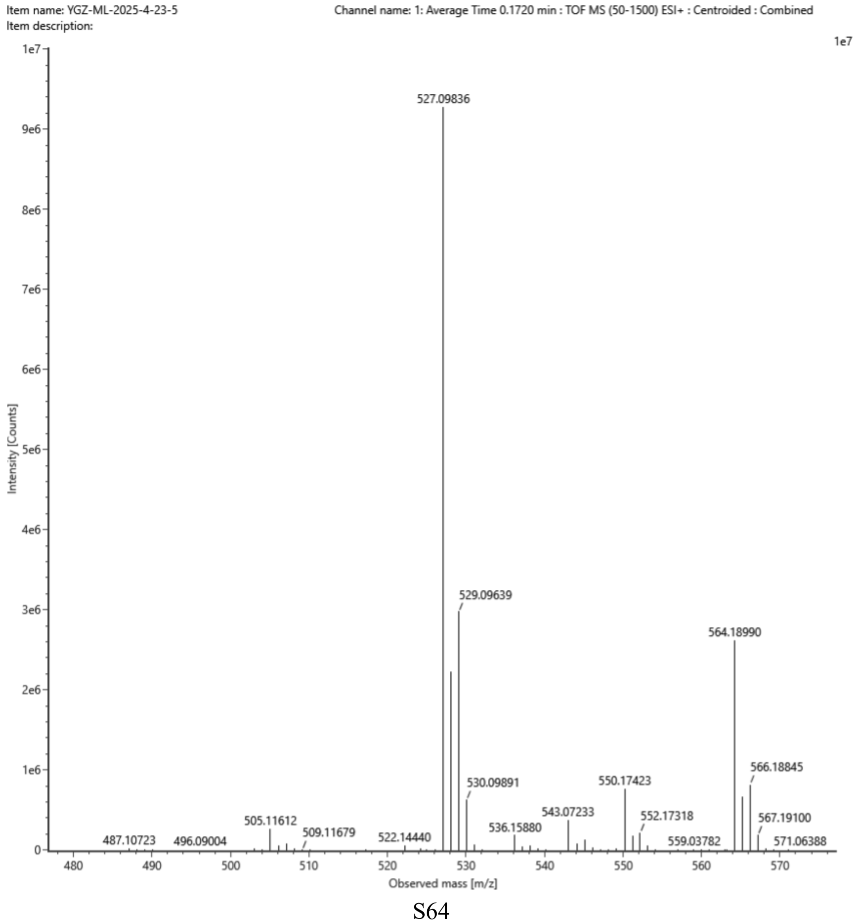

HRMS for Compound **3t**

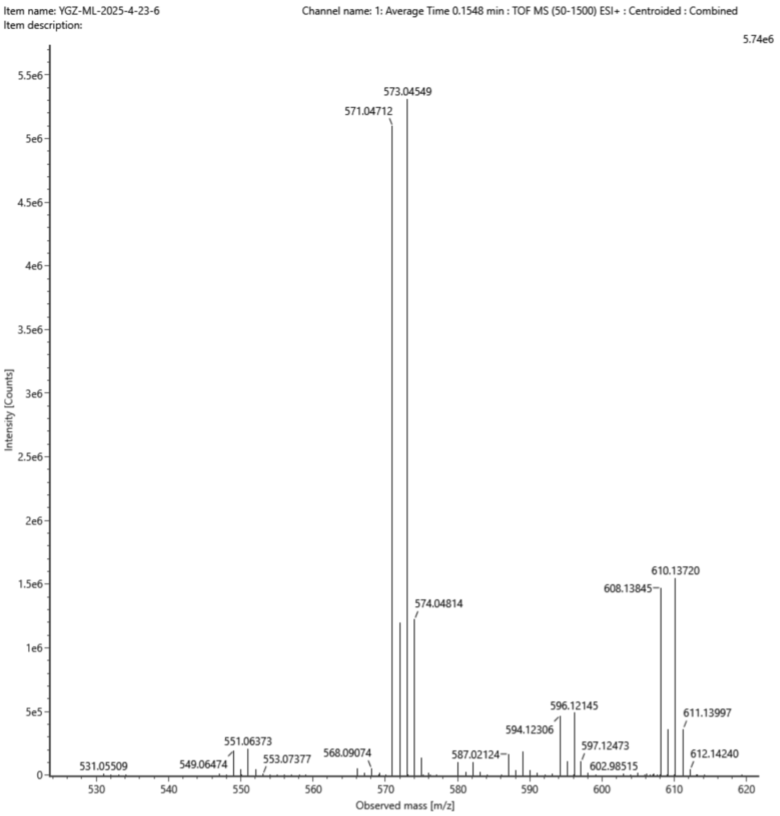

HRMS for Compound **3u**

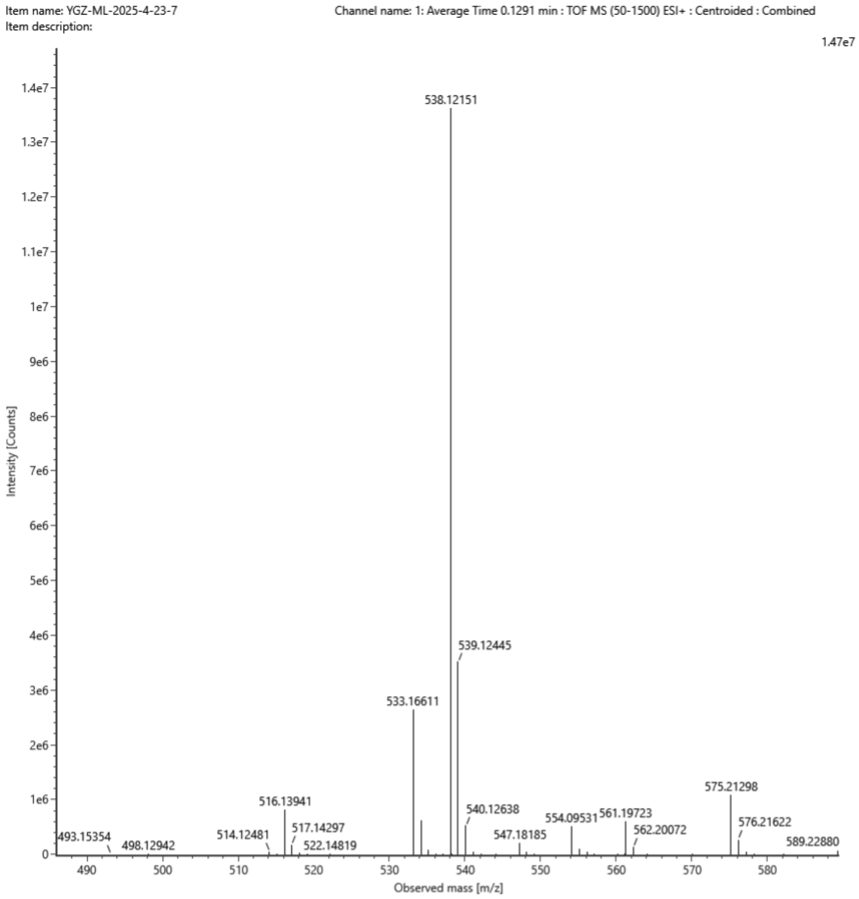

HRMS for Compound **3w**

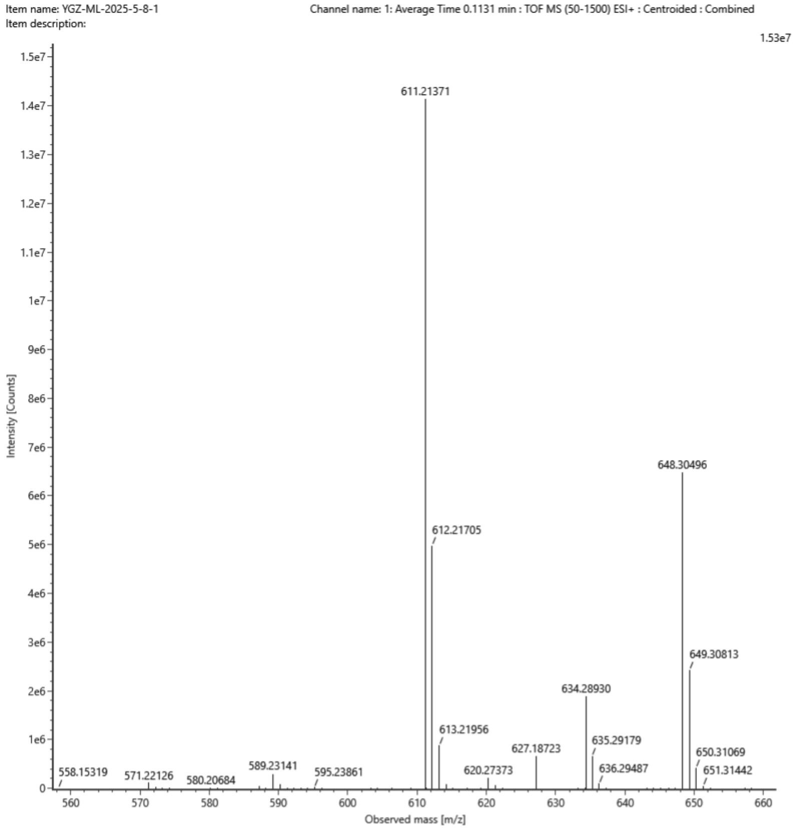

HRMS for Compound **3x**

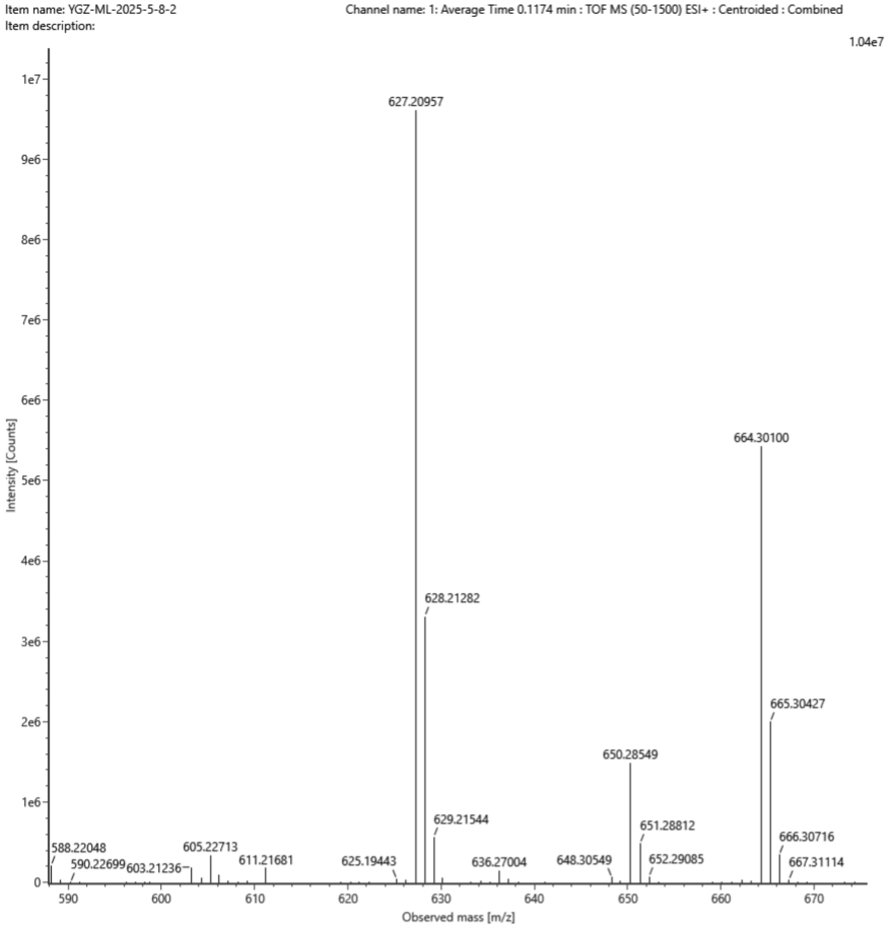

HRMS for Compound **3y**

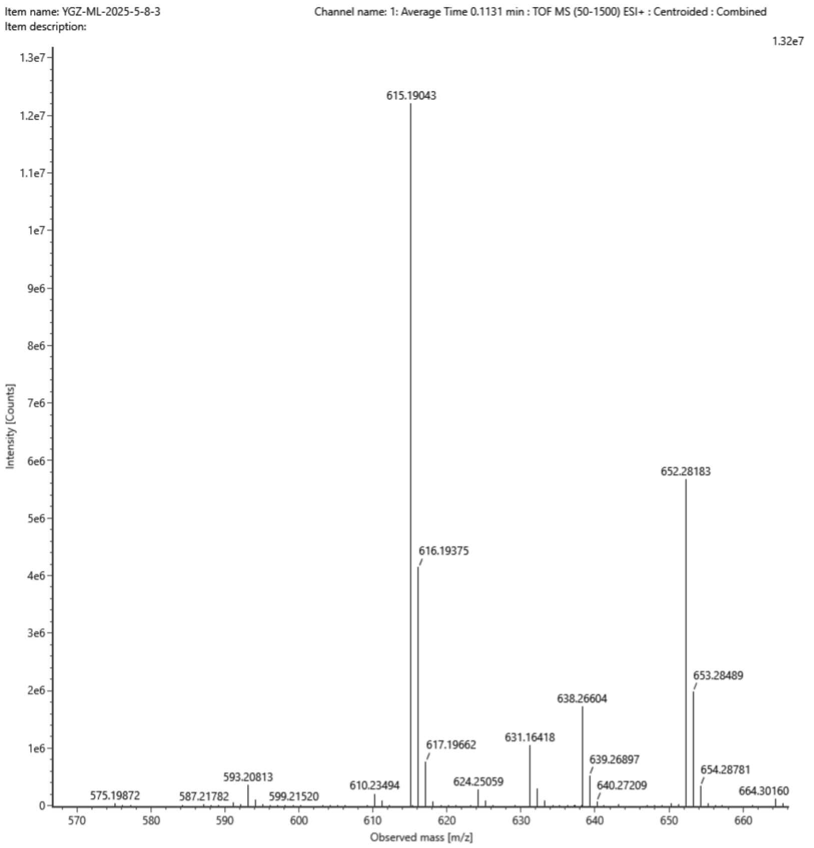

HRMS for Compound **3z**

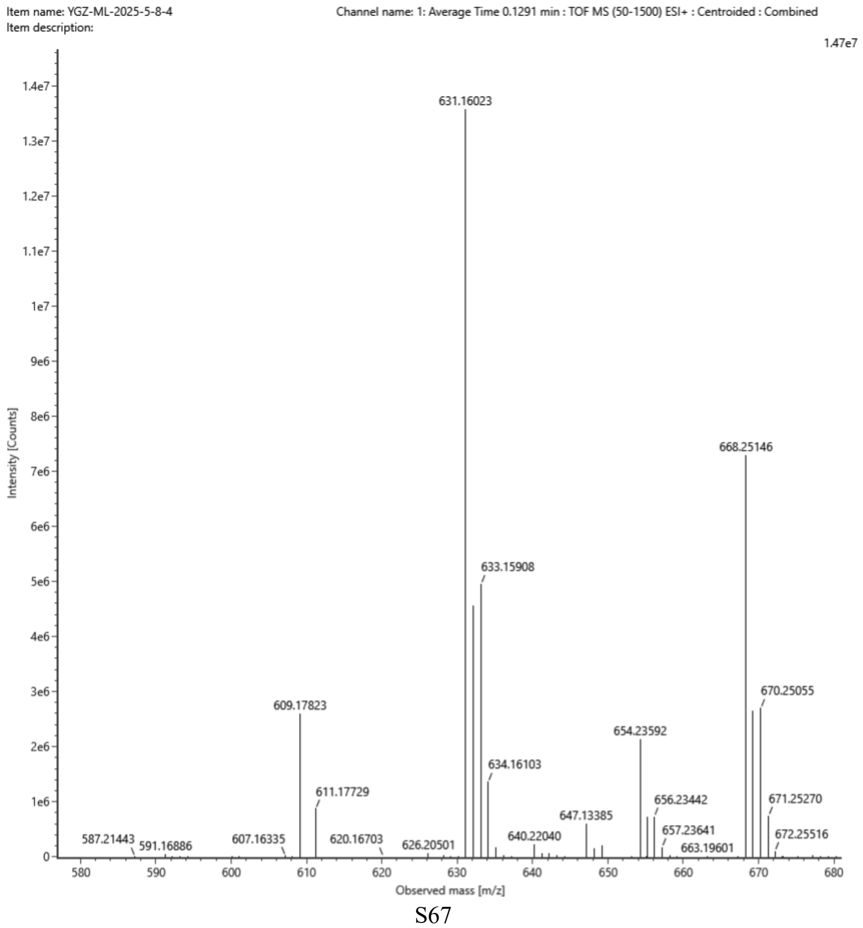

HRMS for Compound **3aa**

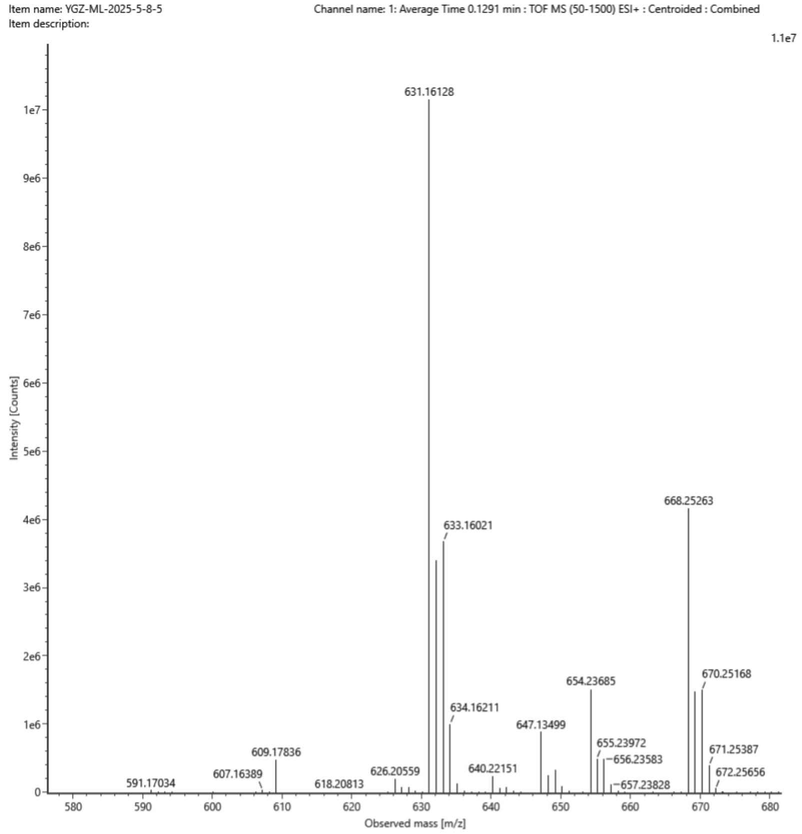

HRMS for Compound **3ab**

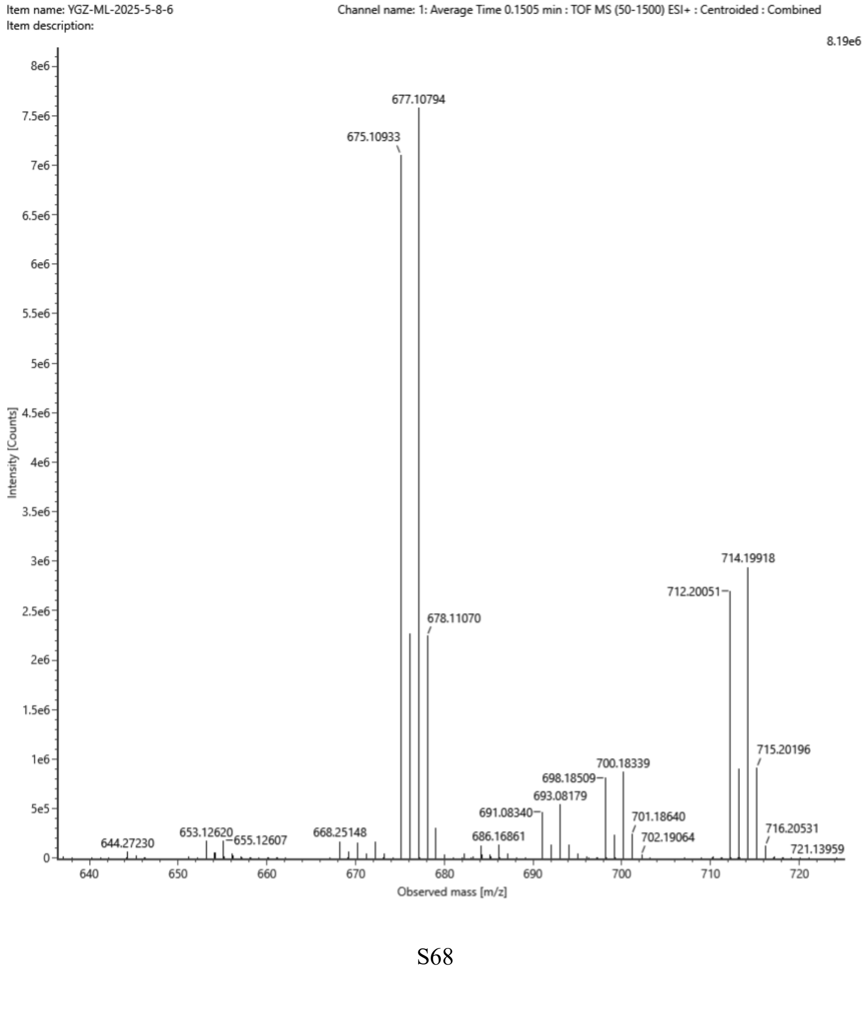

HRMS for Compound **3ac**

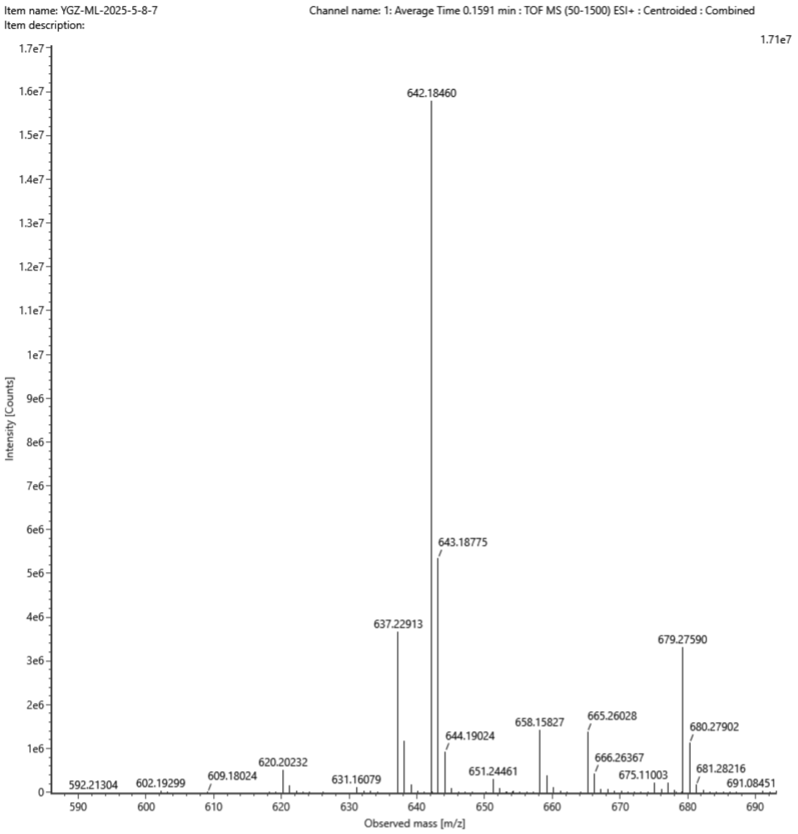

HRMS for Compound **3ad**

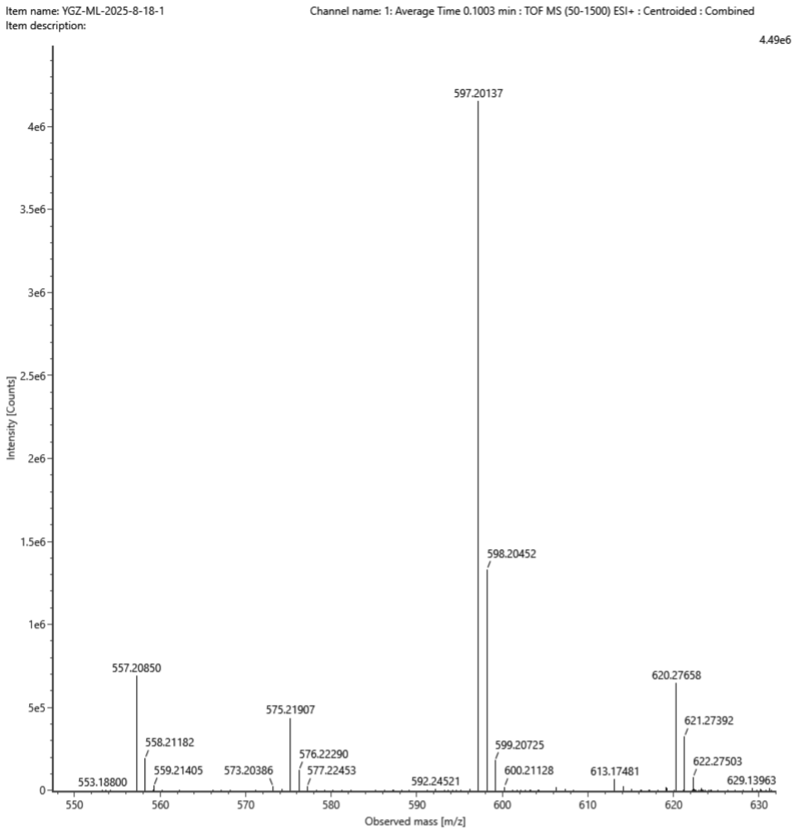

HRMS for Compound **3ae**

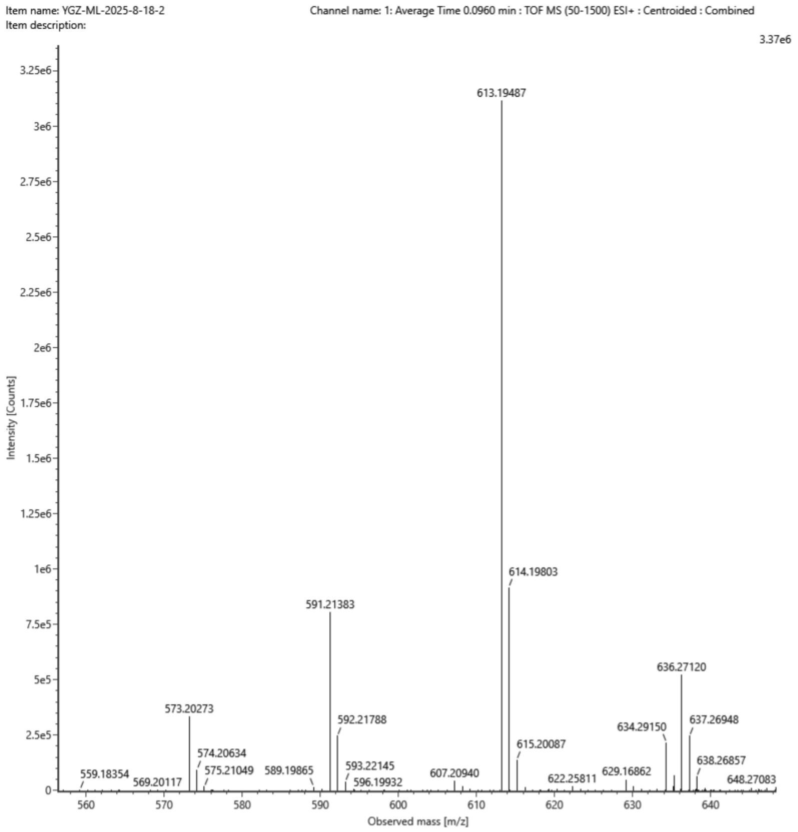

HRMS for Compound **3af**

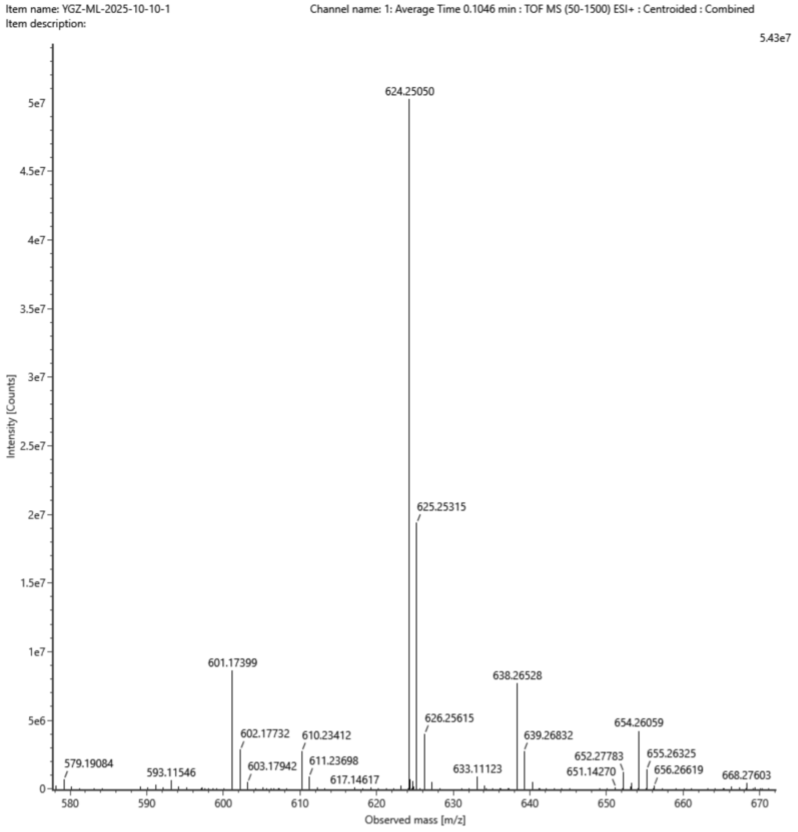

HRMS for Compound **3ag**

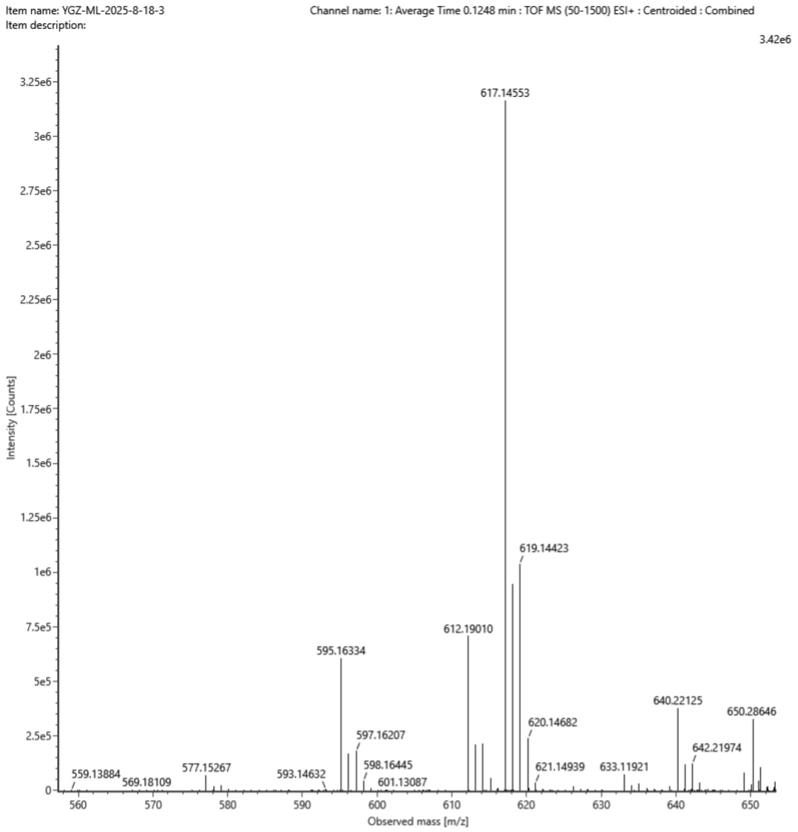

HRMS for Compound **3ah**

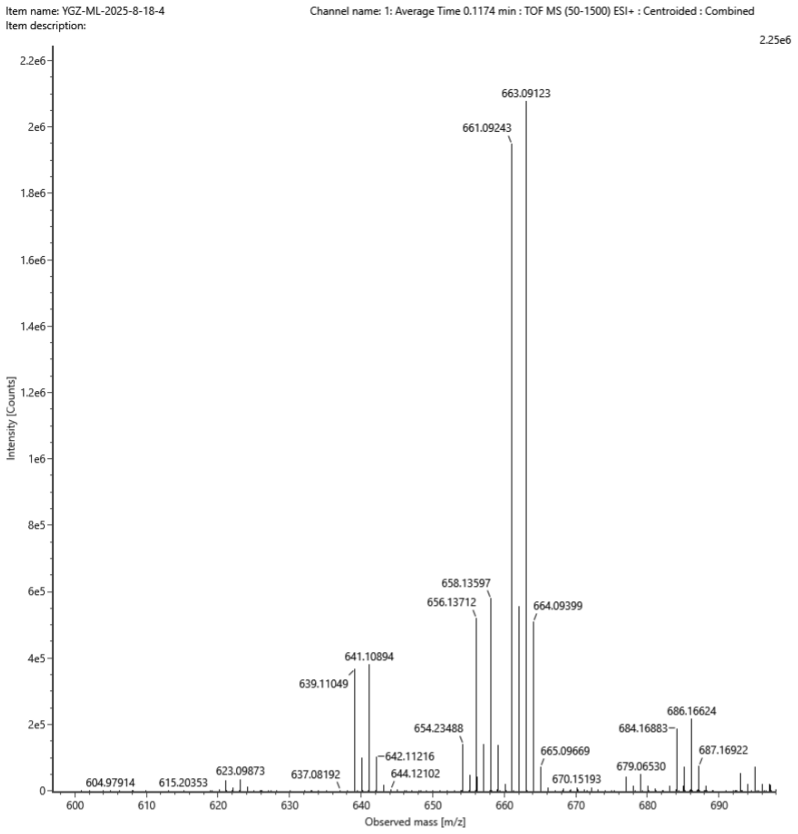

HRMS for Compound **3ai**

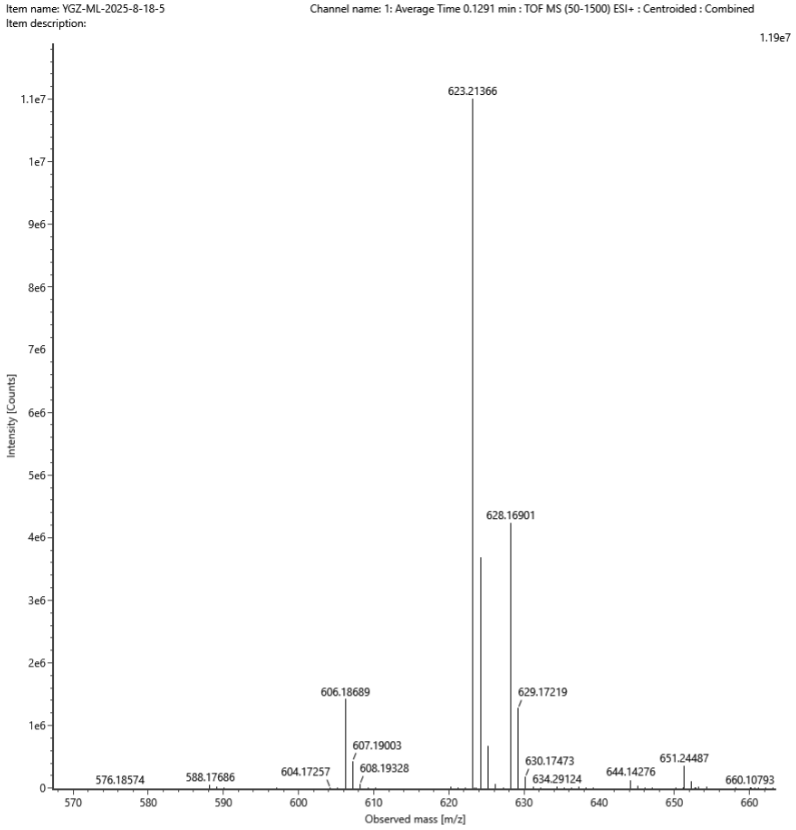

HRMS for Compound **4a**

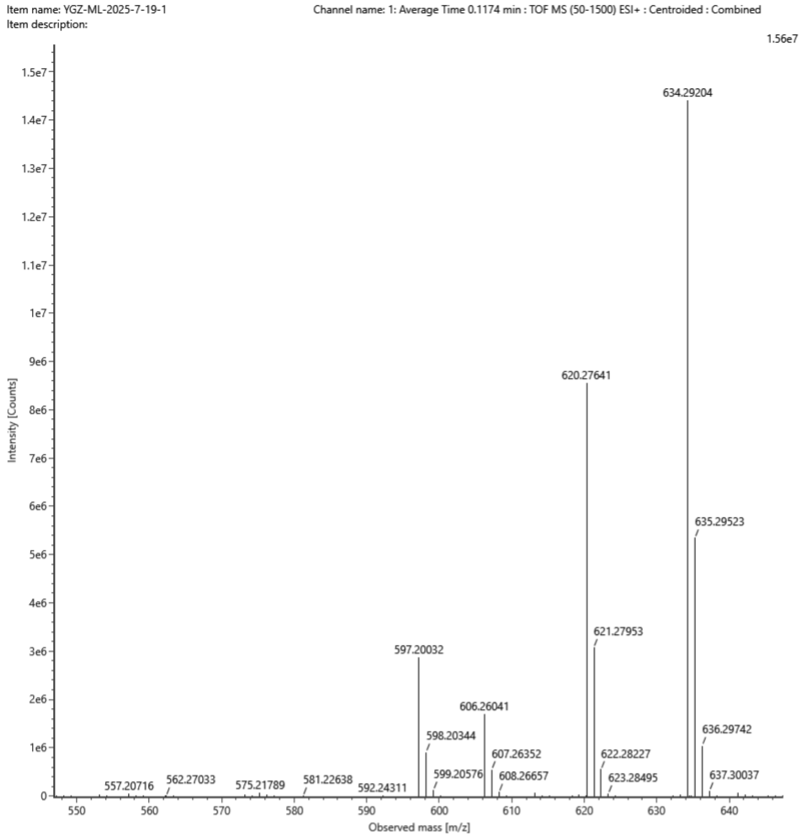

HRMS for Compound **4b**

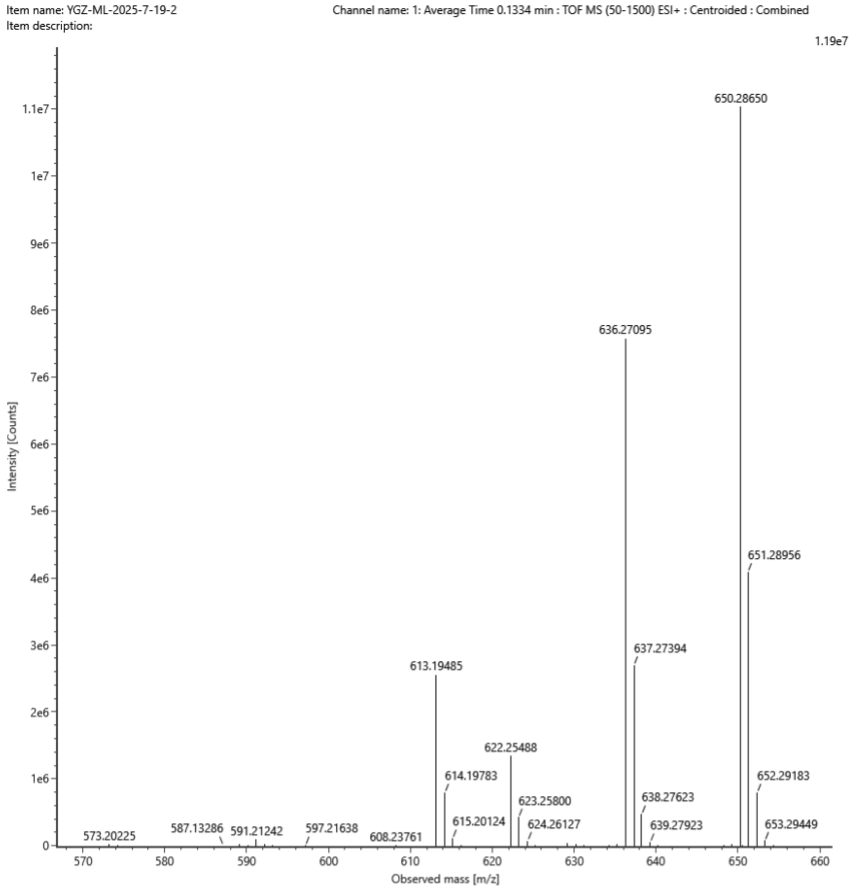

HRMS for Compound **4c**

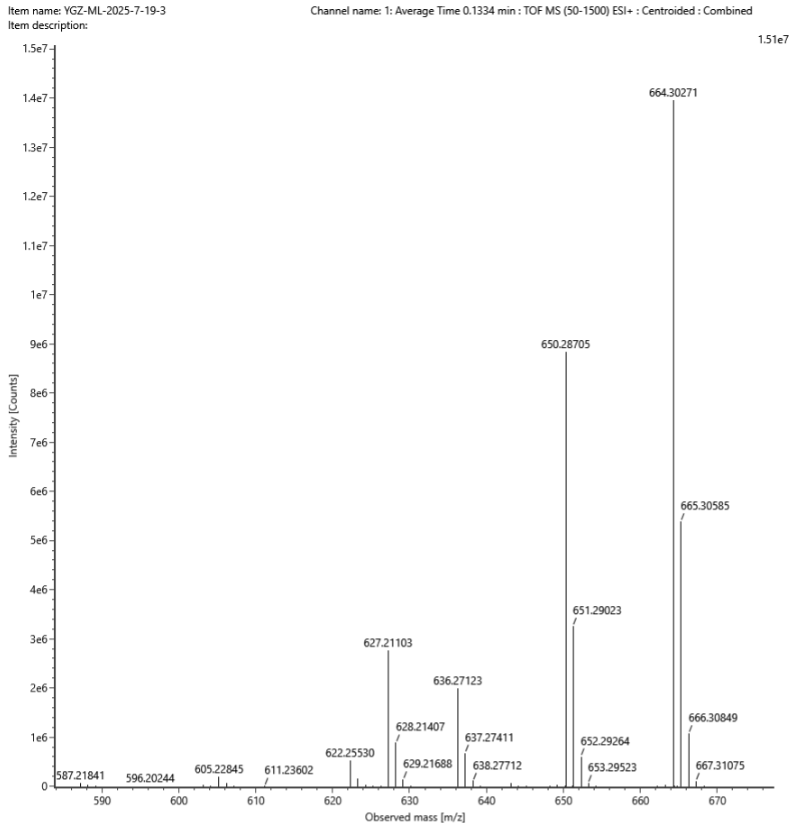

HRMS for Compound **4d**

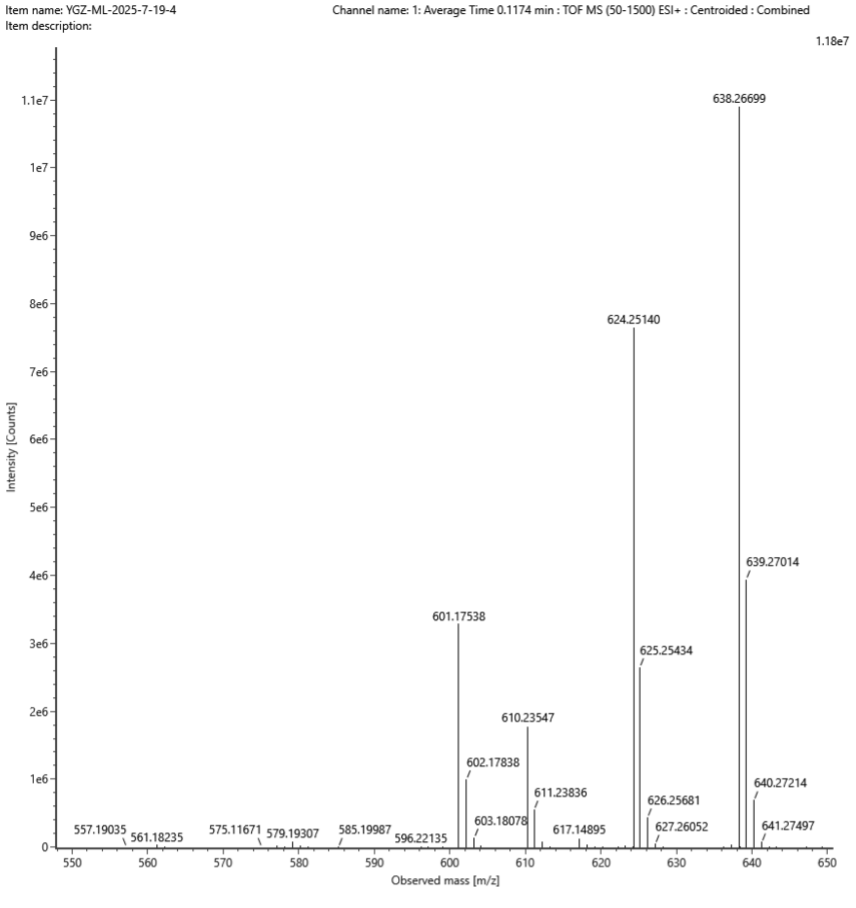

HRMS for Compound **4e**

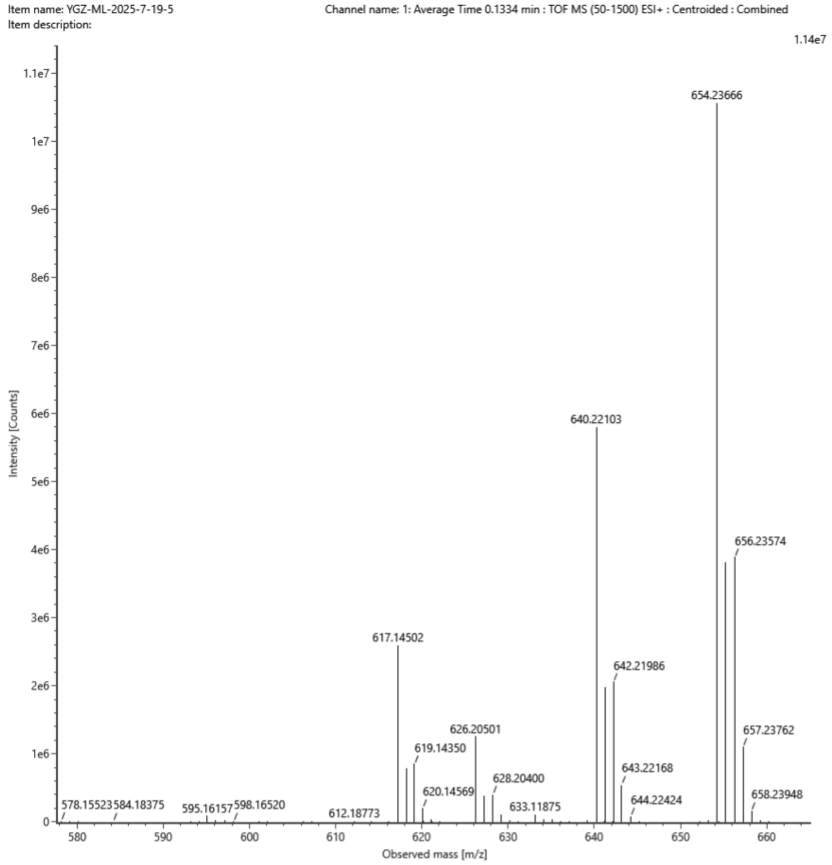

HRMS for Compound **4f**

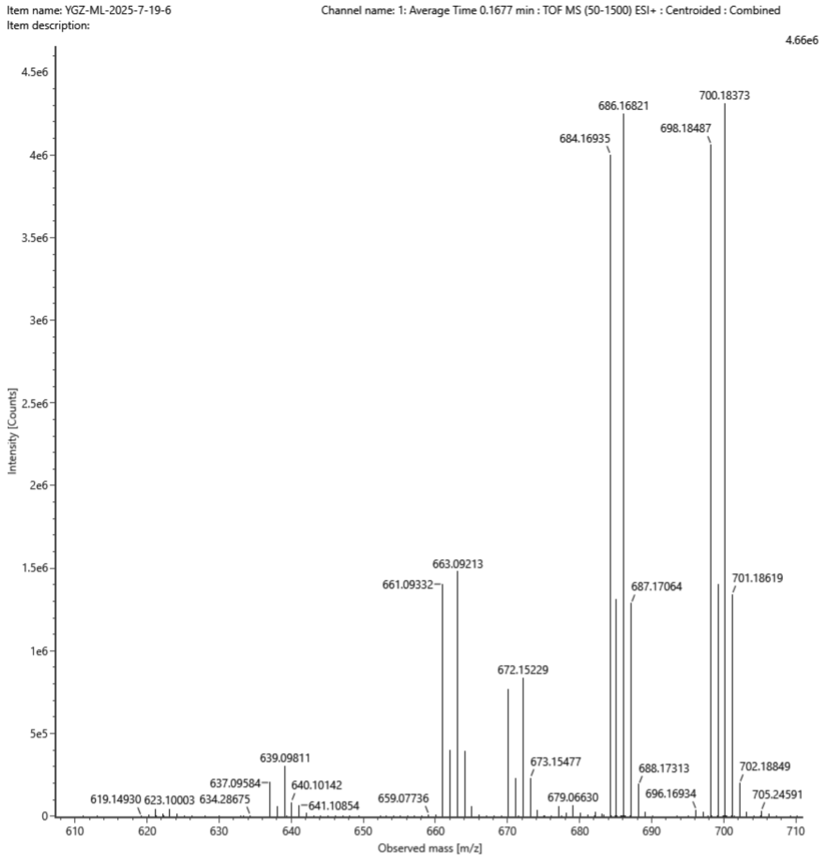

HRMS for Compound **4g**

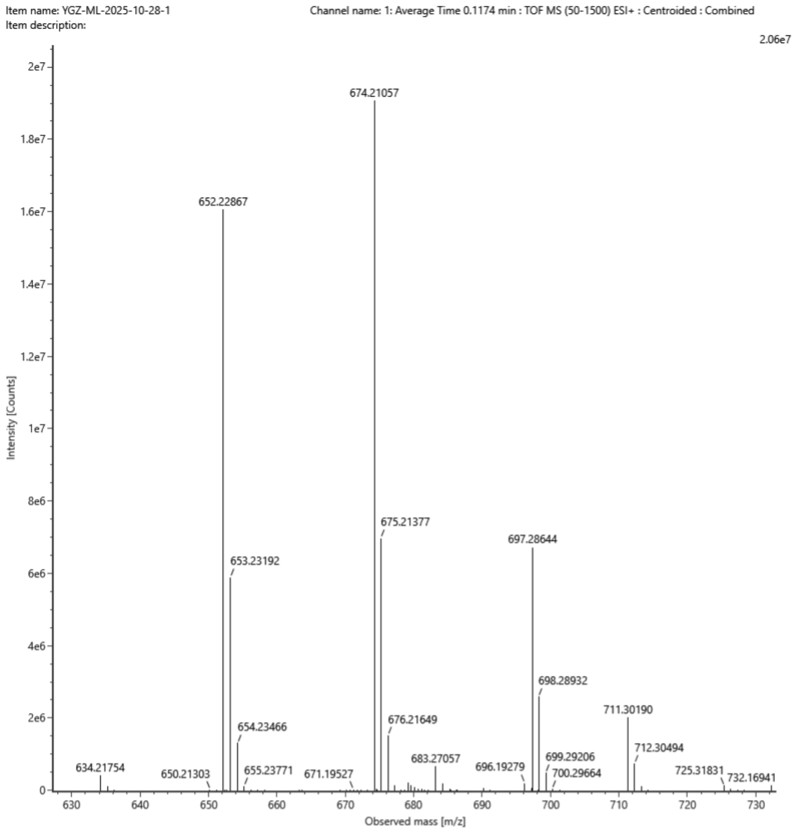

HRMS for Compound **4g´**

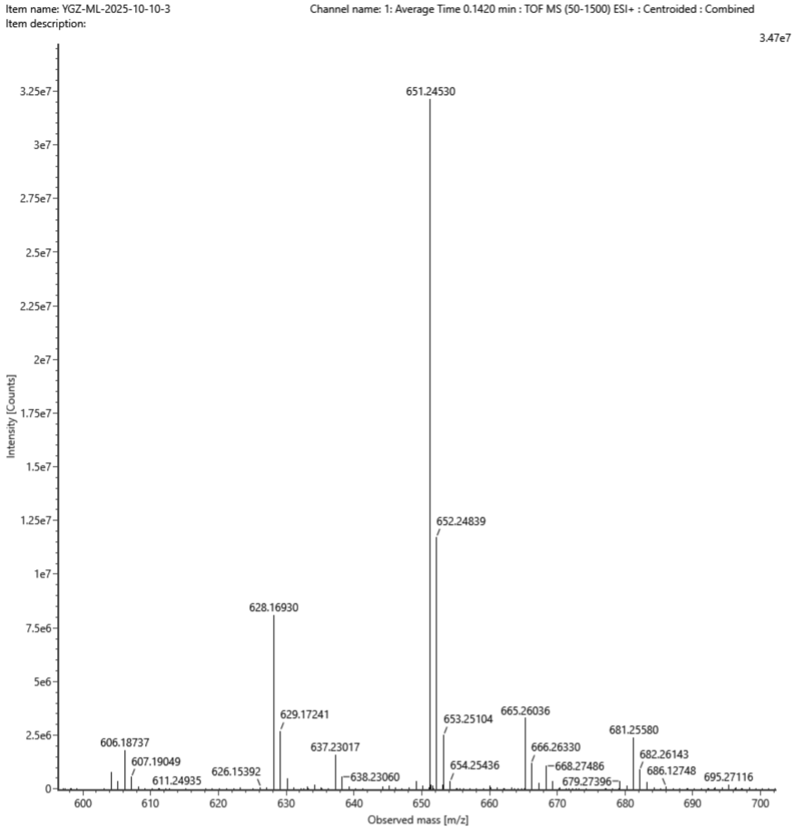

HRMS for Compound **4h**

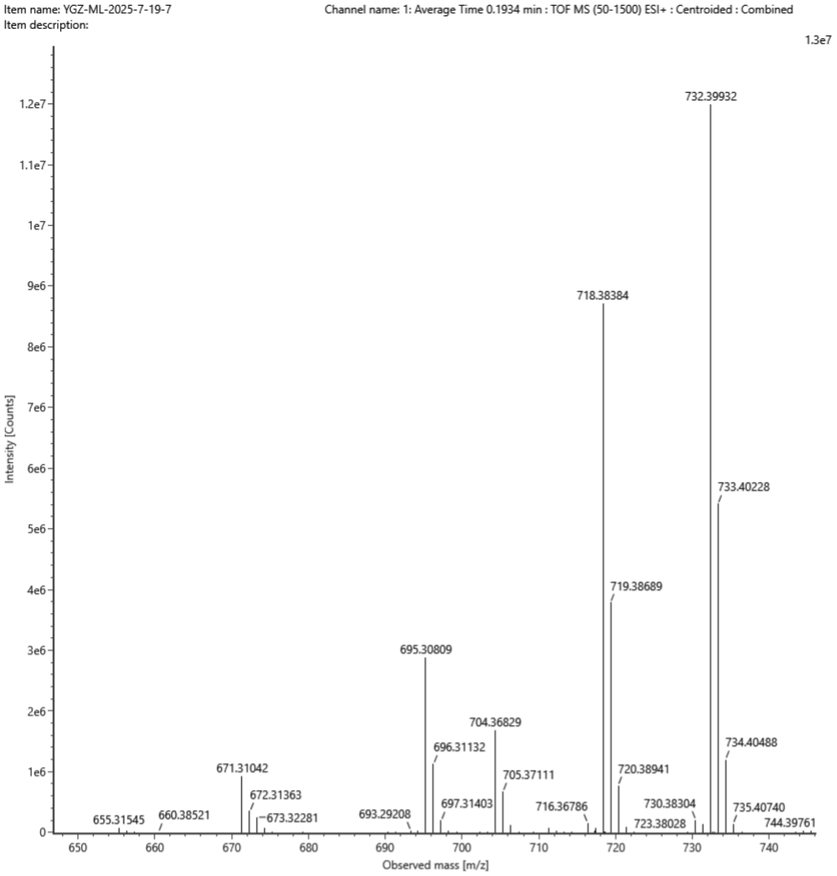

HRMS for Compound **4*J***

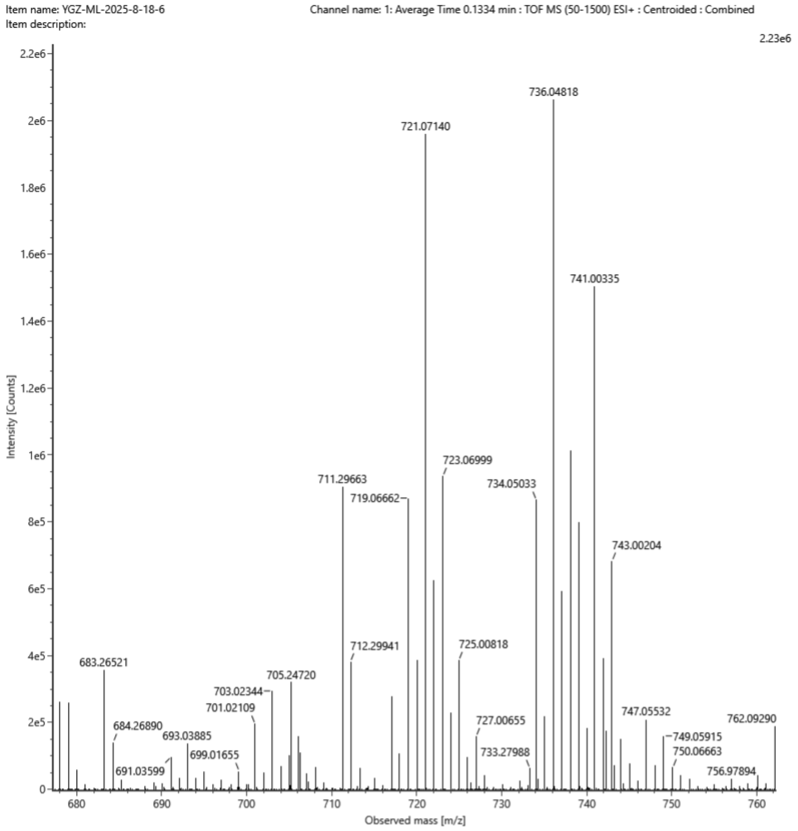

HRMS for Compound **4k**

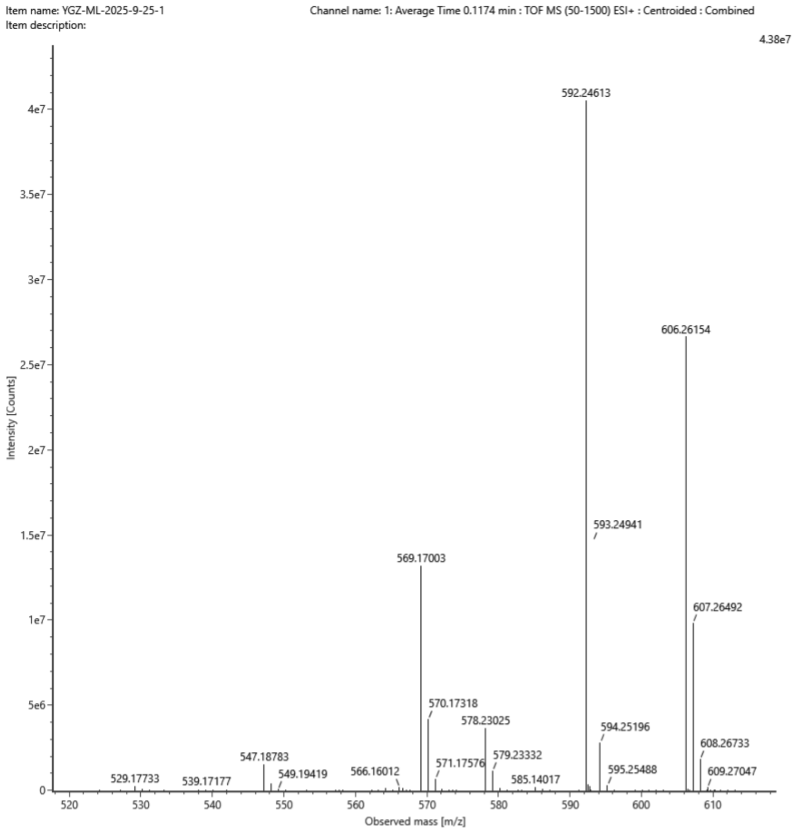

HRMS for Compound **4l**

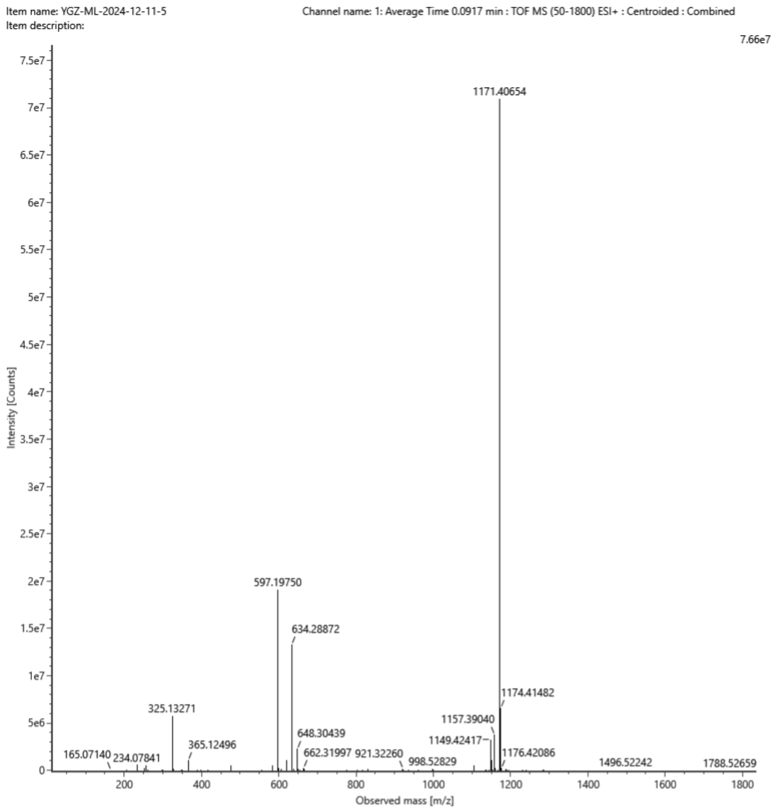

HRMS for Compound **4m**

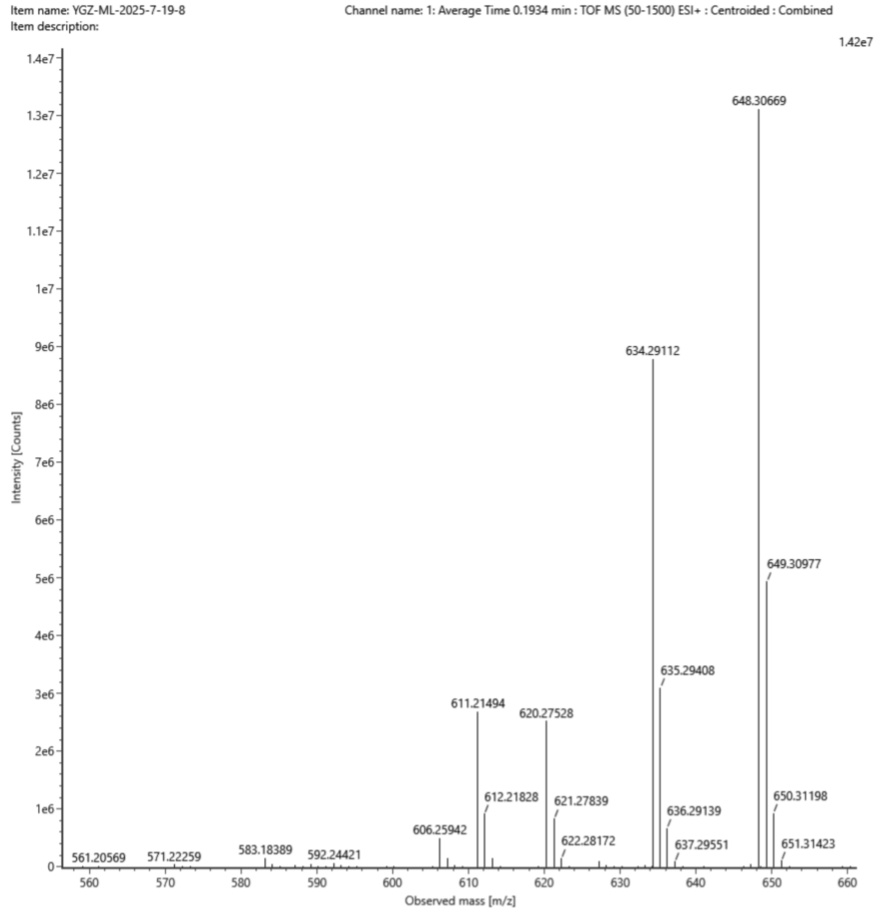

HRMS for Compound **4n**

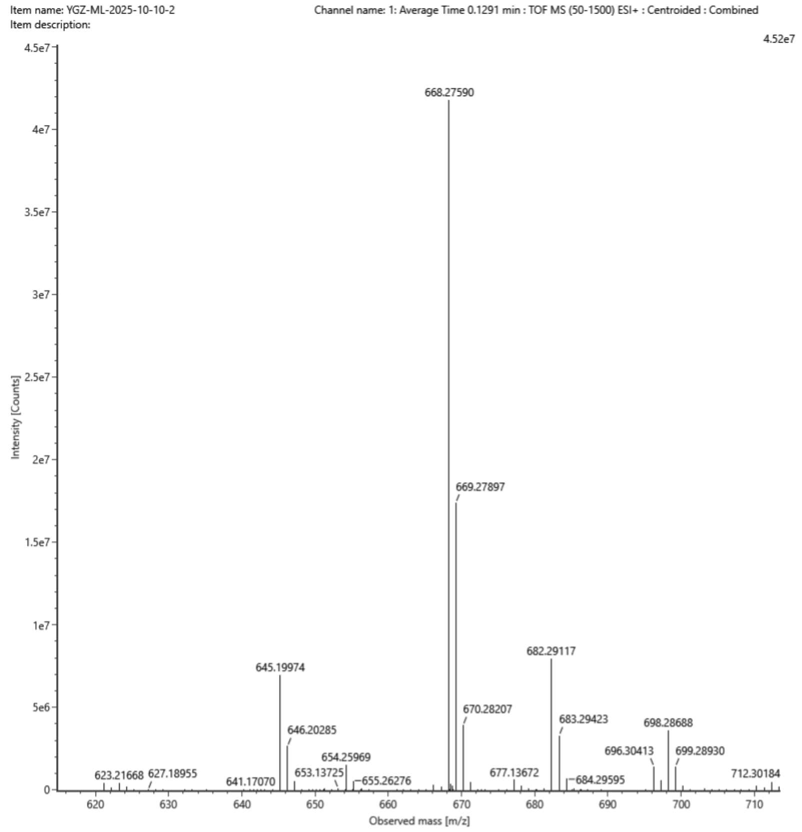

HRMS for Compound **6a**

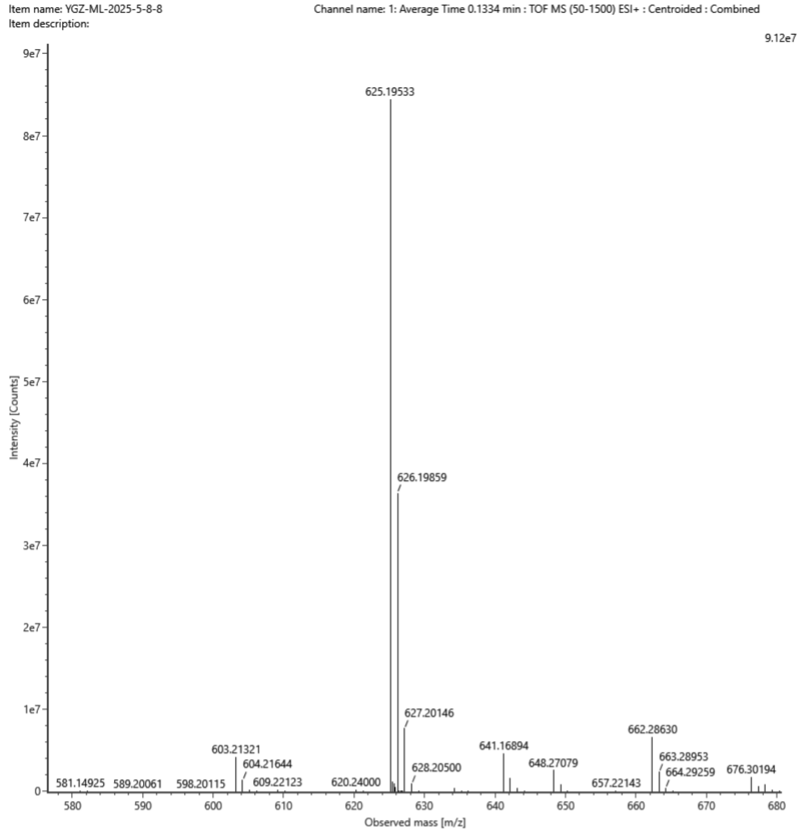

HRMS for Compound **6b**

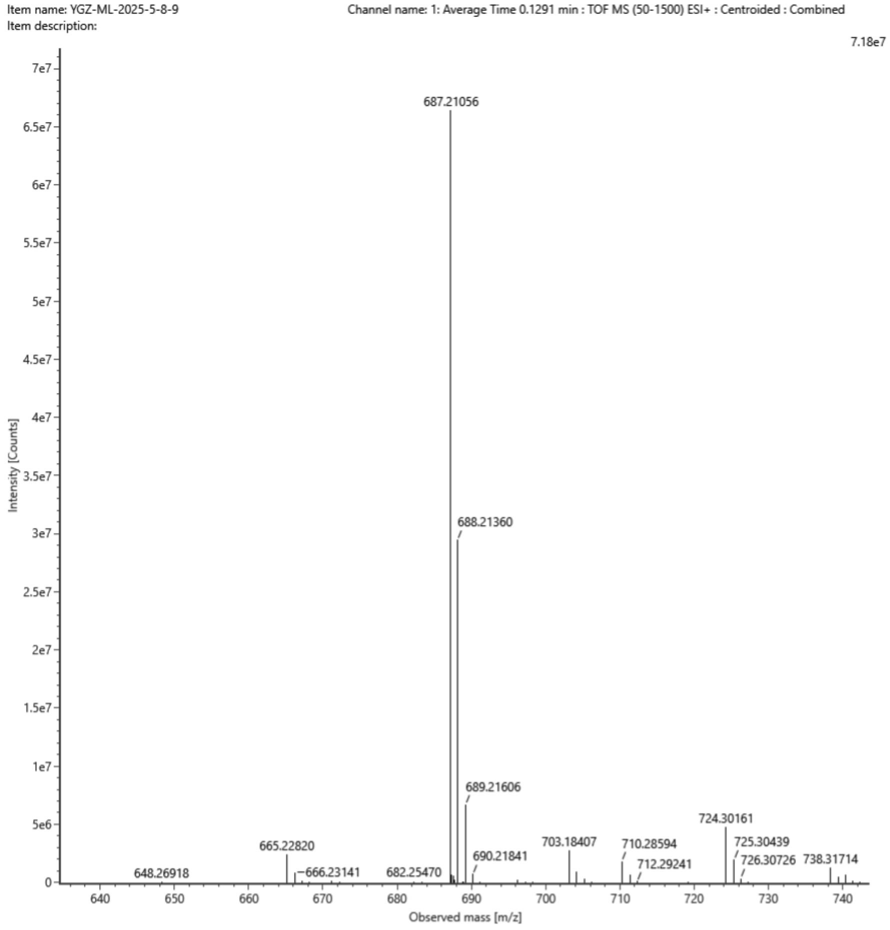

HRMS for Compound **8**

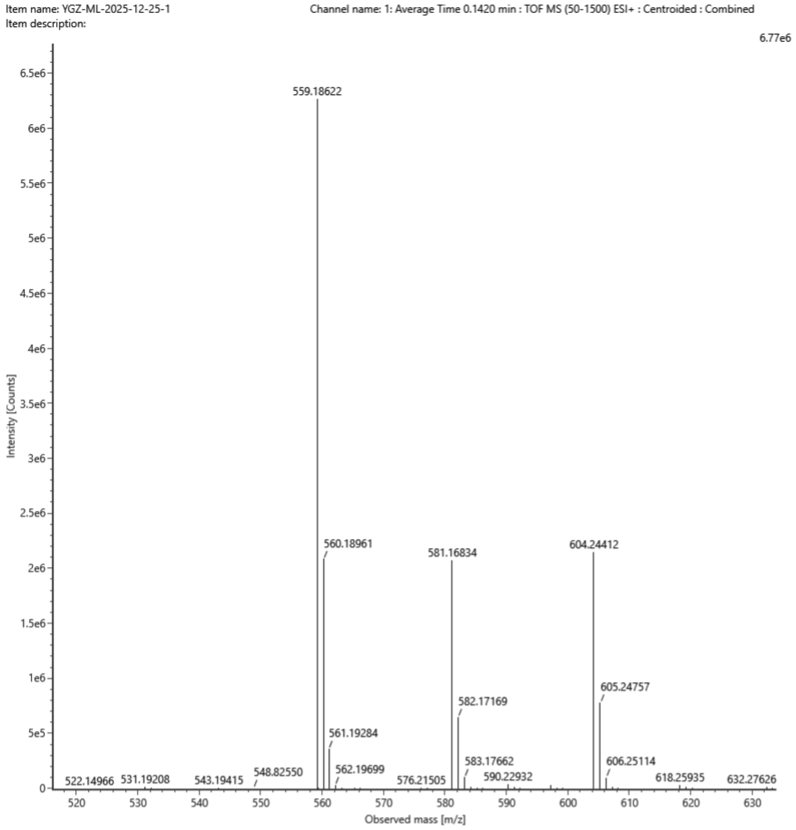

III. Copes of Data of X-ray crystal structure for 3b (CCDC 2375731)

Single crystals of **3b** were recrystallized from EtOH at room temperature for 5 days, and mounted in inert oil and transferred to the cold gas stream of the diffractometer.

Datablock lg2 - ellipsoid plot

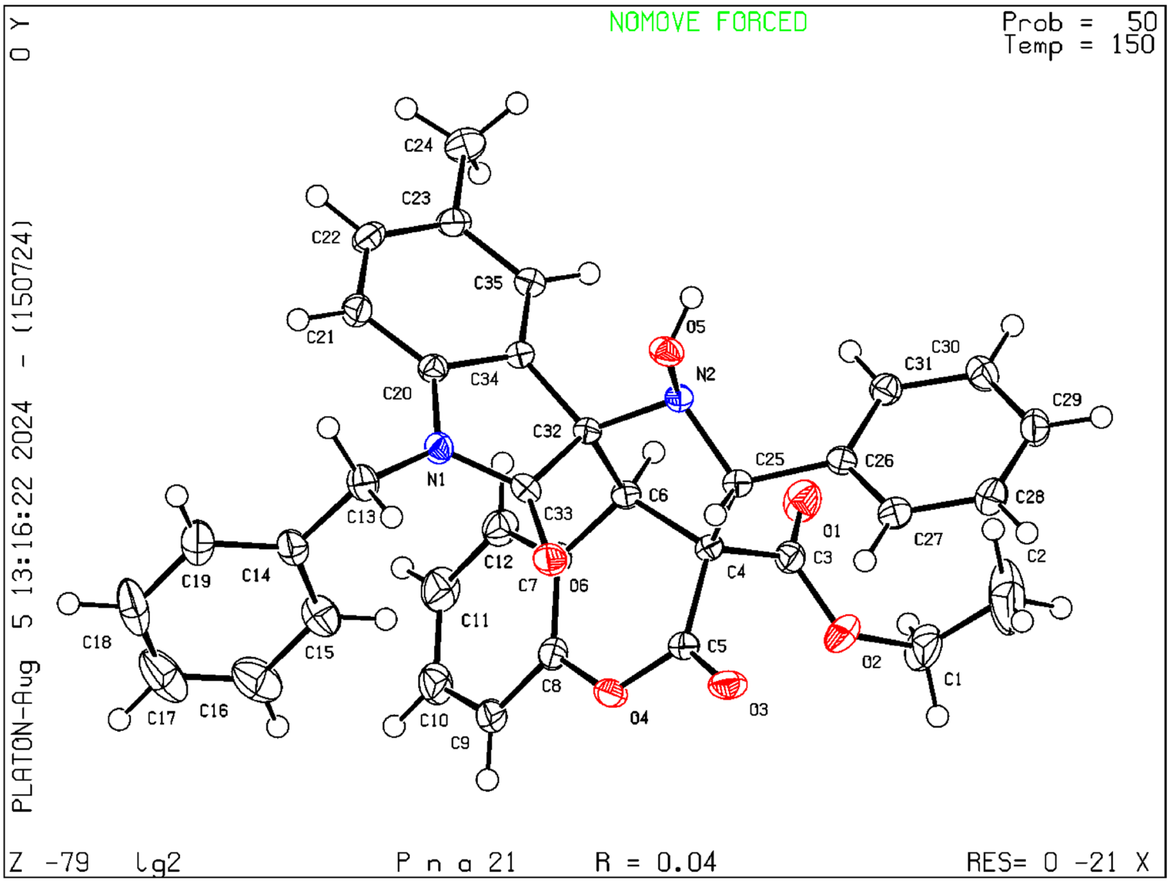

checkCIF/PLATON report

Structure factors have been supplied for datablock(s) lg2

THIS REPORT IS FOR GUIDANCE ONLY. IF USED AS PART OF A REVIEW PROCEDURE FOR PUBLICATION, IT SHOULD NOT REPLACE THE EXPERTISE OF AN EXPERIENCED CRYSTALLOGRAPHIC REFEREE.

No syntax errors found. CIF dictionary Interpreting this report

Datablock: lg2

```
Bond precision:    C-C = 0.0034 A            Wavelength=1.34139

Cell:               a=14.4368(8)      b=23.2190(13)      c=8.4873(4)
                    alpha=90          beta=90            gamma=90
Temperature:        150 K

                  Calculated                   Reported
Volume            2845.0(3)                    2845.0(3)
Space group       P n a 21                     P n a 21
Hall group        P 2c -2n                     P 2c -2n
Moiety formula    C35 H30 N2 O6                C35 H30 N2 O6
Sum formula       C35 H30 N2 O6                C35 H30 N2 O6
Mr                574.61                       574.61
Dx,g cm-3         1.342                        1.342
Z                 4                            4
Mu (mm-1)         0.478                        0.478
F000              1208.0                       1208.0
F000'             1210.87
h,k,lmax          18,29,10                     18,29,10
Nref              5835[ 3124]                  5716
Tmin,Tmax         0.944,0.962                  0.629,0.751
Tmin'             0.909

Correction method= # Reported T Limits: Tmin=0.629 Tmax=0.751
AbsCorr = MULTI-SCAN

Data completeness= 1.83/0.98        Theta(max)= 57.086

R(reflections)= 0.0360( 5136)                          wR2(reflections)=
                                                       0.0851( 5716)
S = 1.066                  Npar= 391
```

```
The following ALERTS were generated. Each ALERT has the format
       test-name_ALERT_alert-type_alert-level.
Click on the hyperlinks for more details of the test.
```

Alert level C

```
PLAT220_ALERT_2_C NonSolvent   Resd 1  C   Ueq(max)/Ueq(min) Range        4.9 Ratio
PLAT222_ALERT_3_C NonSolvent Resd 1  H   Uiso(max)/Uiso(min) Range        6.1 Ratio
PLAT242_ALERT_2_C Low    'MainMol' Ueq as Compared to Neighbors of         C1 Check
PLAT911_ALERT_3_C Missing FCF Refl Between Thmin & STh/L=    0.600          8 Report
                2  0  0,   0  2  0,   2  0  1,   0  1  1,   2  1  1,   1  5  1,
                2  0  2,   0  2  2,
PLAT913_ALERT_3_C Missing # of Very Strong Reflections in FCF ....          6 Note
                0  2  0,   2  0  1,   0  1  1,   1  5  1,   2  0  2,   0  2  2,
```

Alert level G

```
ABSMU01_ALERT_1_G  Calculation of _exptl_absorpt_correction_mu
                not performed for this radiation type.
PLAT003_ALERT_2_G Number of Uiso or U(i,j) Restrained non-H Atoms         10 Report
PLAT007_ALERT_5_G Number of Unrefined Donor-H Atoms .............          1 Report
              H5
PLAT178_ALERT_4_G The CIF-Embedded .res File Contains SIMU Records         2 Report
PLAT186_ALERT_4_G The CIF-Embedded .res File Contains ISOR Records         1 Report
PLAT188_ALERT_3_G A Non-default SIMU Restraint Value has been used     0.0100 Report
PLAT188_ALERT_3_G A Non-default SIMU Restraint Value has been used     0.0100 Report
PLAT792_ALERT_1_G Model has Chirality at C4            (Polar  SpGr)         S Verify
PLAT792_ALERT_1_G Model has Chirality at C6            (Polar  SpGr)         R Verify
PLAT792_ALERT_1_G Model has Chirality at C25           (Polar  SpGr)         R Verify
PLAT792_ALERT_1_G Model has Chirality at C32           (Polar  SpGr)         R Verify
PLAT860_ALERT_3_G Number of Least-Squares Restraints .............         61 Note
PLAT912_ALERT_4_G Missing # of FCF Reflections Above STh/L=  0.600          2 Note
PLAT969_ALERT_5_G The 'Henn et al.' R-Factor-gap value ..........      2.288 Note
              Predicted wR2: Based on SigI**2  3.72 or SHELX Weight  7.98
PLAT978_ALERT_2_G Number C-C Bonds with Positive Residual Density.          5 Info
```

```
   0 ALERT level A = Most likely a serious problem - resolve or explain
   0 ALERT level B = A potentially serious problem, consider carefully
   5 ALERT level C = Check. Ensure it is not caused by an omission or oversight
  15 ALERT level G = General information/check it is not something unexpected

   5 ALERT type 1 CIF construction/syntax error, inconsistent or missing data
   4 ALERT type 2 Indicator that the structure model may be wrong or deficient
   6 ALERT type 3 Indicator that the structure quality may be low
   3 ALERT type 4 Improvement, methodology, query or suggestion
   2 ALERT type 5 Informative message, check
```

It is advisable to attempt to resolve as many as possible of the alerts in all categories. Often the minor alerts point to easily fixed oversights, errors and omissions in your CIF or refinement strategy, so attention to these fine details can be worthwhile. In order to resolve some of the more serious problems it may be necessary to carry out additional measurements or structure refinements. However, the purpose of your study may justify the reported deviations and the more serious of these should normally be commented upon in the discussion or experimental section of a paper or in the "special_details" fields of the CIF. checkCIF was carefully designed to identify outliers and unusual parameters, but every test has its limitations and alerts that are not important in a particular case may appear. Conversely, the absence of alerts does not guarantee there are no aspects of the results needing attention. It is up to the individual to critically assess their own results and, if necessary, seek expert advice.

Publication of your CIF in IUCr journals

A basic structural check has been run on your CIF. These basic checks will be run on all CIFs submitted for publication in IUCr journals (*Acta Crystallographica*, *Journal of Applied Crystallography*, *Journal of Synchrotron Radiation*); however, if you intend to submit to *Acta Crystallographica Section C* or *E* or *IUCrData*, you should make sure that full publication checks are run on the final version of your CIF prior to submission.

Publication of your CIF in other journals

Please refer to the *Notes for Authors* of the relevant journal for any special instructions relating to CIF submission.

PLATON version of 15/07/2024; check.def file version of 15/07/2024

IV. Copes of Data of X-ray crystal structure for 8 (CCDC 2518523)

Single crystals of **8** were recrystallized from DMSO/EtOH (1:4, v/v) at room temperature for 4 days, and mounted in inert oil and transferred to the cold gas stream of the diffractometer.

Datablock I1 - ellipsoid plot

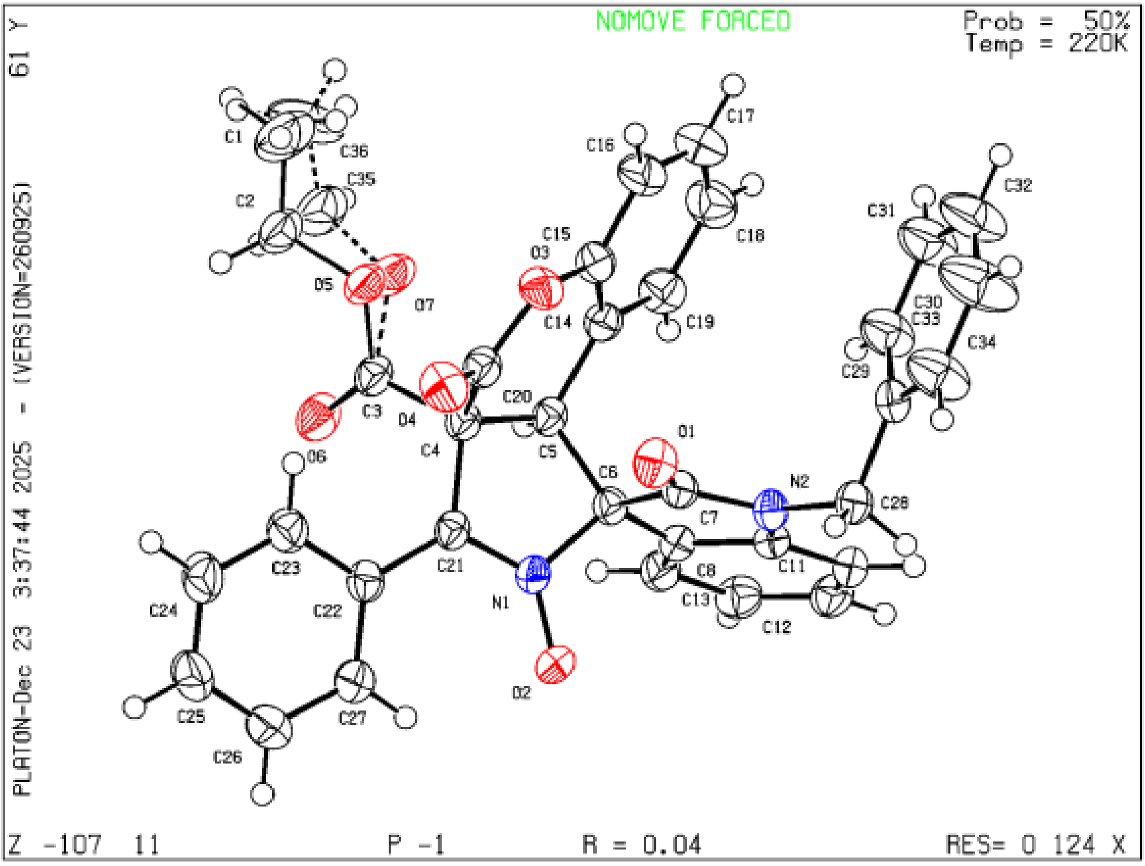

checkCIF/PLATON report

Structure factors have been supplied for datablock(s) 11

THIS REPORT IS FOR GUIDANCE ONLY. IF USED AS PART OF A REVIEW PROCEDURE FOR PUBLICATION, IT SHOULD NOT REPLACE THE EXPERTISE OF AN EXPERIENCED CRYSTALLOGRAPHIC REFEREE.

No syntax errors found. CIF dictionary Interpreting this report

Datablock: 11

Bond precision: C-C = 0.0022 A Wavelength=1.34139

Cell:	a=9.6767(6)	b=11.3133(7)	c=13.0267(8)
	alpha=82.884(2)	beta=78.990(2)	gamma=73.017(2)
Temperature:	220 K		

	Calculated	Reported
Volume	1335.28(14)	1335.28(14)
Space group	P -1	P -1
Hall group	-P 1	-P 1
Moiety formula	C34 H26 N2 O6	C34 H26 N2 O6
Sum formula	C34 H26 N2 O6	C34 H26 N2 O6
Mr	558.57	558.57
Dx,g cm-3	1.389	1.389
Z	2	2
Mu (mm-1)	0.500	0.500
F000	584.0	584.0
F000'	585.41	
h,k,lmax	12,14,16	12,14,16
Nref	5599	5509
Tmin,Tmax	0.925,0.942	0.650,0.751
Tmin'	0.905	

Correction method= # Reported T Limits: Tmin=0.650 Tmax=0.751
AbsCorr = MULTI-SCAN

Data completeness= 0.984 Theta(max)= 57.651

R(reflections)= 0.0409(4690) wR2(reflections)= 0.1112(5509)

S = 1.074 Npar= 409

The following ALERTS were generated. Each ALERT has the format
test-name_ALERT_alert-type_alert-level.
Click on the hyperlinks for more details of the test.

Alert level C

```
PLAT220_ALERT_2_C NonSolvent   Resd 1  C   Ueq(max)/Ueq(min) Range        3.1 Ratio
PLAT241_ALERT_2_C High   'MainMol' Ueq as Compared to Neighbors of       C33 Check
PLAT906_ALERT_3_C Large K Value in the Analysis of Variance ......      2.675 Check
PLAT911_ALERT_3_C Missing FCF Refl Between Thmin & STh/L=    0.600        15 Report
                   0  1  0,    1  1  1,    2 -3  2,    1 -1  2,    2 -1  3,    1  1  3,
                   2  2  3,    6 -5  5,    7 -5  5,    9 -3  5,    4 -7  6,    6 -7  6,
                   6 -6  7,    8 -4  7,    6 -2  9,
```

Alert level G

```
ABSMU01_ALERT_1_G  Calculation of _exptl_absorpt_correction_mu
                   not performed for this radiation type.
PLAT002_ALERT_2_G Number of Distance or Angle Restraints on AtSite          2 Note
PLAT003_ALERT_2_G Number of Uiso or U(i,j)  Restrained non-H-Atoms          6 Report
PLAT154_ALERT_1_G The s.u.'s on the Cell Angles are Equal ..(Note)      0.002 Degree
PLAT172_ALERT_4_G The CIF-Embedded .res File Contains DFIX Records          1 Report
PLAT178_ALERT_4_G The CIF-Embedded .res File Contains SIMU Records          1 Report
PLAT186_ALERT_4_G The CIF-Embedded .res File Contains ISOR Records          1 Report
PLAT188_ALERT_3_G A Non-default SIMU Restraint Value has been used     0.0100 Report
PLAT230_ALERT_2_G Hirshfeld Test Diff for    C1         --C2        .      8.0 s.u.
PLAT301_ALERT_3_G Main Residue  Disorder .............(Resd     1)          7% Note
PLAT367_ALERT_2_G Long?  C(sp?)-C(sp?) Bond  C3         - C4        .     1.54 Ang.
PLAT793_ALERT_4_G Model has Chirality at C4          (Centro SpGr)          S Verify
PLAT793_ALERT_4_G Model has Chirality at C5          (Centro SpGr)          R Verify
PLAT793_ALERT_4_G Model has Chirality at C6          (Centro SpGr)          R Verify
PLAT860_ALERT_3_G Number of Least-Squares Restraints .............         73 Note
PLAT912_ALERT_4_G Missing # of FCF Reflections Above STh/L=   0.600        75 Note
PLAT933_ALERT_2_G Number of HKL-OMIT Records in Embedded .res File          1 Note
                   5 -9  7,
PLAT969_ALERT_5_G The 'Henn et al.' R-Factor-gap value ..........       4.392 Note
                 Predicted wR2: Based on SigI**2  2.53 or SHELX Weight 10.36
PLAT978_ALERT_2_G Number C-C Bonds with Positive Residual Density.         13 Info
```

---

0 **ALERT level A** = Most likely a serious problem - resolve or explain
0 **ALERT level B** = A potentially serious problem, consider carefully
4 **ALERT level C** = Check. Ensure it is not caused by an omission or oversight
19 **ALERT level G** = General information/check it is not something unexpected

2 ALERT type 1 CIF construction/syntax error, inconsistent or missing data

```
 8 ALERT type 2 Indicator that the structure model may be wrong or deficient
 5 ALERT type 3 Indicator that the structure quality may be low
 7 ALERT type 4 Improvement, methodology, query or suggestion
 1 ALERT type 5 Informative message, check
```

It is advisable to attempt to resolve as many as possible of the alerts in all categories. Often the minor alerts point to easily fixed oversights, errors and omissions in your CIF or refinement strategy, so attention to these fine details can be worthwhile. It is up to the individual to critically assess their own results and, if necessary, seek expert advice.

PLATON version of 26/09/2025; check.def file version of 20/09/2025

duplicate check

No duplication found
